# Dispersion Profiles and Gene Associations of Repetitive DNAs in the Euchromatin of the Beetle *Tribolium castaneum*

**DOI:** 10.1534/g3.117.300267

**Published:** 2018-01-08

**Authors:** Josip Brajković, Željka Pezer, Branka Bruvo-Mađarić, Antonio Sermek, Isidoro Feliciello, Đurđica Ugarković

**Affiliations:** * Department of Molecular Biology, Ruđer Bošković Institute, HR-10000 Zagreb, Croatia; † Dipartimento di Medicina Clinica e Chirurgia, Università degli Studi di Napoli Federico II, I-80131, Italy

**Keywords:** satellite DNA, repetitive DNA, transcription, gene regulation, *Tribolium castaneum*

## Abstract

Satellite DNAs are tandemly repeated sequences clustered within heterochromatin. However, in some cases, such as the major TCAST1 satellite DNA from the beetle *Tribolium castaneum*, they are found partially dispersed within euchromatin. Such organization together with transcriptional activity enables TCAST1 to modulate the activity of neighboring genes. In order to explore if other *T. castaneum* repetitive families have features that could provide them with a possible gene-modulatory role, we compare here the structure, organization, dispersion profiles, and transcription activity of 10 distinct TCAST repetitive families including TCAST1. The genome organization of TCAST families exhibit either satellite-like or transposon-like characteristics. In addition to heterochromatin localization, bioinformatic searches of the assembled genome have revealed dispersion of all families within euchromatin, preferentially in the form of single repeats. Dispersed TCAST repeats are mutually correlated in distribution and are grouped in distinct regions of euchromatin. The repeats are associated with genes, are enriched in introns relative to intergenic regions, and very rarely overlap exons. In spite of the different mechanisms of repeat proliferation, such as transposition and homologous recombination, all TCAST families share a similar frequency of spreading as well as dispersion and gene association profiles. Additionally, TCAST families are transcribed and their transcription is significantly activated by heat stress. A possibility that such common features of TCAST families might be related to their potential gene-modulatory role is discussed.

Satellite DNAs are tandemly repeated sequences organized in long arrays located within pericentromeric and/or subtelomeric heterochromatin. In addition to participating in centromere and heterochromatin formation, it has been proposed that satellite DNAs might also act as regulatory elements of gene expression (Ugarković 2005; Tomilin 2008; Pezer *et al.* 2012). The argument for the possible gene-regulatory role of satellite DNAs is their transcriptional activity, in particular satellite DNA transcripts targeted by silencing mechanisms, including RNA interference, which are involved in epigenetic modifications within heterochromatin (Grewal and Elgin 2007; Fagegaltier *et al.* 2009). In addition, functional elements such as promoters and transcription factor binding sites are present within some satellite DNA sequences (Pezer and Ugarković 2009; Pezer *et al.* 2011; Vourc’h and Biamonti 2011; Bulut-Karslioglu *et al.* 2012). In order to have a potential gene-regulatory function, satellite DNA elements are expected to be distributed in the vicinity of genes within euchromatin. Such examples of satellite DNAs, which are not only located in constitutive heterochromatin but are also dispersed within euchromatin, are *Drosophila melanogaster* 1.688 (Kuhn *et al.* 2012) and *Responder* (Larracuente 2014) satellite DNAs, as well as two satellite DNAs in the red flour beetle *Tribolium castaneum* (Brajković *et al.* 2012; Feliciello *et al.* 2015a). Comprehensive analysis of satellite DNAs in the genome of migratory locust (*Locusta migratoria*) revealed mixed locations for many of them, both in heterochromatin and euchromatin (Ruiz-Ruano *et al.* 2016).

*T. castaneum* has large blocks of pericentromeric heterochromatin on all chromosomes, with no prominent heterochromatic regions detected cytogenetically on chromosome arms (Stuart and Mocelin 1995; Ugarković *et al.* 1996a). A major *T. castaneum* satellite DNA TCAST1, which comprises 35% of the genome, and a minor satellite TCAST2 are both preferentially located within pericentromeric heterochromatin of all chromosomes, but are also found in the form of short arrays or single repeats in the vicinity of genes within euchromatin (Feliciello *et al.* 2011; Brajković *et al.* 2012). The heterochromatin of *T. castaneum* is highly susceptible to heat stress, which results in the increased transcription of a major TCAST1 satellite DNA (Pezer and Ugarković 2012). Heat stress-induced TCAST1 transcripts in the form of small interfering RNAs affect histone modifications at TCAST1 homologous regions not only in heterochromatin but also within euchromatin, and in this way transiently suppress the expression of neighboring genes (Feliciello *et al.* 2015b). Such an interplay between satellite DNA repeats located within heterochromatin and euchromatin seems to be indispensable for the influence of satellite DNA on genes, and represents the first experimental proof for the gene-modulatory role of a satellite DNA. Aside from satellite DNAs, there are many studies on another class of repetitive DNAs, transposons, which are interspersed throughout the genome and were shown to affect expression of neighboring genes under standard physiological conditions in plants (Eichten *et al.* 2012; Wang *et al.* 2013), mammals (Rebollo *et al.* 2011), and *Drosophila* (Sienski *et al.* 2012), as well as in response to abiotic stress (Makarevitch *et al.* 2015).

The genome of the beetle *T. castaneum* has been sequenced (Richards *et al.* 2008), but due to technical difficulties associated with sequencing and assembling highly repetitive regions, only 0.3% of the assembled 152 Mb genome consists of a major TCAST1 satellite (Wang *et al.* 2008). Since, based on experiments, the genome size of *T. castaneum* has been calculated as 204 Mb (Alvarez-Fuster *et al.* 1991) and TCAST1 as a major heterochromatin component encompasses up to 60 Mb, it appears that the pericentromeric heterochromatin is almost completely excluded from the assembled *Tribolium* genome. Therefore, the *T. castaneum* assembled genome is comprised mostly of euchromatin. Bioinformatic analysis has enabled the identification of major classes of repetitive elements including highly repetitive families (HighA) (Wang *et al.* 2008). Additional repetitive families were identified by genome-wide analysis of tandem repeats in the assembled *T. castaneum* genome (Pavlek *et al.* 2015). They are organized in numerous arrays of more than five repeats and are almost exclusively located within euchromatin. Unlike these euchromatic tandem repeats, the repetitive families belonging to the HighA class (Wang *et al.* 2008) are not characterized regarding structure, origin, and organization, except for the previously described major TCAST1 and minor TCAST2 satellite DNAs (Feliciello *et al.* 2011, 2015a). Based on the role of TCAST1 repeats in the modulation of neighboring gene expression (Feliciello *et al.* 2015b), it can be hypothesized that some other HighA class repetitive DNA families might have distinct structural and organizational characteristics, similar to TCAST1, which could implicate their potential functional significance and/or gene-regulatory role.

In this study, we characterize eight additional highly repetitive DNAs from *T. castaneum*, named TCAST3–TCAST10, which were previously identified by bioinformatic analysis of *T. castaneum* genomic data (Wang *et al.* 2008). We analyze their sequence characteristics, genomic content, and organization, as well as distribution (particularly relative to genes), and compare them with the characteristics of the previously described satellite families TCAST1 and TCAST2. Our study reveals similarities in genomic organization and distribution of all TCAST families along *T. castaneum* chromosomes, as well as in their transcriptional activation and in their positioning relative to the genes. In addition, molecular mechanisms responsible for their dispersion throughout the euchromatin are proposed and the potential gene-modulatory role of TCAST repetitive DNAs is discussed.

## Materials and Methods

### Database screening

BLASTN version 2.2.22+ was used to screen the NCBI refseq genomic database of *T. castaneum*. The program was optimized to search for highly similar sequences (megablast) to the query sequences corresponding to the repeats of TCAST repetitive DNAs. Genes flanking TCAST repeats were found automatically by NCBI blast. Sequences corresponding to hits, as well as their flanking regions, were analyzed by dot plot (http://www.vivo.colostate.edu/molkit/dnadot/), using standard parameters (window size 9 and mismatch limit 0) or more relaxed conditions (window size 11 and mismatch limit 1), to determine the exact start and end sites of specific TCAST-like elements. Annotation of TCAST elements on *T. castaneum* chromosomes (Tcas_3.0 assembly) was performed using the program Geneious 8.0.14. TCAST elements were analyzed in detail for the presence of hallmarks, such as terminal inverted repeats (TIRs) and target site duplications, with the aid of the Gene Jockey sequence analysis program (Apple Macintosh). Secondary structures were determined using the default parameters of the MFOLD program available online (http://mfold.rna.albany.edu/?q=mfold) (Zuker 2003). Repbase, a reference database of eukaryotic repetitive DNAs, was screened using WU-BLAST (Kohany *et al.* 2006).

### Phylogenetic analysis

Sequence alignment was performed using the MUSCLE algorithm (Edgar 2004) combined with manual adjustment. All sequences were included in the alignment, with the exception of the ones that did not at least partially overlap with other sequences. Gblocks was used to eliminate poorly aligned positions and divergent regions of the alignments (Talavera and Castresana 2007). Markov chain Monte Carlo Bayesian searches were performed in MrBayes v. 3.1.2. (Huelsenbeck and Ronquist 2001) under the best-fit models (two simultaneous runs, each with four chains; 3 × 10^6^ generations; sampling frequency 1 in every 100 generations; and majority rule consensus trees constructed based on trees sampled after burn-in) and branch support was evaluated through posterior probabilities. Pairwise sequence diversity (uncorrected p-distance) was calculated using the MEGA 6.06 software (Tamura *et al.* 2013).

### Simulations and statistical analyses

Bedtools shuffle (Quinlan and Hall 2010) was used to randomly permute the locations of nonexonic TCAST elements across the Tcas_3.0 assembled genome. Assembly gaps, exons, and unlocalized and unplaced scaffolds were excluded as locations in which TCAST elements should not be placed. Shuffled intervals were allowed to overlap as we found several TCAST elements overlapping in our data (Supplemental Material, Table S1 in File S3). We performed 1000 such random placements and for each TCAST element counted how many times the reported number of elements (and the corresponding base pair count) was met or surpassed in simulation, in both introns and intergenic regions.

We used bedtools shuffle and bedtools closest (Quinlan and Hall 2010) to randomly redistribute intergenic TCAST elements across the genome and calculate their distance to the nearest genes. For each of the 10 TCAST elements, 100 random placements were performed excluding assembly gaps and genes. We compared the distributions of distance to genes between our data and simulated results using a two-sample Kolmogorov–Smirnov (K–S) test in R. TCAST distribution among and within chromosomes was analyzed using a linear regression model that was performed in R using the *lm* function.

### DNA extraction, PCR analysis, and dot blot hybridization

DNA was extracted from adult insects of the GA2 strain, used for genome sequencing, using the DNeasy Blood & Tissue Kit (QIAGEN). Primers used for PCR amplification of TCAST elements where designed using Primer3Plus software and are listed in Table S2 in File S3. A specific touchdown PCR program was designed to amplify TCAST multimers: 3 min denaturation at 94°; 15 cycles of 30 sec at 94°, 30 sec at 57–0.25°, and 1 min at 72°; 25 cycles of 30 sec at 94°, 30 sec at 52°, and 1 min at 72°; and a final extension of 3 min at 72°. Amplification products were sequenced using an ABI Prism 310 (Applied Biosystems).

Dot blot hybridization was performed as described in Feliciello *et al.* (2015a). *T. castaneum* DNA serially diluted with denaturing buffer was dot-blotted in the range from 25 to 400 ng on a nylon membrane and fixed by baking at 80°. TCAST3–10 repeats amplified by PCR from genomic DNA, with primers listed in Table S3 in File S3, were dot-blotted in the range from 0.05 to 1 ng, and used as a positive control and as a calibration curve. The same primers were used to prepare biotinylated probes using amplified TCAST repeats as templates. Hybridization and washing was performed under high-stringency conditions.

### RNA isolation and reverse transcription

RNA was isolated from *Tribolium* adult beetles of the GA2 strain (approximate weight of sample, 20 mg) using the RNeasy Mini kit (QIAGEN) according to the manufacturer’s instructions. RNA was additionally digested with Turbo DNase (Ambion), quantified with the Quant-IT RNA assay kit using a Qubit fluorometer (Invitrogen), quality-checked on a gel, and PCR-checked for the presence of TCAST DNA. RNA (∼1 µg) was reverse transcribed using a PrimeScript RT-PCR Kit (Takara) in 10 µl reactions using random primers. Negative controls without reverse transcriptase were used for all samples.

### Quantitative real-time PCR (qPCR) analysis

cDNA samples were amplified using an Applied Biosystem ABI7300 Real-Time PCR System. The qPCR reactions were done in triplicate, in 50 µl reaction volumes, with 0.5 µM specific primers, 2× Power SYBR Green PCR Master mix (Applied Biosystems), and 30 ng of cDNA. Primers for the expression analysis of TCAST families are listed in Table S3 in File S3. To correct for the differences in sample composition and in the yield of the reverse transcription reaction, ribosomal protein S18 (RPS18) was used for normalization (Lord *et al.* 2009; Toutges *et al.* 2010). The thermal cycling conditions were as follows: 50° for 2 min, 95° for 7 min, 95° for 15 sec, and 60° for 1 min for 40 cycles, followed by a dissociation stage: 95° for 15 sec, 60° for 1 min, 95° for 15 sec, and 60° for 15 sec. Amplification specificity was confirmed by dissociation curve analysis. Specificity of amplified product was additionally tested on agarose gel. Control without template was included in each run. Postrun data were analyzed using LinRegPCR software v.11.1. (Ruijter *et al.* 2009, 2013). The software enables calculation of the starting concentration of the amplicon (“No value”), which is expressed in arbitrary fluorescence units and is calculated by taking into account PCR efficiency and baseline fluorescence. No value determined for each technical replicate was averaged. Averaged No values were divided by No values of endogenous control for normalization. Statistical analyses of qPCR data were done using GraphPad v.6.01. Normalized No values were compared using an unpaired Student’s *t*-test, which compares the mean of two unmatched groups.

### Data availability

The authors state that all data necessary for confirming the conclusions presented in the article are represented fully within the article.

## Results

### Characteristics of TCAST repetitive families’ origins, genomic organization, and content

The repetitive DNA families TCAST3–TCAST10 were previously identified by bioinformatic analysis of the *T. castaneum* genome as highly repetitive DNAs, which occupy > 0.1% of the genome, and were annotated as *R* = 35 (TCAST3), *R* = 38 (TCAST4), *R* = 45 (TCAST5), *R* = 56 (TCAST6), *R* = 117 (TCAST7), *R* = 176 (TCAST8), *R* = 273 (TCAST9), and *R* = 2028 (TCAST10) (Wang *et al.* 2008). Satellite families TCAST1 and TCAST2 were also identified as members of the same class, but were also experimentally characterized (Feliciello *et al.* 2011, 2015a).

**Figure 1:**
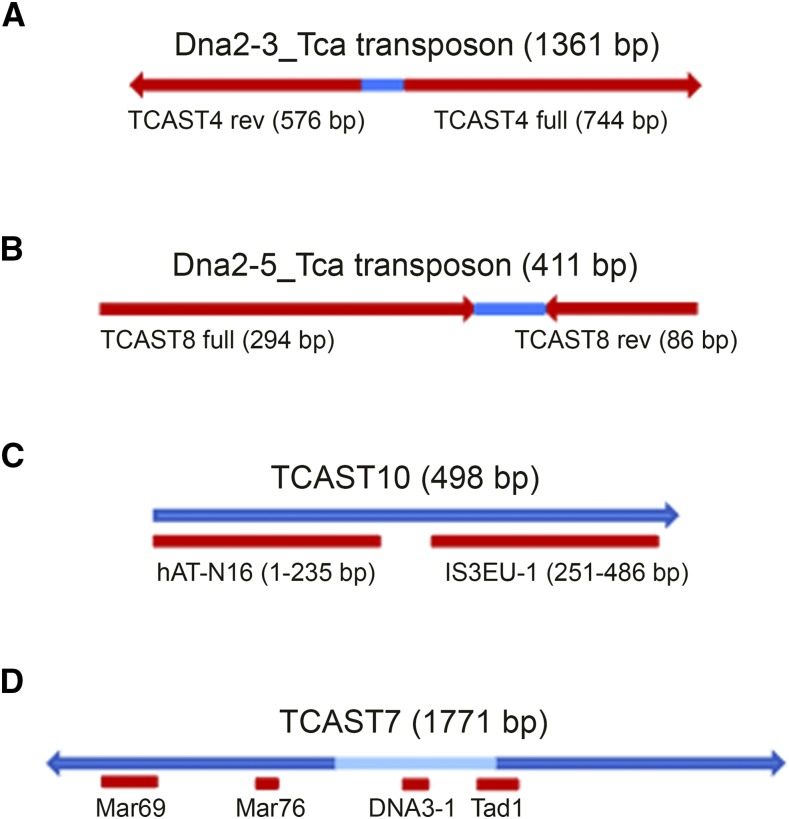
Schematic representation of the Dna2-3_Tca transposon (A), which has 91% similarity to the full-size TCAST4 repeat of 744 bp. The transposon also contains the partial 576 bp-long reverse (rev) complement of TCAST4 (146–740 bp). TCAST4-like terminal inverted repeats of a transposon have similarity of 92% and are separated by 41 bp TCAST4-unrelated sequence (blue). (B) The Dna5-3_Tca transposon, which has 99% similarity to the full-size TCAST8 repeat of 294 bp. The transposon also contains the partial 86 bp long reverse complement of TCAST4 (208–294 bp). TCAST8-like terminal inverted repeats of transposon have similarity of 86% and are separated by 31 bp TCAST8-unrelated sequence (blue). (C) The TCAST10 repeat, which shares similarity with DNA transposons hAT-N16_Lmi [1–235 nucleotides (nt)] and IS3EU-1_TC [251–486 nucleotides (nt)]. (D) TCAST7 repeat, composed of two 735 bp-long, inverted repeats, which share mutual similarity of 90% and are separated by 300 bp of unrelated sequence (light blue). Short segments of TCAST7 sequence (indicated by red rectangles) share similarity with transposons Mariner-69_HSal (120–238 nt), Mariner-76_HSal (428–468), Dna-3-1_DPur (750–804 nt), and retrotransposon nonLTR/Tad1 (1002–1085 nt).

Repetitive families TCAST1–TCAST10 are characterized as A+T-rich 294–1771 bp-long monomer repeats (Table 1), which do not have mutual sequence similarity. To disclose the possible origin of TCAST repeats and their relationship to the other repetitive families, we searched for elements similar to the TCAST repeats within Repbase (http://www.girinst.org/repbase/). The search revealed no significant similarity of any deposited element with TCAST3, TCAST5, TCAST6, or TCAST9 repeats. However, TCAST4 showed a similarity of 91% along the whole sequence to a part of the Dna2-3_Tca mariner nonautonomous transposon, while TCAST8 was 99% similar along the whole repeat to a part of the Dna2-5_Tca mariner nonautonomous transposon (Figure 1, A and B). TCAST10 showed a similarity of 76% along the first half of the sequence to the hAT DNA transposon, while the second half had 86% similarity to the IS3EU-TC DNA transposon from *T. castaneum* (Figure 1C). The two halves of TCAST10 did not have significant sequence similarity. TCAST7 had four short regions, 40–120 bp-long, similar in sequence to two Mariner-type DNA transposons, as well as to DNA3-1 nonautonomous transposon and Tad1 non-LTR retrotransposon (Figure 1D). The TCAST7 sequence is complex, composed of two 730 bp-long TIRs of 90% similarity, separated by 300 bp of unrelated sequence (Figure 1D), and in organization resembles satellite DNAs from the related *Tribolium* species *T. madens* (Ugarković *et al.* 1996b) and *T. brevicornis* (Mravinac *et al.* 2005). A high similarity of TCAST1 with non-LTR retrotransposon CR1-3_TCa was reported previously (Brajković *et al.* 2012), while a similarity of 70% along the AT-rich part of the TCAST2 repeat with DNA transposon Crypton-2_Tca was not considered as significant (Feliciello *et al.* 2015a). Sequence similarity to transposons indicates that TCAST1, TCAST4, TCAST7, TCAST8, and TCAST10 might be of transposon origin. TCAST7 and partially TCAST1 have an additional hallmark of transposons, namely the TIRs, which are indispensable for the potential interaction with transposase and transposon mobility (Capy *et al.* 1998).

**Table 1:** Characteristics of dispersed repetitive DNA elements TCAST1–TCAST10

	TCAST1	TCAST2	TCAST3	TCAST4	TCAST5	TCAST6	TCAST7	TCAST8	TCAST9	TCAST10
Mon. length/bp	360	359	581	744	417	814	1771	294	386	498
A+T %	73.3	71.5	68.7	74.6	68.3	67.2	69.7	70.7	73.8	72.7
No. elements	68	324	384	154	360	137	108	339	136	372
No. multimers	18	2	12	24	13	2	1	2	4	7
No. aligned monomers	77	307	360	146	343	122	88	303	136	353
Av. divergence %	6.7/12.7	11	15,8	20.1	12.7	10.2	13.6	13.9	11.7	14.4

Mon., monomer; No., number; Av., average.

**Figure 2:**
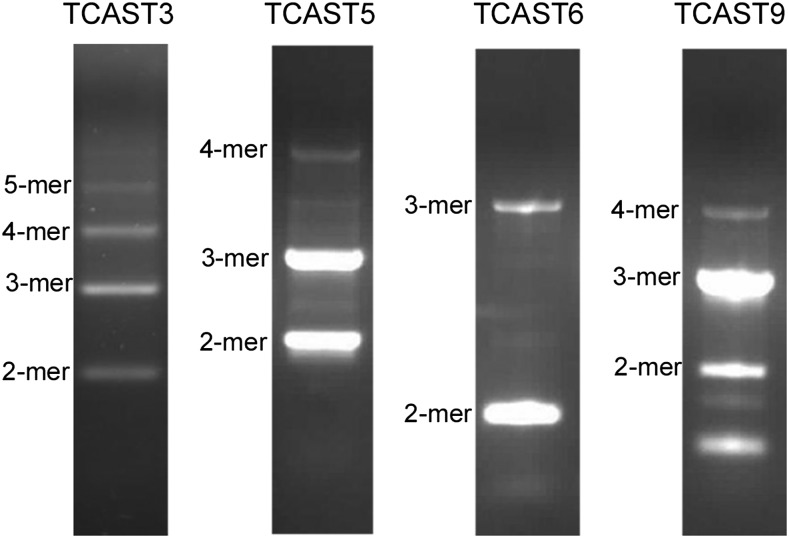
Polymerase chain reaction amplification using specific primers that amplify tandemly arranged dimers and larger multimers, and subsequent separation of products by agarose gel electrophoresis. Sequencing of products reveals satellite DNA-like tandem organization of TCAST3, TCAST5, TCAST6, and TCAST9 repeats within the *T. castaneum* genome.

Regarding genome organization, repetitive families can be categorized as satellite-like DNAs with a predominant tandem arrangement of repeats or as transposon-like families characterized by an interspersed organization of repeats. TCAST1 and TCAST2 were previously classified as satellite DNAs (Feliciello *et al.* 2011, 2015a). In order to check experimentally if the repetitive families TCAST3–TCAT10 might belong to the tandemly arranged satellite-like DNAs, we designed pairs of primers starting from the same position of each repetitive unit but in the opposite orientation, which enables the amplification of dimers and longer multimers. PCR was performed using a specifically designed program to amplify multimers (see *Materials and Methods*). A regular ladder-like pattern characteristic for tandemly arranged repeats was detected using TCAST3-, TCAST5-, TCAST6-, and TCAST9-specific primers, and sequencing confirmed that the bands correspond to multimers of corresponding repeats (Figure 2). For TCAST4, TCAST7, TCAST8, and TCAST10 repetitive families, we were not able to get products corresponding to dimers and longer multimers using various PCR amplification conditions. Based on the obtained amplification patterns, we classified TCAST3, TCAST5, TCAST6, and TCAST9 as tandemly repeated satellite-like families. Due to sequence similarity to transposons and an absence of tandem organization of repeats, TCAST4, TCAST7, TCAST8, and TCAST10 are considered to be interspersed transposon-like repetitive families.

**Figure 3:**
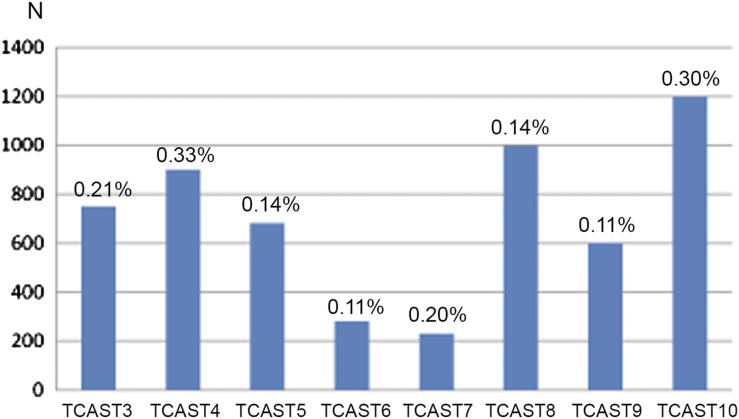
Genomic content indicated as a percentage of the total genome and copy number (N) of repetitive families TCAST3–TCAST10 in the *T. castaneum* GA2 strain, estimated by dot blot hybridization.

The genomic content of repetitive families TCAST1 and TCAST2 in *T. castaneum* strain GA2 is 35 and 0.38%, respectively, of the total genomic DNA, as determined experimentally (Feliciello *et al.* 2011, 2015a). This corresponds to 2 × 10^5^ copies of TCAST1 and 2 × 10^3^ copies of TCAST2, respectively, per haploid genome. Here, we examined the genomic content and copy number of families TCAST3–TCAST10 in the GA2 strain using dot blot hybridization. The results reveal that TCAST families comprise between 0.11 and 0.33% of the genome (Figure 3). Considering that *T. castaneum* has a genome size of 204 Mb (Alvarez-Fuster *et al.* 1991), this corresponds to full-size copy number ranging from 230 for TCAST7 to 1.2 × 10^3^ for TCAST10 (Figure 3).

### Characteristics of dispersed TCAST repeats copy number and structure

In order to find elements similar to TCAST3–TCAST10 repeats, we screened the NCBI refseq_genomic database of *T. castaneum* using the alignment program BLASTN version 2.2.22+. Only genomic sequences with ≥ 40% of repeat sequence length and > 80% identity to the query sequences were considered for further analysis. This analysis revealed dispersed elements similar to TCAST3–TCAST10 ranging in number from 108 TCAST7-related elements to 384 TCAST3-related elements (Table 1). Considering the total genomic copy number of TCAST3–TCAST10 estimated experimentally (Figure 3), it seems that a significant portion of TCAST repeats are also present within the unassembled heterochromatic part of the genome. To see if there is a difference in the frequency of dispersed elements between families originating from transposons (TCAST1, TCAST4, TCAST7, TCAST8, and TCAST10) and families of nontransposon origin (TCAST2, TCAST3, TCAST5, TCAST6, and TCAST9), we compared the two groups using the unpaired Student’s *t*-test. The results reveal a mean number of dispersed elements of 208.20 ± 138.36 and 267.80 ± 121.68 for the first and second groups, respectively. The corresponding *P*-value of 0.4901 shows no statistically significant difference in the frequency between the two groups of repetitive families, indicating that the frequency of spreading is not significantly related to the origin of repeats.

The structure of all dispersed elements was further analyzed to reveal if they are composed of single repeats or multimers. The number of multimers found in each TCAST family is shown in Table 1. Only 3% of TCAST3 and TCAST5 elements, as well as up to 2% of TCAST6, TCAST7, and TCAST9, are multimers in the form of partial or full-size dimers composed of monomers in direct orientation. In addition, among TCAST5 elements, there is a single 3-mer and a 10-mer, while among TCAST9 elements there is a single 5-mer and a 7-mer. TCAST4, TCAST8, and TCAST10 elements also have a low percentage of multimers exclusively in the form of dimers, which are composed of partial or full-sized monomers in inverted orientation separated by unrelated sequence, resembling TIR DNA transposons in organization. Previous results showed that all TCAST2 elements are dispersed in the form of single repeats except for one dimer (Feliciello *et al.* 2015a), while 25% of dispersed TCAST1 satellite elements are in the form of tandem repeats, up to a tetramer (Brajković *et al.* 2012). The results reveal that, in spite of probably different origins, all dispersed TCAST elements exhibit preferential organization in the form of single repeats. In contrast to TCAST3, TCAST5, TCAST6, and TCAST9, where multimers are exclusively composed of repeats in direct orientation, the existence of complex multimers composed of inverted repeats in TCAST4, TCAST8, and TCAST10 indicates a different origin of the two groups of TCAST repetitive families.

### Phylogenetic analysis of dispersed TCAST repeats

In order to see if there is any sequence clustering of TCAST elements that could indicate a difference in the homogenization at the level of local array, chromosome, or among different chromosomes, sequence alignment and phylogenetic analyses were performed. Previous analysis of dispersed TCAST1 and TCAST2 repeats showed no significant sequence clustering on phylogenetic trees, which indicates a similar level of sequence homogenization among nearby and distant repeats (Brajković *et al.* 2012; Feliciello *et al.* 2015a). Since repeats within TCAST3–TCAST10 families differ significantly in size, the alignment and phylogenetic analyses were performed on those repeats that mutually overlap in their sequences, while the others were excluded from the analysis. The number of such repeats for each TCAST family is shown in Table 1. The alignments were additionally adjusted using Gblocks (File S1), and phylogenetic analyses performed by the Bayesian method gave phylogenetic trees with generally very weak resolution within all eight TCAST families (File S2). On the TCAST3 tree there are several supported groups, most of them composed of only two elements, which usually derive from different chromosomes with only one group containing two tandemly arranged monomers (File S2a). On the TCAST4 tree there are several supported clusters, composed mostly of repeats from different chromosomes (File S2b). However, the clustering of neighboring repeats organized in inverted orientation within 19 multimers is not observed. The TCAST6 tree has three supported clusters composed of five to six repeats from different chromosomes and several clusters with only two repeats mostly from different chromosomes (File S2d). In the TCAST7, TCAST8, and TCAST10 trees there are several supported clusters composed of a few repeats, either from the same or from different chromosomes (File S2e, f, h).

Significant clustering is characteristic for tandem repeats within 3-mers and 10-mers in the TCAST5 phylogenetic tree (File S2c), as well as for the 7-mers and 5-mers in the TCAST9 tree (File S2g). The remaining repeats in the TCAST5 and TCAST9 trees show very weak grouping, with several supported groups of two to three elements mostly from different chromosomes. Therefore, the dispersed repeats of all TCAST satellites seem to exhibit a type of evolution characterized by a similar level of sequence homogenization between nearby and distant repeats, and a general absence of sequence clustering. The average sequence divergence is between 10.2% for TCAST6 and 20.1% for TCAST4 (Table 1), and this is comparable to the previously determined divergence of TCAST1 and TCAST2 dispersed repeats (Table 1). The clustering of monomers within longer multimers such as 5-mers and 7-mers in TCAST9, as well as 10-mers and 3-mers in TCAST5, could be related to the relatively recent insertion of multimeric elements from heterochromatin, where tandem repeats are efficiently homogenized (Feliciello *et al.* 2005, 2006). Such multimers seem to be unstable within euchromatin and, due to internal recombination, exhibit copy number polymorphism among *T. castaneum* populations as demonstrated for TCAST1 multimers (Feliciello *et al.* 2015b). The final outcome of this process seems to be a loss of multimeric tandem repeats from euchromatin and a prevalence of single dispersed elements, which are characteristic for all TCAST families.

### The pattern of dispersion of TCAST repeats among and within chromosomes

To get more detailed insight into the dispersion patterns of TCAST elements, we analyzed their distribution among, as well as within, *T. castaneum* chromosomes. TCAST elements are present within the euchromatin of all 10 chromosomes and their distribution among chromosomes is shown in Table 2. All dispersed TCAST elements are present in the lowest number of copies on chromosome 1 (X) and in the highest copy number on chromosome 3, except for TCAST7, which has the highest copy number on chromosome 10. To test if the distribution of TCAST elements on chromosomes is preferentially associated with chromosome size or number of genes per chromosome, we performed a simple linear model fit. We found that the number of TCAST elements overall is a function of chromosome size (*R*^2^ = 0.813; *P* = 3.6 × 10^−4^; Table 2). However, when looking at each of the 10 elements independently, the number of TCAST7 appears unpredictable in relation to the size of the chromosomes (*R*^2^ = 0.053; *P* = 0.524). TCAST3 chromosomal distribution shows a possible independence of chromosome length (*R*^2^ = 0.414; *P* = 0.045). Although we find that the number of genes on chromosomes can be predicted by chromosome size as well (*R*^2^ = 0.569; *P* = 0.012), the distribution of all TCAST elements does not appear correlated to it (*R*^2^ = 0.194; *P* = 0.202). However, there is some possibility, indicated by *P* < 0.05, that the genomic distributions of TCAST8 and TCAST10 are associated with the number of genes (*R*^2^ = 0.415; *P* = 0.045 and *R*^2^ = 0.414; *P* = 0.045, respectively). The results reveal that the size of a chromosome is a reliable predictor of number of TCAST elements as well as of number of genes, but that the correlation between the number of TCAST elements and genes is generally weak and statistically insignificant.

**Table 2:** The distribution of TCAST elements and genes on chromosomes

	TCAST1	TCAST2	TCAST3	TCAST4	TCAST5	TCAST6	TCAST7	TCAST8	TCAST9	TCAST10	All TCAST	All Genes	Chr Size (bp)*^a^*
Chr1	2	5	3	5	3	5	0	7	5	2	37	698	7.277.635
Chr2	4	41	28	13	37	14	6	33	11	33	219	1.337	14.518.415
Chr3	18	83	97	52	99	46	30	100	44	112	680	1.614	28.591.480
Chr4	4	32	19	8	30	11	1	26	5	31	167	1.245	12.094.384
Chr5	3	26	21	10	25	12	0	32	6	32	167	1.550	14.335.781
Chr6	4	19	25	13	23	3	6	13	11	24	141	898	8.976.827
Chr7	5	28	25	9	32	10	2	31	8	39	189	1.671	15.432.854
Chr8	6	31	44	15	41	12	8	40	11	39	247	1.361	13.521.898
Chr9	16	40	53	14	44	19	13	42	14	44	299	1.243	15.459.655
Chr10	6	19	69	15	26	5	42	15	21	16	234	502	7.486.040
*P* chr size	**0.011**	**1.5E−05**	**0.045**	**0.002**	**5.4E−05**	**1.2E−05**	0.524	**1.3E−05**	**0.015**	**1.2E−05**	**3.6E−04**	**0.012**	
*P* gene no.	0.387	0.059	0.751	0.346	0.096	0.090	0.360	**0.045**	0.687	**0.045**	0.202		

Correlation between number of TCAST elements and chromosome size as well as gene number is expressed in *P*-values, and statistically significant values are indicated in bold. Chr, chromosome; no. number.

a Assembly gaps are excluded.

**Figure 4:**
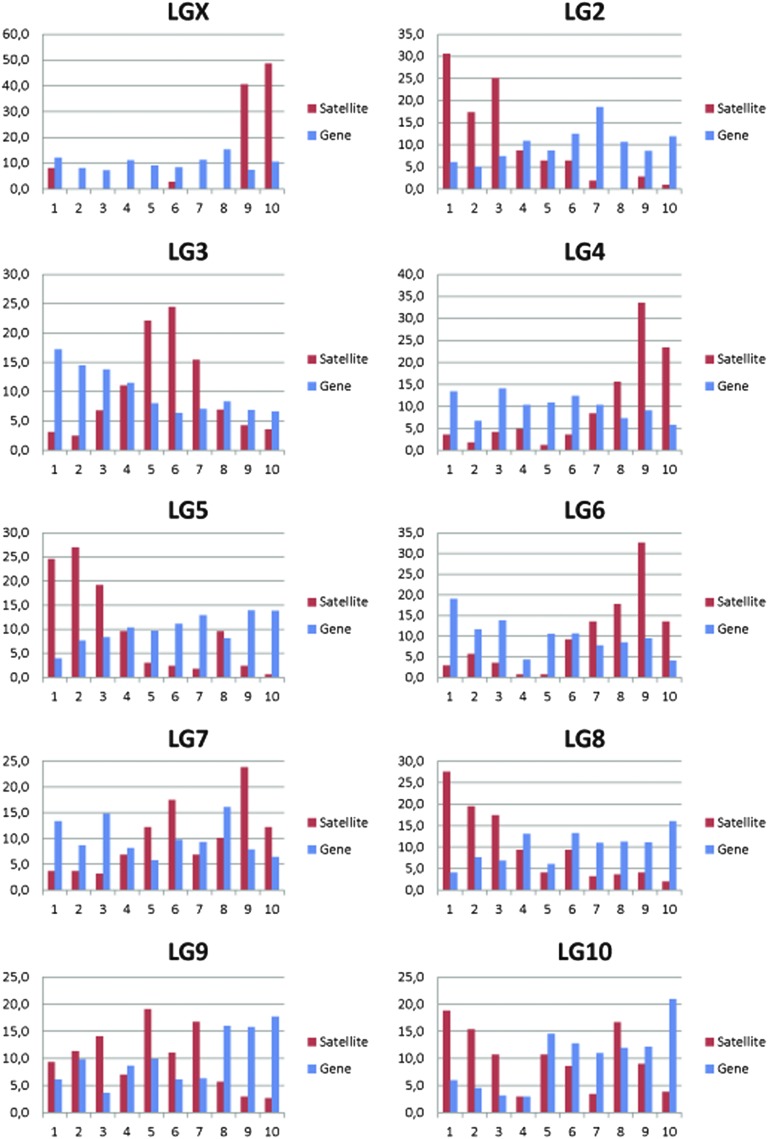
The histograms show the percentage of all dispersed TCAST elements (red), as well as the percentage of genes (blue) along the length of 10 *T. castaneum* chromosomes. The percentage is calculated for the intervals corresponding to 1/10 of length of each chromosome. LG, linkage group.

In addition to studying the distribution of TCAST elements among chromosomes, we were interested in analyzing their distribution within chromosomes, along the length of each chromosome. Such analyses can reveal if TCAST elements are grouped in some specific chromosomal regions or if they are equally dispersed along the chromosome length. The results can also show if different TCAST elements are mutually correlated in their distribution, and if there is any correlation in the distribution of TCAST elements and genes along the chromosomes. To analyze the distribution of TCAST elements within chromosomes, we examined their frequency along the regions corresponding to 1/10 of each chromosome length as well as along intervals corresponding to 1 Mb size. The histograms showing the percentage of TCAST elements as well as the percentage of genes along the length of 10 *T. castaneum* chromosomes, within intervals of 1/10 chromosomes’ length, are presented in Figure 4. The results reveal a preferential grouping of TCAST elements in some regions of chromosomes; for example, almost 80–90% of TCAST elements on chromosomes X (1), 4, and 6 are within a cluster at a 3′ chromosome site, TCAST elements on chromosomes 2, 5, and 8 are preferentially clustered at a 5′ site of the chromosome, while on chromosome 3 they are grouped in the middle. Along chromosomes 7, 9, and 10, TCAST elements are grouped within two to three clusters.

The grouping of all TCAST elements in distinct chromosomal regions indicates that different TCAST elements are mutually correlated in distribution and have similar profiles of dispersion along chromosomes. To confirm this, we analyzed the colocalization of different TCAST elements along each chromosome based on data of distribution of each TCAST family along each chromosome (Table S4 in File S3). The results reveal a statistically significant positive correlation in the distribution of different TCAST elements (*R* > 0.700 and *P* < 0.05) along all chromosomes; for example, on chromosomes 1 (X), 2, 3, and 5, all TCAST families are colocalized in the same regions and significantly correlated in distribution. The exceptions are the TCAST1 and TCAST7 families, which due to low copy number, did not exhibit a statistically significant correlation with the remaining TCAST families. On chromosomes 4, 6, 7, 8, 9, and 10, not all TCAST families are mutually correlated in distribution, but some of them are colocalized (*e.g.*, on chromosome 4 TCAST families 2, 3, 8, and 10 are colocalized, as well as families 4, 5, and 9). On chromosomes 6, 7, 8, 9, and 10, several TCAST families, usually the most abundant, also exhibit significant colocalization on chromosomes (*R* > 0.700 and *P* < 0.05). In a similar manner, we analyzed the colocalization of all TCAST elements and genes within distinct regions along all chromosomes, either at 1 Mb intervals or regions corresponding to 1/10 of a chromosomes’ length. The analysis revealed a very weak and statistically insignificant negative correlation in the distribution between TCAST elements and genes along most chromosomes, (*R* > −0.500 and *P* > 0.05) except on chromosomes 5, 8, and 9, where a correlation was significantly negative (R < −0.68 and *P* < 0.05).

Uneven distribution of TCAST families and their mutual correlation in dispersion among as well as along chromosomes results in the clustering of different TCAST repeats in distinct chromosomal regions. However, there is no statistically significant correlation in the distribution of TCAST repeats and genes among or along most chromosomes, which reveals that TCAST repeats are not preferentially clustered in gene-poor regions, as would be expected by random insertion.

### Positioning of dispersed TCAST repeats relative to the genes

We analyzed the genomic positions of TCAST repeats, as well as distances of intergenic elements to the nearest gene, within the assembled *T. castaneum* genome (Tcas_3.0). The analysis revealed nine TCAST elements that partially overlap exons at untranslated or coding regions (Table S5 in File S3), while all other repeats are distributed within introns (1416 elements) and intergenic regions (957 elements; Table 3). The portion of elements located in introns considering their size in base pair ranges from 55.5% for TCAST9 to 64.3% for TCAST3 (Table 3). From 1000 simulations, we calculated the probability at which the reported or higher size in base pair, or number of TCAST elements, is observed by chance within introns and intergenic regions. The calculation revealed a statistically significant overrepresentation of TCAST3 repeats in introns (*P* = 0.007), while for other TCAST families no significant enrichment within introns or intergenic regions was discerned. However, if all TCAST elements are taken into account, their statistically significant overrepresentation within introns is calculated (*P* = 0.005; Table 3).

**Table 3:** Genomic position of dispersed TCAST elements and their expectancy at random

	Base Pair Count					Probability*^a^* by Chance in:	
	Intergenic	Intronic	Exonic*^b^*	Total	% in Introns	Introns	Intergenic Regions
TCAST1	12,416 (32)	15,558 (36)	—	27,974 (68)	55.6 (52.9)	0.624 (0.843)	0.376 (0.222)
TCAST2	35,283 (130)	50,411 (193)	234 (1)	85,928 (324)	58.7 (59.6)	0.434 (0.325)	0.566 (0.705)
TCAST3	72,876 (144)	133,882 (237)	1,481 (3)	208,239 (384)	64.3 (61.7)	**0.007 (0.041)**	0.993 (0.973)
TCAST4	51,800 (60)	89,061 (93)	923 (1)	141,784 (154)	62.8 (60.4)	0.074 (0.197)	0.926 (0.841)
TCAST5	53,851 (141)	78,164 (218)	336 (1)	132,351 (360)	59.1 (60.6)	0.357 (0.196)	0.643 (0.836)
TCAST6	36,452 (52)	64,027 (83)	1,286 (2)	101,765 (137)	62.9 (60.6)	0.067 (0.180)	0.933 (0.855)
TCAST7	65,928 (40)	103,028 (67)	1,633 (1)	170,589 (108)	60.4 (62.0)	0.101 (0.081)	0.899 (0.947)
TCAST8	45,216 (151)	56,919 (188)	—	102,135 (339)	55.7 (55.5)	0.754 (0.866)	0.246 (0.157)
TCAST9	30,531 (57)	38,107 (79)	—	68,638 (136)	55.5 (58.1)	0.623 (0.532)	0.377 (0.534)
TCAST10	47,373 (150)	67,330 (222)	—	114,703 (372)	58.7 (59.7)	0.405 (0.324)	0.595 (0.715)
All TCAST	450,656 (957)	696,194 (1,416)	5,893 (9)	1,152,743 (2,382)	60.4 (59.4)	**0.005 (0.054)**	0.996 (0.950)

Values in parentheses denote number of TCAST elements or calculations based on it.

a Probability at which the reported or higher base pair count (number of elements) is observed by chance; calculated from 1000 simulations. Statistically significant values are indicated in bold.

b Any overlap with exon was considered a hit.

**Figure 5:**
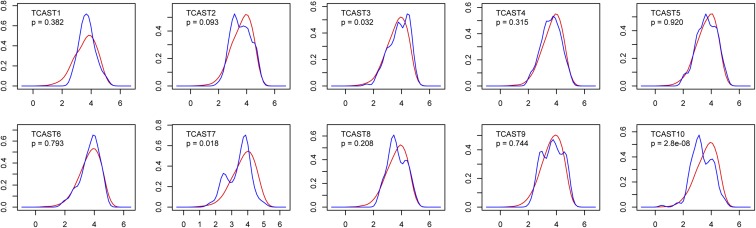
Distances of intergenic TCAST elements to the nearest gene. Probability densities are plotted (*y*-axis) against log10-transformed distances (*x*-axis). Similarity of probability distributions between real data (blue curve) and simulated data (red) are calculated by Kolmogorov–Smirnov test and *P*-values are indicated for each TCAST element.

For TCAST elements located within intergenic regions, we analyzed the profile of their distances relative to the nearest gene and compared it to the profile of distances set down at random (Figure 5). Given that the TCAST elements were identified from the sequenced genomic portion and on assembled chromosomes, we excluded all gaps and unplaced scaffolds from simulations. The similarity of probability distributions between real data (shown by the blue curve) and simulated data (shown in red) was calculated by the K–S test, and *P*-values are indicated for each TCAST element. Within intergenic regions, the distribution of most TCAST families corresponds to a random dispersion pattern (*P* > 0.05). The exceptions are TCAST10 (*P* = 2.8 × 10^−8^) and TCAST7 (*P* < 0.05) repeats, which are positioned closer to genes than is expected by chance. Both elements show two distinct peaks, indicating separation of sequences into two populations with different proximity to genes. TCAST3 (*P* < 0.05) repeats are more distant to the nearest gene than expected if distances are set down at random (Figure 4).

The results reveal a general enrichment of TCAST repeats within introns, while within intergenic regions most repeats, except TCAST10, TCAST7, and TCAST3, exhibit a random distribution. It is interesting that TCAST1, which was previously shown to modulate the expression of neighboring genes (Feliciello *et al.* 2015b), also exhibits a random distribution pattern within intergenic regions.

### Transcriptional activity of TCAST families

Transcription of the major TCAST1 satellite DNA occurs under standard physiological conditions but is significantly induced by long-term heat stress, and this transcriptional increase is correlated with the suppression of genes positioned in the vicinity of dispersed TCAST1 repeats (Feliciello *et al.* 2015b). Similar to TCAST1, the minor TCAST2 satellite is predominantly located within heterochromatin (Feliciello *et al.* 2015a), while the TCAST3–TCAST10 families also seem to be overrepresented within an unassembled heterochromatic portion of the genome. Since *T. castaneum* heterochromatin is highly sensitive to stress conditions (Pezer and Ugarković 2012), an increase in expression of not only TCAST1 but of other TCAST repetitive families could be expected after heat stress.

**Figure 6:**
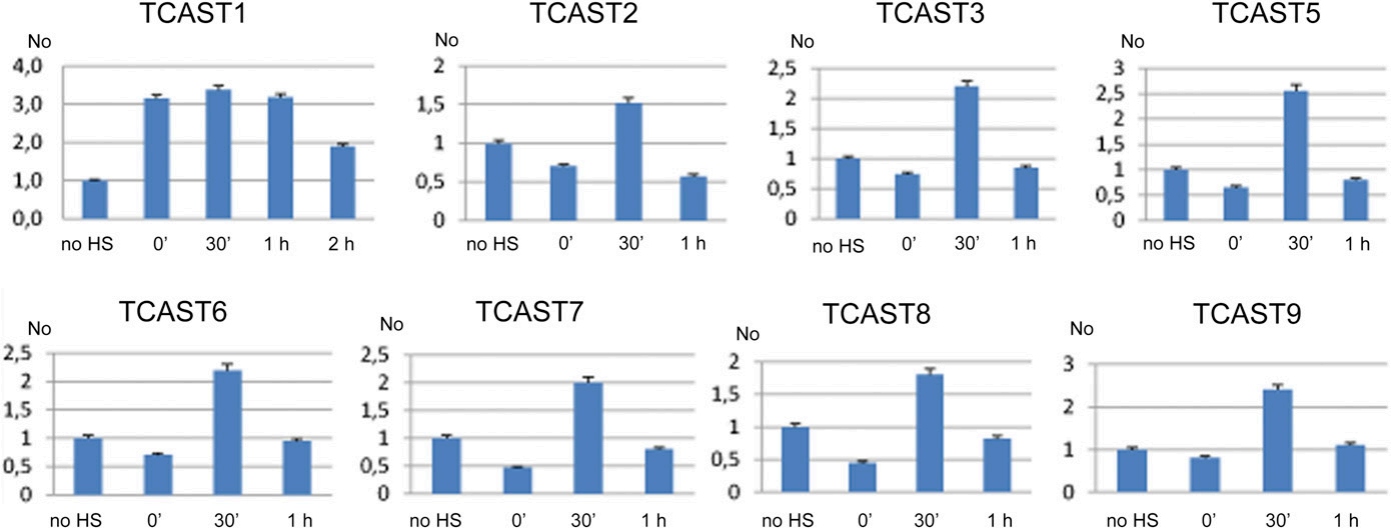
Dynamics of expression of repetitive TCAST families measured under standard conditions [no HS (heat shock)], immediately after long-term heat stress of 24 hr at 40° (0 min), at 30 min, or 1 or 2 hr of recovery at 25°. Relative No values are shown, which are obtained by dividing each No value by No value of control (no HS) for each repetitive DNA. Columns show averages of two different qRT-PCR experiments performed in triplicate and error bars represent SD.

To see if TCAST families are transcriptionally active and specifically induced by heat stress, we subjected adult beetles to long-term heat stress for 24 hr at 40°, and tested the expression of TCAST families immediately after heat stress and during a recovery period at 25° using qPCR. Expression of TCAST repetitive families was detected under standard physiological conditions (Figure 6) with the exception of TCAST4 and TCAST10, which showed very low and unreliable levels of expression, and were excluded from further studies. Considering the dynamics of expression after heat stress, TCAST1 is significantly induced immediately after heat shock and remains constantly increased, ∼3× (*P* < 0.05) relative to the control during a 1 hr recovery period, while after 2 hr of recovery it drops to the control level. For other families, a maximal increase relative to the control (no heat shock) is observed after a recovery period of 30 min, ranging between 1.5× for TCAST2 and 2.5× for TCAST5 (*P* values < 0.05), while after 1 hr of recovery, the expression of all families returns to the control level (Figure 6).

The results reveal that most TCAST families are transcriptionally active at standard physiological conditions and, in addition, are induced by the same heat stress conditions. The dynamics of transcription activation after long term heat stress are very similar for low-copy number TCAST families, occurring within a short time frame of ∼30 min of recovery period. The transcription activation of the major TCAST1 family is more permanent, starting immediately after heat shock and remaining constantly increased within the first hour of recovery after heat stress.

## Discussion

The genome of the beetle *T. castaneum* is rich in repetitive DNAs (Wang *et al.* 2008), satellite DNAs in particular, among which the major satellite DNA TCAST1 makes up 35% of the genomic DNA (Feliciello *et al.* 2011). Bioinformatic analysis of *T. castaneum* genomic data revealed additional repetitive DNA families (Wang *et al.* 2008; Pavlek *et al.* 2015), and some of them were characterized in this study with respect to their structure, origin, genome organization, and transcriptional activity. The principal aim of the performed research was to investigate if different TCAST repetitive families exhibit common features comparable to the major TCAST1 satellite family, which was previously shown to influence gene expression under specific stress conditions (Feliciello *et al.* 2015b).

Our study shows that all TCAST families, TCAST1–TCAST10, are dispersed within the euchromatin of 10 *T. castaneum* chromosomes, preferentially in the form of single repeats. Dispersed TCAST elements were retrieved from the well-assembled parts of the *T. castaneum* genome, mostly from introns and gene vicinities, and therefore their sequence features could be considered as a reliable. There is a similar level of sequence homogenization between nearby and distant repeats for all families, and a general absence of sequence clustering, indicating that dispersed repeats result from independent insertion events. After insertion, repeats were not significantly homogenized either at the local, chromosomal level, or among chromosomes. The only exceptions were tandem repeats (3-mer, 5-mer, 7-mer, and 10-mer) of TCAST5 and TCAST9, which cluster on the phylogenetic tree and, due to their homogeneity and tandem organization, are proposed to derive from heterochromatin. Within heterochromatin, long arrays of tandem repeats are efficiently homogenized by genetic mechanisms of concerted evolution (Dover 1986; Feliciello *et al.* 2005, 2006) and are protected from internal recombination by heterochromatin-specific histone modifications that are responsible for chromatin condensation (Volpe *et al.* 2002; Grewal and Elgin 2007). However, heterochromatin structure is highly sensitive to environmental stress, in particular to heat shock, which in *T. castaneum* induces demethylation (Feliciello *et al.* 2013) and the overexpression of TCAST1 satellite DNA (Pezer and Ugarković 2012). Results presented in this study show that, in addition to TCAST1, other low-copy number repetitive DNA families are also overexpressed after heat stress. Heat stress-induced remodeling of the heterochromatin structure is proposed to stimulate recombination within satellite DNA arrays, leading to the excision of repeats in the form of extrachromosomal circles, which were experimentally detected in plants and animals (Navrátilová *et al.* 2008; Cohen and Segal 2009). Extrachromosomal circles could be subjected to rolling circle replication, which can lead to rapid changes in the amount of satellite DNA observed at the level of populations in *T. castaneum* (Feliciello *et al.* 2015a), *D. melanogaster* (Wei *et al.* 2014), and *Triatoma infestans* (Pita *et al.* 2017), while in some species can be even detected at the individual level (Cardone *et al.* 1997). Rolling circle replication is proposed to contribute to the high sequence homogeneity of repeats within heterochromatin, since newly amplified homogenous repeats might replace homologous segments that have accumulated mutations, and in this way, could act as a satellite DNA repair mechanism (Feliciello *et al.* 2005, 2006). Site-directed recombination was proposed to occur between motifs within extrachromosomal circular DNAs and homologous motifs within the genome, leading to the dispersion of tandem repeats from heterochromatin to euchromatin (Brajković *et al.* 2012; Feliciello *et al.* 2015a). Within euchromatin, such tandem repeats appear to be unstable and prone to excision, resulting in a decrease in copy number or a complete loss of repeats (Feliciello *et al.* 2015b). Unlike TCAST1–10 repetitive families, which are located both within heterochromatin and as predominantly single dispersed repeats within euchromatin (Brajković *et al.* 2012; Feliciello *et al.* 2015a; this paper), there are repetitive families within the *T. castaneum* genome that reside as short tandem arrays exclusively in euchromatin (Pavlek *et al.* 2015). Such diversity in genomic organization and the distribution of repetitive families in *T. castaneum* genome might be related to their potential multiple roles in genome organization and evolution, as well as in gene expression modulation. It is interesting that higher-order repeats are detected in tandemly repeated families residing either in the heterochromatin or euchromatin of *T. castaneum* (Vlahović *et al.* 2017).

In addition to site-directed homologous recombination, which is proposed as a mechanism responsible for the excision of repeats from heterochromatin and their spreading within euchromatin, transposition is another mechanism of repeat proliferation that can be envisaged. TCAST elements of probable transposon origin, such as TCAST4, TCAST7, TCAST8, and TCAST10, as well as the TCAST1 element embedded in a more complex repeat that resembles a DNA transposon, also have short direct duplications at the insertion sites, mostly of 3 bp, which is characteristic for the mariner/Tc1 superfamily of DNA transposons (Capy *et al.* 1998; Feschotte and Pritham 2007; Brajković *et al.* 2012). In addition, TCAST1 and TCAST7 exhibit hallmarks of DNA transposons such as TIRs, while TCAST4, TCAST8, and TCAST10 are sometimes organized in dimers composed of inverted repeats. (Re)transposons are considered as a source of some satellite DNAs in insects and plants (Heikkinen *et al.* 1995; Macas *et al.* 2009; Sharma *et al.* 2013), while transposons of the *Helitron* superfamily are suggested to be involved in the generation and spreading of satellite DNAs throughout the genome of *Drosophila* (Dias *et al.* 2015) and molluscs (Šatović *et al.* 2016).

In the red flour beetle *T. castaneum*, TCAST1 satellite DNA, as the major heterochromatin constituent, plays a role in heterochromatin remodelling during development and the environmental stress response (Pezer and Ugarković 2012). Increased levels of TCAST1 transcripts are accompanied by an increase of the repressive epigenetic modifications of histones at satellite DNA repeats dispersed within euchromatin, and correlate with the suppression of nearby genes (Feliciello *et al.* 2015b). Dispersed TCAST1 repeats therefore act as nucleation cores for repressed chromatin. Since this novel mode of gene regulation does not seem to be unique to the specific satellite DNA sequence/gene, it could be hypothesized that, like TCAST1, some other TCAST repetitive families that are partially dispersed within the euchromatin and transcriptionally active could also influence the expression of associated genes by a mechanism of “heterochromatinization.” Our study reveals that diverse TCAST repetitive families share common characteristics, such as dispersion in the vicinity of genes and transcriptional activation at specific stress conditions, suggesting their possible gene-modulatory role. This makes case for the future studies of the *T. castaneum* transcriptome at standard and heat stress conditions, which may reveal whether or not there is a correlation in the expression of particular TCAST families and suppression of genes associated with their dispersed repeats.

Considering the distribution of TCAST elements and genes along chromosomes, they are not significantly correlated, but there are chromosomal regions with clustered and colocalized TCAST repeats, *e.g.*, analysis of TCAST1-associated genes reveals that 70% of them have other TCAST elements in close vicinity, with a maximum of 24 TCAST elements near a single gene (Table S6 in File S3). This indicates that their influence on genes within such regions might be either neutral or even beneficial, while TCAST repeats are probably impoverished in the regions where their influence on genes might be predominantly deleterious. In spite of the different origins of TCAST families, the similarity of their dispersion profiles suggests that they are shaped by the same process, most probably by selection, and not by mechanisms of spreading such as transposition and homologous recombination. This is in accordance with theoretical studies on transposons that showed that transposition rate has a weak influence on the frequency of their spreading within the genome and the population, respectively (Charlesworth and Charlesworth 1983; Langley *et al.* 1983). Instead of transposition, selection against the deleterious impacts of transposable elements is considered to play a role in the shaping of their dispersion profiles (Lee and Langley 2010; Blumenstiel *et al.* 2014). Recent data show that transposons can positively influence gene expression by increasing the complexity of the transcriptional response to the environment [reviewed in Bodega and Orlando (2014)].

In summary, our study reveals common features of diverse TCAST repetitive families with respect to their genome organization, chromosome distribution, and association with genes, as well as transcriptional activation. We consider this work a valuable resource for the future study of the potential gene-modulatory role of TCAST repetitive families.

## Supplementary Material

Supplemental material is available online at www.g3journal.org/lookup/suppl/doi:10.1534/g3.117.300267/-/DC1.
