# Supplementary material for: Dispersion Profiles and Gene Associations of Repetitive DNAs in the Euchromatin of the Beetle Tribolium castaneum
Source: G3 (Bethesda). 2018 Jan 8;8(3):875–86. doi: 10.1534/g3.117.300267 (PMC5844308; doi:10.1534/g3.117.300267)
Supplement: Supplementary file 1 [file 875FileS1.doc]

**Suppl. File 1**

**A Alignment of repeats belonging to dispersed TCAST3 elements**

>1LG2

agcgtgttaggaaatggtttgcaaaaatttagtagatagagaataagagaaatcgtataa

gcaaaagtaaaaaatgcatagttttccttctgaaattaaagtcccaaattttgagaacgc

tctagaaaaatcgtaaataataaatttacaggtaggtccactttcagttattaattttac

gcaaaattttttcaggacgaacctccatgaaaaaattctcaaacccattatgcaaataaa

aaaagccagtggctctcaacaggtgggttaataaaatttctgaagaaagtacctaataaa

tgtataaggatttttaactcgtctgatcacgacaaattcacatacgaaatattgcta

>349LG8

ggcgtgttacggaattgttttcaaaaatttagtagatagagaataagacaaatcgattaa

gcaaaagtaaaaaatgcataattttccttctgaaattaaagtcccaaattttgagaacga

actagaaaaatcttaaataatatattaacaggtaggtcca--------------------

--aaagtcttct--------------------------------------tgctaataaa

ataaaccagtggctctcaataggtgtgttaataaaacatttgaaa------------aaa

tgtataagggtttttaattcgtctgatcatgacaaattcacatacgaaatattgcta

>62LG3

agcgtgttagaaaatggtttgcaaaaatttagtagatagggagtaaaacaaatcgattaa

gcaaaagtaaaaaatgcatagttttccttctgaaattaaagtcccaaattttgagaaaga

actagaaaaatcgtaactaatattttgacaggtaggtctacttttaattattaattttac

a-aaaattttttcagg------tcct----aaaaactctcagaatcattttgctaataaa

ataagccagtggctctcaacatgtgggttaataaaatttccgaaaaatgtatgcgaaaaa

tatataagggtttttaactcgtctgatcatgactaattcacatacgaattgttgcta

>88LG9

agcctgttagggaatggtttgcaaaaatttagtaaatagcgaataagacaaatcgattaa

gcaaaagt-------------------ttctgaaattaaagttccaaattttgagaacga

actagaaaaatcataaataatatattgacaggtaggtccactttcaattattaatttatc

gcaaaattgccacag-----------atgaaaaaattctcaaaaccattatgcgaataaa

ataagccagtggctctcaacagatgagttaataaaatttctgaaaaaagtacgcgaaaaa

tgtaaaagggtttttaactcgtctgatcatgacaaattcacatacgaaatattacta

>480LG5

------------------------------------------------------------

---------------------------ttctgacattaaagtcccaaattttgagaacga

actagaaaaatcgtaaataatatattgacaggtaggtccactttc---------------

-------------------------------------------------------ataac

ataagctagcggctctcaacaggtgggttaataaaatttctgaggaaagtacgcgataaa

tgtgtaagggtttttaacaagtctgatcttgacaaatgcacatacgaaatattgcta

>2LG6

agtgtgctagggaatggtttgcaaaaatttagcaaatagagaataagacaaatcgattaa

gcaaaagtaaaaaatacatagttttccttctgaaattaaagtcccaacttttgagaacga

cctagaaaaatcgtaattaatatattgacaggtaggtccactttcagttattaattttac

gaaatcctttttcaaaacaaacctccatgaaaaaattttcaatactattttgctaataaa

atgagccagtggttctcaaccggtgggttaacaaaatttctgaagaaagtacgcgaaaaa

tgtataagggtttttaactcgtctgattatgacaaatttacatacgaattactgcta

>14LG6

agcgtgttagggaatggtttgcaaaaacttagtaaatagagaataagataaatcgattaa

gcaaaattaaaaaatgcatagttttccttctgaaattaaagtcccaaattttgagaacga

actagaaaaatcgtaaataatatattgaca----ggtccactttcaattgttaattatac

gaaatattttttcaggac--------atgaaaaaattctcaaaactattttactagtaaa

ataagccagtggctctcaacaggtgggttaataaaatttctgaagaaagtacgcgaaaaa

tgtatga-ggtttttaactcgtctgaccatgacaaattcacatacgaaatattgcta

>3LG3

agcttgttagggaatggtttgcaaaaatttagtaaatagaaaataagaccaatcgattaa

gcaaaaggcaaaaatacatagtttttcttctgaagttaaagtcccaaattttgagaacga

actaaaaaaatcgtaaataatatagtgacaggtaggtcctctttcaactattaattttac

gaaaaattttttaaggacgaatcttcataagaaaattctcaaaaccattttgccgataaa

ataatccagtggctctcaacaggtgggttaataaaatttctgaagaaagtacgcgaaaag

tgtataagggtttttaactcgtctgatcatgactaattcacatacgaaatattgcta

>228LG2

------------------------------------------------------------

------------------------------------------------------------

-----------------taataaattgacaggtaggtccactttcagttattaattttaa

gaaaaattttctcaggacgaatct--ataagaagattcttaaaaccattttacaaataaa

ataagccagtggctctcaacatgtgggttaataaaatttctgaagaaagtacgcgataaa

tgtgtaaggatttttaactcgtctgattatgacaaatgcacatgcgaaatattgcta

>4LG9

agcgtgttagggaatggtttgcaaaaatgtagtagatagagaataagacaaatcgattaa

gcaaaagtaaaaaattcatagttttcctcctaaaattaaagtcctaaattttgagaacga

tttagaaaaatcataaataaaaaattgacaggtaggtccactttcaattattaattttaa

gaaaaattttttcagaacgaatcttgaaaaaaaaattctcaaaaccattttactaataaa

ataaaccagtagctctcaacaggtgggttcgcaaaatttctgaagaaagtacgcgataaa

tgtataagggtttttaactcgtctggtcatgacaaattcacatgcgaaatattggta

>25LG9

agcgttttagggaattgtttgcaacaatttagaagatagagaataagacaaatcgattaa

gcaaaagtaaaaattacatagttttccttctgaaattaaagtaccaaattttgagaacga

actgaaaaaatcgtaaataatatattgacaggtagatctactttcaattattaattttac

gaaaaattttttcaggacgaacttcaatgaaaaaactttcaaaaccattttgttaattaa

atcagtcagtggctctcaacaggtgggttaataaaatttctgaagaaagtacacaggaaa

tatataagggtttttaactt-------------------------------------

>83LG9

agcgttttagggaattgtttgcaaaaatttagtagatagagaataagacaaatcgattaa

gcaaaagtaaaaagtacatagttttcc----------------ccaaattttgagaacga

attagaaaaatcgtaaataatatattgacaggtagatctactttcagttattaattttac

gaaaaattttttcaggacgaacttcaatgaaaaaactttcaaaaccattttgctaataaa

ataagccagtggttctcaatagttgggttaataaaatttctgaaaaaagtaaccggaaaa

tgtataagggttttta-----------------------------------------

>79LG6

----------------------------------------------gagaaatcgattaa

gcaaaagtaaaaaatgcatagttttccttctgaaattaaagtcccaaattttgagaacga

actagaaaaatcgtaaataatatattgagagttaggtccactttaaattattaattttag

gaatt-ttttttcagggcgaacttccatgagaaatttctcaaaaccattttgctaataaa

ataagccagtggttctcaacaggtgggttaataaaatttctgaagaaagtacccggaaaa

tgtataagggttcttag----------------------------------------

>36LG8

agcgtcttagagaatggttttcaaaaaattagtagatagagaataagactaatcgattaa

gcaaaagtaaaaaatgtatagttttccttctgaatttaaagtcccaaattttgagaacga

aatagaaaaatcgtaaattatatattgaaaggttggtccactttcaattaaaaattttaa

gaaaa-atttttcaggacgaatctccatgaaaaaattctcaagagcattttgctaataaa

ataagccagtggttctcaacaggtgggttaataaaacttctgaagaaagtacccgaaaaa

tgtataagggtt------------gatcatgacaaattcacttac-aaatattgcta

>126LG10

------------------------------------------------------------

--aaaagtaataaatgcatagttttcctcttgaaattaaagttccaaattttgagaacga

actagaaaaatcaaaaataatatattgtcaggtaggactactttcaattattaattgacg

aaaaacttttttcgggacgaacctccatgaaaaaactctcaaaactattttgctaataaa

ataagccagtggctctcaacaggtgggttaataaaatttctgaagaaagtacgcga----

---------------------------------------------------------

>275LG5

-------------------------------------------------------tttaa

gcaaaagtaaaaaatgcatagttttcc----------aaagtcccaaattttgagaacga

actagaaaaatcgtaaataatatatttacaggtgagttcacttttaattattaattttaa

gaaaa-atttttcagtacgaacctccacgaaaaaactctcaaaacaattttgctaataaa

ataagtcagtggctctcaacaggtgggtttaaaaaatctctgaa----------------

---------------------------------------------------------

>184LG8

---------------------------------------------agacaaatcaattag

gcaaaagtaaaaagtgcatagttttccttc-------------ccaaattttgagaacga

actagaaaaatcgtaaataatatattgacaggtaggcccactttcaattattaattttac

gaaaaa---cttcaggacgaacctccacgaaaatattttcaaaaccacttcgctaataaa

accagccagtggttctcaacaggtgtgttaataaaatttctgaagaaagtaggcaaaaaa

tgtataagggtttttaattcgtcta--------------------------------

>9LG2

---------ggaaatggtttgcaaaaatttagtaaatagagaataaaacaaatcgattaa

gcaaaagtaaaaaatgcatagttttccttctgaaattaaagtcccaaattttgagaacga

actaggaaaatcgtaaataatatattgacaggtaggttcactttcaattattaattttac

aaaaaactgtttcaccacaaacatccacgaaaaaattttcaaaacct--ttgctaataaa

ataagccagtggctctcgacaggtgggttaataaaatttataaagaaagtatgcgaaata

tttataagggtttttaactcgtctaa----aacaaattcacataggaaatattgcta

>38LG9

------------------------aaatttagtaaatagagaataagacagatcgattaa

gtaaaagtaaaaaatgcatagtttttcttttaaaattaaacttccaaattttgagaatga

atcagaaaaatcgtaaataatatattgacaggtaggtccacttttaattattaattttac

caaat-------------------------------------------------------

------------------------------------------------------------

---------------------------------------------------------

>134LG2

aacgtgttagtgaatggtttgcaaaaatttagtatatagaggataagacaaatcaatcaa

gcaaaactaaaaaatgcatagtttttcttttgaaattaaagttctaaattttgagaagga

actagaaaaatggtaaataatatactgataggtaggtccaccttcaattattaattttat

gaaaaatgtttttcggaggtacctctatgaaaaaattcccaaaaccattttgctaataaa

ataagccagtggctctcaacaagtgggttaataaaat-----------------------

---------------------------------------------------------

>328LG3

----atgttgggaatggtttgcaaaaattgagtaggtagagaataagacaaatcgattaa

gcaaaagtaaaaagtgcatagttttccttctgaaattgaagtctcaaattttgagaacga

actagaaaaatcgtaaataatataatgacaggtacgtctacttttaattattaattttac

aaaaa-------------------------------------------------------

------------------------------------------------------------

---------------------------------------------------------

>10LG3

----tgttacggaatggtttgcaaaaatttagttgatagagaataagacaaatcgattaa

gtaaaagtaaacaatgcatagttttccttctgaaattaaagttacaaattttgagaacga

actagaaaaatcgtagataatatattgacaagtaggtccaccttcaattattaattttac

gaaa--ttttttcagaacaaacttccatgaaaaaattgtcaaaaccatgttgctactaaa

ataaaccagtggctttcaatacgtgggattgta-aatttctgaagaaagtacgcgaaaaa

tgtataagggtttttaactcgtctaatcacgccaaattcacatacgaaatattgcta

>101LG10

-----------------------aaaattta-tagatagagaataagacaaatcgattaa

gtaaaagtaaaaaatgcatagttttccttttgaaattaaagtcccaaattttgagaacga

actagaaaaatcgtaaataatatatcgacaggtaggtccacattcaattattaattttc-

------------------------------------------------------------

------------------------------------------------------------

---------------------------------------------------------

>102LG10

-----------------------aaaattta-tagatagagaataagacaaatcgattaa

gtaaaagtaaaaaatgcatagttttccttttgaaattaaagtcccaaattttgagaacga

actagaaaaatcgtaaataatatatcgacaggtaggtccacattcaattattaattttc-

------------------------------------------------------------

------------------------------------------------------------

---------------------------------------------------------

>103LG10

------------------------aagtttagtagatagagaataaaagaaaccgattaa

gta---gtaaaaaatgcatagttttccttctgaaattaaagtcccaaattttgagaacga

actagaaaaatcgtaaaaaatatatcgacaggtaggtccacattcaattattaattttc-

------------------------------------------------------------

------------------------------------------------------------

---------------------------------------------------------

>35LG9

agcgtgttagagaatgatttgcaaaaatttagcaaataaagaataaaacaaatcggttaa

gcaaaagtaaataatgcatagttttccttctgaaattaaagtcccaaattttgagaacga

actagaaaaatcataaataatatattgacagcaaggcccactttcaattattaattttac

gaaaaattttttcaggacaaacctccacgaaaaaattttaaatttca--ttgataataaa

gtaagccagtggctctcaatcggtgggttaacaaaatttctgaagaaagtacgcgaaaag

tt-ttttgggtttttaactcgtctgattacgaaaaattcacaaacgaattagtgcta

>95LG4

agcgtgttagggaatggtttgcaaaaatttagcaaacagaaaatatgacaaatcgattaa

gcaaaaataaaaaatgcatagttttccttctgaaattgaagtcctaaattttgagaacga

actagaaaaaacgtaaataatatatttacaggtaggtccactttcaattattaatcttac

gaaaataactttcagaacaaacctccatgaaaaaattctaaaaaccgt-ttgctaataaa

ataagccagtggctctcaaccggtgg--taacaaaatttctgaagaaagtatgc------

---------------------------------------------------------

>259LG3

agcgtgttagggaatggtttgcaaaaatttagtagatagagaataagacaaaccgattaa

gcaaaagtaaaaaatgcatagttttccttctgaaattaaagtcccaaattttgagaacga

actagaaaaatcgtaaatattatattgccaggaaggtccactttcaataattaattttac

aaaa--------------------------------------------------------

------------------------------------------------------------

---------------------------------------------------------

>67LG6

atcgtcttagggaattgtttgcaaaattttagtagatagagaataagacaaatcgattaa

gcaaaagtaaaaaatgtatagttttccttctgaaattaatgtcccacattttgagaacga

actagaaaaatcgtaaacaatatattgacaggcaggtccactttcacttattaattttac

gaaaaattttttcaggacgaatcgcc-----aaaattttcaaaaccatattgctaataat

ctaagccagtggctctcaacaggtgggttaataaaatttctgaagaaagtacacgagaaa

tg-ataagggtttttaactcgtctgaacttgacaaattcacataggaaatattgcta

>182LG7

agcatattagggaatagtttgcaaaaatttagcagataaagaataagacaaatcgattaa

ataaaagtaaaaaatgcatggtttttcttctaaaattaaagtcccaaattttgagaacga

agtagaaaaatcgtaaataatatattgacaggtaggtccattttcaataagtaattttac

g-----ttttttttgaacgaacctccgtgaaaaaattctcaaaaccaatttgttaataaa

atcaaccagtggctcttaataggtgggttaatataatttgtaaagaaaa-----------

---------------------------------------------------------

>408LG6

agcgtgttagggaatggtttgcaaaaatttagtagatagggaataagacaaatcgattaa

a------caaaaaatgcatagttttccttctgaaattaatgtctcagattttgagaacga

actagaaaaagcgtaaataatatattgacaggcaggtccactttcaaa------------

------------------------------------------------------------

------------------------------------------------------------

---------------------------------------------------------

>324LG2

-tcgtattagggaatggtttgc-aaaatttagtaaatagagaataagacaaatcgattaa

gcaaaagtaaaaaatgcatagttttccttctggaattaaagtcccaaattttgagaatga

actagaaaaatcgtaaataatatattgacaggtaagtccactttcaattggtaattt---

------------------------------------------------------------

------------------------------------------------------------

---------------------------------------------------------

>49LG8

agcgtgttagggagtggtttgcaaaaatttagtagatagagaataagacaaaccaattaa

gtaaaagtaaaaaatgcatagttttctttctgaaattaaagtcccatattttgaaaacaa

actaggaaaatcgtaaataatacattgacaggtaggttctctttcaattattacttttac

aa----ttttttcgggac-----tctatgaaaaaattctcaaaaccattt--tttataaa

atgagccagtggctcccaacaggtgggttaataaaatttctgaaaaaagtaggca-----

--tataagagtttttaaatcgtctgatcatgacaaattcacgtacgaaatattgcta

>13LG9

agcatgttagggaatggtttgcaaaaatttagtagatagagaataagacaaatcgattga

gcaaaagtaaaaaatgtatagttttccttctgaaattaaagtcccacgttttgagaacaa

actagaacaatcataaataatatattcacaagtaagtccacttttaattattaattttac

gaaaaattttttcaggacgaacctccatgataaatttctcaaaaccattttgctaataaa

ataagcaagtggctctcaataggtgggttaataaaattcttgaagaaagtacgcgaaaaa

tgcttaaaagtttttaattcgtctgatcatgacaaattcacatacgaaatattgtta

>371LG2

agcatgttagggaatggtttggaaaaaattagtagacagaaaataggacaaatcgattaa

gcaaaagtaaaaaatgtacagttttttttctaaaattaaagtcccaaattttgagaacga

actagaaaaatcgtaaataatatattaacag---gatccactttcaattat---------

------------------------------------------------------------

------------------------------------------------------------

---------------------------------------------------------

>294LGX

accgtgttagggaatgatttgcaaaaatttagtaggtagagaataagacaaattgattaa

acaaaagtaaaaaatgtatagtttttcttttgaaattaaagtcccaaattttgacaagga

aatagaaaaatcgtaaataatatattgacaggtaggtccactttcaattattaattgtac

aaaaa-ttttttca--------------gaaaaaatcctcaaaaccattttactaataaa

gtatgcgagtggctctcaacaggtgggttaataaaatttctgaaggaagtaagcgaaaaa

tgtacaagggtttttaactcgtctgatcatgacaaattcacatacgaattattgcta

>112aLG9

agcgtgttaagaaatggtttagaaaaatttagtagatagaaaataaaacaagtcgattaa

gcaaaagtaaaaaatgcacagttttccttctgaaattaa-gtcccaaattttgaaaacga

actagaaaaatcgtaaataatatattgacaggtaggtcccctttcaattattaattttac

gaaaaattttttcaagatgaacctccatgaaataattctcaaaactattttgctaataaa

attagccagtggctctcaacaggtgggttaataaattgtttgaagaaagtacgcgaaaaa

tg-atatgagtttttaatcc---------tgaaaaattcacatacgaaataatgcta

>5LG7

agtgtgtttgggaatggtttgcaaaaatttaataaatagagaataagacaaatcgattaa

ggaaaagtaaaaaatgcatagttttccttttgaaattaaagtcccaaattttgagaacga

actagaaaaat--taaattatatattgacaggtaggtctactttcaattattaatattac

gaaaaattttgtcaggacgaacttccatgaaaaatttcccaaaaccattt--ctaataaa

ataagccagtggttctcaacagttgggttaataaaatttctgaagaaagtacgcggaaaa

tatataagggtttttaactcgtctgatcgtgacaaatttacatacgaaatattgcta

>11LG3

agcgtgttagagtatggtttgcaaaaatttagtagatagagaataaaacaaatcgattaa

gcaaaggtaaaaaatgcatagttttccttttgaaattaaagtcacaaattttgagaacga

actaaaaaaatcgtaaataatatattgac---taggtccactttcaattattaattttac

gaaaaattttttcagaacaaacttccatgaaaaatttctcaaatccattt----aattaa

ataagccagtggttctcaacaggtgggttgataaaaattctgaaaaaagtacgcggaaaa

tatataaaggtttttaactcgtctgatcatgacaaatttacatacaaaatattgctc

>21LG10

agcgttttagggaatggtttgcaaaaaattagtaaatagagaataagacaaatagattat

gcaaaagtaaaaaatgcatagttctttttctgaaattaaagtcccaaattttgagaacga

actagaaaaatagtaattaatatattgacacgtaagtccactttcagttattaattttac

gaaaaaatatttcaggacgaatctccattaaaaaattttcaaaacaattttgctaataaa

ataacccagtggttctcaacagttgggttaataaaatttttgaaaaaagtacgcggaaaa

tgtgtaagggtttttaactcgtctgatcatgacaaattcacatacgaaatattgcta

>141LG3

agcgtgctggggaatagtatgcaaaaatttagtagatagaaaataaaacaaatcaattaa

tcaaaagtaaaaaatccatagatttccttctgaaattaaagtccccaattttgagaacga

actagaaaaatcgtaaataatatattgacaggtaggtccactttcaattattaattttac

gaaaaa--ttatcaggacgaatctccaagaaaaagttctcaaaactattttgttaattaa

ataagccagtggctctcaacaggtga----------------------------gtgata

tgtataagggtttttaactcgtcagatcatgacaaattcacatgcgaaatattgcta

>352LG3

attgtgttaggaaatgttt-gcagaaatttggtagatagagactaaaaccaatcgattaa

acaaaagtaaaaaatgcacaattttccttctgaaattaaagtcccaaattttaaaaacga

attagaaaaatcgtaaataatatattgacaggtagatccactttcaaatattaatttttc

gaaaaa------------------------------------------------------

------------------------------------------------------------

---------------------------------------------------------

>28LG3

aacgtgttaagtaacggtttgcaaaaatttagtagatagaaaataagacacatcgattaa

gcaaaattata----------ttttccttctgaaattaaaatcccaaattttgagaacga

actagaaaaatcgtaaataatatattgacaagtaggaccactttcaattattaattttac

gaaaaattttttctgtatgaacctctatgaaaaaattgttaaaaccattttgctaataaa

ataagccagtggctctcaacaggtcggttaataaaatttcttaagaaagtacacgaaaaa

tatataagggtttttaactcgtctgcttataacaaattcacatacgtaatactgcta

>466LG9

agcgtgttagggaatggtttgtaaaaatatagtaaatagagaataagacaaatcgattaa

gca-----------------------------aaattgaagtcccaaattttgagaacaa

actagaaatatcgtaaataatatattgacaggtaggtccactttcagttattaatttaac

gaaaaattttat------------------------------------------------

------------------------------------------------------------

---------------------------------------------------------

>356LG8

----------------gtttgcaaatatttaacagatagagaataagacaaatggattaa

gcaaaagtaaaaaattcatacttttccttcttaaattaaagtcccaaattttgagaacaa

actagaaaaatcgtaaataatatattgacaggcaggtccactttcaattattaattttg-

---------------------------------------caaaaccattttgctaataaa

ataggacagtggctctcaacaggtgggttaataaaatttctgat----------------

---------------------------------------------------------

>50LG3

accgtgttagggaatagtttgcaaaaatttagcagataaagaataagacaaatcgattaa

gcaaaagtaaaaaaagcataattttctttctaaaattaaaggcccaaattttgagaacga

actagaaaaatcggaaataatattttgccaggtaggtccacttttgattattaagtttac

aaaaaactttttcagaacgaacttccaagaaaaaattttcaaaaccattttgctaataaa

ataagccagtggctctcaacaggtgggttaataaaatttctgaaaaaagtacgcgaaaaa

tgtata---------------------------------------------------

>17LG9

agcgtgttaaagaatggttggcaaaaatttagcaaatagaaaataagacaaattgattta

gctaaagtaaaaaatgcatagtttttcttctgaaattatagtctcaaattttgagaacga

actaggaaaatcgtaaataatatattgactggtaggtccattttgaatttttaattttac

gaaaaatttttccaggacgaatcttcacgaaaaaattctcaaaaccactttgctaataag

ataagtcagtggctctcaatagttcggttaataaaatttctgaagaaagtaggtaaaaaa

tttataagggttttttaatcgtctgatcatgacaaattcacatatgaaatattgcta

>12LG2

agcgtgttagggaatggtttgcaaaaatttagcccatagagaataagacaaatcgattaa

acgaaagtaaaaaatgcatagttttccttctgaaattaaagccccaaatgttgagaacga

actagaaaaatcgtaaataatatgatgacaggtaggtctactttcaattaataatttaac

gaaaaattttttcaggataaacctctatgaaaaaattctcaaaaccattatgctaataaa

ataagccagtggttctcaaccggtgggttatcaaaatttctgaaaaag--acgcgaaaaa

tgtataagaatttttaactcgtctgattatgac-aattcacatatgaaatattccta

>61LG6

agcgtgttagggaatggcttgcaaaaatttagtagataaaaaataaaacaaatcgattaa

gcaaaaataaaaaatgcatagttttcattgtgaaattaaagtttcaaattttgagaatga

actagaaaaatagtaactaatatattgacagataggtccactttctattattaattttac

aaaaaatttttttcggacgaacctctataaaaaaattctcaaaatcattttgctgataaa

------cagtggctctcaacaggtgggttaataaactttctgaagaaagtacgcgaaaaa

tgtataagggtttttaa----------ctcgacaaattgacatacaaaatattgcta

>267LG3

agcgtgttagttaatggtttgcaaaaatttactagataggaaataggacaaatcgattaa

gtaaaagtaaaaaatgcttagttttccttctgaaattaaaatctcaaattttgaggacga

gctagaaaaatcgtaaatagtatattgacaggtaggtccgctttcaattattaattttac

gaaaaaatttttcaggacgagcctccatgaa-----------------------------

------------------------------------------------------------

---------------------------------------------------------

>342aLG3

agcgtgttagggaatgctttgcaaaaagttattagattgagaataaaacaaatcgattaa

gcaaaagtgaaaaatgcatcgtttttcttctgaaactaaagtctcaaattttgagaacga

aatagaaaaatcataaataatatgttgacaggtaggtccactttcagttattaattctac

gaaaaa------------------------------------------------------

------------------------------------------------------------

---------------------------------------------------------

>233LG10

agcgtgttagggaatggttggcaaaaatttaatagatagagaataaaacaaactgattaa

gtaaaaattaaaaatgcatagttttttatttgaaattaaggactcaaattttgagaacga

actagaaaaatcgtaaataatatattgacaggtaagtctacttttaattattaattttac

gaaaaattttttctggacgaacctccatgaaaaacttcttaaaac-----tccataagcc

------cagtggctctcaacagatgagttaataaaatgtctgaagaaagtacgcgataaa

ta-------------------------------------------------------

>64LG2

agtgtgttagggaaaggcttgcacaaattttttagatagagaataagacgaatcgattaa

gcaaaagtaaaaagtacatcgtttttcttctgaaattaaagtcccaaattttgagaacga

agtagaaaaatcataaataatatattgacaggtacgcccactttcacttattaattttac

aaaaaggtttttt-gaacgaacctccatgaaaaaattctcaaaaccgttttgataataaa

accaaccagtggctgtcaacaggtgggttaataaaatttttgaaaaaagtacgcgaagaa

tgtataaaggtttttaactcgtctgatcatgacaaattcacatacaaaatattgcta

>7LG10

-------taaggaatg-tttgcaaaaaattagcacctagagaataagacaaatcgattac

gcaaaagtaaaaaatgcatatttttccttctgaaattgaagccctaaattttgagaacga

atgagaaaaatcgcaaataatatgttgacaggtaggtccactttcaa-tataaaatttac

gaaatattttttcaggacaaacctccatgaaaaaattctcaaaaccattttgctaataaa

atatgccagtggctctcaaccggtgggttaacaaaatttctgaagaaaatacgcgaaaaa

tgtataaggttttttaactcgtctgattatgtcaaattcacatatgaaatattgcta

>15LG6

-tcgtgttagggaatggtttgcaaaaatttaataaatagagaataagacaaatcgattaa

gccaaagtaaaaaatgcatggttttccttctgaaattgaagttccaaattttgagaacga

actagaaaaatcctaagcaatatattgacacgtaagtccactttcaattat-gattttac

gaaaaattttttcaggacgactctttatgaaaaaattcttaaaattattttgctaataaa

ataaaccagtggctctcaacaggtgggataataatttttctgaagaaagtacgcggaaaa

tgtgtaagagtttttaacttgtcggatcatgacaaattcaaatacgaaatactgcaa

>375LG8

-----------------------aaaatttagtaaatatagaatgagacaaatccattaa

gcaaaagtaaaaaatgcatagttttccttctgaaattaaagtcccaacttttgagaacga

actagaaaaatcgtaaataatgtattgacaggtaggtccacctacaattattaattttac

aaaaaa--------------------atgaaaaaattcgctaaatcattttactaataaa

ataagccggtggctatcaacatatgggctaataaaatttctgacgaaaatacgcgaaaaa

tgtataagtaattttaactcgtctgatcattacaaattcacatgcgaaatattgcaa

>48LG9

------------------ttgcaaaaatttagtagatagagaataagacaaatctattaa

ataaaaataaaaaatgcataggttatctactgaaattaaaggcccaaattttgagaacga

attaaaaaaatcgtaaataatatattgtcaggtaggtccattttcaattattaattttac

gaaa--atttttcaggacgaacctccatgaaaaaattcttacaaacattttgctaataaa

ataagacagtggttctcaacatgtgggttaataaaatttctgaaaaaagtacgcgaaaaa

tgtataagggttttttaa-cgtctgatcaaggcaaattcacatacgaaatattgcta

>415LG9

------------------------------------------------------------

------gtaaaaaatgcatagatttccttctgaaattaaagtcttaaattttgagaacga

actagaaaaatcgtaaataatatattgac-----ggtcccatttcaattattaattttac

aaaaaatttttccaggacgaacctccatgaaaaaattctcaaaaa-----tgctaataaa

ataagacagtggctctcaacaggtttgttaataatatttctggaaaaagtacgcgaaaaa

tgtatat--------------------------------------------------

>8LG9

agcgtgttagggaatggtttgcaaaaatttagcaaatagagaataagacaaatcgattaa

gcaaaagtaaaaaatgtatagttttctttttgaaattaaagtcccaaattttgagtacga

actga-aaaatcgtaaataatattttgacaggtaggtccactttcaattattagttttac

gaaaaagtttttcacgacgaaccttcatgaaaaaaattttagaatcattttgctaataaa

ataagccagtggctctcaacaggtgggttaataaaatttctgaagaaagtatgcgaaaaa

attataggggttttcaacttgtctgatcataacaaattcacatacgaaatattgcta

>440LG9

cgcgtgatagggaatggtttgcaaaaatctaa-agacagaaaatatgacaaatgaattaa

gcaaaagtcaaaaatgcatacttttctttctaagattaaagtcccaaattttgagaacga

acta----------aaataatatattgaaaggtaggtccactttcaattattaattttaa

gaaaaa------------------------------------------------------

------------------------------------------------------------

---------------------------------------------------------

>454LG9

cgcgtgttagggaatggtttgcaaaaatttaa-agacagaaaatctgacaaattgattaa

gcaaaagttaaaaatgcatagttttccttctaagattaaagttccaaattttgagaacga

acta----------gaataatatattgaacggtaggt-cactttcaattattaattttaa

gaaaaa------------------------------------------------------

------------------------------------------------------------

---------------------------------------------------------

>93LG3

agcgtgttagggaatggtttgcaaaaatttagtagatagagaataaaacaaatcgattga

gcaaaagtaaaaaatgcatattttttcttctgaaattaaagtccaaaattttgagaacga

actagaaaaatcgtaaatgatatatggacaggtagttccactattaagtattaattttat

gaaaaattttttcaggacgaacttccatgaaaaaattttcaataccatttttaaagaaaa

-tgagccagtggctctcaacaggtgggttaataaagtttctgaagaaagtatgctaaaaa

tgtgtaagggtttttaacttatctaatcaagacaaattcacatacgaaatattgcta

>133LG4

agcgtgttagggaatggtttgcaaaaattcagtagatagagaataagacaaatcgattaa

gcaaaagtaaaaaatgcataacttttcttctgaaatt--agtcccaaattttgagaatga

acttgaaaaatcgtaaataatatattgacaggtgggttcactttcaattattaattttac

gaacaattttttcaggacgaacctttatgaaaaaattctcaacaccattttg--------

------------------------------------------------------------

-------------ctaaataaactaa-------------------------------

>261LG3

agcgtattagggaatggtttgcaaaaatttagtagatagagaataagacaaatcgtttaa

gcaaaagtaaaaaatgcatagttttcctcttaaagttaaagtcccaaattttgagaacaa

actcgaaaaatcgtaaataatatattgacaggtaggtccacttttaattataaattttac

aaaata--ttttcaggaagaacctccataaa-----------------------------

------------------------------------------------------------

---------------------------------------------------------

>327LG9

agcgtgttaggaagtagtttgcaaaaatttagtagatagagaaaaagacaaatcgattaa

gcaaaagtaaaaaatgcatagttttccttctgaaattaaagttacaaatttagagaacga

actagaaaaatcgtaaataatatattcacaggtaggtctactttcagtaattcattttac

aaaaaa------------------------------------------------------

------------------------------------------------------------

---------------------------------------------------------

>301LG3

tgcgtgttaggaaatggtttgcaataatttagtaaatagagaataagacaaatcgattca

gcaaaagtaaaaaatgcatagttttctttctgaaattaaagtcctaaactttgagaacga

actggaaaaatcataaataatatattgagaggtaggtccactttcaacgattaattttac

aaaaaatttt------------------gaaaaaattttcaaaactattgtactaataaa

ataaatcagtggctctcaacaggtgggttaataaaatttttggaaaaagtacgcgaaaaa

tgtctaagggtatttaactcgtctgatcatgacaaattcacataagaaatattgcta

>314LG2

agcgtgttagagaatggtttgcaaaaatttaacaa--tgagaataagacaaatcgattaa

gcaaaagtaaaaaatgcatagttttccttctgaaactcaagtcccaaattttgagaacga

actgtaagaatcgtaaataatatattgacaagtaggtccactttcaattattaattttac

gaaaaa--------------------------------------------tgctaat-aa

ataaacctgtggctctcaacaggtgggttaataaaatttctaaataaagtaa--------

---------------------------------------------------------

>59LG10

agcgtgttagggaatggtttgcacaaatttagtagataggaaataagaca-----attaa

gcaaaagtaaaaaatgcatagtttttcttctgaaattaaagtcccaaattttgaggacga

actaaaaaaaacgtaaataatatattgacaggttggtccactttcagttatcaattttac

gaaaaa-ttttgcaggacgaatctccatgaaaaaattctcaaaaccattttgctaataaa

atgaatcagtggctctcaacatgtgggttaataaaatttctgaagaaagtacacgaaaaa

tgtataaggatttttaactcg------------------------------------

>89LG3

agcgtgttatggaatagtttgcaaaaatttagcaaatagataataaaacaaatcgattaa

gctaaagtaaaaaatgcatagttttctttctgaaattgaagtcccaaattttgagaacga

acaagaaaaatcttaaataatatattgacagctaggtccactttcaaatattaattttac

gaaaa-------------aatcactcaaaaaaaaattctc-aaaccattttgctaataaa

ataagccagtggctctcaaccggtgggtaaacaaaatttctgcaaaaagtacgcgaaaaa

tgtttaagggtttttaactcttctgattatgacaaattcacatacgaattattgcta

>225aLG4

agcgtattacggaatggtttgcaaaaaaatta-gaatagacaataagacaaatcgattaa

gcaaaagtaaaaaatgcatagttttccttctgaaattaaagtcccaaattttgagaacga

actagaaaaatcataaattatatattgacagaaaggtttattttcagttatt--------

------------------------------------------------------------

------------------------------------------------------------

---------------------------------------------------------

>235LG3

agcgtgttaaggaatggtttgcaaaaattttgtagatagaaaaaaagacacatcgattaa

gcaaaagtaaaaaatgcataattttccttctgaaatt-aagtcccaaattttgaaaacga

actaggaaaatcgtaaataatatattgacagataggtccatttccaattattaattttac

gcaaa-------------aaatttttaggagaaaattctcaaaaccattttgctaataaa

ataagtcagtggctt---------------------------------------------

---------------------------------------------------------

>174aLG3

agcgtgttagtgtatggtttgcaaaaatttagtaaacagagaatatga-aaatcgattaa

acaaaagtaaaaaatgcatagttttcctttcgaaattaaagtcccaaattttgagaacga

actagaaaaatcgtaaataatatattgacaggtaggttcactttccattattaatttttg

gaaaaattgtttctgggcaaacttccatgaaaaatttctcaaaaccattttggtaata--

------------------------------------------------------------

---------------------------------------------------------

>426LG2

agcgtattagaaaatggtttgcaaaaatttagtagatagagaataaa-----------aa

acaaaagtaaaaaatgcttagttttccttttgaaattaaaatcccaaatttttagaacga

aagagaaaaatcataaataatatattgacaagtaggtccactttcaattattgattttt-

------------------------------------------------------------

------------------------------------------------------------

---------------------------------------------------------

>174gbLG3

agcgtgttaatgaatggtt-gcaaaaatttagtagatagaaaatatga-aaatcgattaa

acaaaagtaaaaaatgcatagttttcctttttaaattaaagtctcaaattttgagaacga

actagaaaaatcgtaaataatatattgataggtaggttcactttcaattattaattttag

gaaatattgtttctggac--------atgaaaaatttttcaaaa-cattttgctaataaa

aaaagccagtggttctcaaca---------------------------------------

---------------------------------------------------------

>65LG8

agcgtgttagcgaatggtttggagaaacttagtagatagagaataagacaaatcgattaa

gctaaagtaaaaaatgcatagtttttcttctaaaattaaaatctcaaattttgagcacga

actagaaaaatcataaataatatattgacaggtaggcccactttcaattattaattttac

gaaaat----tttaggatgaacctctatgaaaaaattctcaaa--cattttgctaataaa

ataagtaaatggctctcaacaggtgggttaataaaatttccaaagaaaacacgcgaaaaa

tttataagggtttttaacttgtctaatcatgacaaattcacatacgaaatattgcta

>18LG3

-----cttagggaatggtttgcaaaaatttagtaaatagagaataaaacaaatcgattaa

gcaaaagtcaaaaatgcacag-ttttcttctgaaattaaagttccaaattttgagaacga

actagaaaaatcataaataatatattaacaagtaggaccactttcaattattaattttac

gaaaaatttttttaggaggaatctccatgaaaaaattttcaaaaccatatcactaatgaa

ataagccagtggttctcaatatgtgggttaataaaatttttgaagaaagtaagcgaaaaa

tgtataagggtttttcactcgtgtgatcatgacaaatttacatacgaaatattgc--

>341LG6

-----attaaggaatggtttgcaaaaatttagtaggtagagaataagacaaatcgattaa

gcaaaagtaaaaaatgcatagatttcctcctgaaattaaagtgccaaattttgagaaaga

actagaaaaattgtaaataatatattgacaggtaggcctactttcaattatcaattttac

gcaaa-tttttt------------------------------------------------

------------------------------------------------------------

---------------------------------------------------------

>214LG9

---------------------------ttaagtacatagagaataagacaaatcgattaa

gcaaaattaaaaaaaacatacttttctttctgaaattaaagtcccaaattttgagaacga

actagtaaaatcctgaataatatattgacaagtaggtccactttcaattattaattttac

gaaaaa------------------------------------------------------

------------------------------------------------------------

---------------------------------------------------------

>30LG7

agcatattagggaaatgtttgcaagaagttagtagatagagaataagacaaatcgattaa

gcaaaagtaaaaaatgcctagtttttcatctgaaaataaagtcccaaattttgagaacga

aatggaaaaatcgtaaatagtatattgacagataggtccactttcaattattaatttcac

gaaaaatctttt--------------atgaaaaaattctgaaaaccatta------taaa

ataagccagtggctctcaacaggtgggttaataaaatttctgaagaaagtacgcgaaaaa

tgtataagggtttttaactcggctgatcatgacaaattcacaaaccaaatattgcta

>57LG5

atcgtgttagaaaatgctttgcaaaaatttagtagatagagaataagacaaatcgattaa

gcaaaagtaaaaaatgcatagtttttctttacaaattaaagtcccaaattttgagaacga

actagaaaatttgtaaataatatattgaccgataggttcactttcaattattaa---taa

gaaaaattttttcaggacgaatctccatgaaaaaattcttaaaaccgttttgctaataaa

attagccagtggctctcaacaaatgggttaataaaatttctgaaaaaagtacgcgaaaaa

tatataagggtttttaactggtctgatcttaacaaattcacatgcgaaatattttta

>75LG5

---------gggaatggtttgcaaaaatttaatatatggagaacaaggcaaatcgattaa

gctaaagtaaaaaatgcatagtttttcttttgaaattaaagccccaaattttgagaacga

attagaaaaatcgtcaataatatattaacaggtaggtccactttcaattattaattttat

aaaaatctttttcaggtcgaaccttcatgaaaaaatttt-----ccatttcgctaataaa

--aagtcagtggctctcaaccggtgggttaataaaatttctgaagaaagtacgcgaaaaa

tgtataagaatttttaattcctctggtcatgact-----------------------

>159LG10

------------------atgcaaaaatttagcagattgagaataagacaaatcgattaa

gcaaaagtaaaaaatgcatagtttttctctctgaattaaagtcccaaatttttagaacga

actagaaatatcgtaaataatatattgattggtaggtccactttcaattattaattttac

aaaattttttttcagaacgaacctccttgataaaattcttaaaaccattttgctaataaa

------cagtggctctcaacagctgggttaataaaatttctgaagaaagtacgcgaaaaa

tgtataatagttttt------------------------------------------

>87LG3

agcgtgttagggaatggtttgcaaaaatttagtaaataaagaataagacaaatcaattaa

gcaaaaaaaaaaaatgcatagttttccttctgaaattaaagtcccaaattttgagaacga

acttggaaaatcgtaaataacatattgac---taggtccactttcatttaataattttac

aaaaaaatttttcagtacgaaccaccatgaaaaaattttcaaaaccacattggctataaa

ataagccagtggttctcaacagatgggt---taaaatttctgaagaaagttcacgaaaaa

t--------------------------------------------------------

>136LG2

agcgtgttagggaatggcttgcaaaaatttagtagatagagaataagacaagtcaattaa

gcaaaagtaaagaatgcatagtttatcttctgaaattgaagtcccaaattttgagaacga

acgagaacaactgtaaataaaatattggcaagtaggtccacttt----tattaattttac

gaaaaattttttcagggcgaaccaccatgaaaaaattctcaaaaccattttg--------

------cagtggctctcaacagatgggttaataatacttttgaagaatgtacgcgaaaat

tgtataagggtttttaacttgtctgatcatggcaaattcaca-acgaaatattgcta

>338LG7

agcgtcttagggaatggtttgcaaaaatttggtagataaagaataagacaaattgactaa

gcaaaaatataaaatgcattgttttccttctgaaattaaagtcccaaattgtgagaacga

accagaaaaatcgtaaataatatattgtctggcaggtccacttt----tattaattttac

aaaaaattttttcaggacaatccttcataaaaaaattctcat------------------

------------------------------------------------------------

---------------------------------------------------------

>155LG6

agcgtgttagggaatggtttgcaaaaatttagtagataaaaaatgagacaaatcggataa

gtaaaagtaaaaaatgcatagtttacctcgtgaaattaaagtcccaaattttgagaacga

actggaaaaatcgtaaataatatattgacaggtgagtctactctcaattattaattttac

gaaaaactttttcaggataaacctccatgaaaaaattcttaaaaccattttgttaataaa

tttaaccagtggttctcaacaggtgggttaataaaatttctgaaaaaat-----------

---------------------------------------------------------

>34LG10

agcgtgttagagaatggtttgcaaaagtttactagatagaaaataagacaaatcaattaa

gcaaaagtaaaaaatgcatagttttccttctgaaaataaagttccaaattttgagaacga

actagaaaaatcgtaaataatatattgacaagtaggtccactttcaattattaattttgt

aa----ttttttcagtacgaacctccagaaaaaaactttcaaaaacattttactaataaa

ataagccagtggctctcaacaggtgggt-----aaatttttttaaaaagtacgcgaacaa

tgtataaaagtttttaaatcgtctgatcatgacaaattcacatacgaaatattgcta

>171LG10

agcgtgttagggaatggtttgcaaaaatttagcaaatacaaaatgagacaaatcga----

-----------aaatgcatacttttccttctgaaattaaagtaccaaattttgagaacga

actagaaaaatcgtaaataatatattgataagtaggttcactttcaattattaattttg-

------------------------------------------------------------

------------------------------------------------------------

---------------------------------------------------------

>264LG10

agcgtgttagggaatggtttgcaaaaatttagcagatagaaaataaaacaaatcgattta

gcaaaagtcaaaaatgtatagtttttcttctgaaattaaagtcacaaattttgagaacaa

gctagaaaaatcgtcaataatatattgacaagtaggtcgactttcaattactaattttac

aaaaatttttttcaggacgaaccttcctgaaaaaa-------------------------

------------------------------------------------------taaggg

tgtataagggtttctaactcgtctaatcatggcaaattcacatacgaaatattgcta

>334LG3

agcgtgttagggaatggtatgcaaaaatttagtaggtagagaataagacaaatcgattaa

gcaaaagcaagaaatgcatagttttccttctgaaattaaagtctcaaattttgagaacga

actagtaaaatcgtaaataatatattg-catgtacgttcactttcaattattaaatttac

aaaaat------------------------------------------------------

------------------------------------------------------------

---------------------------------------------------------

>160LG10

agcgtgttagggaatagtttgcaaaaacttagcaggtagagaataagataattccattaa

gtaaaagtaaaa-atgtataatttaacttctgaaattgaagtctcaaattttgagaacga

actagaaaaaccgtaaataatatattgacaagtaggtccactttcaattattaattttaa

ga----------------------------------------------------------

------------------------------------------------------------

---------------------------------------------------------

>366LG3

agcgtgttagggaatggcttgcaaaaatttagttgataaaaaataagacacatcgattaa

gtaaaagtaaaaaccgcatagtttttcttctaaaattaaagtctcacattttgaggacaa

acttgaaaaatcgtaaataaaatactgacaggtaggtctactttcaattattaattttac

gaaaaattttttcaggacgaacttggataac-----------------------------

------------------------------------------------------------

---------------------------------------------------------

>213LG9

aacgtgttagggaatggtttgcaaaaatttagtagatagagcataagacaaatcgattaa

gcaaaagtaaaaaatgtataggttaccttttgaaattaaagtttcaaattttgagaatga

actagaaaaatcgtaaatactaaattgacaggtaggtttacttttaattattaatttaaa

aaaagt-tctttcagtacgaacctccttgaaaaaac---------aattttgttaataaa

ataagccggtggctctcaacaggtgagttattaaaaattctgaaaaaagtacgcggtaaa

tgtataagcgtttttaactct---gattataacaaattcactttcgaaatattgcta

>19LG2

agcgggttaggaaatggtttgcaaaaatctagtagagagagaataagacaaatcgattag

gcgaaagtaaaaaatgca---gttttcttctaaaattaaagtaccaaattttgaaaacga

aatagaaaaatcataaataatatattgacatgtaagtccactttcaataattaattttac

aaaaaacttattcaag-agaacctccataaaaaaattctcaaaaccattttgttaataaa

atacgccagtggttctcaacaggggggttaataaaaattctgaagaaagtacgcaaaaaa

tgtataagaatcgactgcaca--------tgacaaattcacatacgaaatattgcta

>420LG8

------------------------------------------------------------

------------------------------------------------------------

----------------------------------agtccactttcaat-aataattttac

aaaaaactttttcaggtcgaaccgccaggaaaaaattctcaaaagcattttgctaataca

ataagccagtggttctcaacaggtgggttaatacaatttctgaagaaagtacgcgaaaa-

-----------------ctca--------tgacaaattcacatacgaaatattgcta

>66LG3

agcgtgttaggaaatggtttgcaaaaatttagtagagagaaaataagacaaatcgagtaa

gtaaaagtaagaaatgcatag------ctctgaaattaaagtctcaaattttgagaacga

actagcaaaatcgtaaataatatattgacaggtaggtccactttcaattgttaattttac

ggaaaactttttcaggacgaacctccatgaaaaaattccaaaaaccattattcttataaa

ataaaccagtggctctcaacaggtgggttaatataatttctgaagaaagttcgcgaaaag

tttataagggtttttaactcg--------tgacaaatttatatacgaaatattgcta

>270LG6

agcgtgttagggaatggtttgcaaaaatttagtagatcgagaataaggcaaattgattaa

gcaaaagtaaaaaatgcatagttttccttctgcaattacagatccaaattttgagaacga

aattgtaaaatcgcaaataatacattgacaggtaggtccactttcaattattagtaccac

gaaaaattttttcaagatgaactttcaggaaaaaattctcaaaac---------------

-----ccagtggctttcaacaggtgggttaataaaatttctgaagaaagtacgcgaaaaa

tgtataagagtttttaactcgtctgatcatgacaaattcacattcgaaatattgcta

>51LG2

-----gttgggaaatggtttgcaaaaatttaataaatagaaaataagaaaaatcgattga

acaaaagtaaaaaatgcatagtttttcttctgaaattaaagtcccaaattttgagaacga

actagaaaaaacgtaaataatatattgacaggtaggcccacttccaattattaattttac

aaaaaattttgtctggacgaacctccataaaaaaattttcaaaac-----tgctaat-aa

ataagccagtggctctcaacaggtaggttaataaaatttctgaagaaagtatgcgaaaaa

tgtataagggtttttaacttgtcagattatgacaaatttatatacgaaatattggta

>299LG8

------------------------------------------------caaatcgattaa

gcaaaagtaaaaaatacatagttttccttctgaaattaaagtcccaaacgttgagaacga

actaaaaaaaacgtaaataatatattgccaggtatgtccatgttcaattatttattttac

gaaaaaacttttcagaatgaacctccaggaaaaaattctaaaaac---------------

-----ccagtggctctcaacaggtgggttattaacatttctgaagaaagtacgcgaaaaa

tg-----------ttaactcgtctgatcatgacaaattcacatacgaaatattgcta

>58LG2

-------------ttggtttgcaaaaatttagtagatagagaataagacaaatcgattaa

gcaaaggtaaaaaatgcatagttttccttctgaaatt-aggtcccaaattttgagaacga

aataaaagaatcgtaaataatatattgacaggtaggtccactttcaattttaaattttac

gaagaacttttttttgacaaacttccaggaaaaaattctcaaaaccattttgctaaaaaa

ata----agtagctctcaacaggtgggttaataaaatttctgaagaaagtacgcaaaaaa

tgtacaagggtttttaactcgtctgattatgacaaattcacatacaaaatattatta

>20LG10

agcgtgttaggaaatgctttgcaaaaatttagtatatagagaataagacaaatcgat---

------gtaaaaaatccatagttttccttttgaaattaaagtcccaaattttgagaacga

actagaaaaatcgtaaataatatattgacaggtaggtccactttgaattattaattttac

aaaaaattttttctggaaaaatctcc---gaaaaattttcaaaat---cttgctaataaa

ataagccagtggctctcaacaggtgggttaacaaaatttctgaagaaagtacgcgaaaaa

tgtatac-ggtttttaacttgtctgatcatctaaaattcacacacgaaatattgcta

>24LG10

agcgtgttaaagaatggtttac-aaaatttagtagagagagaataagacaaatcgattaa

gcaaaagtaaaaaatgcatggttttccttctgaaattaaagtcccaaattttgagaacga

actaaaaaaatcgtaaataatatattga-aggtaggtccactatcaattattacttttat

gaaaaa-tttttcaggacgaacttacataaaaaatttctcaaaaccatttttttaataaa

ataagccagtggctctcaacaggt-ggttaataaaatttctg----aagtacgcggaaaa

tgtatacgggtttttaactcgtctgatcatgacaacttcacatacgaaatattgcta

>350LG3

agcgtgttagggaatggtttgcaaaaatttagtagatagagaataagacaaatcaattaa

gcaaaagtaaagaatgcatagttttccttctgaactcaagatctcaaattttgagaacga

acttgcaaaataa----------------aggta--------------------ttttac

aaaaaattttttcaggacgaacctacatgaaaaaattctcaaaaccatttttctaataaa

ataagccagtggctctcaacaggtgggttactaaaatttctgaagaaaaaacgtggaaaa

tgtatatgagttttcaactcgtctgatcatgacaaattcgcatacgaaattttggta

>22aLG3

agcatgttaggaaatggtttgcaaaaatttggtatatagagaataaaacaaatcgattaa

acaaaagtaaaaaatgcatagttttccttctaaaattaaagccacaaattttgagaacaa

actagaaaaatcgtaaataatatactggcaagtgggtccactttcaaat-------ttac

gaaaaaatttttcaggacgaacctccatgaaaaaattttcaaaaccattttgctaataaa

aaaagccagtggctgtcaacaggtgggataataaaatttctgaagaaagta--cgaaaga

tgtgtcatggtttttaactcatctgatcatgacaaattcacatactaaaaattgcta

>109LG10

agcatgttagggaaatgtttgtaaaaatttcgtacatagagaataagacaaatcgattaa

gcaaaagtaaaaaatgcatagtttttcttctgaaattaaagttccaaattttgagaacga

actagaaaaatcataaatagtatattaacaggtaggtccacttgcaatattaatttttac

a-----atttttcaggacgaacctctatgaaaaaattttc-aaattattttgctaataaa

ataagtcagtggctctcaacaggtgggttaataaattttccgaagaaagtacag------

---------------------------------------------------------

>147LG10

--------------tggtttgcaaaaatttagtagatagaggaaaagacaaatcgattga

gcaaaagtaaaaaatgcatagttttacttctgaaattaaagtcccaaattttgagaacga

actagaaaaatcgtaaataatatattgtgtggtaggtccactttcaattattaatttttc

g-----ttttttcagggcgaatctccgtaaaaaaattctcaaaaccattttg-------a

ataagacagtggttctcaacaggtgggttaataaaatttc----agaattacgcgaaaaa

tt-------------------------------------------------------

>94LG10

---------ggaaatggtttgcaaatactcaatagatagagcataaaacaaatcgattaa

gcaaaagtcaaaaatgtatagttttttttccaaaattaaagtcccaaattttgagaacga

actagaaaaatcgtaaataatatattgacaggtaaatccacttttaattattaattttgc

aaaaacttttttcaagacaaacctccataaaaaaattctcaaaaccattttgttaggaaa

------cagtggctctcaacaggagggttattaaaatttctaaaaaaagtacgcaaaaaa

tgtctaaggatttttaattcgtctgatcatgacaaattcacatacgaattattt---

>153LG9

agcgtgttaggaaatggtttgcaaaaatttagttgatagagaataagacagatcgattaa

gcaaaa--------ttgatagttttccttctgcaattaaagtcccaaattttgagaacga

actaaaaaaatcgtaaataatatattgagaggtagatccactttcaattattaattttac

gaaaaattttttccggacgaactttcatggaaaaattctcaaaaccattttgctaacgaa

------cagtggttcccaacagttgggttaataaaatttctgaagaaagtacgggaaaaa

tatgtaagggtttttaactcctctgatcatggcaaattcacatt-------------

>163LG7

agcctgttagggaatggtttacaaaaatttagtagatagagaataaaacaaatcgattaa

gcaaaaattagaagctaatagttttccttctgaaatgaaagtcccaaa-tttgagaagga

actagaaaaattgtaaataata-attgacaggtaaactcattttcaactactaattttac

aaaaaattttttcagaaggaacctccataaaaaaattctcaaaaccatttagctaataaa

acaaaccagtggctctcaacaggtgggttaataaaatttctgaagaaaatacgcgaagaa

tgtataagggtttttaactcgtctgatgatgacaaattcacaaacaaaatattgcta

>27LG10

-------------------------------gtagatagagaataagac--atcgattaa

gcaaaagtaaaaaatgcatagttttccttctgaaattaaagtcccaaattttgagaacga

actagaaaaatcgtaaataatatattgacaggtaggtccactattaattatcaattttat

gataaattttttcaggacgaaccttcatgaaaaaattctcacgactattttgcttataaa

gtaagccagtggctctcaacaggtgggttaataaaaattctgaagaaagtaggccagaaa

tg-ataagggtttctaactcgtctgatcatgacaaattcgcatacgaaatattgcta

>439LG9

------------------------------------------------------------

--caaagtaaaaaatgaataattttccttctgaaggcaaagtgccaaattttgggaaca-

--tagaaaaatcgtaaataatacattgacaagtcggcccactttcaattattaattttaa

g-----tttttttaggacgaacctcc-----aaaattctcaaaac---------------

------------------------------------------------------------

---------------------------------------------------------

>29LG10

--cgtattaggaaatggtttgcaaaaacttagtagatagagattaagacaaatcgattaa

gcaaaagtaaaaaatgcatag-tttccttttaaaattaaggtcccaaattttgagaacaa

actagaaaaatcgtaaataatataccgacaggta--tccactttcaattattaattttac

aaaaaattttttcaggacgaacctcccagaataaattctcaaaaccattttgctaataaa

ctaagtcagtggctctcaacaggtggattaataaaatttctgaagaaagtactcgaaaaa

tgtataagggtttttaatttgtctgatcataacaaattaacatacgaaatattgata

>63LG10

-----------------tttgcaaaaatttagtagatagagaattgaacaaatcgattaa

gcaaaagtaaaaaatgcatagttttcattctgaaattaaagtcccaaattttgagaacga

actggaaaaatggtaaataatatactgataggtatgcctcctttcaattatcaattttat

aaa---ttttttcaggataaacctccatgaaaaaattatcaaatctattttgctaataaa

ataagcca--ggctctcaacaggtgatttaataaaatttctgaagaaagtacgcgaaaaa

tg-acatgggttttcaactcatctgatcatgacaaattcacataagaaatattgcca

>91LG9

agcgtgttagtgaatggtttgcaaaaatttagtaattagaaaataagacaaatcaattta

gcaaaaataaaaaacgcatagatttcctc--------------ccaaattttgagaacga

actaagaaaatcgtaaataatatattcacaggtaggtccact-----ttattaattttac

aaaaaattttttcaggacgaaccaccataaaaaaaattttaaatccattt----aataaa

ataagccagtggctctcaacaggtgggctaataaaatttctgaagaaagtactcgaaaaa

tgtattagggtttttaactcgtctgatcatgacaaattcacgtatgaaatattgctg

>37LG4

-------------------------------------------------aaatagattaa

gcaaaagtaaaaaatgcacagttttccttctgaagt-aaagtcccaaattttgagaacga

actagaaaaatcgtaaataatatactgacaagtaggtccactttcaattattaattttac

gaaaaattttttcagaacgaacctccatgaaaaaattctcaaaaccatttttctaatgaa

ataaaccagtggctctcaacaggtgaggtaataaaatttctgaagaaagcacgggaaaaa

tgtatgagggtttttaactcgtttgatcatgaaaaattcacatacaaagtattgcta

>178LG3

------------------------------------------------------------

------------------------------------------------------------

------aaaatcgtaaataatatcttaacaggcaggtccactttcaattattaattttac

gaaaaactttttcaggacgaacctccaggaaaaaattttcaaaaccattttgctaataaa

ataagccagtggttctcaacaagtgggttaataaaatttctgaagaaagtccgcgaaaaa

tgtacaagggtttttaactcgtttgatcataacaaattcacaggcgaaatattgcta

>158LG5

------------------------------------------------------------

----------------------------------------------aattttgagaacga

actagaaaaatcgtaaataatatattgccaagaaggtccacttaacattattaattttac

gaaaaactttttctggatgaacctccaggaaaaaattctaaaatctattttactcataaa

ataagccagtggctctcaacaggtgggttactaaaatttctgaagaaagtacgcgaaaaa

tgtataagggtttttaactcgtctgatcatggcaaattcacatacgaaatattgcta

>112bLG9

--------------------------------------aagattaaaataaatcgattaa

gcaaaattaaaaaatgaatagttttcct-------------tctaaaattttgagaacga

actagaaaaatcgtaaataatatatttacatataggtcaacttttaatca-taattttac

gaaaaattttttcagaatgaaccttcatgaaaaaatttttaaaaccaccttggtaataaa

ataagccagtggccctcaacaggtgggttaataaaacttctgaagaaaatacgcgaaaaa

tgtataggggtttttaactcgtctgaccatgataaattcacatacaaaatattgcta

>173LG8

------------------------------------------------------------

--------------------------------------------aaaaatttgggaacga

aatagaaaaatcgttaataatatattgacaggtaggtccattttcaattataaattttac

gaaaaatgtgttcaggacgaacctccaggaaaaaattcctaaaaccttcttgctaataaa

ataagtcagtggctctcaataggtgggttaataaaatttctgaagaaaatacgcgaaaaa

tgtataagggtttttaactcgtctggtcatgacaaattcaca---------------

>114LG6

-----------------tttgcaaaaatttagtagatagagaaaaagacaaatcgatcaa

gtaaaaataaa----------------tcctgaaattaaagtcccaaattttgagaacga

actagaaaaatcgtaaataatacattgacagatgagttcactttcaattataaattttac

gaaaaagtttttcaggacgaacgtccatgaaaaaattttccaacccattttgctaataaa

ataggccagtggctctcaacaggtgggttaagaaaatttttgaagaaaatacgcgaaaaa

tgtatgagggtttttaactcgtctgatcattacaaattcacacaaaaaatattgcta

>45LG10

------------------------------------------------------------

--------------------------------------------gaaattttgagaacga

actagaaaaatcgtatacaatatatagacaggtaggaccactttcaattattaattttaa

gaaaaattttttcgggacgaacctccatga-ataattcttaaaaacgtttggctaataaa

ataagctagtggctctcaacaggtgggttaataaaatttctgaaaaaagttagc------

---------------------------------------------------------

>98LG7

-------------------------------------------------------attaa

gcaaaagtaaagaatgtgtatttttttttctgaaattaaagtcccaaattttcagaacga

attagaaatatcgtaactaatatattaacaggtgggtccattttcaattattaattttac

gaaaaactttttcagaacgaacctccttgataaaatttttaaaaccattttgctaataaa

ataagccagtggttctcaacaggtgggttaataaaatttctgaagaatttaagc--aaaa

cgtgtaaaggtttttaacttctctgattatgacaaattcacgtacgaaatattgcta

>137LG7

------------------------------------------------------------

--aaacgcaaaaatgcacaggttttccttccgaaattaaagtcccatattttgagaacga

actagaaaaatcatacataataatttgacagttaggttcactttaaattattaattttac

gaaaaatttttttttgacgtacctccatgaaaaaattctcaaaaccaatttgctaataaa

ataagccagtggctctcaacaggtggcttaataacatttctgaagaaagtacgcgaaaga

tttataaagatatttaactcgtctgatcatgacaaattcacatg-------------

>253LGX

------------------------------------------------------------

-----------------------------------------tcccaaattttgagaacga

actggaaaaatcataaataataaattgacaggtaggtccattttcaatcattgattttaa

gataca---attcatgacgaaccttcataaaaaaattctcaaaaccactttgctaataaa

ataagccagtggctctcaacaggtgggttaataaaatttctgaagaaactacac------

---------------------------------------------------------

>138LG8

-----------------tttgcaaaaatttactaaatagagaataagat-----------

gcaaaaataaaaaaattatagtttcccttctcaaattaaagtcccaaattttgagaacga

actaaaaaaatcataaataatatattgacaagtagatccattaacaattcttaattttac

gaaaaattttttcaggacgaacctccatgaaaaaattcttaaaaccactttgcaaataaa

atgagccagtggctctcaacaggtgggttaataaaatttctgaccaaagtacgcgaaaaa

tgtataagggttttagactcatctgaa------------------------------

>283LG10

------------------------------------------------------------

---------------------------ttctgaatttaaagttccagattttgagaacga

tctagaaaaatcgtaaataacaaattaacaagtagatccactttcaattattaattttac

gaaagatttttgcgggaggaacctccatgaaataattcttaaaacaattatgcaaataaa

aacagccagtggctctcaacaggtgggttaataaaatttctgaagaa-------------

---------------------------------------------------------

>172LG10

------------------------------------------------------------

-----------aaatgcatag-ttttcttctgaaattaaagtctcaaattttgagaatga

actagaaaaatcataaataatataatgtcaggtaggcccacttttaatttttaattttaa

gaaaaattttttcgagacgaacctccatggaaaaattctcaaaaccattttgctaataaa

ataagacagtggctctcaacaggtgggttaataaaatttct----aaagtacgcaaaaaa

tgtataaggg-----------------------------------------------

>187LG10

------------------------------------------------------------

---------aaaaatgcatcgttttccttctgaaatttaagtttcaatttttgaaaacga

actaaaaaaatcgtaaataatatattgccaggtaggtctactttcaattattaatttaag

gaaaaattttttcaggatgaaccttcaagaaaaaattttaaaaactattttactaataaa

ataagccagtggctcccaacaggtgcgttaataaaattt-tgaagaaagtacgcaaaaaa

tg-ataaggatttttaactcgtctgattatgacaaattcacgtacgaaatattgcga

>32LG6

agcgtgttagggaatggtttgcaaaaatttaacaaatagagactaaaaaaaaacgattaa

gcaaaactaaaaaatgcatagtttcccttctgaaaatttggttataaattttgaaaacga

actaagaaaatcgtaaataatatattgacaggtaggtccactttcaattattaatttttc

aaaaaa-tttttcagaacaaacctccatgaaaaaatcttcaaaaccattttgctaataaa

ataagccagtggctctcaaccggtaggttaacaaaatttctgaagaaaatacgcgaaaaa

tttataagggttgttaactcgtctgattaagagaaattgacatacgacgtagtgcta

>39LG3

agcgtgttagggaatggttttc-aaaattttgtagatagagaataagataaagcgattaa

gcaaaagtaaaaaatgcatagttttcc-tttgaaatttatgcctcaaattttgagaacga

actagaaaaatcgtaaataatattttgacaggtaggtccactttcaattagtaattttac

aaaaatttttttcagaacgaacctccatgaaaagattttcaaaaccattttgctaataaa

ataagacagtggctctcaacaggtgggttaataaaatttctggagaaagtacgcgaaaaa

tgtgtaagggtctttaa----------------------------------------

>190LG10

------------------------------------------------------------

-------------atgcatagttttccttccgaaattaaagtcccaaattttgagaacga

actagaaaaatcgtaaatagtatattgacaggtaggtccactttcatttattgattttac

gaaaaa-tttttcaggacgaacctccatgaaaaaattttcaaaacc---ttgttaataaa

ataagtcagtggctctcaacaggtgggttaataaaaattctgaagaaactacgcggaaaa

tgtataagggtttttaaca--------------------------------------

>229LG10

------------------------------------------------------------

--------------------------------------aagtcccaaattttgagaacga

actagaaaaatcgtaaataacatattgacaggtaggtccactttcaat-----attttcc

aaaaaattttttcgggacgaacctccaggaaaaaattctcaaaatcattttgttaataca

ataagccagtggctctcaacaggtgggttaataaaatttctaaagaaagtacgcaaaaaa

attataagagtttttat----------------------------------------

>68LG6

agcgt-ttagggaatgctttgcaaaaatttagtagatagagaataagacaaatcgattaa

gcaaaagtaaaaaatgtatagtttttc------aaaaaatatcccacattttgagaacga

actagaaaaatcgtaaataatatattgacaggtag-------ttcaattattaattttac

g-----ttttttcaggacgaatctccatggtaaaatcttcaaaaccattttgctaa----

------------------------------------------------------------

---------------------------------------------------------

>106aLG9

agcgtgttagggaatgatttgcaaaaatttagtagatagagaataagacaagtcgattaa

gcaaaagtaaaaaatgcatagtttttc---------aaacgtcccaaattttgagaacga

actataaaaatcttaaataatataatgtcagataggtccacttacaattattaatttttc

aaaaagttttttcaagacgaactttcatgaaaaaattttcaaaaccattttgctaataaa

acaagccagtggttctcaacaggtgggtgaataaaatttctgatgaaagtacgcgaaaaa

tctatatgggtttttaactcgtct---------------------------------

>148LG7

------------------------------------------------------------

----aagtaaaaaatgcataactttttttctgaaattaaagttccaaattttgagaacga

attagaaaaatcgtaa-tagtatattgacagataggt---ttttcaattattaattttac

gaaaaactttttcacgacgaacctccataacaaaatttttaaaaccatcattctaataaa

ataattcagtggctctcaacaagtgggttaatgaaatttctgaagaaagtacgcgaaaaa

tgtataagggttttcaactcgtctaatcataacaaattcacatacgaaatattgcta

>74LG9

agcgtgttagggaatggtttgcaaaaatttgatagatatagaataaaacaaatcaataaa

gttattataaaaaatgcatagttttcttt--------aaaatccaaaattttgagaacga

actataaaaattgtaaataatatattgacaggtaggtcaactttcaattattaattttag

gaaaaattttttcataatgaacctccaagaaaaaaatctcaaaaccattctgttaataaa

aaaatccagtggctctcaaaaggtggtttaataaaatttctgaagaaattacgtgaaaaa

tgtataagggtttttaacccgtctgatcgtgacaaattcacatacgaaatattacaa

>56LG3

--------------------------------------gggcat-agacaaatcgattaa

gcaaaagtaaaaaatgcatagcttcccttccgaagttaatgtcccaaattttgagaacga

actagcaaaatcgtaaataatataatgacaagtaggaccattttcaattattaattttac

gaaaaatcttttcaggacgaacttccatgaaaaaattctcaaaaccatttcgctaataag

ataagccagtggctcccaacagatggattaacaaaatttctgaataaa------aaaaaa

tgcataagggtttttaactcgtctgatcgtgaaaaagtcaaatacgaaatatttcta

>166LG4

------------------------------------------------------------

---------------------------atctgaaattaaagtccaaaattttgagaacgg

aatagaaaaatcgtaaatgatatattgacaggtaggtccacttttgattattaattttaa

gaaaaa---tttcagaacgaacctcca-aaaaaaattctcaaaaccatttagctaataaa

ataggccagtggcactcaacagttggattaataaaattt-tgaagaaaatacgcaaaaaa

tatataagggtttttaactcg-----ttttatcaaattcacatacgaaatattgcta

>47LG3

------------------------------------------------------------

--aaaagtaaaaaatgcatagttttctttctgaaaatgaagtcccatattttgagaacga

aatagaaaaactgttaataatttattgacaggtaggtccactttcaattattaattttaa

gaaaaattttttcaggacgaagctccatgaaaaaaatctcaaaaccatttagctaataaa

ataagccagtggctctcaacagttgggttaataaaatttctgaagaaagtacgctaaaaa

tgtataagggtttttaactaacctgatcacgaaaaattcacatacaaaatattacta

>248LG3

------------------------------------------------------------

-------------------------------gaaattgaagcctcaaatttggagaacga

actagaaaaattgtaaataatatgttgacagataggttctc-ttcaattattaattttaa

gaaaaattttttcagattaaacatccctaaaaaaattctcaaaaccattttgctaataaa

ataagccagtgactcttaaccggtgggctaacaaaatttctgaaaaaaatacgcaaaaaa

tgtataaaggtttttaactcc------------------------------------

>53LG3

agcatgttagggaatggtttgcaaaaatttagtagatagagaataaaacaaataaattaa

gcaaaagtaaaaaatgcaatagttttcttctgaaattaaagtcccaaattttgagaacga

attagaaaaatcatgtataatatgttcacaattaggtccacttgcaaatattatttttac

gaaaaa----atcactacgaacctccatg-aaaaattcccaaaactattttgctgataaa

ataagacagtggctctcaacaggtgggttaataacattcccgaagaaagtacaggaaaaa

tgtataagggtttttaactcgtctaatcatgacaaagtcacatacgaaatattgcta

>296LG6

agcgtgttagggaatggtttgaacaaatttagtagatagagaataaaacaagtcgattaa

gcaaaagtaaaaaatacatagttttccttttgaaattaaagtccctaattttgagaacga

actagaaaaatcg-------------------taggtccactttcaatta-taatattac

gtaaaattttttcaggacgaacctccatgaaacaactctcaaaaccattttgctaat-aa

aca----actaattctcaacatgtgagttaataaaatttctgaagaaagtattcgaaaaa

tgtatataggtttttaactcgtctgatcatgacaaattcacatacgaaatattgcta

>60LG10

aacgtgttaggaaatcatttgcaaaaatttagtatatagagaat-----------attaa

gcaaaagtaaaaaatgcatagttttccttttgaaat-taaatcccaaattttgagaacga

a-tagaaaaatcgtaaataatatatggacaagtaagtcaattttcaattattaattttac

gaaaaaatttttcaagac-------catgaaaaacttctcgtaaccattttgctaataaa

aaaagccagtggctctcaacatgtgagttaataaaatttttgaagaaagtactcgaaaaa

tatataagggtttttaactcgtctgatcatgacaaattcacattggaaatattgcta

>271LG3

--------------------------------------------------------ttaa

gcaaaagtaaaaaatttatagttttcc----------aaagctccaaattttgggaacga

actagaaaaatcataaataataaattgacaggtaggtctactttcaattactaattttaa

gaaaacttttttcaggac--------ataaataaaatctcaaaaccattttgctaataaa

ataagccagtggctctcaacagatgggttaataaaattt------------cgcgaaaaa

tgta----------caactcgtctgatcatgacaaattcacatgcgaaattttgcta

>6LG3

--agtgttagggaatggtttgcaaaaatttattatataaagaataagacaaatcgattaa

gcaaaagtaaaaaatgcatggttttccttctcaaattaacgtcccaaattttgagaacga

actagaaaaatcgtaaattatatattgacaggtaggtccactttcatttattttttt---

--acaattgtttcgggacgaaactccatgaaaaaattctcaaaaccattttgctagtaaa

ataagccaatggctctcaacaagtgggttaataaaaattctggagaaagtacac-aaaaa

tgtatatggatttttaactcgtctgatgatgacaaattcacataggaaatattgcta

>38LG7

-------aggtatatggtttgcaaaagtttactagatagagaataagacaaatcgattaa

gcaaaagtaaataatgcatggttttccttctgaaaataaagtcccaaattttgagaacga

actaaaaaaatcgtcga---aatatcgacagattggtccaccttcaattattaattttat

gaaaaattttttcaggacgaaccttcatgaaaaaattcccaaaaccactttgctaataaa

ataagccagtggctctcaacaggtgggttaataaaatttctgaagaaagtacgcaaaaaa

tgtacaagagtttttaactcatctgctcatgac-aattcacttacgaaactttgcta

>42bLG10

------------------ttacaaaaattcagtagatcgaaaataaaacaaatcgattaa

gtaaaagaaaaaaatgcatg-ttttccttctgaaattaaagtcccaaattttgagatcga

tctagaaaaatcgtaaataatatattaacaggcaggtccactttcaattattaatttta-

-aaaaattttttcaggaggaaactcc-----aaaattcttaaaatcatattgctaataat

ataagccagtggctctcaacaggtgggttaataaaatttctgaataaagtacccaaaaaa

tgtataagggtttatacctcgtcagatcatgacaaatttacataaaaaatattacta

>387LG10

------------------------------------------------caaatcgattaa

gcaaaaggaaaaaatgcatagttttttttctgaaattaaagtcctaaattttgagaacga

actagaaaaagcgtaaataatatattgctaggtaagttcacgttcaattattaattttac

aaaaaattttttcaggacgaacctc----------------aaaacattttactaataaa

ataagccagtggctctcaacaggtgggttaataaattttttaaaaaaagtactcgaaaaa

tatataat-------------------------------------------------

>177aLG3

--------------------------------tagatattgaataaaacaaatcgattaa

gcaaaagcaaaaaatgcatagtattct--------------tctcaaattttgagaacga

actagaaaaatcgcaaataatatattgtcaagcaggtccactttcaattattaatgttac

gaaaaatttttccaggacgaacctccattaaaaaaatctgg----cattttgctcataag

ataagccagtggctctctacaggtgggttaataaaatttctgaagaaaatacgca-aata

tgtacaagggtttttaactcatctgatcccggcaaattcacatccgaaatattgcta

>390LG7

----------------------------------------------aacaaatcgattaa

gcaaaagtaaaaaatgcatagtttttcttctgaaattaaaattccaaattttgagaacga

actaaaaaaatcgtaaataatatattgtcaagtagatccattttcaattatcaattttac

gaaaatcttttccaggacgaacctccataaa--------------cattttgctaaaaag

ataagccagtggctctctacaggtgggttaataaaatttctgaaaaaagtacgcagaaaa

tttacaagggtttttaactcatctga-----------tcacagccgaaatattgcta

>433LG10

---------------------------------------------aagcaaatcgattga

gcaaaagtaaaaaatgcatagttttccttcaagaattaaagtcccaaattttgagaacga

actagaaaaatggaaaataatatatcgtcaggtaggtccatt-------attaatcttac

aaaaaatttctccaagacgaacctccatgaa-------------------tgctaataaa

ataagccagtggttctcaacagctgggttaataaaatttcagaagaaggtacgcgaaaaa

tatatg---------------------------------------------------

>72LG3

------------aatggtgtgcaaaattttaatagataaagaataagacaaatcgattag

gcaaaagtaaaaaatgcatccttttccttctgaaattaaagttccaaattttgagaacga

actagaaaaatcgtaaacaatatattgacaggtaggtccactttaaattattaaatttac

aaaaacttttttaaggacgaacctccatgaaaaaattctcaaaatcattttgctaataaa

ataaggctgtggctctcaacaggtgggttaataaaattcctgaagaaagtactcgaaaaa

tttatacgagtttttaatttgtctgatcatgacaaatttatatacgaaatattgcta

>33LG5

-------------------tgcaaaaatttagtagatagagaataagacaaatcgattaa

gtaaaagtaaaaaatgaatagttttccttctgaaattaaagttccaaattttgagaacga

actagaaaaatcgtaaataatatattgacaggta--------ttcaattattaat-----

-aaaaattttttcagaacga-cctccatgagaaaattctcaaaaccatttagctaataaa

atccgtcagtggctctcaacaggtgggttaataaaatttctgaaaaaaatacacgaaaaa

tttataagggttttcaacgcgtctgattataataaattcacacacaaaatattgcta

>378LG3

-------------------tacaaaaatttagtagatagagaatatgacaaattgattaa

gcaaaagtaaaaaatgcatagttttccttttgaaattaaagtgccaaattttgaaaacga

attagaaaaatcgtaaataatatattgacaggtaggtccactttcagttataatttttt-

--cgaactttttcaagacga-----------aaaattctcaaaaccattttgataatgaa

ataagccagtggctctcaacaggtgggttaataatttttttgataaaggaacgcgaaaaa

tgtataagggtttttaactcgtctgatcataataaattcttgtatgaaatattgcta

>92LG9

-----------------tttgcaaaaatttagtagataaagattaaaacaaattgattaa

gcaaaagtaaaaaatgcataattttctttctgaaattaatgtctcaaattttgagaacga

attaaaaaaatcataaataatatattcacgggtaggtgcactttcaattgttaattttcg

aaaaaattttttcaggatgaacctctatgaaaaaaatcttaaaatctttttgctaataaa

------cagtggctctcaacaggtgggttaataaaatttctgataaaagttcacagaaaa

tgtataacggtttttaactcgtctgattatgacaaattctcatacgaaatattgcta

>16LG10

agcgtgttagggattggcttgcacaaatttagtagacagagaataagacaaatcgattaa

gtaaaagaaaaaaatgcatagttttccttttgaaattaaaataccaaattttgagaacga

actaaaaaactcgcaaataatatattggca-ataggtttacttttaattattaattgtac

gaagaattttttcagtacgaacctccatgaaaacattttcaaaaccaatatgctaataaa

ataagtcagtggctctcaacaggtgggttaataaaatttctgtcgaaaattctggaaaaa

tgtataaaggtttctaactcgtctggtcatgacaaattcacatacgaaatattgcta

>46LG8

-------------------------------------agagaataagacaaatcgattaa

gcgaaagtaaaaaatgcatagttttccttctgaagttaaagccctaaattttgagaacga

actagaaaaatcgtaaataatatattaacagataggtccactttcaattattaattttac

gaaa--ttttttcaggacgagtctctatgaaaacatttttcaaaccattatgcaaataaa

aaaagccagtggctctcaacagatgggttaacaaaatttctgaa---------ggaaaaa

tgtgtaaaggtttttaactcgtctaattatgacaaatttacatacgaaatattgctt

>258LG10

------------------------------------------------------------

------------------------------------aaaaatcccaaattttgagaacga

tgtagaaaaatcgtaaataataaattaacgggtaggtccactttcaactatttattttac

gaaaaattttttcaggacgaacctgcataaaaaaattctcaaaaccattatgcaaataaa

aaaagccagtggctctcaacaggtgggtaaataaaatttctga-----------gaaaaa

tttatga--gtttttaactcgtctgatcatgac------------------------

>200LG10

agcgtgttagggaatgatttgtaaaaatttagcaaataggg---aaaacaaatagattaa

gcacaagtaaaaaatgcatagtttttc----------aaagtcccaaattttgagaacga

actagataaatcgtaaataatatattggc-----ggtctactttgaattattaattttac

aaaaaagtttttcaagacggacttccctgaaaaaatttttaaaaccattttactaacaaa

ataagtcagtgaatctcaacaagtgggttaataaaatttcagaaaaaa----tagaataa

tgtttaagggtttttaactcgtctggtcatgacaaatttacatacgaaatattgcta

>185LG8

cgcgtgataggaaatagtttgcaaaattttagtagaa-------aagacaaatctattaa

gcaaaaataaaaaatgcatagttttcttactgaatttaaagtcccaaattttgagaacga

cctacaaaaatcttaaataatatattaactggtaggttcactttcaattattaattttac

gaaaaattttttcaggacgaacctctataaaaaaattctcaaaaccgttatgcaaataaa

gaaagccagtggctctcaacaggtgggttaataaaatttttgaa--------gtaaaaaa

tttagaaaggtttttaattcgtctgattataacaaattcc-----------------

>117LG3

agcgtgttagggaatggtttgcaaaaatttagtagaaagagat------------attaa

gcaaaagaaaaaaatgcatagttttccttctgaaattaaattcccaaattttgacaacga

tctagaagaatcgtaaataatgtattaacaggtaggttcacttttaattataaattttac

gaaaac------ccaggcggacctccaagaaaaaattctcaaaaccattatgcaactaaa

aaaagccagtggctctcaacaggtaggttaataaaacttctgaagaaaa---ccgaaaaa

tgcgtacgggtttttaattcgtctgagcatgacaaattcacatacgaaatattgtta

>316LG10

agcgtgttagggtatggtttgcaaaaatttag----tagagaataagacaaatctattaa

gcaaaagtaaaaaatgcatagc----------------aagtcccaaattttgagaacga

agtagaaaaatcgtaaataatatattgacaggtaggaccacttttaattattaattttac

gaaaaattttttcaggacgaacttccatgaa---------gaaaccattttgttaataaa

ataagccagtggttctcgacatttgggttaataaaatt----------------------

---------------------------------------------------------

>96LG8

agcgtgatagggaatggtttgcaaaaatgtagaagatagagaataaaacaaatctatta-

--aaaagtaaaaaatgcaa--ctttccttccaaaattaaagtttcaaattttgagaacaa

actaaaaaaatcttaaataatgtactgacaaataggtccactttcagttattaattttac

gaaaaattttttcaggacgaagttctatgaaaaatttttcaaaatcattttgctaataaa

ctaagccagtggttctcaacagttgggttaataaaatttctgaagaaagtacgcggaaaa

tg-atgtaagtttttaactcatctgatcatgt-------------------------

>220LG9

-----------aaatggtttgcaaaaatttagtagacagagaat--------------aa

gcaaaagtaaaaaatgcatagctctccttctgaaattaaagtcccaaattttgagaacga

actagaaaaatcgtaaatgatatattaaaaggtaggacaattttcagttattaattttag

gaa---ttttttcagga-------ctatgaaaaaattctcaaaaccatttgtctaataac

ataagccagtggttctcaacaggtggattaataaaatttcgaaagaaagtacgcgaaaaa

tg-----aggtttttaactcgtctga-------------------------------

>286LG10

------------------------------------------------------------

---------------------------tttcaaaacataagttccaaattttgagaacga

actagaaaaatcgtaaataatatattgacaaggaggtccactttcaattattaattttac

gaaaaa--ttttcaggacgaaactccatgaaacaattcttaaacccattttcctaataaa

ataaaacagtggctctcaacaggtgggttagtaaatttctaaaaaaaagtgcgcgaaaaa

tg-------------------------------------------------------

>139LG3

agcgtgttagggaatggtttgcaaaaatttggcagatagagaataagacaaatcgattaa

gaaaaagtaaaaaatgcatagttttcc----------atcgttcaaaattttgagaacga

actggaaaaatcgtaaatggtatattgacaggtaggtccacttttaattattaattttat

gaa---ttttttca-gacaaaacaccatgataaaattctcaaaaccattttgctaataaa

ataaactactgatgctcaacaggtgggttaacaagaaa----------gtacgcgaaaaa

tgtataagagtgtttaattcgtc-gatcatgacaaattcacatacgaattattgcta

>71LG7

agcgtgttagggaatggttcgcaaaaatttggtagacagagaataagaccaatcgattaa

gtaaaagtaaaaaatgcatagttttcc--ccgaaattaaagtctcaaattttgaaaacga

aatagagaaatcataaataatatattgacaggtaggtccactttcaattattaatcttat

gaaagaatttttcagaacgaactccagtaaaaaatttctcaaaaccattttgctaataaa

ataagccagtggctctcaacaggtgggtttaacaaatttctgaagaaagtacaggaaaaa

tgtataag--tgtttaactcgtctgatcatgacaaattcacatttgaaatattgcta

>115LG8

agtgtgttaggaaagggtttagcaaaatttagggaatagagaatgagacaaatcgattaa

gcacaagtaaaaaatgcatagttttccttttgaaat-------------tttgagaacga

acaagaaaaatcgtaaataatatattgataggtagatccactttcaatgattaattttac

aaaaaaatttttcaggccgaacctccatgaaaaaattctcaaatctactttgcaaataaa

ataagccagtggctctcaacaggtggggtaataaaatttctgcagaaagcacgggaaaaa

tgtacaagggtttttaactcgtctaat--------------------actattgcta

>219LG10

-------------------------------------agagaataagtcaaattgattaa

gcaaaagtaaaaaatgcatagtttttc-----aaatcgaaa----aacttttgggaacga

actataaaaat--taat-aatatattgacaagtagatccactttcaattattaatttttc

gaaaaattttttcaggtcgaacctctatgcaaaaatttttaaaaccatttttctaataaa

aaaagccagtggctctcaacaggtcggttaataaaatttttgaagaaagtaggcgaaaaa

tatattacagtttttaatttgtttgataatgacaaattcacatacgaaatattggta

>254LG10

------------------------------------------------------------

------------------------------------------------------------

---------at------------attgacaggtaggtccactttcaattattaattttaa

gaaaaattttttcgggacaaacgtccatgaaaaaattctcaaaaccattttactaataaa

ataagccagtggctatcaataggggggtaaataaaatttctgaagaaagtacgcgaaaaa

ggtataagggtttttaactcgtctgattatgataaattcacatacgaaatattgcta

>406LG3

agcgtgtaagggaaaggtttgcaaaaatttaatagat----aataagacaaatcagtcaa

gtaaaagtaaaaaatgcatagttttccttctgaaattaaagtcccaaattttgagaacga

actagaaaaat------------gttgacagataggtccactttcaattattaattttac

gaaaa-ttttttcaggacgaacctccataaaaagattcttaaaaccatgtttctaataaa

ataaaccagtggctctgaacaggtgggttattaaaatttctgagaaaagtacgcaaaaag

tgtataagggtttttaactcgtctgatcgtgacaaatttacatacgaaatattgtta

>260LG3

------------------------------------------------------------

------------------------------------------------------------

----------------------cattgacagataggtccactttcaatgattaattttac

aaaaaagtttttcaggacgaacctcc----aaaaattctcaaaatcattttgtcaataaa

ataagccagtggctctcaacagatggggtaataaaatttccgaagaaaatacgg------

-gtataagggtttttaactcgtctgaccaggacaaattcatatacgaaatattgcca

>26LG3

agcgtattagggaatggtttgcaaaaa--tagtacatagagaataagacaaatcgattaa

gcaaaagtaaaaaatacatagtttttcttctgaaattaaagccccaaattttgagaacga

actggaaaaatcgtaaataatataatgacaattaggtccactttcaattattaattttac

aaaaaattt-ttgagatc--------agaaaaaaattcttaaaatcattttgcgaataaa

gtaagccagtgactctcaacaggtgggttaataaaatttctgaa----gtacgggaaaaa

tgtataaaagtttttaactcgtctgctcttgacaaattcacatacgaaatattgcta

>90LG4

agcgtgttagggaatggtttgcaaaaatttagtaaatagagaataaaacaaatagactaa

gcaaaagtaaaaaatgcatagttttccttctgaaattaaagtcacaaattttgagaacaa

actagaaaaatcaaaaataatatatcgacaggtaggtcctctttcaatgattaattttac

aaaaaatttgttcaagac-------------aaaattcttaaaatcattttgctaataaa

aaaagctaattactctcaacaggtgggctaataaaatctca-------------aaaaaa

tgtataagggtttttaactcgtctgatcatgacaaattcacatacgaattattgcta

>412LG5

agcgcgttagggaatggtttgcaaaaatttag----taaagactaaaacaaagcgattaa

gtaaaaataaaaaatgcattgtttttcttctgaaattaaagtcccaagttttgagaacga

actgaaaaaatcgtaaaaaaaaaattgccaggtaggttcactttcgactattcattttac

aaaaaatga---------------------------------------------------

------------------------------------------------------------

--tataaaagtttttaactcgtctgatcatgacaaattcacatatgaagtagtgcta

>52LG2

agggtgttagaaagttttttgcaaaaattt-gttgatagagaataagacaaatcgcttga

gcaaaagttaaaaatacatagttttccctctaacattaaagttccaaattttgagaacga

actaaaaaaatcaaaaataatatattggcggataggtccagtttcaattataaaatttcc

a-aaaattttttcagcacgaacctccataaacaaattctcaaaaccattttgctaataaa

ataagccagtggctctcaacaggtgggttaa-aaaaattctgaagaaagtacgcaaaaaa

tgtaaaaagg-ttttaactcgtctgattctgacaaattcgcatacgaaatattgtaa

>210LG3

----------------tttta----------gtagatagagaataagacaaatcaattaa

acaaaggtcaaatatgcatagttttcc----------------ccaaattttgaggacga

agtagaaaaatcgtaattaatatattgacaggtaggtccattttcatttattaattttac

aaaaaagtttttctggatgaatctccatgaaaaaattttcaaaaccactttgctaataaa

ataagtcagtggctctcaacaggtgggttaa--aaaattctgaaaaaagtacgcgaaaaa

tgtataaaggtttttaa----------------------------------------

>243LG8

------------------------------------------------------------

------------tatgtattgttttccttctgagattaaagccccaaattttgagaacga

aataaaaaaatcgtaaataatatattggcaggtaggctcacattcaattatttattttac

g-aaaactttttcaggacgaaccttcctaaaaaaattctcaacaccactttgataataaa

ataagccagtggctctcaacaggtgggt-----gagtttctaaataaagtacgcgaaaaa

tgtataagggtttttaactcgtctgatcatgaca-----------------------

>202LG10

---------------------------------------------------atccattaa

gcaaaagtcaaaaatgcataattttccttctgaaattgaagtcccaaattttgagaacga

agtagaaa---gcgtaatactatattgac-----ggtccactttcaattattaattttag

taaaaaaaacgtcagcacgaacctccagggaaaactttttaaaactattttgtttataaa

gtaagtcagtggctctcaacaggtgggttaataaaatttccgaagaaaatacgcgaaaaa

tgtataaggc-ttttaactcgtctgatcatgacaaattcacataagaaataa-----

>203LG10

------------------------------------------------------------

--------aaaaaatgcatagtttt-cttctgaaattgaagtcccaaattttgagaacga

actagaaa---cgtaaataatgtatttacaggcggatccactttaaattattaattttag

t-----aaacttcaggacgaacctcc----aaaacttgttaaaactattttgtttataaa

gtaagtcagtggctctcaacaggtgggttaataaaatttccgaaaaaaatacgcgaaaaa

tgtataagt------------------------------------------------

>100LG10

--------------------------------------------------aatcgattaa

gtaaaagtaaaaaatgcatagttttccttctgaaattaaagtcccaaattttgagaacga

actagaaaaatcgtaaataatatatcgacaggtaggtccacattcaattattaattttac

gaaaaatttgttcaacacata-----atgaaaaaattctcaaaaccattttgctaataaa

ataaaccagtggctctcaacaggtggat---taaaatttctgaagaaagtacgcgaaaaa

tgtataagagtttttatttcgtctgatcac-----------aaacgaaatattgcta

>380LG3

-----------------------------------------------tcaaatcgattaa

gcaaaggtaaaaaatatatagttttccttctgaaattaaagtctcaaattttgagaacga

actagaaaaatcataaataatatactgacaggtagttccactttcaattattaattgtac

aaaaaattttttcag----cacctacgtgaaaaaattct-aaaaccattttgcaaataaa

ataagccagtggctctcaacagttagtttaat-atttttttcaacaaagaacgcgaaaaa

tgtataagggttttttactcgtctgatcatgacaaattcacatacgaaatattgcca

>144LG3

------------------------------------------------------------

-----------taatgcataggttttcttctgaaatt--agtgtgaaattttgagaacga

actttaaaaatcataaacgatacactgacaggtgggttcacattcaattattaatcttac

aaacaatttttttaggataaaccttcatgaaataattctcaaaaccattttgcgaataaa

ataagccagtggctctcaacaggtgggttaataaagttttagaagaaagtacgcgggaaa

tatataagggtttttaactcgtctgatcatgacaaattcacatacgaaatattgcta

>70LG4

agtgtattagtgaatgatttgcaaaaatttagtagatagagaataacacaaatcgattaa

gcaaaactaaaaaatgcatagttttccatcggaaattagagctctaaattttgagaacga

actagaaaaatcgtaaataatatattgacaggtaggacctttttgaattattaattttac

gaatt-ttttttcaggacgaacctccatgaaaaaattctcaaaaccattttgcaagtata

attaaccagtggttctcaacaggtgggttaataaagttttagaagaaaggacacgaaaaa

tgtataaaggtttttaattcgtctgatcatgacaaattcacttacgaaatattaatg

>255LG3

agcgtgttagggaaaggtttgcaaaaatttagtagatagagaataaaacaaattgattaa

acaaaaataaaaaaatcgt---------tctgaaattaaagttcaaaattttgagaacga

attggaaaaatcgtaaataatatattgacagg-------ttttttaattattactttaaa

aaaacattttttcaagacgaacctccatgaaaaatctttcatcactattttgctaataaa

acaagccagaggttctcaacaggtgggttaataaaatttctgaagaaagtacgcaaaaaa

tgtataaaggtttttaactt-------------------------------------

>99LG8

-----tttagggaatggtttgtaaaaatttagtagatagagaat----caaatcgattag

gcaaaagtaaaaaatccatagttctccttccgaaattaaagtcccaaactttgagaacga

actagtaaaatggtaaataatatattgacaggtgggtccactttcaattattaattttac

gaaaaattttttcaagacgaacctctttgaaaaataattcaaaactattttgctagtaaa

ataaga---------------------ttaataaaatttctgaagaaagtacgcgaaaaa

tttataagggtttttaactcgtctgatcatgacaaacttacaaacgaaatattgcta

>322LG3

----------tgaatgctttgcaaaaatttagtacttggagaataagacaaaccgattat

ccaaaagtaaaaaatgcagagttttccttcagaaattaaagtcccaatttttgagaacga

actaagaaaatcgtaaataatatattgtc-----------ct------tattagttttac

gagaaattttttctggacgaacctccattaaaaaattctcaaaaccattttgcttctaaa

ataagcca-tggttctcaacaggtgggttaataaaatttctgaagaaagttcatgaaaaa

tgtataagagtttttaactcgtctgaccatgacaaattcacatacgaaatattgcta

>107LG10

---------nggaatggtttgcaaaaatttagtaggtagagaataagacaaatcaattaa

gcaaaagtaaaaaatgcatagttttctttctgaaattaaagtcccaaattttgagaacga

actagaaaaatcgtaaataatatattgacaga--ggtccactttcaattattaattttac

gaaaaatttttttgggac----------gaaaaaattctcaaaaccattttgctaataaa

acaagccagtggctctcaacaagtggtttaataaaatttctgaaaaaagtacacgaaaaa

tgtataagggtttttaactcgtctgatc-----------------------------

>77LG3

agcatgttaaagaatggtttgcaaaaatttagtaaataaagaataagacaaatcgattaa

gcaaaagtaaaaaatgcatagttttccttctgaaattaaagtcccacattttggaaacga

actagaaaaatcgtaaatataatattgacacataggtccactttaaa-tattaaatttac

gaaaaattgtttcaggacgaacttttatgaaaacattctttaaaccacttcgctaataaa

ataagccagtggctttcaatagttgggtt-ataaaatgtctgaagaaagtacacgaaaaa

tgtataaagttttttaactcgtctgatcatggcaaattcacatacgaaatattgcta

>354LG7

agcgtgttagggtatggtttgcaaaattttagtatagaaagaga--------------ta

gcaaaagtaaaaaatgcatagattttcttttgaaattaaagtcctaaattttgagaacaa

actagaaaaatcgtaaataatatattgacaggtatgatccacttcaattattaatattac

gaaaaattttttcaggacgaatcttcatgaaaaaattcttagaac-----tgctaataaa

ataaaccagtggctctcaacaggtgggttaataaaatttctgaagaaagtacgcgaaata

tgtataaagggttttaactcgtctga------caaattcacatacggattattgcta

>40LG10

agcgtgttagggaatgttttgcaaaaatttagtagatagagaataagacaaatcaatt-a

gcaaaagtaaaaaatacacacttttttttctgaaattaaagtcccaaattttgagaacgc

actacaaaaatcctaaataaaataatgacaggtaggttcactttcaattattaattttac

-aaaaattttttcaggacgaacctccatataaaaattttcaaaactattttgctaataaa

ataagccagtggctctcaacaggtggggtaataaaatttctcaagaaagtacgcgaaaaa

tgtataaa-------------------------------------------------

>453LG10

agtgtcctagggaatggtttacaaaaatttagtaaatagagaataagacaaatcgattaa

gcaa--gttaaaaatgcaaa----tccttctgtaattaaagtcccaaattttaagaacga

actagaaaaatattaaataatatattgacagataggtccactttcaatgattaattt---

------------------------------------------------------------

------------------------------------------------------------

---------------------------------------------------------

>73LG3

agcgtgttaaggaatggcttgaaaaaatttaatagatacagaataagacaaatcgattaa

tcaaaagtaaataatgcataggtttccttctgaaattaaagtcccaaattttgagaacga

actaaaaaaatcgtaaataatatattgacaggtaggtccactttcaattattaattttac

gaaaa-agttttca-gacgggcctccatgaaaaaattctcaaaacctttttgctaataaa

agaaac---------tatataggt-ggtaaatgaaatttctgaagaaagtacgcgaaaaa

tatataagggtttttaactcgtctgatcatgacgaaa--------------------

>156LG10

------ttagtgaatggtttgcaaaaatttagtaaatagaaaataagacaaatcgattat

gcaaaagtaaaaaatgc--agttttttttctaaaattaaagttccaaattttgagaacga

actagaaaaatcgtaaataatatattgacaggtaggtccactttcatttattaattttat

gaaaaattttttcaggacgaacctccaagaaaaaattctcaaaacctttttattaataaa

ataagccagtggctctcaacaggt------------------------------------

---------------------------------------------------------

>391LG8

----------------------aaaagattagtagatagagaataagacaaatcgattaa

gc-aaaataaaaaatgcatagttctcc-------------gtcccaaattttgagaacga

actagaaaaatcgcaaataatatattgacaagtaggttcactttcaattattaattttat

gaaag-ttttttcaggacggacctccataaaagaatt-----------------------

-------------tctcaac----------------------------------------

---------------------------------------------------------

>401LG8

----------------attagcaaatattcagtaaacagagaataaaacaaatcgattaa

gcaaaagtaaaaaatgcatagttttccttttgaaattaaagtcccaaattttgagaacga

actataaaaatcgtaaataatatattggcaggtaagtccacttttaattattaattttaa

gaaaa-ttttttcaagatgaacctatataaaaaaattctcaaaa------tactaataaa

ataagtgagtggctctcaacaggtgggttaataaaatttctgaa-aaattgcgcgaaaaa

tg-tcttgagtttttaactcgtctgatcatgacaaattcacatacgaaatatggcta

>197aLG5

agcgtgttagagaatggtttgcaaaaatttagtagatagagaataagacaaatcgattaa

gcaaaagtaaaaaatgca-gttttcccttctgaaattaaagtttcaaattttgagaagaa

actagatatatattatattaaatattgacaggtagatccactttcaattattaattttat

aaaaa-------------------------------------------------------

------------------------------------------------------------

------------------------------aacaa----------------------

>149LG4

agcgtgttaggcaaaggtttgcaaaaatttagtagataaagaataagataagtcgattaa

gcaaaagtaaaaaatgcatagttttctttctgaaattaaagtcctaaattttgagcacga

actagaaaaatcgtaaataatatattgacaagtaggtccactttcagttactaattttaa

gaaaattgtattcaggactaaccaccataaaaaaattctcaaaatcactttgctaataaa

acaag-------------------------------------------------caaaat

ggtctga------------------------acaa----------------------

>268LG5

agcgtgttagggaatggtttgcaaaaatttagtagatagagaataagataaatcgattaa

gcaaaagtaaaaaaggcatagtttgccttctgaaattaaagtcccaaattttgagaacga

actagaaaaatcgtaaataatat-ttgacaggtaggtccactttcaaatattaatattac

aaaaaattttttcaagacgaacctct----------------------------------

------------------------------------------------------------

---------------------------------------------------------

>204LG4

agcgtgttaggaaatagtttgcaaaaatttagcaaatagagaa-aagacaaatcgattaa

gaaaaagtaaaaaatgaatagttttcgttctgaaattaaagtcccaaatttttagaacaa

actagagaaatcgtaaataaaatattgacaggtaggtccactttcaattaataattttac

aaaaaatgttttcaggataaacctccatgaaaaa-atctcaaaaccattttgctcaaaaa

------tcatatccatcaaccgatgggttgacaaaatttct-aagaaagtatgcaaaaaa

tgtgtaagagtttttaactcgtctgt-------------------------------

>266LG10

agcgtgttagggaatggtttgcaaaaatttactaggtaaagaataagacaaatcgattaa

gcaaaagtaaaaaatgcatagtttttcttttgcaattaaagtcccaagttttgagaacga

cctagaaaaatcgtaaaaaatatattgacaggtacgcccactttcaattaataattttac

aaaaaattttttcaggacgaatcttcatgaaaaaa-----aaaaccattt----------

------------------------------------------------------------

---------------------------------------------------------

>368LG9

----agttagggaagggtttgcaaaaatttcgcaaataaagaataaaacaaatcgattaa

gcaaaagtaaaaattgcatag-ttttcttctgaaattgaagtccca-------------a

actagaaaaatcgtaaataatatattgacaggtaggtctaatttcaattattaatgttaa

gaaaaa--ttatcaggacgaacctcc--acaaaaattctcaaaacctttttgctaataaa

a-----------------------------------------------------------

---------------------------------------------------------

>290LG3

agcgtgttaaggaatagtttgcaaaaatttaatagatagggaataagacaaatcgattaa

gtaa------aaaatgcatagttttccttttgaaattaatgccccaaattttgagaacga

actagaaaaaacgtaaataatatattgacaagtaggttcactgttaattatt-attttac

aaaaaattttttcaggacgaacctttataaaaaaattctcaaaat---------------

------------------------------------------------------------

---------------------------------------------------------

>124LG10

agcgtgttagagaatactttgcaaaaatttagtagatagagaataagacaaatcgattaa

ctaaaagtcaaaattgcatagttttccttctgtaattaaggtcccacattttgagaacga

aataagaaaatcgtaaataatatattgacaaataggtccaatttcaatgattaatttttc

aaaatattttttcagaaca---ctccataaaaaaattctcaagaccattgttctaataaa

atgtaccagtggttctcaacaggtgggttaataaaatttctgaaaaatgtaggtgaaaaa

tatgtaaaggttttta-----------------------------------------

>125LG6

agcgtgttaggaaatgttctgcaaaaatttagtagatagagaataaaacaaatcgtttaa

gtaaaagtaaaaaatgcatagttttcgttctgaaattaaagtcccaaattttgagaacga

actagaaaagtcgtaaataatatattgacaggtaggtacactttcaattattaattttcc

aaaaccttttttcaggacagacctccataacaaaattttcaaaactatttttctaataaa

ataaaccagtggctctcaacaggtgggttaataaaatttctgaaaaaagtacgcgaaaaa

---------------------------------------------------------

>180LG8

agcgtattagggaatggtttgcaaaaattaagtaaatagagaataagataaattgattaa

gaaaaagtaaagaatgcatatttttcattctgaaattaaagtcccaaaccttgagaacga

actagaaaaatcgtaaatagtatattgacaagaatatttaatattaattaaatattttaa

gaaaaattttttcaagacaaacctccatgaaaaaaatcacaaaaccattttgctaataaa

ataagccactggctctctccatgtgagttaataaaatttctaaaa-aagtatgcgaaaaa

tgtac-aagggttttaacacgtctgatcatgacaaattcacatacgaaata---aat

>319LG3

agcgtgttagggaatggtttgcaaaaatttaatagatacagaataagacaaatcgattaa

acaaaagtaaaaaatgcatagttttccttctgaaatttaagtcccaaattttgagaacga

actataaaaatcgtaaacagtattttgacaggtagctccactttcaactattaattttac

g------------------aacctccatgataaaattctcaaaagcattttgctaataaa

at-gaccagtgactctctgtaggtgggttaataaaatttttgaagaaagtacgcgaataa

tgtatgagggtttttaattcgtgtgatcatgacaaattcacatacgaaatattgcta

>346LG7

agcgtgttagggaataatttgcaaaaacttagtagatagagaataggacaaatcgattaa

gcaaaagtaaaaaatgcatagttttccttctgaaattaaagtcccatattttgaaaacga

actagaaaaatcgtaaatattatattaaa-------------------tataaattttac

gacaaatttttttaggacgaacctccataaaaaaattctcaaaataattttgtcaatgaa

attagccagtggctcttaacaggtgggttaacaaaatttttgaag-aagtacgagaaaaa

tgta--aaggttttaaactcctctgatcatgacaaatttaaatacgaaatattgcta

>435LG9

agcgtgttaggaaatggttagcaaaaa-ttagtaattagagaatacgacaaatcgattaa

gcaaaagtaaaaaatacaaagttttccttttgaaattaaagtctcaaattttgagaacga

actaggaaaatcgtaaataaaatattga--------------------------------

------------caaaaccgaacttc-----aaaattctcaaaaccattttgctaataaa

atatggcagtggctctcaacaggtgggttaacaaaaa-----------------------

---------------------------------------------------------

>288LG3

agcatgttaggaaatggcttgcaaaaatttagtagatcgagaataaaacaaatcgattaa

gccaaaagaaaaaatgtatagttttcc-----------------caaattttgagaacga

actagaaaaatcgtaaattatatattgacaggtaggtccactttcaactagtaattttac

aaaaca-tttttca----ggacctccatgaaaaaattttcaaaaccattttgctaataaa

ataagccagtggctctcaataggtagattaataacatttttgaaaaaagtacgcgaaaaa

tatata---------------------------------------------------

>113LG8

agcatgttagggaatggtttgcaaaaatttagcaaatagagaataagacaaatcgattaa

gcaaaagtaaa-aaaatttttttttccttt-----------tgaacgattttgagaacga

actagcaaaat-----taattatattgacaggtaggtcc---ttcaattattaattttac

gaaaaattgtttcaggacgaacctctatgaaaaaattctcaaaaccattttgctaataaa

aaatgacagtggctctcaacaggtggattaataaaatttctgaagaaagtacgcgaaaat

tgtaaaagggtttttatctcgtctaatcatgacaaattcacatgcgaaataa-----

>256LG4

agcgtgttagggaatggtttgcaaaaatttagcaaatagagaataagacaaaccgattaa

gcaaaagtaaaaaatgcataattttcccataattttcaaagttccaaattttgagaacga

actagaaaaa----------tatattgacaggtgggttcactttcaattattaattttac

gaaaaattttttcaggacaaactcccatgataaaattctcaaaacaattatgctgataaa

ataagccagtggctctcaaccgataggttaacaaaattactgaagaaagtacgcgaaaaa

tgtataagggtttttaatttgtctgattctgacaaattcacatacgaattattgcta

>201LG3

-------cagggaatggtttgcaaatcttaaatgtatagagaataaaacaaatcgattta

gcaaaagtaaaaaattcatagttttccttc--------------caaattttgggaacga

actagaaaaatcgcaaataataaattgacagataggttcggttttaattattgattttat

gaaaaattttttcaagacgacccttc-----aaatttctcaaaaccattttgctaataaa

ataagccagtggctctcaacagatgggttaataaatttcttgaagaaagtacgcgaaaaa

tgtatatgagtttttaactcgtctgatcataacaaattcacatacgaaatattgcta

>44LG10

agcgtgttaggtaatggtttgcaaaaatttagtagatagagaataaaacaaatcgattaa

gcaaatttaaaaaatacctagtttttcttctgaaattaaagt-caaaattttgagaacga

actagaaaagtcgtaaataatatattgacaagtaggtctactttcagttattaattttac

gaaaattttttt-------aacctccgaataaaaattctcaaaatcgttttgctgataaa

ataagccagtggctctcaacaggtgggttaattaaatttctgaaaaaagtacgc-gaaaa

tatttaagggattttaattcctctgatcatgataaattcgcatacgaaatattgcta

>85LG5

agcgtgttaggggatggttttcaaaaatttagtagatacaaaataaaat-aatcgattaa

gcaaaagtaaaaaatgcatagttttccttctggaattaaagtcccaaattttgagaacga

actataaaaatcttaaataatatattgacaggtagttccactttcaattattaattttat

aaaaaa-tttttcaggacgaacccca-----aaaatttccaaattcattttgctaataaa

ataaaccagtggctctcaacaggtgggttaataaaaattctgaagaaagtacgcgaaaaa

tgtataagagtttttaagtcatctgattataacaaattcacagacgaaatattgcta

>154LG8

agcgtgttagggaatggtttgcaaaagtttagtagatcgagaaaaagacaaatcgattaa

gcaaaactaaataatgcatagttttccttctgaaattcaagtcccaaattttgagaacg-

actagtaaaatcataaataatatgttgacagataggtctattttcaattatgaattttaa

gaaaat---ttttgg--t-------------aaaattctcaaaagcgttttgttaataaa

ataagccagtggctctcaacaggtgggttaatgaaatttctgaagaatgtacgcgaaata

tgtataagtgtttttaactcgtcttatcataacaaatttacata-------------

>310LG2

agcgtgttaaggaatgtattgaaaaaatttagtagatagggaataagacaaatcgattaa

gcaaaagtaaaaaatgcatagttttacttttgagattaaagtctcaaattttgagaacga

actagaaaaatcgtaaataatatattaacaggtagattcactttcaaataatagttttac

aaaaacaaattttg-----------------aaaattttcaaaaccattttgctaattca

ataagccagtggctctcaacaggtgggttaataaaatttctgaagaaagcacgcgaaaaa

tgtatgagggtttttaacttgtctgatcatgacaaattcacatgcgaaatattccta

>97LG3

agcgtgttagggaatggtttgca-aaatttagccgatagagaatag--------------

gcaaaagtaaaaaatgcatagttttccttctaaaattacagttccaaattttgagaacga

aattaaaaaatcataaataatatattgacagataggtccactttcaattatcaattttac

aaaaaccattttaaagacgaacctccaggaaaaaattcttaggactatttcgccaacgaa

ataagccagtggctctcaacaggtgggttaataaacgttctgaagaaagtacgctaaaaa

tgtat--gagtttttaattcgcctgatcctgacaaattcacatacgaaatattgctt

>188LG3

agcgtgctagggaatggtttgcacaaatttagtaggtagagaatagcacaaagcgattta

gcaaaagtaaaaaatgcatagttttcc----------atcgtcccaaattttgagaacga

actggaaaaatcgtaaataatatattgac--ataggtccactttcaattattaatttttc

aaaaaac-----cagaacgaacttccaagaaaaaattctcaaaaccatttggttaataaa

ataagctagtggctttcaaccggtgggttaaaaaa--ttctgaagaaaatatgcgaaaaa

tgtataaaagtctttagctcgtcttattataacaaattcgca---------------

>86LG3

agcgtgttagggaatggtttgcaaaaatttagtagatagagaataaaacaaatcgattaa

gcaaaagtaaaaaatacatagt-----ttctgaaattaaagccccaaattttgagaacga

actagaaaaatcgtaaataataaattgacaggtaggtccactttcaattattaattttac

-aaaaaaaatctcaggacgaaccttcatgaaaaaattctcaaaactattttgccaataaa

ataagccagtggttctcaacaggtg-------a-aatttctgaaaaaagtacgcggaaaa

tgtataaaggtttttaactcgtctgatcatgacaaattcacatacaaaatattgcta

>339LG9

agcgttttagggaatggtttacaaaaatttagtagatagagaataaaaaaaatcgattaa

gcaaaagtaaaaaatgcatagttttccttctgaaataaaagccccaaattttgagaacga

aaaaggaaaatcgtaaataatacatatacagg--ggtccactttcaattattaattttac

aaaaaaatttttcaggacgaacct-------aaaattttcgaatccaact--ccaaaaaa

gtaagccagtggctctcaacaggcgggttaata-ttttttttaagaaagtacgc------

---------------aactcgtctgattatgacaaattcacatacgaactattgcta

>118LG10

agcgtgttagggaatggtttgcaaaa------------------aagacaaatcaattaa

gcaaaagtaaaaaatgcatacttttcctcctaaaattaaagtccaaaattttgagaacga

actagaaaaatcgtaaataatatattgacaggtacgtccactttcaattattaatttttc

gaaaaatttttt------aaatttttatgaaaaatttctca----cattttgctaataaa

atatgccagtggttctcaacaggtgggttaataaaatttctgaagaaagtatgcgaaaaa

tatataagggtttttaactcatttgatcatgacaaattcacacacgaaatattgcta

>120LG10

aacgtgttaagaaatggtttgcaaaagtttagt----agagaatacgacaaatctattat

gtaaaagtaagaaatttatagtttttctactgaaataaagcccccaaattttaagaacga

actagaaaaatcgtaaataatatattaacagata--tccactttcaattactaatttttg

aaaaaatttttt------gaacctccataagaaaattttcaaaactaatttgctaattaa

ataagccagtggttctcaacaaataggttaattaaatttttgaaaaaagtagaagaaaaa

tgtataacggtttttaattcgttttatcataaaaaattcacatacgaattattgcta

>249LG9

------------------------------------------------------------

------------------------------------taaagtcccaaattttgagaacca

actagaacaactgtaaataatatattgagaggtgggtccact------tattaattttac

gaaaaatttttt------aaacctctatgaaaaaatcctcaaaaccattttgctaat---

aaaagccagtggttcttaatgggtggattaataaaatttctgaagaaagtacgcgaaaat

tgtataagggtttttaacttgtctgatcatgaaaaattcacattcaaaatattgcta

>145LG3

agcgtgacaggaaatggttc--aaaaatttagtagatagataataagacaaatcgattaa

gcaaaagtaaa-------tagttaccccaaaaaaattaaagtctaaaattttgagaacga

actagaaaaatcctaaataatatattgacaggtaggtccacttccacttgttaatttaac

gaaaaaaaatttcaatacaaaccttcataa-aaaattctcaaaactagtaagccaataaa

ataaggcagtggctctcaacagctgtgctaa----------tgtgaaattacacgaaaaa

tgtataagagtttttaactcgtctgatgatgacaaattcacatacgaaatattgcta

>165LG2

-----attaaggaatggtttgcaaaaatttaataatttaaca-----acaaatcaattaa

gcaaaagtaaa-aatgcataattttcctc--------------gcaaattttgagaacgg

acta-aaaaatcgtaaataatatattgctatataggtccactttcaattattaattattc

-aaaaaatttttcaggagcaacctgcatgaaaaaattctcaaa-atattttgccaataaa

ataaaccagtaactctcaataagtgggttaataaaatttctgatgagagtacgtgaaaaa

tgtataagggtttttaactcgtctgatcataacaaattcacatatgaaatattgata

>308LG7

------------------------------------------------------------

-------------------------------------------gcaaattttgagaacga

acttgaaaaatcgttaataacatattgacaggtaggtccactttcaattgttaattttac

gaaaaatttttt---tacgaacgttcatgat--------------ctttttgctaacaaa

ata----agtggctctcaacaggtgggttaataaaatttcttattaaagtacgcaaaaaa

tgtataagggtttttaacttgtctgaccaggaaaaattcacatacgaaatattgcta

>146LG2

--------------------------------------------aaagcaaatcgattaa

gaaaaactaaaacatatatagttctccttctaaaataaaagtccaaaattttgagaacga

actagaaaaatcgtaaataatatattgccaggtaggtccactttcaattattaatgttac

aaaaac-tttttcaggacgaacctccataaaaaaattctcaaaaccattttgctaataaa

ataagccagtggccctcaacagatggattaacaaaatttctgaagaaagtacgc---ata

tgtataaaggtttttaattcctctgatcgtgacaaattcacatacgaaatattgcta

>181LG2

------------------------------------------------------------

------------------------------------aaaagtccaaaattttgagaacga

actagaaaaatcgtaaataatatattactaggtaggtccactttcaattattaattttac

aaaaac-tttttcaggacgaacctccataaaaaaattctcaataccattttgctaataaa

ttaagccagtggctctcaacagatggattaacaaaatttctgaagaaagtacgcggaaaa

tgtataagggtttttaattcctctgatcatgacaaattcacatacgaaatattgcta

>197bLG5

------------------------------------------------------------

--------------------------------------aattctcaaatttttaaaacga

actagaaaaatagtaaataatatattaacaaatagatccactttcaaatcttaattttac

gaaaaattttttcaggacgatcct--atgaaaaaattcttaaaaccattttgctaataaa

ataagccagtagctcttagcaggtgtgttaataaaatttctgaaaaacgtacgcgaaaaa

tatacaagggttattaactcctctgatcacgacaaattcaaatacaaaatattgata

>289LG4

------------------------------------------------------------

--------------------------------------------gaaattttgagaacga

actaggaaaatcgaaaataatatattgac-----ggtctactttcaattattaattttac

aaaaaattttttcaggacgaaccttcatgaaaaaattttcaaacccgttttgttaataaa

atatgacagtggctctcaacatgtgggttaataaaatttctgaagaaagtacgcg-aaaa

tgtagaggggtttttaactcgtctgatcatgaca-----------------------

>309LG8

------------------------------------------------------------

------------------------------------taaaggcaaaaattttgagaacga

actagaaaaatcgt-aatattatattgac---gaggtccactttcaattacgaatttt--

taaaaatttttgctggacgaacctgcgtgaaaaaatttttaaacccatcttgttaataaa

ataagccagtggttctcaacaggtgggttaataaaatttctggagaaagtacgcgaaaaa

tttgtaagggttgttaag---------------------------------------

>240LG3

agcatgatagggaatggtttgtataaatttagtagatagagaataagacaaatcaa----

-----------aaatgcattgttttccttc-------------ccaagttttgagaacga

actagtaaaatcgtaaataataaattgacaggtaggcccactttcaattattaatcttac

gaaaaattttttcacgacgaacctccttgaaaaaatt-taaaaatcattttgctaataaa

ataagacagtggctctcaacaggtaaataaacaaaatttttgaagaaagtacgctaaaaa

tgtttaaggatttttaactcgtttcattatgacaaattcacaaacgaaatattgtta

>221LG10

-------------------------------atagataaaaaataaaacaaatcgattaa

aaaaaagtaaaaaatggttagttttccttctgaaattaaagtcccaaattttgagaacgg

tctaga-----------aaattttttgac---taggtcctctttaaattattgatttaac

gaaaaa-----tcaggacgaatctccataaaaaaattctcaaaaccaatttgctaataaa

ataagccagtggctctcaacaggtgggttaataaaatttctgaaaaaaatacgcgaaaaa

tgtataag--tttttaactcgtctgttcatcacagatttacatacaaaatattgcta

>287LG10

------------------------------------------------------------

------------------------------------------------------------

----------------------------------tgtctact----attattaattttac

aaaaaaatttttttggacgaacctctatgaaaaaattctcaaaacc--tttgctaataag

ataagacagtggctctcaacaggtgggttaataaaatttctgaaaaaagtacgtgaaaaa

tatatatgggtttttaactcgtctgatcatgaaaaatttacatccgacatattgcta

>31LG10

agcgtgttagggaatggttttcaaaaatttagtaaatagagaataagacaaatccattaa

gtaaaagtaaaaaatgcatacttttccctctgaaattaaagtcccagattttgagaacga

actagaaaaatcgtaaataatttattgacaattaggtctacttttaattattaattttac

aaaaaattttttcaggacgaacctccaagaaaaaattctcaaaacc-ttttgctaataaa

ataagccagtggctctcaacaggtgggttcataaaatttctgaagaaagaacgtgaaaaa

tgtataagggtttttaactcgtttgatcatgacaaagtcacatacgaaatattgcta

>222LG3

agcgtgttagggaatagtttgcaaaaatatagtagatagagaataagac-----------

------gttaaaaatgcatagttttccttctgaaattaaagtcccaaattttgagaacga

actagtaaattcgtaaagaacatattgacaggtaggtctactttcaattattaattttac

aaaaaattttttctggacgaacctctacgaaaaaattctcgaaaccattt----------

------tagtggctctcaataggtgggttaatacaatttcggaagaaagtacgcgaaaaa

tgt-----ggtttttatatcgtctgatgatgacaaatttacatactaaatattgcta

>81LG2

agcgtgttaggaaatggtttgcaaaaatttagtagatagaaaataaaacaaatcgattaa

tcaaaactaaaaaatgcatagttttccttctgaaattaaagtcccaaactttgagaacga

accagaaaaacaataaatgatatattgtcaggtaggtccactttcaattattaattttac

aaaaaaatttttcaggacaaacccccataaaaaaattcttaaaaccattt---tactaaa

------cagtggtcctcaataagtagattaagaaaatttctgaaaaatgtacgcaaaaaa

tatataaaggttcttaactcgtcttatcatgacaaattcgcataggaaatattacta

>157LG9

agcgtgttagggaacggtttgcaaaaatttaatagatagagaataagacaaatcgattaa

acaaaaataa-----------ttttccttctgaaattaaagtcccaaattttgagaacaa

actagaaaaatcgtcaataatatattaacggataaggttgttttcaattattaattttaa

gaaaaaattttt---tacgaaccatcatgaaaaaattctgaaaactattttgataataaa

gtaagtcagtggctttcaacagatggattaataacatttctcaagaaagtacgc-gaaaa

tgtataagggtttttaactcgtctgatcatgacaaatttacatacgaaatactgctg

>257LG7

agcgtattagggaatggtttgcaaaaatttagtagataaagaataagacaaatcgattaa

taaattgtaaa----------------tcctgaaattaaaatcccaaattttgggaacga

actagcaaaaacgtaaataatatattgtcaggcaagtccact------tattaattttac

gaaaaattttttcagtccgaacctccataaaaaaattttcaaaagtactttgctaataaa

ataagccagtggctctcaacagatggattaata------------aaattacgcgacaaa

tgtataagggtttttaactcgtctcatcatgacgaaa--------------------

>307LG8

-----------------tttgcaacaatttagtagatagagaataagacaaatcgattaa

gcaaaagtaaaaaat------ttttcctttt------------tcaaattttgagaatga

actaaaaaaatcgtaaaaaatatattgaccggtaggttcatattcagtta-taattttac

gaa---tattttcaagacgaacctccaggaaaaaattatgaaaactttt-cactaataaa

ataaggcagtggctctcaac----------------------------------------

---------------------------------------------------------

>143LG3

agagtgttggggaatggtttgcaaaaatttagtagatggagaataagacaaaccgattaa

gcaaaattaaaaaatgcaaagtttttcttctgaaattaatgttccaaattttgagaacaa

actaaaaaaatcgtacataatatattgtcatgctggtccact------tattagttttac

gaaaaattttttctcgacgaacctctatgaaaaaattatcaaaaccattttgcttataaa

ataagccagtggttctcaacaggtgggttaataaaatgtctgaagaaagttcacgaaaaa

tgtataaaggtttttaactcgactgatct----------------------------

>365LG8

agcgtgttagagaatggtttgcaaaaa--tagtggatggagaataagacaaaccgattaa

gcaaaagtaaaaaatgcatagttttccttctgaaattaaagttctaaattttgagaacga

gctagaaaaatcataaataatatattgtctggtaagtccgct------tattaattttac

gaaaaattttttctcgac--------a----aatattttcaaaaccattgtgcttataaa

ataaac-------------cagtaaaattaataaaatgtctgaagaaaatttacgaaaaa

tgtataagattttttaactcgtctgatcatgacaaattcacatacgaaatatt----

>394LG10

agtgtgttagggaatggtttgcaaaaatttattagatagagaataagacaagtcgattaa

gcagaagtaaaaaattcataatttttc------------------------tgagaacga

actagaaaaatcgtaaataatata----------ggtccattttcagttattgattttac

gaaaaattttttcaagacgaatctgcatgaaaaaaactttaaatccattttattattaaa

atatatcagtggctctcaacaggtgggttaataaaatttttgaagaaagtacgcgaaaaa

tgtataagggtttttaactcgtctgatcatgactaa---------------------

>108LG5

agcgtgttaggatatggtttgcaaaattttagtagatagagaataagacaaacagattaa

ggaaaagaaaaaaaaatacagttttccttctgaaattaaagtcccaaattttgagaacga

actagaacaactgtaaataatatattgacaggtaggtccacttccaattcttaattttac

aaacaattttttctggacgaacctcgatgaaaaaattttcaaaactaatttcctaataag

ataagccagtggctctcaacaggtgggttaataaaatttttgaagaaagtactcgaaaaa

---------------------------------------------------------

>326LG10

agcgtgttagaa---agtttgaaaaaatttagtagatagagaataagat-----------

---aaattaaaaaatgcatagttttccttctgaaattaaaattccaaatattgagaacga

tctagaaaaatcacaaataatatattgacaggtaggtctacttgcaataattaattttta

aaaaaattttttaagaatgaacctctatg--aaaattttcaaaactattttgctaataaa

ataagtcagtggctctcaacaggtgggt--------------------------------

---------------------------------------------------------

>358LG9

agcgtgttagggaatggcttgcaaaaatttagtagatagagaataagacaaatcgattaa

gtg-----aaaaaatgca-gttttcccttctaaaattaaagtcccaaattttgagaacga

actacgaaaatcgttattaatatcctgacaggtaggtccactttcaattattcat-----

-aaaaattttttcaggacgaacctccaggaaaagattctcaaatccattt----------

------------------------------------------------------------

---------------------------------------------------------

>169LG10

agcgtgttagggaatggtttgcaaaaattta-------------aagacaaatcgattaa

gcaaaagtaaaaaatgcatagtttttcctctgaaattaaggtctcaaattttgagaacga

actagaaaaattataaataatatattgataggaagctccactttgaattattaattttac

gaaaaa-tttttcgggatgaacctgcgtgaaaaaattctcaaaactattttgctaataca

atcagccagtggctctcaacaggtgggttaataaaaaaattcaagaaaatacgcgaaaaa

tgtataatgatttttc-----------------------------------------

>186LG2

agtgtgttaggaaatagtttgcaaaaatttagtaaat------------------ttaag

gttaaaataaaaaatgcatagttttccttctgaaattaaaatcccaaattttgagaacga

actagaaaaatcggaaataatatattgacaggtaggtccactttcaattattaattttac

gaaaaattttttcaggacgagcttccatgaaaaaattctcaaaaccattttgctaataaa

aaaagccagtggctctcaacgagtgggttaacaaaatttctgaagaaattacgc------

---------------------------------------------------------

>231LG5

------------------------------------t------------------attaa

gcaaaagttaaaaatgcatagtttttcttcttaaa--aaagtcccaaattttaagaacga

acttgaaaaatcgtaaataatatattgacagataggtccactttcagttattaattttac

aaaaaatttttttagtacaaacctccaggaaaaaattctcaaaaccattttgctaaaaaa

attagccagtggctctcaac----------------------------------------

---------------------------------------------------------

>377LG3

------------------------------------------------------------

-----ggtaaaaaatgcatagttttccttatgaaattaaagtcccaaattttgagaacgg

actagaaaagtcgtaaataatatattcacaggtaggttcactttaaattattaaccttac

gaa-------atcaggacaaaactccatgaaaaaattctcaaaactattttgcta-----

------------------------------------------------------------

---------------------------------------------------------

>22bLG3

agcgtgttagggaatcatttacaaaaatttagtagatagagaacaagacaaatcgattaa

gcaaaagtaaaaaatgcatagttttctttctgaaattgaattcctaaattttgagaacga

actaa-aatatcgtaaataatatattgataggtaggtccactttcaattattaattttgc

aaaaaattttttcaggggcaccctccatgaa--------------cattttgccaataaa

ataagctagtggctcttaagaagtgagttaata-aaattctgaagcaaatacgtaaaaaa

tgtataagggtttttaacttgtctgaacttgacaaattcacttatgaaatattgcta

>361LG4

agcgtgttagggaatggtttgcaaaaattcagtagacagggaataagacaaatcgattaa

acaaaagtaaaaaatgcattgttttccttctgaaattaaagtcccaaatttttagaacga

actagaaattttattatcagaacattgacatgtaagtccactttcaacta-ttattttac

gaaaaatttttttaggacnnnnnnnnnnnnnnnnnnnnnnnnnnnnnttttcttaataaa

ataagccagtggctctcaatagataacttaataaaatttctgaagaaagtacgcgaaaaa

tgtaaaagggtttttaactcatctgatcatgacaaactcacatacgaaataatgct-

>121LG9

agcgtgttaaggaatagtttgcaaaaatttaagaatg-------aaaacgaatcgattaa

ggaaaagtaaaaaatgcatagttttccttctgaaattaaagtcccaaattttgagaacga

actagaaaaatcgtaaaaaatatagtaacaggtaggtccactatcaataatcaattttcc

-aaaaaaatttacaaaacgaacctccatgagaaaattttcaaacccactttgctaataaa

acacaccagtggctctcaacaggtgggtttgtaaactttttgaagaaagcctgcaaaaaa

tttaataaggataataatttgtctgatcaagacaaattcacatacgaagtattgtta

>167LG8

agcgtgttagggaatggtttgcaaacattttatagagaa----taaaacaaattgattaa

ataaaagcaaaaagtgcatagttttccatctgaaattaaagtcccaaattttgagagcga

actggaaaaatcgtaaataatatattgccaggtatatccatattcacttatcaattttcc

aaaaaaaatttacagaacgaacctcc-----aaaattttcaaaaccactttcctaataaa

aaaaaccagtggctctcaacagatgggttaataaaatttttgaagaaagtatgcgaaaaa

tgtataagggttattaactcttctaatcatgacaaattcacatacaaaatattgctc

>205LG4

agagtattagggaatggtctgcaaaaagttagtagattgagattgtaaagaatcgattaa

gtaaaagtaaaaaatgcatagttttccatctgaaattaaagtctcaaattttgagaacga

----gaaaaatcgtaaataatatattgacaggtaggtccata-tcaattatcaattttcc

-aaaaaaaattacagaaattagct-------acaattttcaaaatcactctgctaataaa

gtaaaccagtggctcttaacaaataaattaataaattttttgaagaaagtatgcgaaaag

tttataagggattttaactcgtctgatcatgacaaattctcatacgaaatattacca

>335LG10

agcgtgttagggaatg----gcaaaattttagtagatagagaataaaacaaatcgattaa

gcaaaagtaaaaaatgcatagttttccgtctgaaattaaagccccaaattttgagaacga

actagaaaagtcgtaaataatatattgacaggtaggcccactattaattattgattttac

-aaaaaatttttcaggat--------------aaattttcaaattcatttgactaataaa

ataagccagtggctctcaacaggtgggttaatacaatttctgaagaaagtacgcgaaaaa

tgtataagggtgtttaactcgtctgatcatgac-aatacacatacaaaatattg---

>427LG4

agcgtgttaaggaagggtttgcaaaaatttagtagatagagaataaaacgaattgattaa

gaaaaagtaaaaaatgcatagatttccatctgataatacagtctcaaattttgagaatga

aaaagaaaaatcctaaataatatattgataggtaggtccatgttcaaa------------

------------------------------------------------------------

------------------------------------------------------------

---------------------------------------------------------

>223LG3

agcgtgttagggaatggtttgcaaaaatgtagtagatagagaataatacgaatcgattaa

gtaagagtaaataatgcatagttttccatctgaaattaaagttccaaattttgagaacga

actggtaaaatcgtaaataatatattgacaggtaggtttatgttcaattatcaattttcc

aaaaaaaatttacagaacgaacctttatgagaacattgcg-----cactttgctaataaa

ataaatcagtggctctcaacaggtgggttaataacttttttgaataaagtatacgaaaag

tgtatcagtatttttaactcgcctgatcatgacaaattcacacacgaaatattgcta

>374LG2

aacgtgttagggaatggtttgcaaaaatgtagtggatagaaaataaaacgaatcgattaa

gtaaaagtaaaaaatgcataatttttcatctgacattaaagtcccaaattttgagaacga

actaaaaaaatcttaaataatatattgaggagta---ttctgttcacttattaattttcc

-aaaaaaatttactgaacgaacctccatgagacaaatttgaaaaccactttgttaataaa

------------------------------------------------------------

---------------------------------------------------------

>418LG3

---atgtcagggaatggtttgcaaaaattttgtcgatggagaataagttaaatcgattta

gcaagagtaaaaaatgcatagtttttcttttcaaattaaagtccaaaattttgagaacga

gctagaaaaatcgaaaattatatattgacacgtaggtccaattccaattattaattttgc

-aaata---------------------------------------cattt----------

------------------------------------------------------------

---------------------------------------------------------

>189LG6

accgtgttagggaaagctttgcaagaagttagtagatagagaataagacaaatcgattaa

acaaaattaaaaaatgcatagtttttcttccgaaaataaaatcccaaattttgagaacga

actagaaaaagcgtaaataatatattgacagataagtccacttgcaattattaattttag

g-----ctttttcaggacgagcctcc-----aaaattctgaaaaccattttgctaataaa

ataagccagtggctctcagcaggtgggttaaaaaattctgcgca----------gaaaaa

tgtataagggttttt-attcgtctgatcatgacaaattcacatacgaaatattgtta

>82LG5

----------------------------ttaataaatagagattaaaacaaagcgattaa

gcaaaagtaaaaaacgcatagtttttcttctgaaattaaaat-taaaattttgagaacga

actagaaaaatcgtaaataatatatcgacaggtaggtccattttcaattattaatttt--

--acgattttttcaggatgaacccccatgaaataattctcaaaac-----tgataataaa

ataagccagtcgctctcaacaggtgggttaatattttttttaaagaaag-tagcgaaaaa

tgtataagggtttttaa-tcatctgatcataaccaattcacatacgaaataatgcta

>162LG10

------------------------------------tagagaataagacaaatcgattaa

gcaaaagtaaaaaacgcatagttttctatctcaaattaaagatccaaattttgagaacga

actataaaaatcgtaaa-------tctgcaggtaggtccacttccaattattaattttac

gaaaaattttttcaagacaaacatccatgaaaaaattctcaaaatcaatatgataataaa

ataagtcagtggctctcaacaagtgggttagtattttttttta--aaagtacgtgaaaaa

tatataagggtttttaactcgtctgattatgacaaattcccatacgaaatactgtta

>104LG7

----------------------aaaaatttagtagatacagaataagacaaatcgattaa

gcaaaagtaaaaaatgcatagttttacttttgaaataaaaattccaaattttgagaacga

actagaaaaatcgtaaataatataacgacaggtaggtccattttcaat-aataattttac

gaaaaactttttcaagatgaaccttcataaaataattttcaaaactattttgctaataaa

ataagccagtggctcccaacacgtgaattaatattttttttgaaaaaagtacgcaaaaaa

tttataagaatttttaactcatctgatcatgacaaattcacatactacataatgcta

>237LG10

---------------------------------------------------aacgattaa

acaaaagtaaaaaatgcataattttccttctgaaattaaagttccaaattttgagaacga

actaaaaaaatcctaaataatatattgacagaaaaatctact----ataattaattttac

caaaaattttttcaggacgaatgtacatgaaaaaattctcaaaaccattttgctaataaa

acaagccagtggctttcaacaggtgggttaatgaaatttgtgaagaaagcacgcgaaaaa

tgtataagggtttttaactcgtgtgatca-----------catacgaaatcttgcta

>206LG10

agcgtgttaggaaatggtttagcaaaaattagtaaattgagaataagacaaatcgattaa

gcaaaagcaaaaaatgcatagttttccttttgaaattaaagtcccaaattttgagaacga

actagaaaaat---------------taaaggtacgtccactttcaaatattaattttac

taaaaattttttcaggacaaacctccatgggaaaattctcaaaactattttgttaataaa

ataagcgagtggctcccaacaggtga---aatgaaatttctgttaaaagtacgcgaaaga

agtataagagtttttaactcgtctgatcataacaaattcac---tgacttattgcta

>212LG3

---------------------------------agatagagaataagacaagtctattaa

acaaaagtaaaaaatacatagtttt--ttc--------tgaaattaaattttgggaacga

actagacaaatcgtaaataatatattgac----aggtccactttcaatta---gctttaa

taaattttttttcaggacgaatttttatgaaaaaattctcaaaaccattttgctaataaa

ataagccagtggctctcaataggtgggttaataaattttctgaagaaagaacgcgaaaaa

tatataacggtttttaactcgtctgatcac---------------------------

>208aLG3

--------------------------------------------aagacaaattaattaa

gcaaaagtacaaaatgcatagttttacttctgaaattaaagtcccaaattttgagaatga

actataaaaatcgtaaataatatattgacaagtagtcccactttcaattattaattttac

gaaaaatttttttaggacgcatgttcataaataaattttcaaaaccattt----------

------------------------------------------------------------

---------------------------------------------------------

>208bLG3

------------------------------------------------------------

---------------------ttttacttctgaaattaaagttccaaattttgagaacga

actataaaaatcgtaaataatatattgacaagtagtcccactttcaattattaattttac

gaaaaatttttttaggacgaacctccatgataaaattctcgaaaccattttgctaagaaa

ataagccagtggctctcaacatgtgggttaaaaaaatttctgaagaaagtataa------

--tacgagaacttttacctcatctgatcatgacaaattcacatacgaattattgcta

>84LG9

agcgtattagggaatgg---acaaaaatttagtagatagagaataaaacaattcgattaa

ggaaaa-----aaatgcatagtttcacttatgaaattaaagtcccaaattttgagaacga

actaaaaaaatcctaattaatatattgacaggtaggttcactttcaattattaattttac

aaaaaaaattttgaggacgaacctgcatgag--------caaaactattttgctaataaa

acaagccagtggttctcaacagatgagttaataaaatttctgcagaaagtacgctaaaaa

tgtataaaggtttttaactcgtctgatcatgacaaattcgcatacggaatattgcta

>152LG3

-------------------tgcagaaatttagtagatggagaataagacaaaacaattaa

gcaaaagtaaaaaatgtatagttttccttttaaaattaaagtctcaaattttgagaacga

actggaaaaatcgtgaataatatattgagagacaggtccactttcaattattaattttac

caaaaattttttcgggacgtaattccataaaaaaattctcaaaaccattttgccaaaaaa

ataagccagtggctctcaacaggtgagttaatataatttctgaagaaagtacac------

---------------------------------------------------------

>238LG8

-----------------------aaaatttagtagatagagaataaaacgaattgattaa

gtaaatgtaaaaaatgcatagttttccatttgcaattagagtcccaaattttgggaacga

actagaaaaattataaataatgtattgacaggaacgtccactttcaattattaatt---a

aaaaa------tcagggtggaccaccatgaaaaaaattccccaaatattttgctgataaa

acacag-----gctttcaacaggtgggttaataaaatttccgaag-aagaacacgaaaaa

ggtctaa------------------------attaacatag----gaaatattgcta

>303LG10

---------------ggtttgcaaaaatttagtaaata------caaacaaaccgattaa

gcaaaagaaaaaaatgcataatttttcttttgaaattaaagtcctaaattttgagaacga

actagaaaaatcaaaaataatatattgatagcgatgtccactttctattatttacg---g

aaaaacttttttcaggatgaacctccataaaataattctaaaaaccaatttt--------

------------------------------------------------------------

---------------------------------------------------------

>142LG6

agcgtgttagggaatggttagcaaacatttaatagataaagaataacacaaatcgattaa

gcaaaagtaaaaaattcatggtttttcttctgaaattaaagtcccaaattttgagaacga

actagaaaaatcctaaataatatattgagaggtatgttcactttcaattacaaattgtaa

gaaat-tttttacgggacgaacctccatgaaaaaattctcaaaaccatgatactactaag

agaagccagtggctctcaacaggtgggctaataaaatttctgaaaaaagtacgcgaataa

tgtataaaggtttttaattcttctgatc-----------------------------

>384LG6

agcgtcttaaagaatagtttgcaaaaatttcatagatcgagagtaagataaatcgattaa

gcaaaagtaaaaaatgcatagttttcc-------------gtaccaaattttgagaacga

actagaaaaatcgtaaataatattttga-acataggttcacttgcaa---------aaaa

aaaaa-ttttctcaggacgaacctccatgaaaaatttctcaaaaccatttgtctaataaa

ataagccagtggctctcaacaggtgggttaaaaaaattt-tggcaaaagtacgcgaaaaa

tgtataaggattttta-----------------------------------------

>280LG6

agcgtgttagggaatggtttgcaaatatttagtagatagagtataaaacaaatc-attca

gca--------aaatgcatagtttgctttctgaaattaaagtccccaattttgagaacga

actaa-aaaatcgtaaataataaattgacaggtaggtccacttttaattattaatgttaa

gaaacatttttgcgggacgaacctccatgaaaaaattcttaaaaccagtttgcttataaa

ataagacagtggatctcaacaagtgggttgaaaaa-------------------------

---------------------------------------------------------

>123bLG10

agcatgttgggtaatggcttgcaaaaatttagtagatagagaataaaacaaatcgactaa

gcaaaa-ttaaaaatacatagtttttcttctgaaattaaagtcat-aattttgagaacga

actagaaaagtcgtaaataatatattggcaagtaggtccactttgaattataaattttac

a--aaattttttcaggacgaacttccatgagaaaatcgtcaaaaccgttttgctgataaa

attagtcagtggctctcaacaggtgggttaataaaatttctgaaaaaagttcgtaaaaaa

tgtataaa-------------------------------------------------

>161LG3

agcgtgttaaggaatagtttgcaaaaatttagtagatagagattaagac--aatgattaa

gc---------aaatgca---gttttcttctaaaattaaagtcccaaattttgagaacga

actagaaaaaccgtaaataatatattaacaggtaggtccactttcaattattaattttac

taaaaattttttcaggacaaccctccatgaaaaaattctcaaaaccattttactaatgaa

ataagccagtggctctcaacaggtgagttaataaaatttctgaagaaagtacgagaaaaa

attataaggatttttaa----------------------------------------

>363LG4

agcgtgttagggaatggtttgcaagaatttagcagatagagaataga-------------

-------------------------------gatagaaaagtcccaaattttgagaacga

actagaaaaagcgtaaataatatattaacagataggtccact-----ttaataattttac

gaaaaatttattcagaacgaacctccatgaaagaatttttaaaactattttgctaataaa

ataagccagtggctctcaacagctgggttaattaaatttccg----aagtactcgaaaat

g-tataagggtttt-------------------------------------------

>230LG9

------------------------------------------------------------

---------------------------ttctgaaattaaaatacccaattttgagaacga

actagaaaaatcgtaaataatatattgacaggtagatctaatttcaattatgaattttac

gaaaaaattttccaggacgaacctccatgaaaaaattctcaaaaccattttgctaataaa

ataagccagtggctctcaacagttgggttaataaactttctgaaaaaagtacgcgaaaaa

tgtatg---------------------------------------------------

>216LG9

------------------------------------------------------------

-------caaaaaatgcattgttttccttctgaaattaaaatcccaaattttgagaacga

actagaaaaatcgttaat-atatactgacaggtaggtccactttcaattattaattttac

g-aaaatttttttaggacgaatcgccatgaaaaaattcctaaaaccattttgttaataaa

ataagccag-ggctctcaacaggtggtttactaaaatttctgaagaaagtacgcgacaaa

tttataagggtttttaactcatcta--------------------gaaatattgcta

>168LG5

agcgtgtaagggaatagtttaattaaatttaatagatagagaataaaacaaattgatcaa

gtaaaagtaaaaaatgcacaatttttcttctgaaattaaagtcccaaattttgagaacga

actagaaaaatcgtaaataatataataatagatagatcaactttcaattaataattttgc

aaaaaagtttttctgtacgaacctccatagaaaaattctcaaaac-----tgctaataaa

ataagccagtggctcttaacaagtgtgttcatgaaatttctaaagaaagtttgcgaaaaa

tgtataagagtttttaaatcgtctgatcatgacaaatttacatacgaaataatgtta

>478LG9

------------------------------------------------------------

------ttaaaaaatgca--gtttttcttcgaaaattatagtcccatattttgaatac--

---agaaaaatcgtaaataatatattgacggataggtccattttcaattattaattttac

gaaaaa--ttttcaggacgaaccttt----------------------------aataaa

ataagccagtggctttcaagtggtgggttaatataattgctgaagatagcacgcgaaaaa

tgtataagggtttttaactcgtctga-------------------------------

>456LG3

agtgtgttaggaaatggtttgcaaaattttagtagacagagaataagacaaatcgattta

gcaaaa--------tgcaaagatttccttctgaaa-taaagtcccaaattttttaaacga

actagaaaaatcgtgaataatatattgacagatagatcctttttcaattgttaattttac

gaaaaatttttt--------------atgaaaaaattctcaaaactactt----------

------------------------------------------------------------

---------------------------------------------------------

>194LG3

agcatattagaaaatggtttgcataaatttagtagatagagaacaaaacaagccttttaa

gaaaaactaaaaaatgcatag------ttctggaattaaagtcccaaattttgagaacga

aataagaaaatcgtaaataatatattgacatgtaggtctacttccagttattaattttac

gaaa--tttttttaagacgaagcttcacgaaataatactcaaaaccattttgctattaaa

ataagaccgtggctgtcaacaggtggattaataaaatttctgaagaaagtacacgaaaaa

tgtacaagggctggtaacttgtctgatc-----------------------------

>245LG9

agcgtgttagagaatggtttgtaaaaatttactagatagagaataaaacaaatcgattaa

gcaaagataaaaaatgcatagttttctttc-------------ccaaattttgagaacga

aatagaaaaatcgtaaagaatatattgacacgtaggcccactttcaattattaattttac

gaaaaattttttcagaacgaatgtccaggaaaaaattctcagaacca-tttgctaataaa

ataata--gtggctctcaacaggtgggttggtacaatttctgaagaaagaacgc------

---------------------------------------------------------

>403LG6

agagtgttagggaatggttaccaaaaatttaggagacagagaataagacaaatcgattaa

gaaaaaataaaaaatgcag--ttgtcc----------------ccaaattttgaggacga

actagaaaaatcataaatgatatactgacagttaggtccactttcaattattaatgttaa

aaa---ttttttcaagacgaatttcaataaaaaaattctaaagac---------------

------------------------------------------------------------

---------------------------------------------------------

>225bLG4

agcgtgttagggaatggtttgcaaaa-----aaagatgaagaataagacaaatctattaa

gtaaaagtaaaaaatgcatagttttccttctgaaatcaaact-caaaattttgagaacga

acaagaaaaattgtaagtaa-gtactgacagataggtcctcttacaattattaa---tac

aaaat-ttttttcgggacgaactttcatgaaaaaattctcaa---cactttgctaataaa

ataagctagtggctctgaacaggtgggtcagtaaaatttctgaagaaagtacgcgaaaaa

tgta-----------------------------------------------------

>397LG4

agcgtgttaaggaatggtttgcaaaaatttagtaaatagagaataaggcaaatcgattaa

gcaaaagtaaaaa---------ttttcttctg-------------aaattttgaaaacga

actagaaaaatcgtaaataatatattgacaggtaggcccacttttatttattaattttac

gaaaa----atttaggacgaacttcca-aaaaaaattcttaaaactattttgctaaaaaa

ataac-------------------------------------------------------

---------------------------------------------------------

>80LG2

agcgtgttagggaatggtttgtaaaaatttagtagatagagaattagacaaaccgattaa

ataaaagtaaaaaatgc--agttttccttctaaaattaaagtcccaaattttgagaacga

actagaacaatcgtaaataatatattgacagatacgtt----ttcgattattaacttttc

gaaaaattttttctggacaaacccccataaaaatgtattctgaacgattt---taataaa

acaagccagtggctctcaacaggtgggttaataaaatttctgaagaaagtacgcaaagaa

tgtataaggtattttaactcgtctgatcatgacaaattcgcattcgaaatattgtta

>298LG7

agtgtgttagggaattgtttgcaaaaatttagtagatagagaat-aaataaatccattaa

gtaaaagttaaacatgcatagttcttcttctgaaattaaagtcctaagttttgagaacga

actagaaaaatgttaaatgatatattgaaaggtaggt-----gtaacatattaattttac

gaaaaattttttcaggaagaatctccataattaaattctcaaaaccattt----------

------------------------------------------------------------

--------------------------------------------------actgcta

>250LG9

agcgtcttaggaaatggtttgcataaatttagtagacagagaacaagacaaattaattaa

gcaaaagtaaaaaatgaatagttttccttctgaaattaaagtactaaattttgataacga

actagaaaaatagtaaataatatattgaaaaataagtatacttgtatttattaattttac

gaaaagttttttctggacgaacgtctatgaaaaaattgtcaaaagcgttttactaataag

-caagccagtggctcttaacaggtgtgttaataaaattg---------------------

---------------------------------------------------------

>55LG3

---------------------------------agatatagaataagccaaattgattaa

acaaaagtaaaaaatgcatagttttccttctgaaatttaagtcacaaattttgagtacga

aattgaaaaatcgtaaataatatattg-c-------------ttcaattgttaattttac

gaaaaattttttcatgaa----------gaaaaaattctcaaaaccattttgctaataaa

ataagccagtggctctcaacaggtgacttaataaaatttctaaagaaagtgcactaaaaa

tga------------------------------------------------------

>218LG6

----------------------------------------caataagacaaatcgattaa

gcaaaagaaaaaaactcggtttttccattccaaaattaaagtcccaaattttgagaacga

acttaaaaaatcgtaaataatatattgccaggtag---tctattcaattattaattttat

gaaaaactttttcagagtgaacgtccaggaaaaaattcttaaaaccattttaccaataaa

atatgccagtggctctcaacaggtggggtaataaaatttcttaagaaagtaggcgaaaga

tgt------------------------------------------------------

>78LG9

--agcgttaaagaatggtttgcaaaaatttagtagataaagaataagacaaaccgattga

gcataagtaaaaaatacatac------ttctgaaattaaggtcccaaattttgagaacga

actagaataatagtatataatatattaacaggtaggtcccctttcaattattaattttac

aagaaaatatttcaggtag-------atgaaaaaattctcaaaaccattttgctaataaa

atgagccagtggctctcaacaggtgggttaataaaatttctcaagaaagtacgcgaaaac

tgtatgag--tttttaactcgtctgatcatgacaaatttatatattaaatattgcta

>150LG6

agtgtgttagggagtgatttgtaaaaatgtattagatagagaataagacaaatcgattaa

gcaaaagtaaaaaatgcatagttttccttctaaaattgaagtctcaaattttgagaacga

actagaaaaatcgtaaatattatattgac-------------ttcaattattaattttac

gaaaaattttttcaggacgaatctccatgaaaaatttctcaaaaccattttactaactaa

ataagc----ggctctcaacaggtgggttaataaaatttttgaagaaagtacgcggaaaa

tgtataag----------------ggtcataacaaattcacatacgaaatattgcta

>246LG3

agcgtgttagggaatggtttgcaaaaatttagtagatagagaat---------------a

acaaaa-----aaatgcag--ttttccttctaaaattaaagtcccaagttttaaggacga

gctagaaaaatcgtacataatatattgacaagtaggtccactttcaattattaattttac

aaaaaattttttcaggacgaacctccaagaaaaacttctcaaaactatttcgctaaaaag

ataagcc----------aacaggtgggtcaataaaatttctgaagaaagtatgcgagaaa

tgtataagggttttaaactcgtctgatcatga-------------------------

>279LG6

------------------------------------------------------------

------------------------------------------accaaattttgagaacga

actagaaaaatcgtaaaaaatatattgacaaatagatccactttcaattattaattttac

gaaaaattttctcaggacgaacctccgtgtaaaaattct-----ccaccttgctaataac

ataaaccagtggctctcaacaattgggttaataaaatttctgaaaaaagtacgc------

--------gatttttaactcgtctgatcatgacaaattcaaatacgaa---------

>291LG5

------------------------------------------------------------

--------------------------------------aagtcccaaattttgagaacga

actagtaaaatcgtagattatatatcgacaggta--aacacttttaattattaattttac

gaaaaattttttcatgacgaacctccttgaaaaaattttcaaaaccatttctacaacaaa

ataagacagtggctctcaacaggtgggttaataaaatttctgaagaaagtacgca-----

---------------------------------------------------------

>313LG10

------------------------------------------------------------

--aaaa------------------------------taaagtcccaaattttgagaacga

actcgaaaaatcgtaaataatatattgacaggtaggtccacttttaattattaattttaa

gaaaaattttttcaggacgaaccaccttaaaaaaattctcaaaaccatttctataataaa

ataggccagtggcactcaacaagtgggttaacaaaatttctgaaaaaattacgcgaaaca

tgtacaagagttttta-ctcgtctgatca------------caacgaaatattgcta

>332LG6

------------------------------------------------------------

-------------------------------------------ccaaattttgagaacga

actagaaaaatcgtataaaatatattgacaagtgggtccattttcaatcattaattttac

gaaaaatttcatcaggacgaacctccatgagaaaattctcaaaaccattttgttaacaaa

ataaaccagtggctctcaacaggtgggttaataaaatttctgaagaaagtacgg------

---------------------------------------------------------

>232LG3

agtgtgttaggaaatgg-tcgcaaaagtttgatagatagaaaataaaacgaatagagtaa

gtgaaagtaaaaaatacataatttttctcttgaatttaaaatcccatattttgagaacga

acttgaaaaatcgtaaat-aaatattgacaggtagcgccacttttaattattcgttttac

aaaaaatgagtacaaaacaaacctccgtgaaaaaattctcaaaattgttttgcaaataat

atgaaccagtgactctcaataggtgggttaatattttttttgaagaaagtatgcgaaaaa

tttataaaggttattaactcgtctaatcatgacaaatttacatataaaacattgcta

>252LG3

agtgtgttaggaaatggtttgtaaaagtttggtagatagctaaaaaaacgagtcgattaa

gtaaaaataaaaaacgcatagttgtccttctcaacttaaagtcccaaattttaataacga

agtggaaaaatggtaaataaaatatagtcggataggtttactttcaattattcattttac

aaaaca--attacagatcgaacctccttgaaaaaattctcaaaatcattttgctaataaa

ataaaccactgactctcaacaggcgtgttgataaaatttttgaagaaagtatgcgaaaaa

tatattagagtttttatttcgtctgatcatgacaaattcacatacaaaatattgcca

>282LG9

------------------------------------------------------------

--------------------------gttctgaacttaaagtcccaaattttgagaacga

actagaaaaatcgtgtttaaaagatgaacaagtaggtcttttttcaatt----attttac

aaaaaa--agtacagaacaaacctccattaaaaaattctcaaaactattttgcaaatcaa

cttaaccagtggttgtcaacaggcaggttaataaaatttttgaagaaagtaagcgaaaaa

tg-ataagggtttttaactcgtctgatcatggtaaattcacatacaaaatattgcta

>473LG3

------------------------------------------------------------

---------------------------gtctcaacttaaagtcccaaattttgagaacga

actaaaaaaat-----ataaaatattgacaggtaggttcactttcactttttt-------

--------attatagaacgaacctccatgaa------ctcaaaaccactttgctaataaa

ataaaccagtggctttcaataggtgggtttataatttttgtgaagaaagtatgcgaaaaa

tg-------------------------------------------------------

>373LG3

------------------------------------------------------------

----------------------tttctctttgaatttaaggtcttaaattttgagaacga

actagaaaaagcgagtataaaatattgacaggtaggtccacttttcattattaattttac

aaaaagaatctacagaacgaacctccatgaaaaaattcttaaaatttttatgctaataaa

ctaaactaatggctcccaacaggtgtgttaatattttattttaagaaagtatgcgaaaaa

tttgtaaggatttttaacttgtctgatcatggcaaattcacatcc--aatattgcta

>251LG2

agcctgttaggaaatgatttgcaaaaatttggtaagtagagaataaaacgaatcaattaa

gtaaagctccaaaatgtatagttttccttctaaacttaaggtcccaaattttgagaacga

actagaaataacgtg-ttaaaatattgacaggtaggtccact------tattaatgttgc

aaaaca--aatacacaacgaacctccatgagaaaatttccaaaaccattatgctaataaa

acgaaccagtggctctcaacaggttgattaatattttttttgaagaaaatatgcgaaaaa

tgtataagggtttttaactcgtctggtcatgacaaattcacacacaaaatattgcta

>459LG8

------------------------------------------------------------

----aagtaaaaaatgcatggtttttcttctgaacttttgttctcaaaatttgagaacga

actagaaaattcgtaaataataaattggcagctaggttcactttcaattactaagtttac

g-----ttttttcaagacgaaccttcatgaa--------------tgcctcgctaataaa

ataagccagtggctctcaacaggtgggttaatattttttttaaagaaagtacgcgaaaaa

tttataagggtttttaactcgtctgatcatgacaaattcacttacacaatattgcta

>151LG3

------------------------------------------------------tattaa

gtaaaagtaaaaaatgcatagtttttcttttgaaattaaagtcccaaattttgagaacga

actaagaaaatcgtaaatattaattgac-aggtaggtccactttcaattattcattttac

aaaaaa---tttcaggacgaatttccatgaaaaaattctcaaaaccattttgctaataaa

ataagccagtggttctcaacaggtgggttaataaaatttctgaagatagtacgc-aaaaa

tgtat--gagtttttaactcgtctgatcatgataaattcacatacgaaatattgctg

>292LG3

------------------------------------------------------------

---------------------------------aattaaaattccaaatttggagaacga

aatagaaaaatcgtaaataatatattgccaggtaggtaattattcaatta---attttac

gaaaaacatttttaggacgaacttgcaggaaaaaatttacaaaaacattttgctaataaa

gtaagccagtgactctcaacaggtaggttaataaaaattctgaagaaagtacgcaaaaaa

tgtacaagggcttttaactcgtctgatcatgacaaattaacatacgaaatattgctg

>244LG9

-------------------------------------agggagtaaaacaaatagattga

gtaaaagtaaaaaatgcattgttttcc----gaacttaaggtcaaaaattttgagaacca

actagaaaaatcgtatataatatattgacaggtaggttcaattttaatttttaattttcc

g-----ttttttaaggacgaatccctatgagaaaattcttaaaaccattttgctaataaa

------cagtggctctcaacaggtgggttaat-aaatttctggagaaagtacgtgaaaaa

tgtataaggatttttaactcgtctgatcatgacgaattcgcatacgaaatattgcta

>409LG4

-----------------------aaaaatgaatagataaagaat-aaatgaattgattaa

gaaaaagtaaaaaat--acaattttccttctgaaattaaagtcccaaattttgagaacga

attagaaaaatggtgaataatatattgacaggctggtccaccttcaattattaattta--

-aaaaattttttcagaacgaacccat----aaaaattctcaaaaccatgttgttaataaa

------cagtggctctcaacaagtggagtaataaaagtt---------------gaaaaa

tgtataaggg-------ctcgtctgattatgacaaattcacgtatgaaatattgtta

>461LG3

------------------------------------------------------------

------ttaaaaaatgcatagttttccttctaaaattaaagtcccaaattttgagaacga

atttaaataatcgtaagtaatatactgagagataggaccaccttcaattattaactaa--

-aaaa-ctttttcagaacgaacctatttgaaaaaattctcaaaac----------ataaa

------cagtggctctcaacaggtgggttaataagatatctgaagaaattacccgaagaa

tgtataagagattttaacttgtttgattatgacaaattcacatgcaaaatattgcta

>211LG7

agcgtgtagggaattgtttagcaaaaatttagtagatagaaaatacgacaaatcgattaa

gcaaaagtaaaaaa-------------ttctgaaattaaagtcccaaattttgggaacga

actaaaaaaattgaaagtaatatatttat---tgggttcactttcaatttttaattttaa

gaaaaactttttcaggacgaacctccatg--aaaattctgaaaattattttgctaataaa

ataaaccagtggctctcaacaggtgggttaataaaatttccgaagaaagtactcgaaaaa

tgtccaagggtttgtaacatgtctgattatgacaaattcacatatgaaatattgcta

>217LG9

agcgtgttagggaatggtttgcaaaaatttagtagattgagaataagacaaatcaattaa

gcaaaagtcaaaaatgcataattgtcattttgaaattaaagt-----------------a

aataagaacatcgtaaatgatatattggcaggtaggtttactatcaattattaattttac

aaaaaa-tttttcagcatggacctccatgaaaaaattctccaaaccattttgataataaa

ataatccagtggctctcaacaggttggttaataaaatttgtgaagaaaatatgcgaaaaa

attataagggtttttaactca--tgatcatgacaga--------cgaattattacaa

>333LG5

agcgtgttaggaaatggtttgcaaaaatttagtagatagagaataaaacaaatcaattaa

gtaaaagtaaaaaatgcatagttttctttctgaaattaaagtccc---------------

-------aaatcgtaaataatatgttgataggtaggtttacctttaattattaattttac

gatt--ttttttcagggccaacctccatgattaaattctcaaaaccattttgctaataaa

ataagccagaggttctcaataaatgggttaataaaatttctgaagaaagtacga------

---------------------------------------------------------

>193LG4

agcgtgttaagatgtggtttgcaaaaatttagtacatagagaataagacaaatcgattaa

gcaaaagtaaaaaatgcataattttccttctgaaattaaagttccaagttttgagaacga

actagataaatcgtaaataatatattgacaattaggtctactttcaaatattaatttttc

gaaaaattttttctcgac--------atgagaaaattctcaaaacaattttgctaataaa

gtaagctagtggcttctaacaggtgggttaataaaatatctgatgaaagtacgc------

---ataagggtttttaacttgtctgattatgacaaattcacatacgaaatattgcta

>199LG2

agaggattagggaatggtttacaaaaatttagtaaatagagaataagaccaattgattaa

gcaaaagtagacaatgtttagttttccttctgaaattaaagtcccaaattttgagaacga

actagaaaaatcgtaaataatatattgatagggaggtccactttcaattattaattttac

aaaaagttttttcaggac--------aaaaaaaaattgtcaagaccattt----------

ataaaccagtggctctcaacaggtgcgttaatacaatttcagaagaaagtgagcgaaaaa

tgtgtaaggttttttaactcgtctaatcatgacaaattcacgtacaaaacattgcta

>241LG10

agcgtgttagggaatggttttcaaaaatttagtagatagaaaataagacaaatcgattaa

gc---------taatgcatagttttccttttgaaatttaagtcccagattttgagaacga

actagaaaaatcgtaaataatatattgacaggtaggtccacttttaattattaattttac

aaaaaaaaaattcaggac--------at---aaaattctcaaaactattttgccaataaa

ataagccagtggctctcaacaggtggattactaaaatttctgaacaaagtacgcgaaaat

ga---acgggtttttaact-------------------------ctaagctatgcta

>483LG6

agcgtgttaaggaatggattgcaaaaatttagtggatagagaataagacaaatctattaa

gtaaaagt-------------------ttctgaaattaaagtcccaaattttgagaacga

actataagaatcataaataatatattgacgtgtaggtgcactttcaattattatttttat

gaaaaatt-------------------tgaaaaaattctcaaaaccattttaataataaa

ataaaccagtggctctcaacaggtgggttaatatggattctaaagaaagtacgtaaaaaa

tgtataaaggtttttaactcgtctaattatcacatattcacatacaaaatattgcta

>179LG9

agcgtgttagggaatggtttgcaagaattttgtagatagagagtaacac--atcgattaa

gcaaaagtaaaaaatccatagttttccttctgaaattagagtcccaaattttgagaacca

actagaaaaatcgtacataatattttgacaggtaggtccacattcaattaat-at-----

-aaaaattttttcaggacgaat---------aaaattctcaaaaccattgtgctaat-aa

ctaaggcagtggctctcaatagatgggttaataaaatttctcaagaaagtacgcggaaaa

tgtataagggttg--------------------------------------------

>276LG5

aatgtgttagaggatggtttgcaaaaatttagtagattgaaaataaaacaaaccaattaa

gcaaaagaataaaacgcatagttttccttttgaaattaaagtcccaaattttgagaacga

actaaaaaaatcgtaaataaa--attgacaagtaattcctctttcaa-ta----------

-aaaaattttttcaagacgaacctcgatgaaaaaattcccaaaaccatat--------aa

atatgcaagtggctttcaacagatgggttaataaaatatctgaag-aagtacgcgaaaaa

tgtataagggttttaa-ctcatttgatcatgaaaaattcac----gaaatattccta

>227LG3

--tgtgttagggaatagtttgcaaaaatttagtaaatagagaacaagacaaatcgattaa

gcaaaagtaaaaaatgcatagtttttcctctgaaattaaagtcccaaactttgagaacga

attattaaaatcgtaaataatatattgacgggtagttccacttttaattattaattttag

gaaaaattttttcaggacgaacctccataaaaaaattttcaaaaccattttgctaataaa

ggaagccagtagctgtcaacaggtaggtaaataaaatttctgaa----gtttttgaaaag

tgtata---------------------------------------------------

>234LG3

agggtgttaaggaatggcttgcaaaactttagtaggtagggaataagacaaatcgatgat

gcaaaagtaaaaaatgcatagtttttcttctgaaattaaagtcccaaattttgagaacga

gctagaaaagttgtaaataatattttcacaggtaattccacttgccattattaattttat

aaaaaagtttttcaggac-------------gaaaacatcaaaacctctttactaataaa

agaagacagtggctctcaataggtgggttaataaaattcctgaagaaaatacgcgaaaaa

tgtataagggtctttaacttgtctgatcatgacaaattcacacacaaaatattgcta

>195LG10

agcgtgttggg--gtggtttgcaaaaatttagtagatagagaat----------gattaa

gcaaaagtaaaaaattcatatttttccttttaaaattaaagccccaaattttgagaacga

actgggaaaatcgtaaataatatattgacaggtaggtccactttcaattattaattttac

aaaaaatttttt--------------atgaaaaaattttaaaatccatttaattaattaa

ataaatcagtggctgtcaacaggtgggttaataaaatttttgaagaaagtatgcgaaaaa

tgtatgag--tttttaactcg-ctgatcatgactaattcacatacgaaattttgcta

>226LG3

------------------------------------------------------------

--aaaa-----aaatgcatagttttccttctgaaattaaagt-cccaattttgaaaacga

attaga-----------taatatactgacaggaagggccactttcaattattaattttac

gaaaaaaattttctggacgaacctccaggaaaaaattttcaaaaccattttgctaataaa

ataagccagtggctctcaacaggtgaattaataaaatttctgaagaaaatacgcgaaaaa

tttataagagttttcaactcgtcagatcataacaaattcacatacgaaatattgcta

>196LG8

-----------------------------------------------ac-----------

--aaaagtaaaaaatgcatagttttccttctgaaattagagttccaaattttgagaacga

tctacgaaaatcgtaaatgctatattgacaggtaggttcactttcaataattaattttac

gaaaaattttttcaggacgaattttcatgattaaattctttaaaatatttaactgataat

ataatccagtggctctcaataggtgggttaataaaatttctgaagaaagtatgcgaaaaa

tttataagggtttttcacttgtctgatcttgacaaatgcgcatacgatatattgcta

>285LG5

------------------------------------------------------------

---------------------------gtctaaacttaaacttccaaattttgagaacgg

actagaaaaatcgtaaataatatattgagaggtatggtcccttttaattattaagttt--

-agaaattttttcaggacgaacctctatgaaaaaattctcaaaaccatttttctaataaa

aaaaaccagtggctctcaacaggtgggttaatataatttctgaagaaagtacgcgaaaaa

tgtataaag-----------gttaaa------gaaattcacatacgaaatattgcta

>183LG8

agcgcgttagggaatggtttgcaaaaatttagtaggtagaaaattaaacaaatcggttaa

gcaaaagtaaa-----catagtttttcttctaaaatcaaagttccaaattttgaaaacga

actagaaaaatcgtaattaatattctaac---tggatccactttcaattattaattttac

aaaaaa------------ttgcctccatgaaaaaattctcaaaaccgttttgccaataaa

ataagc-agtggctctcaacaggtgggttaataaaatttctgaagaaaaaaa--gaaaaa

tgtataagggtttttaattcgtctgatcatgacaagtttacatgcgaaatattgccc

>423LG9

agcgtattagggaatgatttgcaaaaatctaatagatagagaataagataaatcgattaa

gcaaaagtcaa----------------ttctgaaattaaagtcccaaattttgagaacga

actagaaaaatcgtgaataatatattggcaattaggtccacttacaat------------

-aggaa------------gaacctccattaaaaactcatcaaaactattttgctaataaa

ataagctagtggctctcaacaagtcggttaataacatttctggagaaagtagatgaaaca

tgtataaggctttttaactcgtctgatcatgacaaattcacatacaaaatattgata

>396LG8

------------------------------------------------------------

---------------------ttttccttctcaagttaaagactaaaattttgacaacga

actagaaaaac------taatatattgtcaggtaagtccattttcaatcagtacggttac

gaaaaattttttcaagac--------atgaaaaaattctcaaaaccattttgctaataaa

ataagtccgtggctctcaacaagtgggttactaaaatttca-------------gaaaaa

tgtataagggttttcaactcgtctgattatgaaaaatt---acacaa----------

>344LG3

------------------------------------------------------------

----aagtaaaaaatgcatggttttccttctgaaattaaagtccaaaattttgagaacga

aatagaagaatcgtaaataataaattgacaggtaggtttgtt------cattaattaaaa

aaaaaa------cgggacgaacctcgatgaaaaacttctcaaaactattttattaatgaa

atacgccagtggctctcaacaggttagtcaatagaatttctgaaaaaagttttggaaaaa

tttatgaggattcttaactcgtctgatcatgacaaattcacgtacgaaacaatccta

>386LG3

agcgt-ctagggaatggtttgcaaaaatttagtagatagaaaataaaacaagtcgactaa

gcgaaagtaaaaaatgcatagttttccttttgaaat-------taaaattttgagaatga

agtaga-----cgtaaatattatattgat-------------------taataatgttac

acaaaatttttttagtacgtacctccaggaaaaaattctcaaaactgttttgctaataaa

ataagccagtggctctcaaaaggtgggttaataaaatttctgaagaaagtacacgaaaaa

tgtgtaagggtttttaactcgtatggtcatgacaaattcacattcgaaatattgctg

>284LG9

------------------------------------------------------------

------------------------------------------------------------

-----------cgtacataatactatgacaagtaggtccactttgaatcattaattttac

gaaaaatttttg------aaacctccatgaaaaaattctcaaaaccattttgctaataaa

acaagccagtggctctcaacaggtggattaataagatttctaaagaaaattcgtgaaaaa

tgtatgat---------ctcgtctgattatgaaaaattcacaaacgaattattgcta

>360LG9

agcgtgttaaagaatggtttatcaaaatttaatagacagagaataagacaaatcgattta

aaaaaagtaaaaaatgcatagttttccttctgaaatgaaagttccaaattttggaaac-g

actagaaaaatcaaaaataatatatcgacaagtaggttcaccttcaattattaattttac

gaaaaatttttccagaacgaatctccatgaaaaaatttt-----------tgctaataga

ataagccagtggctctcaacacgtgggttaataaaatttctgaagaaagtacgcgcaaaa

tctataag----tttaactcgtctgatcatgacaaattcacatacgaaatattgctc

>331LG3

------------------------------------------------------------

------------------------------------caaagtttaagaag--------ga

actagaaaaatcgtaaataatatattgac-----agtccactgtcaattattaattttac

gaaaaattttttcaggacgaacccccatgaaaaaattctcgaaactactttgctaataaa

atacgctagtggctctt---------------------tctgaagaaagtacgcgaaaaa

tgtataag---------ctcgtctgatcacgacaaattcacatacgaaatattgcta

>277LG9

------------------------------------------------------------

---------------------------ttctggaaataaagtctcaaattttgaaaacga

acaaaaacaatcataaataatatattgtcagacaagcccact------tatt-gttttac

gaaaaatttattctcgacgaacctccaggaaaaaattttcaaaaccattttgcttataaa

ataagccagtggttcttaacaggtgagttagtaaaatttctgacgaaagtacgcgaaaaa

taaataaaggtttttaactcgtcta---------atttcacatacgaaatattgcaa

>330LG3

-------tagagaatgg--------------acaaatgattaat-----taattaatcga

ttaaaagtaaa----------------ttctgaaattaaagtcctaaattttgagaacga

actaaaaatac------tattgtatggataggtaggtccacttttagatattaattttag

gaaaaattttttcaaaacgcaccttcatgaaaaaattctcgaaaccattttgctaataaa

ataacccagtggctctcaataagtgggttaatcaaaagtctgaaaaaagtacgcgaaaaa

tgaataaagatttataactcgtatgattatgacaaattcacatacgaaatattgcta

>281LG3

agcgtgttagggaattttttgcaaaactttagcagaaagaaaataagctaaagcaattaa

gcaaaagtaaacagtgtatcgtttcccttctgaaattaaagtcccaaattttgagaacga

cctagaaaaatcgtatgtaatatattgacaggtaggctcattttcaactattaatttttc

gaaaaactttttcagaacgaaccttcatgaaaaaatctc------cgttttgctaataaa

ataagccagaggctctcaacaggtgggttaataaaaattctgaaaaaa------------

--tataaatttttttaattcatctgatcatgatttatatacatacgaaatattgata

>306LG7

---------tgaagtggtttcaaaaaatttaataaatggagaataagataaatcgattag

gcaaaagtaaaaaatgtatagtattccttctgaaattaaagtcctaaattttgagaacga

actagaaaaatcgtaaataatatattgacaagta--------ttcaattgttaattttac

taaaaatgatttcaggacgaacctccatgaaaaaattttcaaaaccattt----------

------taaccgctttc------------------------gaagaaagtacgctaaaaa

tgtttaagagtttttaactcgtctgatcatgacaaattcaaatacgaaattttgcta

>428LG7

agtgtgttaaggaatagtttgcaagaattttgcaaatagagaataaaacacatcaattaa

gtaaaagtaaaaaaagc----------ttctgaaattgaagttccaaatattgaaac-ga

actagaaaaatcgtaaa-----------------ggtcctcttttaattattaattttaa

taaaaaatttttcaggacaaacctccatgaaaaaattctcaaaaccattttgttagtaaa

------cagtggctcttaacagatgggttaacaaaatttctgaagaaagtacgctaaaaa

tgcctaagggtttttaactcatctgatcatgaacgaat-------------------

>367LG8

agcaag-tagggaatgatttgcaaaaatttaaaaaaaagaaaataaaacaaatagattaa

gcaaaagtaaaaaatgcctagttttccttctgaaat--------------taaagaacga

actagagaaattataaataatacattgacaggtaggtctactttcacttataaatttaac

gaaaaattttttcaagacgaacttccatgaaaaaattctcaaaac---------------

-----------------aa-----------------------------------------

---------------------------------------------------------

>417LG5

agcgtattagggaatggtttgcaaaaatttagtagatagagaatcagacaaaacgattaa

gcaaaagtaaaaaatgcatagctttccttctgaaattaaagttccaaatttcgagaacga

actagaagaaccgtaaataacatattgac-----ggtccacttccagttattaaatttac

g-------ttttctggacgaatcttcatgaagaaattct---------------------

------c-----------------------------------------------------

---------------------------------------------------------

>421LG7

agcctgttagggaatggtttacaaaaatttgatagatagagaataaaacgaatcgattaa

gtaaaagtataaa------------tgttctgaaattacagtcccaaattttgagatcga

actagaaaaatcgtgtataaaatattgacaggtaggttcactttcaattactagttttac

aaaaa-atttgacagaaggaatctccatgaaaaaattctcaaaaccattt----------

------------------------------------------------------------

---------------------------------------------------------

>431LG3

---ctgttaaggaatagtttacaaaaatttgatagatagaaaataaaaagaatcgattaa

gtaaatgtataaaatgcataacttttcttctgaactcgc--tcccaaattttgagaacga

aatagaaaaatcatgtctaaaatattgacaagtaggtccacttttaattattaattttat

aaa---------cagaccgaacctccatgaaaaatttttcaataccattttgctaataaa

ataaaccagtggctcgcagtgtgtgggttaataaaatt----------------------

---------------------------------------------------------

>442LG10

accctattagagaatggttggtaaaaatttagtaaatagagaattagataaatcgattaa

gctaaagtaaaaagtgcatag---------------caaagttccaaattttgagaaaga

actagaaaaatcgtaattaatatattaatatataggtccactttcaattagtaatttttc

gacaa-tttttcc------aacctccatgataaaattcttaaaactattttgcttataaa

atataccagtggttctcaacaggtgggttaataaaaattctgaagaaagtacgcgaaaaa

tgtataagg------------------------------------------------

>355LG7

agcgtgttggagaatggtttataaaaatttggtagatagagaataaaacaaatctattaa

ataaaagtaaaatatgaaaaagttatattctgaacttaacgttacaaatttttagaacga

agtagaaaaatcaaaaataatatattgacaggtaaatccactttcaattattaatttttt

aaatag------taaaacgaacttccgcgaaaaaattttcaataatattttgctaatggt

ataaaccagtggctctgaacaggtaggttaataaaaaaaattaaggaagtatacgaaaaa

tgtataagagcttttaactcatt--atcatgtcaaattcacatacaaaaaattgcta

>321LG9

------------------------------------tagaagataaaataaaccgattaa

gtaaaaataaaaaatgcatagttttccccctgatattaaaatcacaaatttcgagaaaga

attagaaaaatcgtaaataataaattgacaggtaagtttatgttcaattattaattttct

aaaaaaaaattacagaacgacctcccatgggaaaattttcaaaaccactttgctataaac

ataaaccagtggctctc-----atgggttaacaaattgtatgaagaaagtatgcgaaaaa

tgtataag-gtatttaactcctctgatcatgacaaat--------------------

>325LG10

-------------------------------------------------aaatcgattaa

gtaaaagtaaaaaatgcataattttcc--ttcaaattaacgtctaaaattttgagaacga

actagaaaaatcgtaaataatatattgac-----gatccacttttagttattaattttac

gaaaaagtttttccagacgaacctccatgaaaaaattcttacaactgttaagctaataaa

agcaaccagtggttcccaacaagtggattaataaaa------------------------

---------------------------------------------------------

>42aLG10

---gtgttaaggaatggtttgtaaaaatttagtagatagtgaataaaacaaatcgattag

gcaaaaataga----------ttttccttctaaaattccagtcccaaattttgagaacaa

acttaaaaaatagtaaataatatattgacaagtaaatccttttccaattattagttttac

gaaaaa-------------------------aaaaattt------tattttgctaataaa

acaatctaatggctctcaataggtggattaataaaatttctgaagaaagtacgcgaaaaa

tgtataaaggtttttaactcgtc----------------------------------

>369LG5

---------------tgtttgctaaaatttagtaggtacagaataagataaatcgattaa

gtaaaagtaaaaaatgcaaagtttttcttctaaaattaaagtcccaaattttgagaacga

actagaaaaatcgtaaataatatattgacaggcaaattaactttcagataataattttac

gaaaaatttgtt--------------ataaaaaaattcttaaaac-----tgctactaaa

------cagtgactatcaacaggtgggctaataaaatttctgaagaaagtacaggaaaaa

tt------agttttcaaatcgtctgatcatcacaaattcaca---------------

>457LG3

-------taggaaatactttgcaaaaatttagtagatagagaataagacaaagcgattaa

gcaaaagagaaaattgcatagtttttcttctgaaattaaagtctcaaattttgagaacga

actagaaaaatcataaataatatattgac-----tgttcact------tattaattttac

gaaaaa--------------------atgacaaaattttcaaaactattttgctaataaa

ttaagtcagtgggtctcaacaggtgg-------acatttctgaa----gtacgc------

-------------------------aacatgacaaattcacatacgaaatattgcta

>337LG5

------------aatggtttgcataaatttggtagat-aaaaataaaacgaatcgatt--

-taaaactataaaatgtatacttttcctcctgagtttaaagtctctaattttgagaacga

actggaaaaatcgtg--taata-actgacaaaaaggccgactttcaaaaattaattttac

aaaaaa------cagaacgaacttcaatgaaaaaattctgaaaactattttgctaataaa

ataaatcagtggctctcaacagatggatgaataaaatttttgaagaaagtaagcaaaaaa

tgtagaaaagttttcaacacgtctg---atgacacactcacataaaaaatattgcca

>481LG8

------------------------------------------------------------

---------------------------ctgggatgtaatggtcccaaattttgagaacga

actagaaaaatcatgcataaaatattgacaggtaggtccactttcaattattaattttac

aaaaaa------caaaat--------atgaaaagattttgaaaaccaatttgctgataaa

ataagccagtagctctcaacaagtaagttaatt---ttttttcaaaaatcatgcgaaaaa

tt-------------------------------------------------------

>411LG4

------------------------------------------------------------

---------------------------ttctgaaattgaagtcccaaattttgagaacga

actagaaaaatcgtaaataatatattgacaggta--------ttcaattattaattttac

gaaaaatttgttcaagac--------atgaaaaaattttcataactgttttgttcataaa

gtaagccagtggctctcaaccggtgggttaacaaaatttctgaaaaaagtacgcggaaaa

tgtataagggtttttaacgcgtctcattatgacaaattcacatacgaattgttgcta

>398LG8

------------------------------------------------------------

-----------------------------------------------att----------

-----------------------attgacaggtaggttcact------gattaattttac

aaaaaa---tttcaggacgaacctccataaaaaaaaattaaaaactatttcgctactaaa

ataagccagtggctctcaacaggtggggtaataaaaattctgaagaaagtacgagaaaaa

tg------gggttttaactcgtttgatcgtgacaaattcacatagaaaatattgcta

>487LG8

--------------------------atttcgtagatagagattaaaagaaatcaattaa

gtaaaagtaacaaatgcctagtttcccttctgaacttaaagtcccaaatttt-agaatag

attatagaaatcgtaaataatatattgacgattaggtccactttcaggtgttaactttat

aaaaaa------------------------------------------------------

------------------------------------------------------------

---------------------------------------------------------

>422LG3

-acgtgttagggaatggtttgcagaaatttaacaaatagagaataagac-aatcaattaa

gcaaaagtaaaaaatgcatagtttcctttctgaaattgaagtcccaaattataacaat--

------ataat------tattatattgaaaggcaggtccactttcaattattaattttac

gataaactttttcagaacaatccaacatgaaaaaatttttaaaaccattt----------

------------------------------------------------------------

---------------------------------------------------------

>441LG2

agcgtgttaggaaatggtttgtaaa------atagagagaaaaaaaaatgaatggattaa

gttaaagtataaaattcatagttttccttctgaactaaaagtcccaaattttgaaaacga

actagagaagtcgtat-taattaattgacagaca-----actatcaattattaattttac

aaaaaa-----tccgaacgaacctctatggaaaaattctcaaaac-----tgctaataaa

ataaacctgtagctctcaacagctgagttaataaaatttttgaagaaagtatgcgaaaa-

---------------------------------------------------------

**B Alignment of repeats belonging to dispersed TCAST4 elements**

>0142_LG10

------caaa taactccgaa tgtttaggat ttaggttatg gttgtaa--t aacatttatc

gttttttaat tcagcatcga ataatataaa aacaaataag ggttgtcatt aaaataagca

aaacaaaagc tctaaaaatt aaatagtttg gaaatatgta atcaccctgt agtaattcaa

atctcactta attagttaga ccttgtttaa cactacgtag taaaaatgtc ttcaaacaag

cactgtttct attttataat ttttaaatct aaaggtgtaa aaccgttttt ttgttttttt

ttttaacagt aat------- ---------- ---------- ---------- ----------

---------- ---------- ---------- ---------- ---------- ----------

---------- ---------- ---------- ---------- ---------- ----------

---------- ---------- ---------- ---------- --------

>0047_LG3

ttttagcaaa taactccgaa tgtttaagat ttacgtaatg tttgtattaa taagttg---

attttttaat tcagcatcga atgatataaa aataagtagg ggttgccatt taaataagca

aaataaaagc tctaaagatt aaaaagtttg gaaatatata aatgccctat agtaattcaa

atcaaactca cttagttaga ccttgtttaa cattctgtgg taaaaatgtc ttcaaacaag

tactgtttct gttttttaat ttttaaaact aaaggtgtaa aaccgatttt ttgttttttt

ttttaacagt aatgcctgaa ccgatttaaa aaaatttaag acatgtaaaa agctaaaata

tct-gtgagc acaaaaaata atagcttctc attaaaaaaa tatgattatg acgtcatact

agggacactc tgtataaaaa tcttttcctg agtccttaaa ------taca aaatatcaaa

actccaactt tataacgagc tatagaagac gggagcagag ctggacca

>0025_LG3

ttttatcaaa taactccgaa tgtttaggac atatgttatg gatgtattaa taagttgatc

gttttttaat tctgcatcga atgatataaa aataaatagg ggttgccatt taaataagca

aaataaaagc tctaaaaatt aaaaagtttg gaaatatgta aacaccccgt agtaattcaa

atcaaactca ctt-gttaga ccttgtttaa cactacgtag taaaaatgtt ttcaattgag

cactgtttct attttttaat ttttaaaact aaaggtgtaa aaccgatttt ttgtattttt

ttttaacaat aatccttgaa cggattaaac aaaa-ttagg acatgtaaga agctaaggta

tctaatgagt a-aaaaaata atagcttgcc attaaaaaaa tatgactatg acgtcaaact

agggacactc tgtataaaaa tatttttttg agtccttaaa tacctctaca aaatatcaaa

cttacaactt tataagaagc tatagaagac gggaactgag ctggacca

>0024_LG9

tttcatcaaa tgactccgaa tgtttaggat ttatgttatg gttgtattaa taagttgatc

gttttttaat tctgcatcga atgatataaa aatagtttgg ggttgccatt taaataagca

aaataaaagc tctaaagatt aaaaagtttg gaaacatgta aacaccctgt aataattcaa

atcaaactca ctt-gttaga ccttgtttaa cactacgtag taaaaatgtt ttcaaataag

cactgtttct attttttaat ttttaaaact aaagttgtaa aactgatttt ctggttttat

ctttaacagt aatccctgaa ccgatttaaa aaaatttagg acatgtaaga agctaagata

tc--atgagt acaaaaaata atagcttgcc attaaaaaaa tatgactatg acgtcatact

agagacagtc tgtataaaaa tattttcctg agtccttaaa tacctctata aaatat----

----caactt tataaggagc tatataagac gggagctgag ctggatca

>0154_LG9

---------- --------aa tgtttaggat ttacgttatg gttgtattaa atagttgaat

gttttttaat tctacatcga ataatataaa aataaatagg ggttgccatt taaataagca

aagtaaaagc tctaaagatt aaagagtttg gaaatatgta aacaccctgt aataattcaa

atcaaactta cttaattaga ccttgtttaa cactacgtag taaaaatgtc ttcagataag

cactgtttgt atattttaat ttttaaaact ataa-tgtaa aatcgatttt tt--------

---------- ---------- ---------- ---------- ---------- ----------

---------- ---------- ---------- ---------- ---------- ----------

---------- ---------- ---------- ---------- ---------- ----------

---------- ---------- ---------- ---------- --------

>0118_a_LG5

ttttatgaaa taactt--ag ttattaggat ttacgttatg gttgtattaa taagttggtc

gttttttatt tctgcattga atgatataaa aataaatagg ggttgccatt taaataagca

aaataaaagc tctgaagatt aaaaagtttg gaaatatgta aataccctgt agtaactcaa

atcaaactca cttagttaga ccttgtttaa cactacgcag taaaactgtc ttcaaacaag

cactgctttt attttttaat ttttaaaaca ataag-gtaa aacaattttt ttatttt---

---------- ---------- ---------- ---------- ---------- ----------

---------- ---------- ---------- ---------- ---------- ----------

---------- ---------- ---------- ---------- ---------- ----------

---------- ---------- ---------- ---------- --------

>0041_LG2

ttttatcaaa taactccgaa tgtttaggat ttacgttatg gttgtattag taagttgatc

gttttttatt tctgcattga atgatataaa aataaatagg gcttgccatt tagataagca

aaataaaagc tctaaaaatt aaaaagtttg gaaatatgta aacaccctgt agtaattcaa

atcaaattta cttagttaga ccatgtttaa caatacgtag tacaaatgtc ttcaaacaag

cactgttt-- -ttttttaat tttgaaaa-t aaaggtgtga aactgatttt tttgctttt-

-------ggt aatccctgaa ccgatttaaa aaaatttaaa acatgtaaga agctaaggta

tctaatgagc acataaaata ttagcttgct attaaaaaaa tgtgactatg acgtcatact

agggtcactc tgtataaaaa tattttccta agtcctccaa taactgtaca aaatattaaa

cctccaactt tacaacgagc aataaaagac gagagctaag cgggacca

>0066_b_LG7

ttttatcaaa taactccaaa tgtttaggat ttacgttatg attgtattaa taagttgatc

gttttttaat tctacatcaa gtgatataaa aatatataaa ggttgctatt taaataagca

aaataaaagg tctaaagatt aaaaagtttg gaagtatgta aatactctgt agtaattcaa

atcaaactca cttagttaga ccttgtttaa cactacatag taaaaatgtc tttaaacaag

cactatttct attttttaat ttttaaaact taatgtgtaa aagcgatttt ttgttttttt

tattaacagt aatccctaaa ccgattaaaa taaatttagg acatataaga agctaaggta

tctaatgagt tcaaaaaata atagtttgcc attaaaaaaa tatgactatg acgtcaaact

aaggacattc tgtataaaaa tattttct-- ------gaaa taactctaca aaataataaa

accccaactt tataacgagg tatagaagac ggatgctgag ctggacca

>0028_a_LG3

tttcagcaaa ttactccaaa tttttagatt ttacgttatg gttgtgttaa aaagttgatc

gtgttttaat tctgtttcga atgatgtaaa aataaatagg ggattcta-t taaattagca

aaataaaagc tcta-aaatt aaaatgtttg gaaatatgta cacaccctgt agtaattcaa

atcaaactca cttagttaga ccttgtttaa cactatgtag taaaaatgtc ttcaaacaag

gaattttgtg tttttttaat ttctaaaact aaaggtgtaa aaccgatttt ttggttttat

ttttaacagt aattcctcaa ccgatttgaa aaaattttgg acttgtaaga aggtaa----

---------- ---------- ---------- ---------- ---------- ----------

---------- ---------- ---------- ---------- ---------- ----------

---------- ---------- ---------- ---------- --------

>0028_c_LG3

tttccgcaaa taactccaaa tttttaggtt ttacgttatg gttgtattaa aaagttgatc

gtgttttaat tcttttttga atgatgtaaa aataaatagg ggattcca-t taaattagca

aaataaaagc tctaaaaatt aaaatgtttg gaaatatgta aacaccctgt agtaattcaa

atcaaactca cttagttaga ccttgtttaa cactacgtag taaaaatgtc ttcaaacaag

gatt-tttgt gttttttaat ttttaaaact aaaggtgtaa aaccgatttt ttgttttatt

ttttaacagt aattcctcaa ccggtttgaa aaaatttggg acttgtaaga agctaag---

---------- ---------- ---------- ---------- ---------- ----------

---------- ---------- ---------- ---------- ---------- ----------

---------- ---------- ---------- ---------- --------

>0048_LG6

ttttatcaaa taactccgaa tgtttagtat ttacgttata gttgtattaa aaagttgatc

gttttttaat tttgcatcgt a-aatataaa aataaataga ggttgttttt taaataaaca

aaataaaagc tgaaaagatt aaatagtttg gaaatatgta aacaccctgt agtaattcaa

atcaaactca tttccttaga ccttgtttaa cactacgtag taaaaatgtc ttcaaacaaa

ccttgtttca gttttttaat tattaaaact aaagatgtaa aaccgatttt ttgttttttt

tattaacaat aatcccagaa ccgatttaat gtgttttagg acatgtaaga agctaaagta

tctaatgagc acaaaaaata ataacatgct attaaaaaga tgttattttg acgtcacact

tgagccactc cgtataaaaa tattttcctg agtccttaaa taactctaca atatatcaaa

actcctactt tgtaacgagc tacagaagac tggagctgag caagacca

>0035_LG6

ttttatcaaa taactctgaa tgtttaggat ttacgtt--- --------aa aaagttgatt

gttttttaat tttgcatcgt ataatataaa aataaataga ggttgttttt taaataagca

aaataaaagc tctaaagatt aaatagtttg gaaatatgta aacaccctgt agtaattcaa

atcaaactca cttagttaga tcttgtttaa cactacgaag taaaattgtc ttcaaacaaa

cactatttca gttttttaat tattaaaact aaaaatgtaa aaccgatttt ttgttttttt

tttgaacaat gatcccagaa ccaattt--a atattttaga acatgtaaga agctaagcta

tctaatgagc acaaaaaata atagcatgct attaaaaaaa tagtattatg acgtcacact

tgggatcctc tgtgtaaaaa tattttcctg agtccttaaa taactctaca aaatatcaaa

actcctacat tgtaacgagc tacagaaggc cggagctgag caaaacca

>0053_a_LG8

ttttatgaaa taattccgaa tctttaggat ------taga gttgtaataa aaagttgttt

gttttttaat tctgcattaa acaatataaa aataaatagg ggttgccatt taaataagca

aaataaatgc tctaaagatt aaaaagtttg gaaacatgta aacactctat agtaattcaa

atgaaactca cttagttaga ccttatttaa cctaacctag taaaaatgtc ttcaaacaag

cactgtttct gttttttaat ttttaaaact aaaggtgtaa aat--atttt ttattttttt

tcttaacaat aattccagaa ccgatttaac aagttttagg acatgtaaga agctagggta

tctaataaac acaaaaaata atagcatgcc ataaaaaagt tattattatg acgtcatact

tgggaccctc tgtatcaaaa tatttttctg agcccttaag tacctcctca caatatcaaa

aatccaactt tgtaacgaga tacaggaaac gggtgctggg atggacca

>0049_b_LG6

ttttgacaaa taactccgaa tatttaagat ttacgttatg gttgtattaa aaaaatgttc

cttttttaat tctgcatcga atgacgtaaa aataaagagg ggttgacatt taaataagca

aaatataaac tccaaagttt aagaagtgtg aaaatatgca aacacccagt agtaattcag

aacaaactta atta------ ----gtttaa aactacatgc caataatgcc atcaagaaag

caccatttct attttttaat tttaaaaact tacgt---aa aaccaatttt ttattttatt

ttttaacagt aatcgctaaa ccgatttaaa aaaatttacg atatgaaaga agctaaggaa

tctaatgagc acaaaaaata aaaacaaggc ttttaaaaaa tagtattgtg acgtcatact

tgggacactc tgtataaaaa tattttctta agtccttaaa taactctaca aaatatcaaa

actccaactt gataacgagt tacggaagac ggaggttggc ctagtcca

>148_a_LG8

---------- ---------- ---------- ---------- ---------- ----------

---------- ---------- ---------- ---------- ---------- ----------

---------- ---------- ---------- ---------- ---------- ----------

---------- ---------- ---------- ---------- ---------- ---------a

cactgtttca attttttaat ttttaaaact --------aa aaccgatttt ctgatttttt

ttataatagt aatccctgaa ccgatttaaa aaaatttagg atatgtaaga agctaaggca

tctaatgaac acaaaaaata atagtgtgcc attaaaaaag tattatgttg acggcatatt

taggacactc tgtataaaaa tattttccta agtccttaaa taactctaca aaatatcaaa

actccaactt tataacgagc tgtagaagac ggg------- --------

>0186_LG10

---------- ---ctccgaa agtttaggaa ctgcaatagg gttgtcataa aaaaatattc

gttttttaat tctgcatcga atgatataaa aacaaaaaga ggttgctatt taaataagca

aaattaaagc tctaaatttt aaaaagtttg gaaatatgta aacaccctgt agtaactcag

atcaaactta attagctaaa ctttggtcaa cactacctag taataatgtc actaaaaatg

cacattttcc a--------- ---------- ---------- ---------- ---------t

ttttaacaat aatccttgaa ccgatttaaa aaaattcaag acatgtaa-a aactagggca

tcaaataaac acaaaaaata atagcttgtc attaaaaaaa tatgattacg acgacacact

tggaaaactc tgtataaaaa tacttttctg aatccttaaa taactctaca aaatattaaa

agtccaactt tataacaagc tacagaagac tgaagctggg ctggacca

>0055_LG4

---------- ---------- ---------- ---------- ------ataa aaaaatgttc

gttttttaat tctgcttcga atgatataaa aataaaaaga ggttgccatt taaataagca

aaatataagc tctaaatttt aaaaagtttg gaaatatgta aacatcctgt agtaacttag

atcaaacata attagctaga ccttggacaa cactacctag taataatgtc aacaaaaatc

cactgtttct acttgttaat ttttaaaact acaggtgaaa aaccgatttt ttgttttttt

ttttaacaat aatccctgaa ccgatttaaa agaattcaag acatgtaaaa aactagggca

tctaataaac acaaaaaata atagcttgcc aataaaaaat tatcattatg acatcacact

tgaaatattc tgtataaaaa tatttccttg aatccttaaa taactctaca aaatatcaaa

agatcaactt tataactagc tgcagaagac ggtagctggg ctggacca

>0030_a_LG2

ttttggtaaa taacttcgaa agtttaggat ttgcaatagg gttgtcataa aaaaa--ttc

gttttttaat tctgccttga atgatatgaa aataaagagg ggttgccatt taaataagca

aaataaaagc tctgaatttt aaaaagttta gaaatatgta aacatcctgt agtaactcag

atcaaactta attagctaga ccttgatcaa cactacctag taataatgtc accaaaaatg

cactgtttct attttttaat ttgtaaaact acaggtgtaa aaccgatttt ttgttttatt

ttttaacagt catccctaaa ccaatttaaa aagatttaaa acatgtaaga aactagggca

tctaatgaac acaaaaaata atagcttgcc attaaaaaa- tatgattatg acgtcacact

tggaacacta tgtataaaaa tagttttctg aatccttaaa taactctaca aaacatcaaa

agtccaactt tataatgggc tacagaaaac gcgagctggg ctggacca

>0057_b_LG7

ttttggcaaa tgtgtccaaa agtttaagat ttgcaatagg gttgtcataa aaaaatgttc

gttttttaat tctgcttcga atgatataaa aataaagagg gattgccaat taaataagca

aaa------- ---------t aaaaagttcg gaaatatgta aacaccctgt agtaacttag

atcaatcgaa attgg----- -cttgggcaa caatacctag taataatgtc accaaaaatg

cactgtttct attttttaat ttttaaaact acaggtataa aaccgatttt ttgttttttt

ttttaacagt aattcctgaa ccgatttaaa agaattcaag acatgtaaga aactagggca

tctaatgaac acaaaaaata atagcttgcc attaaatttt tctcattatg acgtcacact

tggaacactc tgtataaaaa tattttcctg aatccttaaa taaatctaca aaatatcaaa

agtccaactt tataacgagc tacagaagac gggagctggg ctggacca

>0005_b_LG3

ttttatcaaa taacttcgaa tctttaggat ttacgttagt gttgtaataa aaaattgttc

gttttttaat --tgcatcga aaggtataaa aataaatagg ggttgccatt taaataagca

aaataaaagc tct--agttt taaaagttag gaaatatgta aacaccctgt agtaattcaa

atcaaactta attggttaga ccttatttaa cactatctag taacaatgtc atcaaaaaac

cactgtttct attttttaat ttttaaaact aaaggtgtaa acagattttt ttgattattt

------cagt aatccctgaa ccgaattaac aagttttagg acatgtaaga aactaaagta

tctaatgagc -caaaaaata atagcatgcc attaaaaaat -attattatg acgtcatatt

tggaacactc tgtataaaaa tatttttctg agtccttaaa taactctaca aaatatcaaa

actccaactt tgtaacgagc tgcaaaagac gggagctggg ttggacca

>0019_b_LG3

ttttatcaaa taactccgaa tctctaggat ttaagttagt gttgtaataa aaagttgttc

gctttttaat tctgcatcga atggtataaa aataaaaaag ggttgccatt taaataaaca

aaataaaagc tctaaagttt taaaagtttg gaaatatata aacaccctgt agtaatccaa

atcgaactca actaattaga ccttgttgaa cactatctag taagaatgtc atcgaaaaac

cactgtttct attttttgat ttttcaaact aaagatgtaa aacagatttt ttgttttttt

ttttaacatt aaccccagaa ccgatttaac aagttttagg acatgtaaga agctaatgt-

---------- ----aaaata atattatgcc attaaaaaaa tattattatg gcgtcatact

tgggacactc tgtataaaaa tattttcctg agtccttaaa taactttaga aaatatcaaa

---------- -ataacgagc tacagaagac gggagctgag ctggacca

>0017_a_LG3

ttttatcaaa taactccgaa tctttaggat ttaagttagt gttgttataa aaagttgttc

gttttttaat tctgcatcga atgatgaaaa aataaataag ggttaccatt taaataagca

aaataaaagc tca-aagttt tgaaagtttg gaaatatata aacaccctgt agtaattcaa

acagaacata attagttaga ccttgttcaa caccacctag taataatgtc gtcaaaaaac

cactgtatgt atattttaat ttttaaaact aaaggtgtaa aaaagatttt ttattttttt

ttttgacaga aatcaatgaa ccaatttgac aattcttaga acatgtaaga agctaaggta

tctattgagc acaaaaaatg atagcatgcc attgaaaaaa aattattatg acgtcacact

tgggacactc tgtataaaaa tattttcctg agtccttaaa taactctaca aaatatcaaa

actccaactt tgtaacgagc ---------- ---------- --------

>113_b_LG8

ttttatcaaa taactccgaa tctttaggat ttacgttagt gttctaataa acagttgttc

gtttttcaat tctgcatcga atgatataaa aataaataag agttaccatt taaataagtg

aaataaaagc actaaacttt taaaagtttg gaaatacata aataccgtgt agtaattcaa

atcgaactta acta------ ---------- ------actg taataatgtc atcaaaaaac

cactgcttct attttttagt ttttaaaacc aaaggtgtaa aacagatttt ttgttttttt

ttttaacagt aatccctgaa ccgatttaac aagttttagg acatgaaaga agctaagcta

tctaatgagt acaaaaaata atagcatgcc attaaaaaat -attattatg gcgtcatact

tgggacactc tgtataaaaa tgttttcctg agtccttaaa taactctaca ag--------

---------- ---------- ---------- ---------- --------

>0003_b_LG3

---------- ---------- ---------- ---------- ---------- --------tc

gttttttaat tctgcattga atgatttaaa aataaatagg ggttggcatt taaataaaca

aaataaaagc tctgaatttt taaaatttgg aaaaaatgta aacaccctgt agtaatgtaa

atcaaactta atta------ ---tgttcaa cactacctag taa---tatc atcaaaaaac

cactgtttct attttgtaat ttttaaacct aata-cacct attatttttt ttgttttttt

ttttaacaat aattcctaaa tcgatttaac aagttttaag acttgtaaaa aactaaggta

tctaacaagc acaaaaagta ttagcatccg atacaaaaaa gattattatg acgtcatact

tgagacactc tgtatcaaaa tatttttctg agtccttaaa taactctaca aaatatcaaa

actccaactt tgtaacgaga tacagaaaac gggagctggg ctggacca

>0013_a_LG2

ttttatcaaa taactccgaa tttctaggat ttacgttgga gttgtaataa aaagttgttt

gttttttaat tctgcactga atgatttaaa aataaatagg ggttggcatt taaataagca

aaataatagc tgtaaatttt taaaagtttg gaaatatgta aacaccctgt agtaattcaa

atcaaactta atta------ ---cgttcaa cactacctag tattaaagtc atcaaaaacc

cactcttttt actttgtaat ttttaaacct aataatgtaa aaaaattttt ttgttttttt

ttttaacaat agttcttaaa ccgatttaac aagttttaga acatgaaaga agctaaagta

tctaacgagc acaaaaagga atagcatgct atacaaaaag gattattatg acgtcatact

tgagactttt tgtatcaaaa tatttttctg agtccttaaa taactctaca aaatattaaa

actccaactt tgtaacgaga tacagaaaac gggagctagg ctggaccc

>0083_LG3

---------- ---------- ---------- ---------- ---------- ----------

---------- ---------- ---------- aataaatacg ggttgccact taagtaagta

aaataagagc tctacagttt taaaagtttg gaaatattta accaccctgt attaattcaa

atcaaactta attagttaga cctagtt--- -----ccgag taataatgtc atcaaaaaac

cactgtttct attttttaac ttttaaacct aaaggtgtaa aacagatttt ttgttttttg

ttttaacaat aattcctgaa cctatttaat aaattttagg acatgtaaga agctaaggta

tctaatgagc acaaaaacta atagcatgcc attaaaaga- tattattatg acgtcatact

tgggatactc tgtatcaaaa tatttttctg agtccttaca taactctaca aaatatcaaa

attccaactt tgtaacgagc tctagaaaac gggagctgag ctagacca

>0160_LG3

---------- ---------- ---------- ---------- ---------- ----------

---------- ---------- ---------- ---------- ---------- ----------

---------- ---------- ---------- ---------- ---------- ----------

---------- ---------- ---------- ---------- ---------- -----gaaag

caccgtttct tttttttaac ttttaaagct gaagacgtaa aactattttt ttggtttttt

ttttaacagt agtt------ -------aaa aaaatttagg acatgtaaaa acctaaggca

tccaatgagc acaaaaaaca atagcatgcc attaaaaaaa tattatgtcg acgtcatact

tgggacactc tgtgtaaaaa tattttcctg aatccttaaa taattctaca aagtatcaaa

agtccaactt tataacgagc tacagaagac gggggctgag ctgggcca

>0129_LG6

---------- ---------- ---------- ---------- ---------- ----------

---------- ---------- ---------- ---------- ---------- ----------

---------- ---------- ---------- ---------- ---------- ----------

---aaactta aatagttaga ccttgttcaa cactacgtac taataatgtc attaagaaag

caccattttt gttttttaat ttttaaaact aaaaatatat aatcgttttt tttgtttttt

ttttgacagt aattcctaaa ccggttaaaa aaaa-ttagg acacgtaaga agctaagttt

tctaatgagc acaaaaaatg atagcaagac actaaaaaaa aattatgttg acgtcatact

tgggacactc tgtataaaaa tatttttctg agtccttaaa taattttaaa aaatatcaaa

cgtccaactt tataacgagc tacagaagtc aggaattgag ttgggtca

>0071_LG6

---------- --actcggaa tgtttaaaat ttacgttagg gttgtaaaaa aaaaacgttc

attttctaat tcctcatcga gtggtataaa aataacagg- ---------- ----------

-agttgaaac gctaaaattt aataagtttg aaaatatgta aacaccctat agtaattcag

atcaaactca attagttaga ctttgttcaa gactacgtag taataatctc atcaagaaag

cactgttttt attttttaat ttttaaaact gaaaatgtaa aatcaatttt ttgatttttt

ttttaaaagt aattcctgaa ccgacttaaa aaagtttaag acatgtaaga gactaaaaca

tctaatgagc acaaaaaata atagcacgcc tttaaaaaaa tattatgttg acgtcatact

aggtacactc tgtatacaaa tatttttcta agtccttaaa taactctacc aaatataaaa

agtccaactt tataaggagc tacagaaaac gggagcttag ttgggcca

>0133_LG8

---------- ---------- ---------- ---------- ---------- ----------

---------- ---------- ---------- ---------- ---------- ----------

---------- ---------- ---------- ---------- ---------- ----------

---------- ---------- ---------- -------tag taataatatc atcaaaaata

cgttgcttct attttttaat tttcaaaact aaaaatgtaa aaccgatttt ttgttttttt

tctctaaagt aatccctgaa ccgatttaaa aaaatctaaa acatgtaaaa agttaaggta

tctaatgagc acaaaaaata atagcatgcc attaaaaaaa ttt------g acgtcac--t

ttggacactc tgtataaaaa tattttcctg agtccttaga taactttaca aaatatcaaa

actccatctt cataaagagc tacagaaaac gggagctgag ctgggcca

>0134_LG2

---------- ---------- ---------- ---------- ---------- ----------

---------- ---------- ---------- ---------- ---------- ----------

---------- ---------- ---------- ---------- ---------- ----------

---------- ---------- ---------- -------tag taataatatc atcaaaaata

cgttgcttct attttttaat tttcaaaact aaaaatgtaa aaccgatttt ttgttttttt

tctctaaagt aatccctgaa ccgatttaaa aaaatctaaa acatgtaaaa agttaaggta

tctaatgagc acaaaaaata atagcatgcc attaaaaaaa ttt------g acgtcac--t

ttggacactc tgtataaaaa tattttcctg agtccttaga taactttaca aaatatcaaa

actccatctt cataaagagc tacagaaaac gggagctgag ctgggcca

>0073_LG3

---------- ---------- ---------- ---------- ---------- aaaaaagttt

gttttctaat tccttatcta atggtgtaaa aataaacagg agttgccatt taaatgagca

aaataaaacc tctaaagttt aagatgtttg acaatatgta aacaccctgt agtaattcag

atcaaactta attagttaaa ccttgttcaa gactacgtat taatgatgtc atcaagaaag

caccagttct --------at ttttaaaatt agaagtgtaa aaccgatttt ttttgtttat

ttttaacagt aattcctaaa ccgatttaaa aaaatttagg acttgttaga agctaaagta

tttaatgagc acaaaaaata atagcatgtc atttaaaaaa tgttattatg acgtcatac-

ttggacactc tgtataaaaa tattttcctg agtccttaaa taactctaca aaatatcata

agtccaactt cataacgagc tacagaagac gggagttgag caggtcca

>0082_LG2

---------- ---------- ---------- ---------- ---------- -----cgttc

attttctaat tctctatcga atggtatgaa aataaatagg ggttgtcatt taaataagca

aaacaaaagc tctaaatttt aaaaagttcg aaaatat-tt aataccttat actaattcag

atcaaactca attagttaaa ccttgttcaa aactacgtac taataatgtc atcaagaaag

caccgtttc- -tttttttat ttttaaaact aaaggtgtaa aaccggtttt ta-------t

ttttaacagt aatacttgaa acaatttaaa aagatttagg acatgtaata agctgaggca

tcaaatgagc acaaaaaata atagcatacc attaaaaaa- ---tattatg acgtcatact

tggaacactt tgtataaaaa tatttgcctg agtcctaaaa taactctaca aaatatcaaa

agtccaactt tattacgagc aacagaagat gggagttgag ctgggcca

>0061_LG3

ttttggtaaa taacggcgaa tgtttaggat ttacattagg gttgtaataa aaa---gttc

cttttttaat tccacattga atgacataaa aatgaaaagg ggttgtcatt taaataatca

aaataaaagc tccaaagttt aaaaagtttg aaaatatgtg aacagcctgt agtaattcag

atcaaactta attagttaga tcttgttcaa agctacgtac taataatgtc atcaagtaag

caccgtttct attttttaat ttttaaaact aaaggtgtaa aaacgacttt ttgttttttt

tttt--aagt aatccctgaa ccaatgtgaa aaaatttagg acgtgtaaga tgctaaggta

tccagtgagc actaaaaaca atagcatgcc atgaaaaaa- ---tattttt acgtcgtact

tggaacactc tgcataacaa tattttcctg aaaccttaca taactctcaa aaatatcaaa

agtccaactt tataacgagc tacagaag-- ---------- --------

>0109_LG10

ttttagcaaa taacacggaa ttttt---gt ttatgttagg gttgttataa aaaaaccctc

aaattctaat tccccatcaa atggtataaa aataatctgg ggttgccatt taattaagta

aaataaaagc tctaaaattt aagaagtttg aaaatatgta tacaccctgt agtagttcag

atcaaactta attagttaga ccttgttcaa gactacatac taataatgtc atcaagaaaa

caccgtttct tttttataat ttttaaaatt aaatgtgtaa aaccattttt ttgttttt-t

ttttaacagt aatctttgaa cctatt-aaa aaaatttaga acatgtaaaa agctaaaaca

tgtaatgagc acaaaaaata ctagcatgct attatttttt tattattatt aactcatact

tgggacactt tgtataaaaa tatttccctg a--------- ---------- ----------

---------- ---------- ---------- ---------- --------

>0162_LG3

---------- ---------- ---------- ---------- ---------- ----------

---------- ---------- ---ttataaa aataaacagg gattgtcatt taaacatgca

aaataaaagc tctaaagttg aaggagtttg aaaatatgta aacaccctgt agtaattcag

atcaaactta attagttaga ccttgttcaa gaccacgtgc tagtaatgtc atcaagaaac

caccgtttct attttttaat ttttaaaact aaagttgtaa aaccgatttt ttgttttatt

tttttaaagc aatgcctgaa ccgat--aaa aaaatttagg acatgtacaa agctaag---

---------- ---------- ---------- ---------- ---------- ----------

---------- ---------- ---------- ---------- ---------- ----------

---------- ---------- ---------- ---------- --------

>0043_LG5

---------- ---------- ---------- --------gg gttgtaataa aaaaacgttc

attttccaat tccccataga attgtttaaa aataaacagg atttgtcatt taaataagca

aaataaaagc tctaaagttt aaaatgtttg aaaatatgta aacaccctgt agtaattcag

atcaaactta attagttaga ccttgttcaa gactacgtac taatagtgtc atcaggaaag

caccgtttct atttttgaat ttttaaaact aaa--tgtaa aaccgatttt ttgttttttt

ttttaccagt aatccctgaa ccgatt-aaa aaaactttag acatgtaaga agctagggca

tctaataagc actaaaaata atagcatgcc attaaaaaaa tattaaaatg acgtcatact

tgagacattt tgtataaaaa tattttcctg attccttaaa taactttaca aagtatcaaa

agtccaactt tataaccggc tacaaaagac gggggctgag ctga----

>0021_LG3

ttttatcaaa taactcggaa tgtttaggat ctacgttagg gtcgtaataa aaaaatgtac

attttctaat tccccatgga atggtataaa aataaacagg ggttgtcatt taaataagca

aaataaaagc tctaaagttt aagaagtttg aaaatatgta aacaccctgt agtaatttag

cttaaactca attcgttaga ccttgttcaa gactacgtac taataatgtt gttaaagaag

caccgtatat attttttaat tattaaaaca aaaaatgtag gaccgttttt ttgtattttt

tttttatagt atgccctaaa ccgattagaa aaaatttaga acatgtaaga agctaaggga

tccaatgagc acaaaaaatt attgcatgcc actataaaaa tattattatg aagtcatact

tgggacactc tgtgtaaaaa tattttcctg agtctttaaa taactctaca aattatcaaa

agtccaattt tataacgagg tacagaagac gagacctgag gtaggcca

>0036_LG8

---ttttaaa taactccgaa tgtttaggtt ttatgtaaat gttgtaataa aaaaacgttc

attttctaat tccctatcga acggtct-aa aataaacaag gattaccatt ttaataagca

aaataaaagc tctaaagttt aaaaagtttg gaagtatgta aacaccctgt agtaattcag

atcaaactta attagttaga ccttattcaa cactacctag taatattgtc atcaaaaaaa

cgttgtatct attttttaat ttttaaatct aaagatgtaa aaccgattct ttgttttttt

tttt--aagt aatccctgaa ccgatttaaa aaaatttagg acatgtaaga agctaaggta

tctaatgagc ataaaaaata atagcatgct ttaaaaaaaa tgttatgttg acataatact

tgggacactc tgcataaaaa tactttcttg agtcctaaaa taactctaca aaatatcaga

agtccaaatt tataatgagc tacagaatac aggagatgag ctggacca

>0070_b_LG5

---------- ---------- ---------- ---------- ---------- ----------

---------- --------ga atgatacaaa aattaacagg ggttgccatt taaataagca

aaataaaagc tctacagttt tagaagtttg aaaataagta aacaccctgt agtaattcag

attaaactta attaattaaa tcttgttcaa cactacctgg taataatgtt atcaaaaaaa

aattgcttcc attttttaat ttttaaaact aaagatgtat aaccgatttt ttgttttttt

ttttaacagt aatccctcca ctgaattaaa acaacttaga acatgtaaga acctaaggca

tctaatgagt acaaaaaatt ctagcatgtc tttaaaaaaa tattattagg acgtcatact

ggagacactc tgtataaaaa tattttcctg agtccttaaa taactctata aaatatcaaa

actcc----- ---------- ---------- ---------- --------

>0176_LG3

tttttacaaa taactccgaa tgtctaggat ttacgttagg attgtaagga aaaaatgttt

cttttttaat tctgcatcga atgatataaa aatgtacagg ggttgccatt taaacaagg-

---------c tctaaagttt aagaagtttg aaaatatgta aacaccccgt agtaattcaa

atcaaactta attagttaga ---------- --ctacgtac taataatgtt atcaagaaag

caccgtttct attttttaat ttttaaaact aaagttgtaa aacagatttt ttgttttttt

tttt------ ---------- ---------- ---------- ---------- ----------

---------- ---------- ---------- ---------- ---------- ----------

---------- ---------- ---------- ---------- ---------- ----------

---------- ---------- ---------- ---------- --------

>0137_LG6

ttttatcaaa taactcggaa tgtttaagat ttacgttagg gttataataa aaaaacattt

attttttaat tcccaatcga atggtatcaa aatatacagg ggttgccgtt taaataatga

aaataaaa-- ----aagttt aaaaagtttg aaaatatgta aacaccctgt agtaattcag

ataaaactta attagttaga ccttgtttaa gtctaagtac taataatgtc atcaagaaat

caccgtttgt attttttaat ttttaaaagt aaacatggaa aactgatttt ttgttttttt

ttttaactgt aatccctgaa ccgattcaaa aaac------ ---------- ----------

---------- ---------- ---------- ---------- ---------- ----------

---------- ---------- ---------- ---------- ---------- ----------

---------- ---------- ---------- ---------- --------

>0049_a_LG6

ttttagcaaa taactccgaa tgtttaagat ttatattaga gttgtagtaa aaaaacgttc

aatttctaat ttcccatcga atggtgtaaa aataaacacg ggttgccatt taaataagca

aaataaaagc tctaaagttt aaaaaaaatt gaagtatgta aacaccctgt agtagttcag

ttcaaactta attagttaga ccttgctcaa cacttcctag taataatgtc atcaacaaaa

cgttgcttct attttttaat ttttaaaact aagagtgtaa aaccgatttt ttggttttat

ttttttaagt aatccctgaa ccgatttata aaaattttgg tcatgtaaga agctaagttt

tctaatgagc acaaaaaata gtggcatata attaaaaaaa tattattttg acgtcatgct

tgggacactc tgtataaaaa tatttttcgg aatcattaaa taactttaca aattttcaaa

aatccacctt tatgacgagc tactgaaggc gggagccgag atgg-cca

>0042_LG5

ttttagcaaa taactccgaa tgtttagagt ttacgttagg gttcttataa aaaaatgttc

ctttttt-at tctgcatcga acggtataaa aatcaacagg ggttgctatt taaatcagca

aaataaaagc cctaaaattt aagaagtttg aaaatatgta aacaccctgt agtaattcaa

atcaaagtta ataaattaga ccttgttcaa gaccacgtat taataatgtc atcaagaaag

cacagttact atttttcaat ttttaaaact aaaggtgtca aaccgatttt tt------tt

ttttaacagt aatctctgaa tcgatttaaa taaatttagg atatgtaaga agctaaggca

tctaatgagc acaaaaaata aaagtatacc attaaaaaaa tattattatg acgtcatag-

tgggac---a tgtataaaaa tattttcctg agacct---- ---------- ----------

---------- ---------- ---------- ---------- --------

>0105_LG10

ttttggcaaa tatcttcgaa ttgttaggat ttacgatagg gttatgacga aaaaatgtta

cctttttaat tctgcatcga ataatataac aataaacagg ggttgccatt taaacaagca

aaataaacgc tctaaaattt aagaagtttg aaactatgta aacaccctgt agtaattcaa

atcaaactta attggttaga ccttgttcaa gactacgtac taataatgtc atcaggaaaa

ctccgtttct attttttaat ttttaaaact taaggggtaa aaccgatttt ttgttttttt

ctttaacagt aatctatgaa gctatt--aa aaactttaga gcatataaaa aactaaggta

tctaatgagc acaaaaaata atagcatgtc actaaaatag ttttatgat- ----------

---------- ---------- ---------- ---------- ---------- ----------

---------- ---------- ---------- ---------- --------

>0046_LG4

ttttgacaaa taacttcgaa tgttaaggat ttacgttagg gttgtaata- -aaaatattc

cttttttaat tttgcatcga acggtgtaaa aatagatagg gcttgccatt taaataagaa

caataaaagc tctaaatttt aagaagattg aaaatatgta aacaccctgc agtaattcag

atcaaacttg attagttaga tcatgttcaa gac---gtac taataatgtc atcagaaaag

caccgtttct actttttcat ttttaaaact aaaggtgtaa aatcgttttt ttgttttttt

atttaccagt aatctccgaa ccgatt-aaa aaaatttaga acatgtaaga acctaaggca

tttaatgagc attaaaaata atagcacgcc cttaaaaaa- tattattatg atgtcatact

tgggacactc tgtataaaaa tattttcctg agtcctgaaa taactctata aaatatcaaa

agtccaactt t---acgtgg tgcagaagac gggagctgag ctggacca

>0051_LG3

ttttatcaaa taacttcgaa tgtttaggat ttacgttatg gttgttgtta aaatttgttc

gttttttaat tctgcatcaa atgatataaa aataaacagg tgttgttatt taaataagca

aaataaaaa- -ctgaaattt aaaaagtttg aaaatatgaa aacaccctgt agtagtttaa

attaaactta actagttaga ccttgttcaa cactacctag taataatgtc attaaaaaag

cactatttct attttttaat tttttaaact aaagatataa aactgttttt ttgttttatt

ttttaacagt aatccctgaa ctgatttaaa aaaaattacg acatgtaaga agctaaggta

tctaatgagc acaaaaaata atagcatgcc attaaaaaaa tattattaag acgtcattct

tgggacactt ggtataaaaa tattttccta agtctttaaa taactccaca tactattaaa

acatcaactt gataacgagc tacagaagac gggagctg-- --------

>0023_LG3

tttttgtaaa taactccgaa t-tttaaaat ttacgttagg gttgtaataa aaaaatgttc

gcttcttaat tctgcatcaa atgacataaa aatacatagg ggttgccatt taaataagca

aaataatagc tctgaaattt aaaaagttta attcaa---- -------tgt agtaattcaa

atcaaactta cttaattaaa ccttatttaa caccaaatag taataacgtc atcaaaaaag

cactgttcct attttttgat ttttaaaacc aaaagtgtaa aaccgatttt ttggttttat

ttttaacagt aatccctaaa ccgatttgaa aaaaattagt acatct---- ----------

---aatgagc acaaaaaatc gtagcatgcc attcaaaaaa gattattatc acgtcatact

tgaaacactc tttataaaaa tattttcctg agtcctaaaa taactctaca aaatatcaaa

attccaactt tatacccagt tacagaagac aggagctgga ct-gacca

>0003_a_LG3

tttttgtaaa taactccgaa tgtttagaat ttacgttagt gttgtaataa aaaaatgttc

gttttttaat tctgcatcga ctgatataaa aacacacagg tgttgtcatt taaataagca

atataaaagc tctaaaattt aaaaagtttg ggaatatgtg aacacgcggt agtaattcaa

atcaaactta cttagttaaa ccttgttcaa cactacatag taatatcgtc -tcaaaaaag

cactgttttt actttttgat gtttaaagct aaaggtataa aactgatttt ttggtttttt

tttgaacagt aattccagaa ccgaattaaa aaaattttgg acatgtaaga agctaaggta

tctaatgacc acaaaaaata atagcatgcc gtacaaaaaa tattattatg acgtca---t

tgagacactc tgtataaaaa tattttccta agttcttaaa taactttaca aaatatcaaa

agtctaactt gataacgagc tatagaagac gggagctggg ctggacca

>0013_b_LG2

tttttgtaaa taacttcgaa tgtttagaat ttacgttagg gttgtaataa aaaaatgttc

gttttttaat tttgcatcgg atgatataaa aacacacagg ggttgtcatt taaataagca

aaataaaagc tgtaaaattt aaaaagtttg ggaatatgtg aacatcctgt agtaattcaa

atcaaactta cttagttaaa ccttgtttaa cactacgtag taatatcatc gtcaaaaaag

cactgatttt actttt---- ---------t aaaggtgtaa aaccgatttt tttatttctt

tttcaacagt aattccagaa ccgaataaaa aaaatttagg acatgtaaga agctaaggta

tctaatgacc acaaaaaata atagcatgct gtacaaaaaa tattattatg acgtc----t

tgagccactc tgtataaaaa tattttcctg agttttaaaa taactctaca aaatatcaaa

agtctaactt ---------- ---------- ---------- --------

>0009_LG3

tttttgtaaa taactccgaa tgtttagaat ttaggttagg gttgtaataa aaaaatattc

gttttgtaat tgtgcgtcga atgatttaaa aatacatagg ggttaccatt taaataagca

aaataaatac tctaaagttt aagaagtttg gaaatatgta aacactctgt tgtaattcaa

atcaaactta cttagctaaa ccttgtttaa cactacgtag taataacgtc gtcaaaaaag

cactgtttct atttttttat ttttaaaatt aaaagtgtaa aacaatattt ttgctttttt

ttttaacagt aatccctaaa ttaatttaaa taaaattagg acatgtaaga tgctaaggta

tttaatgaac acaaaaaata atagcatgcc gttcaaaaaa gattattatg acgtcatatt

tgagacactc tgtataagaa aatttttcta aatctttaaa taactgtacg aaatattaaa

act-caattt tataacgagc tg-------- ---------- --------

>0008_LG3

tttttgaaaa taactccgaa tgtttaaaat ttacgttagg gttgtaataa aaaaatgttc

gttttttaat tctgcatcaa acgttataaa aatacacagg ggttgccatt taaataagca

aaataaaagc actaaaattt aaaaagtttg gaaatatgta aacaccctgt agtaatctaa

atcaaactta cttagttaaa ccttgtttaa cactgcatag taataatgtc gtcaaaaaa-

cactgtttct attttttgat ttttaaaact aaaagtgtaa agccgatttt ttggtttttt

ttttaacagt aatccctgaa ccgatttaaa ataatttaag acatgtaaga aactaaggca

c--------- --aaaaaata atagcatgcc attcaaaaaa gatcatgatg acgtcatact

tgagacactc tga------- ---------- ---------- ---------- ----------

---------- ---------- ---------- ---------- --------

>0019_a_LG3

tttttgtaaa taactccgaa tgtttaaaat ttacgttagg gttgtaataa aaaaatgttc

gttttttaat tctgcatcaa acgttataaa aatacacagg ggttgccatt taaataagca

aaataaaagc attaagattt aaaaaaattg gaaatatgta aacaccctgt agtaatccaa

gtcaaactta cttagttaaa ccttgtttaa caccacatag taataacgtc gtcaaaaaag

cactgtttct attttttgat ttttaaaact aaaggtgaca ggt---tttt ttgttatttt

ttttaacagt aatccctgaa ctgatttaaa aatatttagt acatgtaag- ----------

---------- ---------- ---------- ---------- ---------- ----------

---------- ---------- ---------- ---------- ---------- ----------

---------- ---------- ---------- ---------- --------

>0017_b_LG3

tttttgtaaa taactccgaa tgtttaaaat ttacattagg gttgtaataa aaaattgttc

gttttttaat tctgcatcaa acgttataaa aatacacagg ggttgccatt taaataagca

aaataaaagc actaaaaatt aaaaagtttg gaaatatgta aacaccctgt agtaatccaa

gtcaaactta cttagttaaa ccttgtttaa cactacatag taataacgtc gttaaaaaag

cactgtttct attttctgat ttttaaaact aaaggtgtaa aaccgatttt ttggtttttt

ttttaactgt attccctgaa ccgat----a aaaatttgga acatgtaata agctaaggta

tcttatgagc ac-------- ---------- ---------- ---------- --gtcatact

tgagacactc tgtataaaaa tattttcctg agtccttaaa caactctgca aaatatcaaa

actccaactt tataacgagc tacagaagac gggagctggg cgggagca

>0001_LG10

tttttgtgaa taactccgaa tgtttaaaat atacgttagg gttgtaataa caaaatgttc

gtttttcaat tcttcatcga atgatataaa aatacacagg ggttgccatt taaataaaca

aaataaaggc actaaaattt aaaaagtttg gaaatatgta aacactctgt agtaattcaa

atctaactaa cttagttaaa cctaatttaa cactacatag taataacgtc gtcaaaaaag

cactgtttct attttttgat tcttaaaact aaaggtgtaa aaccgttttt ttgttttttt

ttttaacagt aatccctgaa ccgaattaaa aaaaaatagg acatgtaaga agctaaggca

tctaatgagc acaaaaaata atagcatggc attcaaaaaa gattatgatg acgtcatatt

tgagacactc tttataaaaa tattttcctg agtccttaaa taactctaca aaatatcaaa

actccaactt agtaacgagc tacagaacac gggagctggg ctggacca

>0002_LG8

tttttgtaaa ttactcagaa tgcttaaaat ttacgttagg attgtaataa aaaaatgttc

gttttttaat tctgcatcga atgatataaa aatacacaga agttgccatt taaataagca

aaataaaagc tctaaagttt aaaaagtttg gaaatatgta aacatcctgt agtaacacag

atcaaactta cttagttaaa ccttgtttaa cactacgtaa caataacgtt gccaaaaaag

cactgtttcc attttttggt ttttaaatcc aaaagtgtat aaccaatttt ttgttttttt

ttataacagt aatccctgaa ctattttaaa aaaatttagg atatgtaaga agctaaagtg

c-taatgagc acaaaaaata atagcacgca attcaaaaaa gattattatg agacaattt-

---gagacac tgtataaaaa tattttcctg aatcctcaaa taactctgca aaatattaaa

acaccaactt tataacgagc tacgggagac gggagctggg ctgga-ca

>0007_LG6

tttttgtaaa taactccaaa tgtttaggat ttacgtaagg gttgtgataa aaaaatgttc

gttttttaat tctgcattaa acgatataaa aatacacagg ggttgccact tacataagc-

-aataaaagc tctaaagttt aaaaatgttt ggaatatgtg aacaccctgt agtaactcaa

atcaaactta cttagttaaa ccttgtttaa caccacgtag caataacgtc gacaaaaaag

cactgttt-t attttttgat ttttaaaact aaaggtgtaa aaccgatttt ttgttttttt

tttcaacagt aattcgagaa ccgaactaaa aaaatgtagg acatgtaaga agctatggta

tctaatgacc acaaaaaata atagcatgcc gtacaaaaaa aataattatg acgtcacact

tgatacactc tgtataggaa tattttccta agatcttaaa taactctaca aaatatcaaa

agcccaac-- ------gagc tacagaagac gggaactggg ctggacca

>0078_LG6

tttttgtaaa taactccgaa tatttagaat ttatgttagg gttgttataa taaaatgttc

gctttttaat tctgcatcga ataatataaa aatacacagg gattgccatt taaataaaca

aaataaaagc tccaaaattt agatagtttg gaaatatgta aacatcctgc agtaattcaa

atcaaactta cttagttaaa ccttgtttaa cactacgtag taataacgtc gtcaaaaaag

tacagttttt attttttaat ttttaaaact aaagatgtaa aaccat---- --------tt

tttcaacagt aatcactaaa ccaatttaaa aaaaattggg acatgtaaga agctaaggta

tctaatgacc acaaaaaata atagcatgcc attcaaaaaa tattattatg acgtcat---

------acac tgtataaaaa tattttccta agtcctta-- ---------- ----------

---------- ---------- ---------- ---------- --------

>0163_LG5

---------- ---------- ---------- ---------- ---------- ----------

---------- ---------- ---------- ---------- ---------- ----------

---------- ---------- -----ttttg gaaatatgta aacaccctgt agcaattcaa

gttaaactta cttagttaaa ccttctttaa cactacgtag taataatgtt ggcaaaaaag

cattgtttct attttttgat ttttaaaagt aaatgtgtaa aaccgttttt tt-ttgttta

ttttaacagt aatctctaaa ccgattaaaa attatttagg acatgtaaga agctaaggta

tcgaatgagc acaaaaaata atgacatgtc attcaaaaaa gattattatg acgtcatact

tgagccactc tgtataaaaa t--------- ---------- ---------- ----------

---------- ---------- ---------- ---------- --------

>0014_LG4

tatttgttaa taattctgaa tgtttagaat ttacgatatg gttgtaatta aaaactgttc

gttttttaat tctggatcga gtgatataaa aatacacagg ggttgccatt taaataagca

aaataaaagc tctaagatta aaaaaaattg gaaatatgta atcaccctgt agtaattcaa

atcaaactta cttagttaat tcttgtttaa cactacgtag taataacgtc gtcaaaaaag

cattgtttct attttctgat ttttaaaact aaagatgtaa aaccaatttt tt------gg

ttttaacagt aatccctaaa ccgatttaaa aaaatttaga acatgtaaga agttaaggtt

tctaagcac- -aaaaaaata atagcatgcc gttcaaaaaa gattattatg acgccatact

tgaaacactt tgtataaaaa tatttttctg ggtccttaaa taactctaca aaa-------

---------- ---------- ---------- ---------- --------

>0141_LG3

ttctggcaaa tatctccgaa tatttaggat ctacgttaag gttgtaataa aacaatgttc

gatttttaat tctgaatgga ctggtataaa aataaacagg ggttaccatt taaataagca

aaataaaagc tttaaaagtt aaaaaaaatt gaaatatgta aacaccctgc agtagttcaa

atcaaagtca cttagttaga ccttgtgtaa caccatgtag tagctatgtc ttcaaaaaag

cacggtttct attttttaat ttttaaaact a--------- ---------- ----------

---------- ---------- ---------- ---------- ---------- ----------

---------- ---------- ---------- ---------- ---------- ----------

---------- ---------- ---------- ---------- ---------- ----------

---------- ---------- ---------- ---------- --------

>0005_a_LG3

ttttttgtaa taactcccaa tctttaaaat ttacgttagg tttgttataa aaaaatgctc

gcattttaat tctgcattaa atggtataaa aatacatagg ggttgccatt taaataagca

aaatagaagc tctgaaattt aaaaagtttg gaaatatgta aacaccctgt agtaattcaa

atcaaactta cttagttaaa ccttttttaa caccacatag taataacggc gtcaaaaaag

cactgttcct attttttgtt tttttaaatt aaatgtgtaa aaccgttttt ttgttttttt

ttttaacagt gattactaaa ccgattttaa aaa---tagt acatgtaaga agctaaggca

tctaatgagc acaaaaaatc atagcatgtc attcaaaaaa gattattatt acgtcatact

t--gacactc tttataaaaa tattttcctg agtccttaaa taactctaca aaatatcaaa

attccaactt tataacgag- --cagaagac gggagctggg ctgaacca

>0010_LG9

ttttggcaaa taactccaaa tgtttaggat ttacgttaag gttggaaaaa gaaaatgttc

gatttttaat tctgcatcga acagtgtaaa aataaacagg agttgccatt taaataagca

aaataaaagt ttaacaatta aaaaaaattg gaaatatgta aacaccttgt agtaatttg-

---aaagtca cttaacttaa tcttgtttaa cactatgtag taataatgtc ttcaaaaaaa

cattgtttcc attttttaac ttttaatacc aaaggtgtaa aacaga--tt ttggtttttt

ttttaaacct aattcttaat ccgatttaaa aacatttagg acatgtaaga agctaaggtt

tctaatgagc acaaaaaata atagcatgcc attcaaacaa tattattatg acgtcatact

tgggacactc tgtagaaaaa taatttcgtg agcccttaaa taactatgca aaatatcaaa

actcccactt tataacgagc tacagaagac gggatctggt ctaaatca

>0084_LG3

ttttgacaaa tcactacaaa tgtttaa--- ---------- --tgtaatag aaaaatgatc

cttttttaat tctgcatcga atgatataaa aatgaacagg ggttgtcgtt taaataagca

aaataaaaat tctaaagttt aggaagtttg aaaatatgta aacaccctgt agtaattcag

attaaactta tttagttaga cctagttcaa gactatatgc aaataatttc atcaagaaag

caccgtttct attttttaat ttttaaaact gaagatgtaa aaccgtttat ttgttttttt

ttttaaaagt aacccctgaa ctgattaaaa aaaatttagg acatgtaaga aggtaaggga

tctaatgagc agaaagtata acagcatgcc attaaaaaat tattattgtg acgtcatact

tgagacactc tgtac----- ---------- ---------- ---------- ----------

---------- ---------- ---------- ---------- --------

>0074_LG3

ttttggcaaa taactccgaa tctttagaat ttacgttagg attgtaatac aaaaacgttc

cttttttaat tctgcatcga acgatat-aa aataaacatg ggttgccatt taaataagca

aaagaaaatt tct-taattt aaaaagtttc aaaatattta aataccctgt agcaattcag

acaaaactta attagctcga ccttgttcaa gactacgtac taaaagtgtc atcaagaaag

caccgttttt attttttaat ttttaaaact aaatttgtaa aatcataatt ttgttttttt

gtttaacagt gaaccctgaa tcgacttaaa aaaatttgga acatgaa-gg agttaaggca

tctaatgaac acgaaaaata atagcatgcc attaaaaaaa tat------- ----------

---------- ---------- ---------- ---------- ---------- ----------

---------- ---------- ---------- ---------- --------

>0098_a_LG7

ttttggcaaa taactccgaa tgtttaagat ttacgttagg gttgtaataa gaaattgttc

cttttttcat tctgcatcga acgatataaa aatgaacag- -gttgccatc taaataagca

aaataaaagc tcgaaagttt aagaagattg aaaatatgta aacaccctgt agcaattcag

gtcaaactta atcactcaga ctgtgttcaa gaccacgtac taatattgtc atcaagaaag

caccgtttct attttttaat ttttaaa--- ---------- ---------- ----------

---------- ---------- ---------- ---------- ---------- ----------

---------- ---------- ---------- ---------- ---------- ----------

---------- ---------- ---------- ---------- ---------- ----------

---------- ---------- ---------- ---------- --------

>0066_a_LG7

ttttggcaaa taactccaaa tgtttaaggt ttacgtaaag ggttgaataa aaaaaagttc

attttctaat tccccatcaa acggtataaa aatatacagg agttgccatt taaataagca

aaataaaagc tttaaaaa-t aaaaaaaatg gaaatttgta aacaccctat agtaattcaa

atcaaagt-- ---------- ---------- ---------- ---caatgtt ttcaaaaaag

cactgtttct attttttaat ttttaaaact aaagatgtaa aaccgacatt tttattttat

tttttacagt aatccctgaa ccgattaaaa aaaatttagg acatggagga agctaaggtt

tctaatgaac acaaaaaata atagcatgcc attaaaaaaa cattattatg acgttattct

tgggacactc tgtataaaaa taatttcctg agtccttaaa taactctaca aaataacaaa

actccaactt t--aacgagc tacacaagac aggagctggg ctggacca

>0015_LG6

tttttgtaaa taactccgaa tgtttagaat ttacgttaga gttgtaataa aaaaatgttc

gtttttcagt tttgcatcga ttgagataaa aatacacagg ggttaccatt taaataagca

aaataaaagc tctaaaagtt taaaagtttg gaaatatgta aactccctgt agtaattcaa

atcaaactta cttagttaaa ccttgtttaa caatacgtag taataacgtc gtcaaaaaag

cacagtttct gtttttaatt tttt--aact aaaggtgtaa aaccga---- --tttttttt

ttttaacagt aatccctaaa ccgaattaaa aaa---tagg acacgtaag- ----aaggta

tttaataagc acagaaaata atagcatgcc attcaaaaac gcttattatg acgttatact

tgaggcagtc agtataaaaa tattttcctg agtccttaaa taactctaca aaatataaaa

actccaactt tataacgagc tacagaagac gggc------ --------

>0028_d_LG3

ttttatcaaa taactcgcaa agtttaagat ttacattaga gtagtaataa aaaaatgatc

gatttttatt tctgcatcga atcgtataaa aataaataaa ggttgccaat tagaaaagta

caataatagc tttaaaaatt aaaaaa-atg gaaatatgta aacaccctgt aataattcag

atcaaagtca gtgagttagg tcttgtttaa cactacgttg taataatgtc ttcattattc

ta----ttcg attttttaat ctctaaaact aaaggtaaaa accccatttt ttgttttttt

tcttaacaat aatccctgat ccgatttgaa aaaatttagg acaagtaaga agctaaggtt

tctaataagc acaaaaaata atggcatgcc attaaaaaaa cattattatg acgtcatact

tgggacactc tgtataaaaa taatttccta agtccttaaa taaccctaca aaatatcaaa

actctaactt tataacgagc tacagaagac gggagctcgg c-------

>0038_b_LG2

ttttggcaaa taactccaaa tgtttagaat ttatattaga gttgtaataa aaaaatgttt

catttttaat tctgcatcga atgatataac aataaatagg gattgctatt taaataagca

tcaaaaaagt tc--aaaatt aaaaaatttt aaaacatgta aacaccctgt agtaattcag

atcaaactta attagttaaa ctttgttcaa cactacttag taataatgta atcaa-aaaa

cgttgctttt ttttcttaat ttttaaacct aaagatgtaa aaccgatttt ttgttttttt

ttttaacaga aagccctgaa ccgacttaaa aaaatttagg acatgtaaga agctgagaca

tctaataagc acaaaatata atagcatgcc attaaaaaat tattattatg acgtcaaact

ggggac--ac tgtataaaaa tgttttccta agtccttaaa caactcttca aaatatcaaa

agtccaactt tataacgagc tgcagaagac ggaagctgaa ctagagca

>0059_LG9

------caaa taactccaaa tatttaggat ttgcaataag gttgtaatac aaaaatgttg

cttttttaat tccgcatcga atgatataaa aatcaacagg ggttgccatt taaataagca

aaataaaagc tttaaaaatt agaaagttta gaaatatata aacaccctgt agtagtttaa

atcaaagtca cttagctaga ctttgtttaa cactacgtag taataatgtc ttcaaaaaag

gactgtttct agtttttaat ttttaaaact aaaggtgtaa aaccgatttt ttggtttatt

ttttaacagt aattcctaaa cccatttaaa aaaattcagg acatataaga agctagtatt

tctaatgagc acagaaaatg gtgtcaggtc attaaaaaaa cattcttatg acggcatact

tagaacactc agtataaaaa taatttcctg agcccttaaa taacactaca aaatattaaa

actccaactt tataacgagc tatagaagac gaaagctggg ctggacca

>0090_LG9

---------- ---------- ---------- ---------- ---------- ----------

---------- ---------- ---------- ---------- ---------- -aaataagca

aaacaaaagc tctaaatttt aaaaagtttg gaagtatgta aaccccctgt agtaattcaa

atcaaactta attagttaga ccttgtttaa cacttcttag caataatgtc atcaa-aaaa

cgttgcttct attttttaat ttttaaaact aaagatgtaa aattgatttt ttggtata--

----atatat atattttaat gtattttaaa aaaatttagg acatgtaaga acat------

--taataagc ataaaaaata atgacatgtc ataaaaaaac tattatcttg acgtcatact

tgagtcactc tgtataaaaa tattttcctg actcctcaaa taactctaca aaatatcaaa

actccaactt tataacgagc tacagaagac ggaagcttag ctgggcca

>0032_LG3

ttttattaaa taactccgaa ggtccaggat ttatgttatc gttatatttg aaagttgatc

gtttcttaat tctgcatcag atgatataaa aataaatagg ggttgccatt caaataagca

aaataaaagc tctaaagttt aaaaagtttg gaaatatgta aacaccctgt agtaattcaa

atcaaactca cttagttaga ccttgcttaa cactacgtag taataatgcc ttcaaaaaag

cactg----- ---ttttaat ttttaaaact aaaggttttt a-----tttt ttggttttat

ttttaacggt aacctctaaa ccgatttaaa aaaatttacg acatgtttga agctaaggta

tctattgagc acacaaataa ataccttgcc attagaaaaa tatgattatg acgtcatact

cgggacactc tatataaaaa taatttccta agttcttaaa tacctctaca aaatgtcaaa

actccaactt tataatgagc tacagtagac gggagctgag ctgg-cca

>0052_LG5

---------- ---------- ---------- ---------- ------ttaa aaaaatgttc

gttgtttaat tctgcatcta acgttataaa aataaacacg ggtcgccact taaataaaca

aaataaaaac tccaaatttt aaaaagtttg gaaatatgta aacaccctgt aataattcag

atcaaactta attagttaga ccttgtttaa cactttgtag tagtaatgtc atcaaaaaaa

cactgtttct attttttaat ttttaaaact aaaggtgtaa aacctatttt tt--tttttt

ttttaagatt agtacctgaa ccgatttaaa aaagtttagg tcgtgtaaga agttaagata

tttaatgagc acaaaaaaga atagtaatcc attgaaaaaa tattattatg acatcgtact

tggggcactc tgtatcaaaa tattttacta agttcttaag taactctgca aaatatcaaa

agtccaactt tataac-agc tacggaagac aggagctgag gtggacca

>0107_LG3

---------- ---------- ---------- ---------- ---------- ----------

---------- -----atcga atggtataaa aataaacagg gtttgcaatg taaataagca

aaataaaagc tttaaaatta aaaaaaaatg aaaatatgta aacaccttgt agtaactcaa

atcaaagtca cttagttaga ccttgtttaa cactacgtgg taataatgtc ttcaaaaaag

cactgtttca a-tttttaat gtttaaaact aaaggtgtaa aatcgttttt ttgttatttt

ttttaacagt aatctttgat ccaatttaaa aaaacttagg acttgtaaga agctaaggtt

tctaattaat acaaaaaata atagcatgcc attaaaatat cattattacg aggtcataat

tgggatattc tgtataaaaa tacttttctg agcccctaa- ---------a aaatatcaac

actccaagtt tataccgaac tacagaagac gggagctggg ctggacca

>0145_a_LG9

---------- ---------- ---------- ---------- ---------- ----------

---------- ---------- ---------- ---------- ---------- ----------

---------- -------ttt aaaaagttcg aaaatatgta aacaccctgt agcaattcat

gtcaaactta attatttaga ccttgttcaa gat-acgtac taataatgtc atcaagaaag

caccgtttct attttttaat ttttaaaact aaaggtggag atccgatttt ttggtttttt

ttttaatagt aatccctgaa ccgattaaaa aaaattcggg acattttaga agttaaggca

tctaatgagc acaaataata acagcatgcc attaaaaatg tatgattatg atgtcatact

tgggacactc tgtataaaaa tatttttcta t--------- --tttctata aaattacaaa

agtccgactt tataacgaac taccgaagac gggagctgag ctgggcca

>0114_LG3

---------- ---------- ---------- ---------- ---------- ----------

---------- ---------- ---------- ---------- -------att taaataaaca

aaataaaagc tctaaagttt aagaagtttg aaaatatata aacactatgt agtaattaac

attaaactta actagttaga ctttgtttaa aactacatac taataatgtc attgagaaaa

taccgtttat attttttaat ttttaaaact aatggtgtaa aactgatttt ttgattttgt

ctttaacagt aatccctaaa ctaatt-aaa aaaactttgg gcatgtaagg agcgacggca

tttaatgagc acaaaaaata atagcaagtt attaaaaa-- tattattatg acgtcatact

tgggacactt tttataaaaa tgttttcttg agttcttaaa taactctaca aaatatcaaa

aatataaatt tataacaagt tacagaagac gagagctgag ctg-----

>0104_LG10

---------- ---------- ---------- ---------- ---------- ----------

---------- ---------- ---------- ---------- ------catt taaaaaagca

aaataaaagc tctaaaattt aaaaagtttg agattatgtt aataccctgt agtaactcag

accacattta attagttagc tcttgttcaa gacaacatac taataatgtt ataaaaaaag

caccgtttct atttcttgat ttttaaaact aaaaatgtaa aaccgatttt ttgatttttt

ttttaacagt aatccctgaa ctaacacaaa aaaattttgg acatgtaaga agctaaggca

tataatgagc tc-----taa atataatgcc attaaaaaaa tattattatg acgtcatact

tgggacactt tgtataa--- ---ttttctg agtcctgaaa caactctaca aaatatcaaa

actccaactt tataacgagc tacagaagac gggagctgag ctgggcca

>0140_LG10

---------- ---------- ---------- ---------- ---------- ----------

---------- ---------- ---------- ---------- ---------- ----------

---------- ------gttt taaaagtttg aaaatatcta aacaccctgt actaattcag

atcaaactta attaattaga tcttgttcaa aactacgtac tactaatatc gtcaagaaag

caccgttact attttttaat ttttaaaact tcaggtgtaa actattattt ttgttttatt

tttt------ -------aaa ctcatt-aaa aacatgtagg acatgtaaaa agctaagaca

tctaatgtgc ataaaaaata ataacacgct attagaaaat taatattata atatcatact

cgagacactc tgtataaaaa tattttccta agtcgttaaa taactttaca aaatatcaaa

agtccaactt cataacgagc tacagaagac gggagctgag ctgtgcca

>0096_a_LG8

---------- ---------- ---------- ---------- ---------- ----------

---------- ---------- ---------- ---------- ---------- ----------

---------- ---aaaattg aaaaagtttg gaagtatgt- aacaccctgt agtaattcag

attatatttg attagtgaaa ccttgttcaa gactacgtac tagaaatgtc atcaaaaaag

caccgtttct attttttaat ctttaaaact aaagatgtaa aacctatttt ttgctttt--

ttttttaagt aattcctgag ccgattaaaa aaaa--taga acatgtaaca atctaaggta

tctcacgagc acaaaatgtg atagtttacc attaaaaaaa taatattatg atgtcatact

tgggacactc tgtataaaaa tatttttcta agtccttaaa taactcaaca aaatatcaaa

agtccaactt cataacgagc tacagaagac gagagttgac ctgggcca

>0096_b_LG8

---------- ---------- ---------- ---------- ---------- ----------

---------- ---------- ---------- ---------- ---------- ----------

---------- ----aaattg aaaaagtttg gaagtatgta aacaccctgt agtaattcag

attatatttg attagtgaga ccttgttcaa gactacgtac taataatgtc atcaaaaaag

caccgttttt attttttaat ttttaaaact gaaggtgtaa aacctatttt ttgttttt--

tttctcaagt aattcctgag tcgattaaaa aaaa-ttaag acatgtaaca atctacggta

tctcacaagc aaaaaaagtg atagtttacc attaaaaaaa taatattatg aagtcatact

tgggacactc tgtataaaaa tattttccta agttcttaaa taactcaaca aaatatcaaa

agtccaactt tctaacgagc tacagaagac ggaagctgag ctggacca

>0172_a_LG10

---------- ---------- ---------- ---------- ---------- ----------

---------- ---------- ---------- ---------- ---------- ----------

---------- ---------- ---------- ---------- ---------- ----------

---------- ---------- ---------- ---------- taatagtgtc atcataaaag

catcgtttct attttttaat tttcgaaatt aaagatatat accgattttt ttatttta-t

ttttaacagt aattcttaaa tcgattaaaa aaat--tata acatattaga agatagagta

tttaacgtgc acaaaaaatt atattacgcc atcaaaaaat tactattatg acgtcatact

tgggacactc tgtaaaaaaa tatttttcta agggcttaaa taatactaca aagtatcaaa

agtccaattt tataatgagt tacagaagtt gggagttgag ctgggcca

>0172_b_LG10

---------- ---------- ---------- ---------- ---------- ----------

---------- ---------- ---------- ---------- ---------- ----------

---------- ---------- ---------- ---------- ---------- ----------

---------- ---------- ---------- ---tagctaa taataacgtc atcaaaaaag

catcgtttct attttttaat tttcgaaatt aaagatacat accgattttt ttatttta-t

ttttaacagt aattcttaaa tcgatttaaa aaaacatagg acatattaga agatagagta

tttaacgggc accaaaaatt atattacgcc atcaaaaaat tactattatg acgtcatact

tgggacactc tgtaaaaaaa tatttttcta agggcttaaa taatactaca aagtatcaaa

agtccaattt tataacgagt tacagaagtt gggagttgag ctgggcca

>0172_c_LG10

---------- ---------- ---------- ---------- ---------- ----------

---------- ---------- ---------- ---------- ---------- ----------

---------- ---------- ---------- ---------- ---------- ----------

---------- ---------- ---------- ---tacctaa taataatgtc atcaaaaatg

catcgtttct attttttaat ttttgaaatt caagatatat accgattttt ttattata-t

ttttaacagt aatttttaaa tcgatcaaaa aaat--tata atatattaga aaatagagta

tttaacgtgc acaaaaaatt atattacgcc atcaaaaaat tactattatg acgtcatact

tgggacactc tgtataaaaa tatttttata agtccttaaa taattctaca aagtatcaaa

agtccaactt tataacgagc tacagaagtt gggagttgag ctgggcca

>0130_LG6

---------- ---------- ---------- ---------- ---------- ----------

---------- ---------- ---------- ---------- ---------- ----------

---------- ---------- ---------- -------ata aacaccctgt agtaattcag

atcaaactta attagttaga ctttgttcaa gactacgtac taataatgtc atcaaaaagg

cacattttct attttttaat ctttaaaac- gtaggtgtta agtcgatatt ttgttttttt

ttttaagaat aattcctaca ccgattaaaa agaattcagt acatgtaaga aactaagtca

tgtaatgaaa acaaaaaata atggcaagac attaaaaaag tattattata acgtcatact

tgaaacactt tgtataagaa tatttttctg agtccttcaa -----ctaca aaatatcaaa

agttcacctt tataacgagc tacagaagac gggagctgag ctgggcca

>0064_LG10

---------- ---------- -------gat ttacgttaga gttgtaataa aaaaatattt

cctctttatt tctgcaacga atgacgtaaa aatgagccgg gattgccatt taaataagca

aaataaaagc tatgaagtt- ---------- ------tgta aacaccctgt actaattcag

atca----ta attagttaga ccttgttcaa gactacgtac taataatttt ataaagaaaa

taccgttttt attttttact tttttaaaca -----t---- ------tttt ttgttttttt

tttcacgagt aatcccagaa cggatttaaa aaaagttagg acatgtaaca aactaagaca

tctaatgagc acaaaaaata ataacatgcc ttgaaaaaaa tattattata acgtcatact

ttgaacactc tgtataataa tattttcctg agtcct---- ----tctaca aaatatcaaa

agtccaactt tataacgact tacaaaagac gggagctaag ctggacca

>0058_LG10

ttttggcaaa taactccaaa tatttaggat ttacgttagg gttgtaa-aa aaaaatgttc

ccttattaat tctgcatcga atgatataaa aatgaacaga ggttgctgtt taaatcaaca

aaataaaagc tttaaagtt- ---aagctta aaaatatgta aatacccggt agttactctg

atcaaa--ca attagttaga ccttgttcaa gactacgtag tatcagtgtc atcacgaaag

caccgtttct attttttaaa tttataaact aaaagtgcaa aaccgatttt ttggtttttt

ttttaacaat agccccagaa cagatttaaa aaaa-atagg gcatgtaaga acctaagtca

tttaatgaac acaaaaaata atagcgtaca tttataaaac tattattatg acgttatact

tgggacactc tgtataaaaa tattttccta aattcttaaa taactctaca aagtatcaaa

agtccaactt tataacgagc tacgaaagac aaaggcagac ctgagcca

>0177_LG3

---------- ---------- ---------- ---------- ---------- ----------

---------- ---------- ---------- ---------- ---------- ----------

---------- ---------- ---------- ---------- ---------- ----------

---------- ---------- ---------- ---------- ------tgtc atcag-aaag

caactcttct agttttaaat tttagaaact taaggtgcaa aaccgatatt ttggttttat

ttataacagt tacccctgaa ccaat--aaa aaaatttagg acatgtaaga agctaaggca

tcaaataagc acaaaaaata atagtatgca attaaaaaac tattattatg acgtcatact

tgagacactc tgtataaaaa tattttcctg agtccttaaa taactctaca aaatatcaaa

agtctaactt tataa----- ---------- ---------- --------

>0062_LG10

-------aaa taactccgaa tgtttaggat ttacgttagg gttgtattaa aaaaatgttc

attttttaat tctgcgtgga aagatataaa aataaagaag ggttgccatt taaaaatgca

aattaaaaac tctaaagttt aaaaagtcta aaaatattta aacaccctgt agtaattcag

atc-aattta attagttaga ccttgttcaa agcttcgtac taataatgtt atcaagaaa-

----gtttct attttttaat ttt--taact aaagtgataa aaccgatttt ttagttttat

ttttattagt attccctgaa tctatttaaa aaaatttaga gcgtgtaaga agctaaggca

tctaataacc aaaaaaacta atagcatgcc attaaaaaat --atattatg acgtcatact

tgggacactc tgtataaaaa tattttcctg aatccttaaa taactctac- ----------

---------- ---------- ---------- ---------- --------

>0063_LG10

------caaa taactccgaa tgtttaggat ttacgttaaa gttgtaataa aaaaatgttc

attttttaat tctacgtgga acgatgtaaa aataaacaaa gattgtcatt taaataagca

aaataaaaac tctaaatttt aagaagtttg aaaatatgta aagaccttgt tgtaattccg

atcaaactta attaattaga ccttgttcaa agcta----- ------cgtc atcaagaaag

cactattgct attttttaat ttttaaaact acaagtataa atcctatttc tcgatttt-t

ttttaacagt aatctctaaa ccgatt-aaa aaaatttaag acatgtaaaa aactaaggca

tctaataagc acaaaaaata ataacaagcc attacaaaaa tattattat- -----atact

tggaacactc tgtatacaaa tatttttctg agtccttaaa taactctaca caaaatcaaa

cgtccaactt tataa----- ---------- ---------- --------

>0038_a_LG2

ttttatctaa taactcggat tttttaagat ttacgttaag gtcgttacaa aaaaatgttt

gttttccaat tctccatcga atggtataaa aataaatggg ggttgccatt taaataagca

aaataaaagc cctaaatttt aaaaagtttg aaaaaattta aacaccctgt agtaatttaa

atcaaactta attggttaaa ccttgtttaa gactaagtac taataatgtc atcaaacaag

caccatttat actttt---- -------act aaaggtgtaa aaccgatttt tagattttat

ttttaacagt aatccctgaa cagtt--aaa aaaatttatg acatgtgaga agtcagggca

ttcaatgagc acaaaaaata acagcatgcc gtaaaaaa-- ----attatt acgatatact

tgggacactc tgtataaaaa tatttttctg aatatttaaa taactctaca aaatatcaaa

agtccaa-tg tatatcgagc tatagaaaac gggagttgag ctagacca

>0068_LG4

ttttggcaaa taactccgaa tatttaggat ttacgtaaag tttgtaata- aaaaatttgt

cttttttaat tctgcgtcga atgatataag aatgaacagg gcttgccatt taaataagca

aaataaaagc tctaaacttt aagaagttta aaaatatgta aacaccctgt agtaattcag

atgaaactta attagtaaga acgtgttcaa cactaccta- -------gta atcaaaaaag

caacgtttct actttttaat ttctaaaacc ttagatgtga aacagatttt ttagtttt--

---------- ------cgaa aagatttaaa aaaacttagg acatgtaaga tgctaaggca

tctaacgaac acaagtaata atagcatgcc attaaaaaaa tattattatg acgtcatacg

tgggacactc tgtataaaaa tatttt-ttg agttctaaat aacttctacg aaatatcaaa

agcccaactt tataacgagc tacagaagac gggagctggg ctggatca

>0093_LG5

---------- ---------- ---------- ---------- ---------- -aaaaaaatg

ttttttttaa tctgcatcga atgatataaa aatacacaat ggttgccatt taaataacca

aaataaaaac cctaaaattt taaaagtttg gaaatatgta aaca-cctgt aataattcaa

atcagactta ctca------ ----gtttaa gacttcgtag taataacgac gccaaaaaag

tactgtttct attttttgat ttttaaaatt --------aa aaccgatttt ttagttttat

tttttacagt aattcctgaa ccgatttgaa aaaa-atata aaatgtaaga aact-aagta

tctaatgagc acaaaaaata atagcatacc attcaaaaaa tattattatg acgtcatact

tgag--acac tgtataaaaa tattttcctg agtccttaaa agactctaca aaaaatca--

actccaactt tataacgagc tacagaagac gagagctgag ctggacca

>0081_LG8

---------- ---------- ---------- ---------- ---------- ----------

---------- tctgcatcga atgaactaaa aatgcacagg cgttgccatc ttaataagca

aaataaaagc tctaaagttt aaaaagtttt gaaatatgt- aacaccctgt agtaattcat

atcaaattta cttagct--- --------aa tactacgtag taatatcgtc gtcaaaaaag

cactgtttct atttttaaat tattaaaaca aaaggcaaaa aaccgatttt ttggttttat

tttttacagt aattccttaa ccaatttaaa aaaaattaga acatgtaagg agctaaggta

tctaataagc ataaaaaata atagcatgcc attcaaaaaa gattactttg acgtcatact

tgagatactc tgtataaaaa tattttcctg agtctttaaa tcactctaaa aaatatcaaa

attccaactt tataaggagc tacagaaaac gggagctggg ctgaacca

>0115_LG8

---------- ---------- ---------- ---------- ---------- ----------

---------- ---------- -------caa aataaacaaa ggttcctatt taaataacca

aaataaaagc tccaaaattt aaaaattttg gaagtatgtg aacatattgt agtaagttag

attaaattta attagctaga ccttgcttaa tactacctag ttaaaatatt accaaaaaag

cattactcgt atgttttaat ttttaaaact aaaggtgtaa aaccgatttt ttgagttttt

ttttaacagt aatccctgga ccaatttaaa aaat------ ccatgtaaaa agctagggta

cctaatgagc ataaaaaata ctagcatgcc attaagaaaa tattattatg atgtcatatt

tgggacatt- --gataaaaa tattttcctg agtcgttaaa aaactctaca aaatattaaa

agtctaattt tataacgagc tacagaagac gggagctgga atggacca

>0120_LG3

---------- ---------- ---------- ---------- ---------- ----------

---------- ---------- ---------- ---------- ------aatt taaataacac

aaataaaagc tccgaaattt aaaaagtttg caagtatgta aacatcctgt agtaattcag

atccaattta aatagttaga ccttactgaa cattgcctag tcataatgtt atcaaaagag

cactgtttgt atttttcaat ttttaaaact -----tttaa aaacgatttt ttgtttttct

ttctaacagt aatcctaaaa tcgatttaaa aaaatttaga acatgtaaga agctagagta

tcaagtgagc acaagaaata atagcatgtc attaaagata tattattata acgtcacact

tgggacactc tgtataaaaa tattttccag agcccctaaa taactctaca aaatatcaaa

acttcaattt tataacaagc tactgaatac tgaatctgaa caggacc-

>0022_LG3

ttttatcaaa taacttggaa tgtttaagat ttacgttagg gttgtaataa aaaaatgttc

attttctaat tccctatcga atgatataaa aatatatagg ggttgcaatt taaataaaca

agataaaagg tctaaagttt acaaagtttg aaaatatgta aacaccctgt agtaattcag

atcaaactta attagttaga ccttgttcaa gactatgtaa taataatgtc ataaagaaag

caccgtttct atttttttct ttttaaaact aaagatgtaa atccgattt- ----------

------aagt aattcctgaa ccgatttaaa aacatttaga acatgtaaga agctagggca

tcaaatgagc acaaaaaata aaagcatgcc ataaaaca-- -attattatt acgtcatact

tgggacactc tgtataaaaa tatttttctg agtcct---- ---------- ----------

---------- ---------- ---------- ---------- --------

>0098_b_LG7

ttttatcaaa taactcgaaa tgtttaagat ttacgttagg gttgtaataa aaaaacgtct

attttctaat tactcatcgt atggtat-aa aataaacaag ggttgccatc taaatgagca

aaa-aaaagc tataaagttt aaaaagattg aaaatatgta aacaccctgt agtaattcag

atcaaactta attaatcaga ccttcttcaa cactacctag taataatgta atgaa-aaaa

cgttgcttct attttttaat ttttaaaact aaaggtgtaa aactgttt-- --------tt

tttttaaagg aatccctgaa ccgatttgaa aaaatttaga acatgtaaga aactaaagca

tt-------- ---------- ---------- ---------- ---tattatg acgtcatact

tgggtcactc tgtataaaaa tattttcttg agtccttaaa taactctaca aaatatcaaa

agtccaagtt tatagcgagc ttcagaagac gggagataag ctgggcca

>0034_LG9

ttttatcaaa taactcggaa tgtctaagat ttacgttacg gttgtaataa aaaaacgtt-

---tattaat tccctatcaa ataatataaa aataaacagg ggttgccact taaataagca

aaataaaagc cctaaagctt aaaaagtttg gaaatatgta aaccccctgt agtaattcat

atcaaactta attagttaga cgttgttcac cactttctag taataatgtc accattatta

atatttttct attttttaat ttctaaaatc aaaggtgtat aaccgacttt ttgttttttt

tttttaaaca agttcctgaa ctgatttaaa aaaaattaga aca-----gt aatgtaagga

tctaatgagc acaaaaaata atagcatacc attaaaaaaa tattatggcg acgtcatact

atagtcactc tgaataaaaa tattttcctg agtccttaaa taactctaca aaatatcaaa

agttcaactc tataacgaac tacagaagac gggagttgag ctagttca

>0044_a_LG9

ttttatcaaa taactccgaa tgtataggat ttacgttagg gcc-taataa aaaaatgttc

gttttctaat tccctatcga atgatataaa aataaacagg ggttgccttt taaataagca

aaataaaagc tctaaaattt aaaaaggttg gaaatatgta aacaccctgt agtaacttag

atcaaactta tttagctaga acatggacaa cactacctag taataatgtc acaaaaaatg

cactgtttct attttttaat ttttaaaact acaggtgaaa aaccgatttt ttattttt-t

ttttaacagt aatccctgaa ccgatttaaa aaaatttaaa acatgt---- ----------

----atgaac aaaaaaaata atagcttgcc attaaaattt ttatcatatg acgtcacact

tggaacattt tgtataaaaa tattatcctg aatccttaaa taactctaca aaatatcaaa

agtccaagtt tataacgagc tacagaagac gggggctggg ctggacca

>0080_b_LG10

ttttatcaaa taactccgaa tttttagaat tttc-ttgag tttttaataa aaagttgttc

gttttttaat tctgcatcga atgatataaa aataaataga ggttgccatt taaataagca

aaataaaaga tctaaaattt taaaactttg gaaatatgta aacaccctgt agtaattcaa

atcaaactta attagtcaga ccttgtttaa cactacctgg taataaaaca ctgtttctgt

tactgtctct attttttaag ttttaaaact aaaggtgtaa aacaga---- ----------

ttacgacagt aatccctaaa ccgatttaac aagttttagg atatgtaaga agttaatgag

tctaatgagg acaaaaaatt atagcatgcc attaaaaaat tattattatg aggtcatact

tgggacactt tgtatcaaaa tattttcctg agtccttaaa taactctaca aaatatcaaa

actccaacat tgaaacgagc tacagaagac gggaacttgg ctggatca

>0033_LG8

ttttggtaaa taactccgaa tgtctaaaat ttacgttagc attgcaataa aaaaatgttc

attttttaat tctatatcga atgatattaa aataaacagg ggttgctatt taaataagca

a--taaaagc tctaaagtt- -aaaagtttg gaaatatgta aacaccctgt agtaattcaa

atcaaactta cttagttaaa ccttgtttaa cacttcgtag taataatgtc gtc--aaaag

cattgtttct attttttaat ttctaaaagt gaaggtgaaa aaccattttt tagatttttt

ttttaagagt aattcctaaa ccgatttaaa a--------- acatgtaaga agctaaggta

tctaatgaac acaaaaaatt ataacatgcc gttcaaaaaa tattattatg acgtcacact

tgagacaagc tgtataaaaa tattttcctg aatccttaaa taactctacg aaatatcaaa

actccaattt tataacgagc tacagaagac ggaagctggg ctgg-cca

>0011_LG9

---------- ---------- -------gat ttacgttatg gttgtattaa aaagttgatc

gttttttaat tttacatcga atgatataaa aataaataag ggttgccatt taaaaaatca

aaatacaagc tctaaatact aaaatgtttg aaaatatgta aacaccctgt agtaattcaa

atcaaactta cttagttaga tcttgtttga cactacgtag tgaaaatgtt tttcaacaag

cactgtttct attttt--at ttttaaaact aaaagtgcaa aaccgatttt ttgttttttt

tcttaacagt aatccttgaa ccgatttaaa aaaa------ acatgtaaga agctaaggta

tctaatgaca acaaaaaata atagcatgca attcaaaaaa cattattatg acgtcatact

tgagacactg tg---aaaaa tattttcctg agtccttaaa tacttctaca aaatatcaaa

actccaactc aataacga-- ---------- ---------- --------

>0102_b_LG3

ttttaataaa taactccgaa tgtttaagat ttacgtt-cc gttggtatga aaaaatgttc

ggtttctcat tcaacatcga atgatgtaca aataaacagg agttcccatt taaaaaatca

aagtaaaagc tccaaaattt aacaagtttg tatatatatg aacaccctgt agttattcag

atcaaatcta attagttagg cc-------- -actacgtaa taataatgtc atcaaaaatg

cattatttct agtttttaat ta-taaagct aaaggtgtaa taccgatttt ttgt----tt

tttttacagt aatcccaaaa ccgatttaaa aaacttaaga acatgtgaga agctaagaca

tctaatgagt caaaaaaatt ataggatgtc tttaaaaaaa tattattatg acgttatact

tgagacattt tgtataaaaa tattttcttg agtccttaaa tatctaacca aaatatcaaa

gttccaactt tataacgagc tacagaagac aggagctggg ttggacca

>0136_LG9

---------- ---------- ---------- ---------- ---------- ----------

----tttaat tctgcatcga atgatataca aataaatacg agttgccatt t-----agca

aaataaaaac tctaaaaatt tgaaagtttg gaaatatgga aacaccctgc agtaattcaa

atcaaactta cttagctaga ccttgtttaa cactacgtag taataatttc ttcaaaaaag

tactgtttct a-tatttaat ttttaaaact aaagatgtat aatcgatttt tagttttatt

ttttaacagt aagtccaaaa ccaatttaaa aaactttaga ---------- ----------

--caatgagc acaaaaaata aaaacatgct attaaaaaat tatgattatg acatcacact

agagacactc tgtagaaaaa tattttctta agtcctcaaa tacctctaca aaatatcaaa

cctctgactt tataatgagc tacagaagac gggagttgag ttggacca

>0085_LG3

---------- ------tgaa tgtttaagat ttgcgttaga gttgtaataa aaacatgttc

attttctaat tccccactga atggtatata aatgaacggg agttgccatt taaataagca

aaataa---- ----aagttt aaaatgtttg taagtatgta aacccccagc agcaattcac

atcaaactta attagttaga ccttgttcaa cactacttag caataaagtc atcaa-aaaa

cgatgcttct atttttaaat tcttaaaact aaagctgtag aaccgatttt tt--------

---------- aaattctgtt ttaatttaaa acaattttga atatgtaaga agctaaggta

tcaaatgaac acaaaaaata atagcatgac aaaaaaaa-- taatatgttg aagtcatatt

tgg---attc tgtataaaaa tatttttctg agtccttaaa taactttact aaatattaaa

agttcaactt tataacgagc tacaaaagac gggagctgag ttgggcca

>0103_LG3

ttttatcaaa taactcggaa tgttcaagat ttacgttaga gttgtaataa aaaaaagttc

attatcaaat tctctatcaa agggtataaa aatatacagg tgtttccaat taaataagca

aaataaaagc tctaaagttt aaaaagtttg gaagtatcta aaccccctgt agtaaatcag

atcaaactta attagttaca ccttgtccaa tactacctag taataatgtt atcaaaaaaa

g-ttgattct aatttttaat tcttaaaact aaaggtgtac aaccgatttc ttgtttcatt

ttttaattgc aaaattt--- --aatttaaa ttgcatttgg acatgtagga agctaaagta

tccaatgaac ataaaaaata attgcctacc attaaaaaaa ---tattatg acgtcatact

tgggacactc tgtataaaaa tatt------ ------taaa taactctaca aaaaatcaaa

agtccaactt tacaacgagc tacagaagag gggagctgag ctgggtca

>0139_LG3

---------- ---------- ---------- ---------- ---------- ----------

---------- ---------- ---------- ---------- ---------- ----------

aaataaaagc tctaaagttt aaaaagtttg aaaatatgta aacaccctgt agtaattcaa

atcaaactta attcgttaga cattgttcaa gactaagtac taataatgtc atcaggagag

cactgtttct aattttaaat ttttaaaact aaagttgtaa caccattttt ttggttttat

ttttaacagt aatccctgaa ctgatttaaa aaaatttaaa acat------ -gctaaagca

tctaatgaac acaaaaaata atagcatgcc attaaaaaaa tattgtcatg acgtcatact

tgggacactc tgtataaaaa tatttacttg agttct---- ---------- ----------

---------- ---------- ---------- ---------- --------

>0108_LG3

---------- ---------- ---------- ---------- ---------- ----------

---------- ---------- ---------- ---------- -----tcatt taaataagca

aaataaaagc tctaaagttt agaaagtgtg gaaataagta aacacctttt agtaattcag

attaaacata aattagtaga tcgtgttcaa aactacttga tactta---c atcaaaaaaa

tgttgcgtct attttttaat ttttaaaact aaaggtgtaa aaccaatatt ttgttttttt

tttt--aagt aaactctgaa tcgatttaaa aaaactttag acatgcaaga agctaaggta

tttaatgagc acaaaaaata atagcattca attaaaataa tattatgttg acgtcatatt

tgggacactc tgtataaaaa tattttcctg aattcttaaa taagatcaca aaatattaaa

agtccaactt tataacgagc tacaga---- ---------- --------

>0190_LG2

---------- ---------- ---------- ---------- ---------- ----------

---------- ------tcga atggtataaa aataaacagg agttgctatt taaataagaa

aaa---aagc tctaaagttt aaaaagtttg gaagaatgta aacctcctgt agaaattcag

atcaaactta attagttaga ccttttttaa cactacctag taaaagtgtc atcaaaaaac

--------gt tgcttctaat ttttaaaact aaaggtgtaa aaccaatttt tt--------

------cagt aatccctgaa tcgatttaaa agaaaattgg acgtgtaaga agctaaggaa

tctaatgagc acaaaaaata atagcatgac attaaaaa-- tattatgtgg acgttatact

cgggacacta tgtattcaaa tattcttcca ---------- ---------- ----------

---------- ---------- ---------- ---------- --------

>0088_LG3

ttttatcaaa taactccaga tgtctaggat ctacgttagg tttgtaataa aacaatgtt-

---ttctaat tctccaccga atgatataaa aatggacatg ggttgttatt taaataagca

aaataaaagt tcaaagtttt acaaagtttg gaaaaaagta aacaccctgt a-caattcag

atcaaagtta actagttaga ccttgtttaa cattacctag taataatgtc atgaa-aaac

gactgtttct attttttaag tttttaaaac taaggtgtaa aaccgatttt ttgttttttt

tcttaacagt aatccttgaa ccaactt--a aaaatcaagg acatgtaaga agctagggga

actgatgaac acaaaaaata atatcatgcc gttaattggt tattattatg acgatatact

tggaacactt tgtataaaaa tatgctcctt aatccttaaa taacttcaca aaatatcaaa

agcccaactt tataaggagc tatagaagac gggagctgtg ctggacca

>0131_LG7

---------- ---------- ---------- ---------- ---------- ----------

---------- -------tga atgatatata aatgaacaag ggttgccatt taaataagca

aaataaaagc cctaaagttt aagaagtt-- ----tatgta aacaccctgt agtaataca-

---aaattta attagttaaa tcttgttcaa cactacgtac taataatgtc at-aaaaaag

caccgtttct attttttaat ttcgaaaact aaaggtttaa aaccgatttt atcttttttt

tttt------ -------taa tagattaaaa a---attgga acatgtaaga agctatggta

tctaacgagc tcaaaaaatg atagcatacc attaaaataa tattataata acgtcatatt

tagaacactc tttataaaaa tattttcctg agtccttaaa taactctaca aaatagcaaa

actccaactt aatcacgtgc cgcagaagac gggaactgtt ctgggcca

>0117_LG3

---------- ---------- ---------- ---------- ---------a aaaaacttac

cttttttaat tctgtatcga ataacataaa aataaacatc gattgccatt taaataagca

aaataaaagc tctaaagttt aaaaagtttg aaaatatgta aacaaccagt agtaattcgc

atcaaa--ca actagttata ccttattcaa gactacatac taaaaatgtc atcaaaaaag

cagagattct acttttaaat ttttaaaact aaaggtggta aaccgatttt ctgttttttt

ttttaacagt gatccatgaa ccgatt-aaa aaaatttaga acatgtaaga agctaaggca

tctaatgagc actaaaaata atagcaagcc attaaaatta ---aaagtta acgtcaaact

tggaa----- ---ataaaaa tatgttcctg agtccttaaa taattctaca aaatattaaa

------actg tataacgagc tacaaaagat aggaactgag ctggacca

>0151_LG3

---------- ---------- ---------- ---------- ---------- ----------

---------- ---------- ---------- ---------- ---------- ----------

---------- ---------- ---------- ------tagt aaacacctgt attaattcag

gtcaaact-- --tagctaga ccttggtcaa ggctacgta- --ctaatgcc atcaagaaag

caccgtt--- -ttttataat ttttaaaact aaaggtataa aactgattta ttggttttat

tttttacagt aatctctgaa ccgattaaaa aaaatttagg acatgtaaga agctaaggca

tctaatgagc acaaaaaata atagcatgcc attcaaaaaa tattttaata acgtcatact

tggt--acac tgtataaaaa tattttcctg agtcctcaaa taactctata aagtatcata

agtccagctt tataacgatc tacagaagac ggaagctaag ctgggcca

>0207_LGX

---------- ---------- ---------- ---------- ---------- ----------

---------- ---------- ---------- ---------- ---------- ----------

---------- ---------- ---------- ---------- ---------- ----------

---------- ---------- ---------- ---------- ---------- ----------

---------- -------gat ttttaaaact aaaggtgtaa aaccgatttt ttgttttttt

tttcaacagt aattccagaa ccgaattaaa aagatttagg acatgtaaga tgctatggta

tctaatgacc accaaaaata atagcatgcc gtacaaaaaa gataattatg acgtcacact

tgatacattc tgtataaaaa tattttcctg agttcttaag taactctaca aaatatcaaa

agcccaactt gataacgagc tacagaagac gggagctggg ctggacca

>0116_LG2

------caaa taactgcaaa tgttagggat ttacgctagt gttgtaaaaa aaaaacgttt

gttttctaat tctacatcga atggtataaa aataaacagg agtt------ -------gcg

aaataaaagc tctaaagttt aaaaagtt-- gaaatatgta aacaccctgt aatagttcag

gttaaactta attaatttca ccttgttcct tgttacctcg taataatgtc atcaaaaaag

cattatttat attttttaat ttttaacact aaaggcgtta aaccgatttt ttgtttttct

ttttaacaga aattcttgaa cctatttaaa aacatttagg acatgtaaga atttaaggta

tctaatgagc acaaaaaata acagcttgcc attgaataaa tgttattatg acattatact

tgggatactc tgtatgcaaa tattttcctt agtccttaaa taactccacg aaatattaaa

agtccaattt tataacaacc tacaga---- ---------- --------

>0135_LG3

---------- ---------- ---------t ttacgttagg tttt-----a aaaaatgttc

gttttttaat tctgcatcaa atgatataaa aatgtacagg ggttgtctct taaataacca

aaataaaagt tctaaagttt aaaaagtttg gaaatatata aacaccctgt agtatttcag

gtcaaaccta attagttaca gcttgttcaa gactatgtag taataatgtc at-aaaaaag

cactgtttct actttttaat ttgtaaatct gaaagtgtaa aaacgatttt ttggtttttt

ttttaagagt aatccctgaa ccaattaaaa aaaa------ --------ca tgctaagcta

ttcaatgagc acaaaaaatc atagcatgcc attaaaaa-- -attattatg acgttatagt

tgagacattc tgtacaaaaa tattatcctg attccg---- ----tctaca aaatatccaa

actccaactt tttaacaagc tataggagac gagagct--- --------

>0167_LG7

---------- ---------- ---------- ---------- ---------- --aaacgttt

gttttctaat tcc---tcga atggt---aa aataaacagg ggttgccatt tcaataaaca

aaataaaagc tctaaaattt aaaaagtttg gaagtatgta aacaccctgt agtaattcag

attaaactta attagttaga ctttgttcaa taccacctag taataatgta ataatagcaa

ctttgtttct attttcaaat ttgtaaaact aaaagtggaa atccgatttt ttgttttttt

tttgaatagt aatccctgaa tcaatttaaa aaaatttagg acatggagga cgctaatgtt

tgtaataaac acaaaaaata attgcatgc- ---------- ---------- ----------

---------- ---------- ---------- ---------- ---------- ----------

---------- ---------- ---------- ---------- --------

>0159__LG8

ttttggcaaa caacttcaaa tgtttaggat ttgcaatagg gttgtgatta aaaaatgttt

gttttttaat tatttatcg- ttggtataaa taaaaacagg ggttgtcatt taaataaaca

aaataaaagc tttaaattt- --aaagtttg gaaatatgta aacaccctct agtaattcaa

atcaaactta attagttaga ccttgtccaa gactacgta- tgataatgtc atcaaaaaaa

cattatttct attttttaat tcataaaact aaaggtgtta aaccgatttt ttggtttttt

ttttaacagt aatcccttaa c--------- ---------- ---------- ----------

---------- ---------- ---------- ---------- ---------- ----------

---------- ---------- ---------- ---------- ---------- ----------

---------- ---------- ---------- ---------- --------

>0125_a_LG9

tcttggcaaa taactcagaa tgtttaggat ttacgctaag gttgtaataa aaatatgttc

gttttttagt tctgcatcga atggtattaa aataaacagg agttgccatt taaataagca

aaataaaagc tttaaaaatt taaaaaaatg gaaatatgta aacaccctgt actaatttaa

atc-----tt attggttaga ccttgtttaa cactacgta- --ataatgtc ttcaaaaaaa

aactttctat -ttttttaat ttttaaaaca aaaggtgtaa taccaaattt ttggtttttt

ttttaacagt aattccaaaa ctgattttaa aaaatttaag acgtgtatga agctaaggtt

tctaatgagt acaaaaaata atagcatgcc attaaaaaaa cattattatg ctgttatact

tgagacactc tgtataaaaa taatttcctg agcctataag caactcta-a aaataccaaa

------actt tataacgagc tacagaagac gggagctggg ctggac-a

>0030_b_LG2

ttttggcata caattccgaa ggtttaggat atgtaatagg gttgtcatac aaaaatgttc

gtttttttat tctgcttcga atgttgtaaa aataaagagg ggttgccgtt taaataaaca

aaataaaaac tctagatttt aaaaagtttg gaaatatgta aacaccctgt ggtaactcaa

atcaaactta attagctaga ccttggttaa cactacctac caataatgtt accaaaaatg

cactgtttct attttttaat ttctaaaact acaggtgaaa aataaatttt tgggtt----

------cagt aatccctgaa ccactttaaa agaatttatg acatgtaaga aactagagca

tctaatggac acaaaaaata ttgccttgcc attaaaattt tatcattata acgttacact

tagaatactc tgtaa----- ---------- ---------- ---------- ----------

---------- ---------- ---------- ---------- --------

>0086_LG4

ttttggcaaa taactcagaa tgttttgaag atgtattaaa aatgtaataa aaacatgttt

gttttttaat tctgtatcga atggtgtaaa aattaacagg ggttgccatt taaataagca

aaatataaac tttaagtatt aaaaaaattg gaaatatgta aacaacctgt agtaattaaa

agcaaagtca cttaattaaa tcttgcttaa cactacgtag taataatttc ttcacaaaag

tactgtttct attttttaat ttttaatcct aaaggtgtaa aaccaatttt ttgttttatt

ttttaccagg aatctttaaa ccaatttaaa aaaatttagg aaatgtaaaa agctaaggtt

tctaatgagc acaaaaaatg atagtatgcc attaaaaaaa tattatgatg gcgtcatact

tggaactctc tatataaaaa ---------- ---------- ---------- ----------

---------- -ataat---- ---------- ---------- --------

>0123_b_LG4

tttttgtaaa taactccgaa tgtttagaat ttacgttagt gttgtaataa aaaaatgttg

tttttttgat tctgcatcga ataatataaa aatgcccaga ggttgtcact taaataagca

aaataaaagt ttaaaaagtg tggaaatttg aaaatttgta aataacctgt agtaattcaa

atcaaactta cttaatcaga ccttatttaa cactgtgtaa taacaacgtc gttaaaaaaa

cactgtttcc attttttgat taataaaacc aaagatgtaa aaccgatttt tt--------

---------- ---------- ---------- ---------- ---------- ----------

---------- ---------- ---------- ---------- ---------- ----------

---------- ---------- ---------- ---------- ---------- ----------

---------- ---------- ---------- ---------- --------

>0091_LG3

ttttagcaaa taactcagaa tattttggat ttgcgataag gttgcaataa aaaaatgttc

gtttttaaat tctgcgtcga atggtataaa aataaatagg ggtcgccatt taaataagca

aaataaaagc tctaaagtta aaaaagtttg g--------- ---------- agtaattcaa

attaaactta cttagttaaa cctggtttaa cactacgtaa taataacgtt gtccaaaaag

cactgtttct attttttaag ttttaaaact aaatgtgtaa aactgatttt ttgttttttt

ttttaacagt aatccctgaa tcgatttaaa aaagtttagg gcatgtaaga agccaatgta

tccaatgagc acaaaaaata atagcatgcc attcaaaaaa gattgttatg acgtcgtact

tgggacactc tgtat----- ---------- -------aaa taactctaca aaatatcaaa

------actt aataacgagc tacagaagac gggagctgcg ctggacca

>0089_LGX

ttttagtgaa taatcctgaa tgtttaggat ttacattggg gttgtaatga aaaaatgttt

gctttctaat tgctcatcga atggtataaa aataaacaag agttcccatt taaataagca

aaataaaagc tctaaaatta aaaaggtttg gaagtatatg aacagctt-t agtaattcaa

acgtactaat taatgttaga ttttgtttaa cactaactag taatactgtt atcaagagag

cactgtttgt a--------- ttttacaact gaagatgtaa aatgaacttt ttgctctttt

ttttaacagt aattcctcaa ccaatttaaa acaattaagg atatgtaaga agctagagta

tctaataatt acaaaaaata atagcatgcc attaaaaaaa ---tagtatg tcgttatact

tgggacactc tgtataaaaa tatttttttg agtccttaaa taactctaca aaatattaaa

agtccgaatt tataacgagc cacagaagac gga------- --------

>0097_LGX

ttttggtgaa taaatccgga tgtttagaat ttacgctggg gttg--atag aaaaatgttc

gctttgttat tctgcatcga atagtgtaaa aataaacaag ggttcccatt taaataagca

aaataaaagc tctaaagtta aaaaagtttc gaagtatgtg aacaacctgt agtaattcag

atcaaattta atgagttaga ccttgtttaa cactatctag taataatgtt atcaagaaag

cactgttgga attttttaat ttttcaaac- agagttgtaa aacgaatttt tagttttttt

ttttagcagt aatccctgac ccgtttg--a attttttagg atatggaagg agctagggta

tccaatgaga acaaaaaata acagcttgcc attgaaaaaa ---------- ---------t

aggtacactg tgtataaaaa t--------- -gactttaaa taactctaca atatatcaaa

agtccaaatt tataacaagc tacagaaaag gggaactggg ttagacca

>0209_LG5

---------- ---------- ---------- ---------- ---------- ----------

---------- ---------- ---------- ---------- ---------- ----------

---------- ---------- ---------- ---------- ---------- ----------

---------- ---------- ---------- ---------- ---------- -tcaaaaaaa

aactgttttt attttttaat ttttgaaact aaagatgtaa actgattatt ttgttttttt

ttttaacagt aatcgttgac ccgatttaca aaaatttagg agatgtaaga agc-aaggca

tctcatgagc acaaaagata atagcatgcc attaaaaaat gattattatg acatcatact

tgggacactc tgtataaaag tattttcttg agttcttaag taat------ ----------

----tgactg aataacgaac tacagaagac gggagctgag ctggacca

>0212_LG3

---------- ---------- ---------- ---------- ---------- ----------

---------- ---------- ---------- ---------- ---------- ----------

---------- ---------- ---------- ---------- --caccctga actaactcaa

atcaaacttg attatttaga tcgtgtttaa tactacctag taataatatt atcaaaggaa

tactgtttgg gctttttaat ttttaaaact aataaaattc aatatcaatt ttcttttttt

tttt--aagt aatccctgaa ccaattt--a aatttttaag atatgtaagt aactagagca

tcgaatgggc ataaaaaata atagcacgcc attaaaaaaa tgtaattgtg acgtcacgct

tgggacactc tgtataaaaa tattttaata agccctcgca taattctaca gaatataaaa

aattcaattt tataaa---- ---------- ---------- --------

>0199_LG7

---------- ---------- ---------- ---------- ---------- ----------

---------- ---------- ---------- ---------- ---------- ----------

---------- ---------- -------ttg aaaataggta acccccctgt agtaattcaa

atcaaattta attagctaga ccttattcaa gactacatac taataatgtc atcaagaaag

caccgtttca ttttta---- ttttaaaact aaagacgtaa aatcgattaa tttttttaat

ttttaacagt aa-cagtaaa ccgatt-aaa aaaatttagg tcatgtaaga agctaaagca

tttaa--agc gcaaaaagta ataccatgct attaaaaaa- ---tattatg acgccatact

tgggacactc cgtataaaaa tattttcctg agtcct---- -------aca aaatatcaaa

aggtcacctt tataacgagc tacagaagac gggagctaag ctggg---

>0153_LG9

---------- ---------- ---------- ---------- ---------- ----------

---------- --------ta atgatgtaaa aataaaaagg aattgctatt tagataagtg

taatgaaagc tcaaaaatta aaataatt-- -aagtatata aacacgctgt aataattcag

atcaaacttc tttagataaa ccttgttcaa caccaccttg taataatgtt atcactgaaa

aactgtttct gtttttaaag ttttaaaact gaaagtgcaa caccgaagtt ttgttttttt

tttt------ aatctccaaa ccaattaaaa aaaatttagg acatgtaata agctaaggta

tttagtaagc acatataata atagcatgct attaaaaaaa ---tattatt atgtcatact

tgggacactc tgtataaaaa tattttcctg agtccttaaa taacactaaa aaatatttaa

actccaactt taaaaggaac tactgaagac ---------- --------

>0138_LG3

ttttggcaaa taactgtgga tgcttagcat atacgtaaag gttgtaataa aaaaattttg

gttttttaat tctgcattga acaat----- ---------- -attgccatt taaataagca

aaataattgc tctaaagtt- ---------- -aaaaatgta aataccctgt agccattcaa

atcaaacttg ataaattaga ccttgttcaa cactacgtag aaataatgtg atcaaaaaag

cactgttttt atttttcaat ttttaaaaca aaaattgtaa aaccgttttt tt------gt

tttttacagt aatttctgaa ccgatttaaa agaattcaga acatgtaaga agctaaggta

tctaatgagc acaaaaaata atgtcatggc attaaaaata tatcattttg tcgtcatact

tggg--acac tgtataacaa tattttcctg agtccttaaa tagctctagg aaatataaca

acaccaactt c--------- ---------- ---------- --------

>0197_LG5

---------- ---------- ---------- ---------- ---------- ----------

---------- ---------- ---------- ---------- ---------- ----------

---------- ---------- ---------- ---------- ---------- ----------

---------- ---------- -------caa cactacccag tactaatgtc gtcaaaaat-

--tactttct attttttaat ttttaaaatt caaggtgtaa aaccgatttt ttgttttttt

ttttttatgt aatttttgac ccgatttaaa gaaa--tagg acatgtagta agctaaggta

tctaatgagc ccaagaaata ctagcttagc attaaaaa-- tattattgtg acataatact

tggggcaagt tgtataaaaa tattttccta aatataaaaa taactctaca aaatattaaa

agtccaactt tataacgagc cacagaagac ggaagctgag gtggacga

>0144_LG4

tttttgtaaa taactcccaa tgtttagaat ttatgttagg attgttat-a aaaaatgatc

gttttttaat tctgcatcga atgatataaa aatacaca-g gttttccatt taaataacga

aaaaaaaaaa acgaaaatat aaaaagtttg taaatatgta aacaccctgt agtaattcaa

atcaaacttt cctagtcgaa ccttatttaa cactacgcag taataatgtc gtcaaaaaag

tactgttgct attttttggt ttttaaaact aaagatgtaa aaccgatttt ttaattttat

ttttaacagt aattaaaaaa cggatttatt aaaaaatagg acatgtgaaa agct-aaata

tctaatgagc ataaaaaata atagcatgcc attcaaaaca gaccattagg acgtcttatt

tgagacactt catatcaaat tattttccga agtccttaaa taactctaca atatatcaaa

actctatttt tataacgagc tacagaagac gggagctggg ctggacca

>0184_LG3

---------- ---------- ---------- ---------- ---------- ----------

---------- ---------- ---------- ---------- ------catt taaataagca

aaataaaaac tctaaaatt- ---aagtttg aaaatatcta aacaccctgt agaaattcag

atcaaactta attagttagg tctt------ ---------g taatattgtc atcaagaaag

ca-------- -accgtaacg tcttaaaact aaagatgtct gat---gttt ttgttttttt

ttttaacagt aatccctgaa ccgattt-aa aaaatttagg acatatagga aactaaggca

ttgagtgggt acagaaaata atagtatgcc attaaaaaaa tattattatg acgtcatact

taggac--ac tgtataaaaa tattttttag agtcgttaaa taactctaca aaatatccag

agccca---- -atagcgagc tacagaagac ggaagctgag ctgggcca

>0180_LG5

---------- ---------- ---------- ---------- ---------- ----------

---------- ---------- ---------- ---------g gattgccatt taaataagca

aaataaaagc tctaaaattt aaaaagtttg gaaatatgta aatatcctgt agtaatttaa

atcaaactta ctttgttaaa tcttgtttaa cacttcgtag taaaaatgtc ttcaa-----

---------- ------acat ttcttaaact aatgt-ttaa aaccgattgt ttgttttttt

ttgtaacagt aatccctaaa ccgatttgaa aaaatttaaa acatgtaaga agctaaagta

tctaatgagc acaaaaaata atagcgtgtc attaaaaaaa catgattatg acgtcatact

tgggac--ac tgtataaaaa aattttcctg agcacttttg ata------a aaatatcaaa

actccaactt tataactatc tatagttgac gggagctgag ctggacta

>0223_LG4

---------- ---------- ---------- ---------- ---------- ----------

---------- ---------- ---------- ---------- ---------- ----------

---------- ---------- ---------- ---------- ---------- ----------

---------- ---------- ---tgttcaa ccctacctac taataatctt ataaaaaaag

cagtgttctt attttttaat ttt------- taagatgtaa aaccgatttt gtttttttat

cagtatcagt aatctctgaa ccgatttaaa tatctttagg --acataaga aactaaggta

tccaatgagc acaaaaagta ataacatgtc attaaaaaaa tattattgtg gtatcatact

taggacactc tgtataaaaa tattttccta agtccttgaa taaattgaca aaatgtcaaa

agtccaactt tataaccagc tacagaagat ggcagctgag ctgaacag

>0217_LG7

---------- ---------- ---------- ---------- ---------- ----------

---------- ---------- ---------- ---------- ---------- ----------

---------- ---------- ---------- ---------- ---------- ----------

----aactta attagttaag tcttcttcaa gactacatac taataatgtc gtcaagaatg

caccgtttct attttttgat ttttaaaaaa -----t---- ------tttt ttgttttttt

ttttaactgt agtccctgaa ccgattaaaa aa----tagg acatgtaaga agctaaggca

ct-------- ---aaaaaat atagcatgcc attaaaaaaa ---tattatg acgtcatact

tgggacactt tgtataaata tattttcctg agttctaaaa caactgtata aaatatcaaa

tttccaactt tataacgagt tacagaaaac gggagctgag ctgggcaa

>0155_LG3

---------- ---------- ---------- ---------- ---------- ----------

-ttttctaat tccgcatcga atg--acaaa aataaacagg agttcccatt taaaaaagca

aaataaaagc tctaaagttt aaaaattttg gaagtatgta cacaccctgt agtaacgcag

atcaaactta attagttaga ctttgttcaa cactccctag taataatgat atcaaaggag

tcctgtttgt a-tttttaat ctttaaaact aaaggtgtag aaccgatttt ttgctttttt

ttttaacaat aattcttgaa ccgact-gat gtttttcacg ttatgttagt aggtaaagca

ttaaatgagc acaaaaaatt acgacatgcc attaaaaaa- tattattatg gggtcacact

tgg---tcct attataaaaa tctgttccta agtccttaaa taactctac- aaatatcgaa

-atccaactt tagaatgtgg tagagaatgc ggaagctgtg ctggacca

>0214_LG8

-----ataaa taactccgga tgtttagaat gtgcaatgag gttgtaataa gaaaacgttt

-ctttttaat accgcatcaa atgatataga aataaataga aattcttatt taaataagca

taataaaaga tattaaactt aaaaagttta agaacaggta gtaattc--- --ctattcag

atcaaactta attagttaga tcttgtttaa ctctacttag taataatgtt atcaaaacag

cactatgtgt attttttatt ttttaaaatt aaagttgtaa aacgtgttat ttggttttat

ttctaacagt aatttccaac ccgatttaaa aaaa-gtagg atatgtaaga agctagtgta

tctaat---- ---------- ---------- ---------- ---------- ----------

---------- ---------- ---------- ---------- ---------- ----------

---------- ---------- ---------- ---------- --------

>0170_b_LG7

ttttggcaaa tgactccaaa tgtttaggat ttacatt--- ----aggtaa aaaaatgttc

gttttttaat tctacataga atgatataaa aataagcagg gagtgtcatt taaataagca

aaataaaaac tctaatgttt aaatattttg aaaatattta aacaccctg- ----------

atcaaaatta attagttaat ccttgttcaa cactacgtaa taatagagtc ata--aaaaa

caatgtttca attttttagc ttttaaaact aaaggtgtga accgattttt ttgttttttt

ttttaacatc gatat--gaa tcgatttaaa ataatttaag atttataaga aactaaaaaa

tcgtatgggc acaaaaaata atagcatgcc attaaacaat tattattatg a---------

---------- ---------- ---------- ---------- ---------- ----------

---------- ---------- ---------- ---------- --------

>0243_b_LG2

ttttaacaaa taactcagaa tgtttgagat ttatgttagg gttg--gtaa aaaaatgtt-

---ttctaat tccccatcaa atgggacaaa cataaatagg agttg-tatt taaataacca

aaatataagc tctaaagttt gaaaagtttg aaaa------ ---------- ----------

--------tg ttacattaga ccgtgtccaa cattacttgg taataatgtc attaaaaaaa

cattgcttct attttctaat ttttaaaata aaatatgta- ------gttt tttatttttt

tttt---aat aatccttcaa cagacttgaa aacatttagg acatgtaaga agctaaggta

tctaacgagt acaagaaat- atagcatacc attaaaaaaa tactacgttg acgtcatact

tgagatactc tgtataaaaa tattttct-- ---------- ---------- ----------

---------- ---------- ---------- ---------- --------

>0237_LG3

---------- ---------- ---------- ---------- ---------g aaaaatgttc

gatttttaat tccgcatcga atgctggtat aattaatagg ggttgccatt taaataagaa

aaataa---- ---------t ctaaagtttg acaatatgtg tacaccctgt agtaatttaa

atcgaacttc attagttaga cgttgttcaa tactacgtag ttatagtgtt atcaaagaga

gactaattgt attttttgat ttttaaaacg aaaagagcta atgtttgtat tttttatttt

tttaaccgat ttttttggat tcgattt-aa attttttagg atatgaaaac agcttaagca

tcttataggc ccaaaaaata atatcatgcc acttaaaaaa tatcattacg acgtcacatt

ttgaaagtct agtataaaaa tattttggtg agttcttaaa taactctaca aaatattaaa

agtccaacat t--------- ---------- ---------- --------

>0241_LG9

---------- ---------- ---------- -----ttagg attttagtaa aaaaatgttc

gttttttaat tctgcattga atgat----- ---------- ---------- ----------

-aataaattc tttaatgttt tagaagattg aaaatatgta aacaccctgt agtaagtcag

atcaaattta attacttaaa cctcgttcat tattacctag taataataat ttcatcaaaa

g-cagtttct attttttcaa ttttaaaact aaaggcgtaa aaccgatttt tt------ta

tttttaaagt aatcccaaaa ccgattt--a aaaatttagg acatataaga agctaaggga

caaaa----- --agaagcta ttaattttt- ---------- ttttatcttg acgtcatact

ttggac--ac tgtataaaaa tattttcctg agtccttaaa taacactact aaaaa-----

---------- ---------- ---------- ---------- --------

>0236_LG7

tttttacaaa tcactccaaa tgttgagaat ttacgttagg gttgtaataa aaatatat--

-ttttaaaat tcttcatcaa attatgtaaa attaaacaga agctgccatt taaataatca

aaataaaaac tctaaatttt aaaaagtttg taagta---- -------tgt agtaattcag

atcacact-- --tagttaga tctcgttcat aactaccta- --ttaacgtt atcaagaaag

cactgcttgt attttttaat ttaaaggtgt aaaaa----- -aacgaactt ttgtttttat

ttttaacagt aa-------- ---------- ---------- ---------- ----------

---------- ---------- ---------- ---------- ---------- ----------

---------- ---------- ---------- ---------- ---------- ----------

---------- ---------- ---------- ---------- --------

>0286_LG2

ttttaacaaa taacttggaa tttttagaat ttacgttagg gttgtaataa aaaaatgttt

gttttttaat t--------- ---------a tataaacaga agtttacatt -aaataggca

caataaaggc tctaaaattt aaaaagtttg aaagtatgta aacaccctgt ag--------

---------- ----gttaaa cctaattcaa aactacctag taataaagta tcaaaaaaag

cactgttggt gttttttaat tttaaaaaat taagatgtaa aactaatttt ttgtattttc

ttttaacaat aatctgtaaa ccgtttaaaa atag------ atatatacgt actc------

--taatgaag acattaaatt a-agcatgcc attaaaaaa- ---tattatg tcgtgacact

tggaacactc tgtatttaaa tattc----- ---------- ---------- ----------

---------- ---------- ---------- ---------- --------

>0239_LG3

---------- ---------- ---------- ---------- ---------- ----------

---------- ---------- ---------- ---------- ---------- ---------a

aaa------- ----aattaa attaagtttg gtaatatgta aacaccctga acaaattcag

atcaaactta attagttaat ccttgctcaa cacaacgtag aaataatgtg at-aaaaaag

cattgtttct -ctttttaat tcttaaaact aagtgt---- ------tttt ttatttattt

tttgaacagt aatccttgaa ccaatttaaa aaa---tggg acatgtaa-- --------ta

accattgagc acaaaaaata atcgcatgcc attaaaaa-- tattattatg acgtcgtagt

tggaacaatc tgtataaaaa tattttccta aatcgtcaat gtgttttaaa taatttcaaa

agtccaactc taaaacgagc tacaaaagac gggagctgag ttagacca

**C Alignment of repeats belonging to dispersed TCAST5 elements**

>1LG3

---------- ttctattgtg gcaagaatgg ttttagagca cgacgaagga gtgctataga

atgccacaat taagtcaacg atactaattt ttgttgtttt tagcttatca aagtctgctc

gcctcttaat tttgtcaatt gacgctgttg actcacacag attaccccag ccagcactgc

caacaactcc taaaaattta ctaaaaggag ttgaaaagtt ttgtgagac

>7LG8

---------- ttttattgtg gcacgaatgg ttttagagca cgacgaagga gtgctataga

atgccacaat aaagtcaacg atactaattt ttgttgtttt tagcttatca aaatctgttc

acttcttaat tttgtcaatt ggcgctgttg actca-acac attacaccaa ccagcactgc

caacaactcc taaaaattta ctaaaaggag tcgaaaagtt ttgtgaaac

>223LG3

---------- ---------- ---------- ---------- ---------- ----------

---------- ---------- -----aattt tagttgtttt tagcttatca taatatgttc

acctcttaat tttgtcaatt gacgctgttg actgacacac attaccccag ccagcactgc

caacaactcg taaaaattta ctaaaaggag ttgaacagtt tc-------

>56LG7

---------- ---------- ---------- ---------- ---------- ttgctataga

atgccacaat taagtgaacg atactaattt ttattgtatt tagcttatca aaatctgttc

accttttaat tttgtcaatt gacgctgttg actcacacat attaccctag ccagcactgc

caacaactcc taaaaattta ctaaaaggag ttgaaaagct ttgtaaaac

>4LG9

ttattacacg tcctattgtg gcacgaatgg ttttggagca ccacgaagga gtgctataga

atgccataat aaagtcaacg atactaattt tggttgtttt taacttatca aaatctattc

acctcttact tttgtcaatt gtccctgttg actcacacac attactccag ccagcactgc

caacaactcc taaaaattta ctaaaaggag ttgaaaagct ttgtgaaac

>144LG9

---------- ---------- ---------- ---------- ---------- ----------

------gaat taagtcaacg atacaaattt ttgttgtttt tagcttatta aaatctgttt

atttcttaat tttgtcaatt gacgctgttg acttacacac attacctcag ctagcactgc

caacaacttc taaaaattta ctaaaaagag ttgaaaagct ttgtgaaac

>36LG5

ttataataca ttttattgtg gcacgaatgg ttttggagca caacgaaaga atgac---ga

atgccacaat taagtcaacg atactaattt tggttatttt ttgcttatca aaatctgttt

atctcttaat tttgtcaatt gacgctgttg actcacacac attaccccag ccagcactgc

caagaactct taaaaattta ctaaaaggag ttgaaaagcc tt-taaaac

>5LG10

ttgctatacg ttctattgtg gcacgaatgg ttttagagca cgacgaagga gtgctataga

atggcacaac taagtcaacg atactaattt ttg----ttt ttccttttca aaatctgttc

acctcttaat tttgtcaatt gacgctgttg actcatacat attaccccag ccagcactgc

caacaacttc taaaaattta ctaaaagcag ttgaaaaact ttgtaaaac

>281LG7

---------- ---------- ---------- ---------- ---------- ----------

---------- ---------- ---------- --------tt ttgcttaaca aaatctgttc

acctcttaat tttgtcaatt gacgctgtgg acttacgcac attacctcag ccagcactgc

catcaactcc taaaaattta ctaaaagttg ttgaaaagct ttgtaaaac

>195LG9

---------- ---------- ---------- ---------- ---------- -----ataga

atgccacaat aaagtcaacg atactaattt ttg-----tt tagcttatca aaatctgttt

acctcttaat tttgtcaatt gatgctgttg actcacacat attacctcag ccagcactga

caacaacctc taaaaattta ctaaaaagag ttgaaaagc- ---------

>12LG7

ttattatacg ttttattgtg --tcgattgg ttttagagca cgacgaagga gtgctatagg

atgccaaaat taagtcaaca atactaattt ttgatgtttt tagtttatta aaatctgtac

atctcttaat tttgtcaatt gacgctgtgg actcacacac attacctcag ctagcagtgc

caacaactcc taaaaattta ctgaaaggag ttgaaaagct ttgtgaaac

>32LG3

ttattatacg ttctattgtg gcatgaatgg ttttagagca cgacgaag-- --------ga

atgccataat taagtcaacg atactaattt ttgttgtttt tagcttatta aaatctgttc

acctcttagt tttgtcaatt aacgctgctg actcacacac attaccccaa ccagcactgc

caacaactcc taaaaattta ctaaaaggag ttgaaaaggt ttgtgaaac

>145LG6

ttattattcg ttctattgtg gcacgaatgg ttttggagca cgacgaa--- ----------

---------- ---------g atactaattt ---ttgtttt tagctt---- -taactgttc

acctcttaat tttgtcaatt gacgctgttg actcacatac attaccctac ccagcactgc

caacaactcc taaaaattta ctaaaaggag ttcaaaagct ttgtgaaac

>283LG6

---------- ---------- ---------- ---------- ---------- ----------

---------- ---------- ---------- ---------- ---------- ----------

-cctctgaat tttgtcagtt gacgctgttg actcacacac attacctcag ccagcactgc

caaaaactcc taaaaattta ctaaaagtag ttcaaaagct ttataaaac

>149LG2

ttattatacg ttctattatg gcacgaatgg ttttggagca cgacgaag-- ----------

--gccacaaa caagttaac- ------attt ttgttgtttt aaacttatca aaatctgttc

acctcttaat tttgtcaatt gacgctgttg actcaaacac attaccctag ccagcactgc

caacaactcc taaaaatgta ctaaaaggag ttgaaaagct ttgtgaaac

>76LG3

ttattatacg ttctattgtg gcacgaaggg ttttggagca cgacgaagga gt--------

---------- ---gccaacg atacaaattt atgttgtttt tagcttatca aaatctgttt

acctcttatt tttgtcagtt gacgctattg acccacacac atgaccacag ccagcactgc

caacaacttc taaaaattta ctaaaaggag ttgaaaagtt ttgtgaaac

>190LG8

---------- ---------- ---------- ---------- ---------- ----------

---------- ------aaag atactaattt ttgttgtttt tagcttatca aaatctgttc

acttcctaat tttgt-aatt gacgctgttg acttacacac atgaccgcag ccagcacttc

caacatctct taaaaattta ctaaaaggag ttgaaaagct ttgtg----

>69LG7

---------- ---------- ---------- ---------- ---------- ----------

----aacaat taagtcaact atactaactt tggttgtttt tagcttagca aaatctattc

acctcttaat tttgtcaatt gaccccgttg actcacacac attacccaag ccagcactgc

caacaactcc taaaaatgta ctaaaaggag ttgaaaagct ttgtgaaac

>10LG9

ttattatacg ttctattgta gcacgaatgg ttttggagca caacgaagaa gtactataga

atgccacaag taagtcaatg atagtaattt ttgttgtttt tagcttatga aaatctgttc

acctcttaat tttgtcaatt gacgctcttg actcacacac atcaccccag ccagcactgc

caacaactcc taaaagttta ctgaaaagag ttgaaaagct tcgttaaac

>21LG10

ttattatacg ttcta-tgta gcatgaatgg ttttggagca cgacgaaaga gtgctataga

atgccacaat taagtcaacg atactaattt ttgatgtttt tagcttatta aaatctgttc

acctcttaat tttgtcaatc gacgctgttg gctctcacac attaccatag ccagcactgc

caacaactcc ttaaaattta ttaaaaagag ttgaaaagct ctgcgaaac

>182LG8

---------- ---------- ---------- ---------- ---------g gtgctataga

ataccacact taagtcaacg atactaattt ttgttgtttt tagcttatca aaatctgttc

acctcttaat tttgtcaatt gacgctgttg actcacacac tttacccttg ccagcactgc

caacaacccc taaaa----- ---------- ---------- ---------

>62LG3

ttattaaacg ttttattgtg atatgagtgg ttttggagca cgacgatgga gtgctataga

atgccacaat taagtcaacg atacaaattt ttgttgtttt tagctgatca aaaactgttc

atttcttaat tttgtcaatt gacgctgttg acgcacaaac attaccccag ccagaactgc

caacagctct taaaaattta ctaaaagaag ttaacaag-- ---------

>220LG3

---------- ---------- ---------- ---------- ---------- -----atgga

atgccacaat taagtcaacg atagtaattt ttgttgtttt tagtttatga aaatctgttc

atctcttaat tttgtcaatt gacgctgttg actcacacac attaccctag ccagcactgc

caacaactcc taaa------ ---------- ---------- ---------

>6LG9

ttattatacc tcctattgtg gcacgaatga ttttggagca cgacgaacga gtgctataga

atgccacaat taagtcaacg atactaattt ttgttgtttt tagtttatca aaatctgttc

acctcttaat tttgtcaatt gacgctgtag acttacacac attaccccaa ccagcactgc

caagaactcc taaaaatttg ctaaaagtag tagaaaagct ttgtcaaac

>230LG8

ttattatacc ttctattgtg gcacgaatgg ttttagagca cgacgaagta gtgctataga

atgccacaat taaatcaacg atactcattt ttattgtttt tagcttatca aaatctgttc

acctcttaat tttg------ ---------- ---------- ---------- ----------

---------- ---------- ---------- ---------- ---------

>40LG7

ttattatacg ttctattgtg gcacgagtgg ttttggagca cgacgaagga gtgccacaga

atcccacaat taagttaacg atactaattt ttgttgtttt tagcttatca aaatctgttc

acctcttaat ttggacaagt aactctattg attcacacac attaccccag ccagcactac

caacaactcc taaaaattta ctgaaagga- ---------- ---------

>257LG8

ttattatacg ttctattgtg gcacgaatgg ttttggagca cgacgaagga gttctataga

atgccataac taagtcaaca atactaactt ttgttgtttt tagcttatca aaatctgttc

accatttaat tttg------ ---------- ---------- ---------- ----------

---------- ---------- ---------- ---------- ---------

>232LG9

ttattatatg tcctattgta gcacgaatgg ttttggagca cgacgaaagg gtgatataga

ttgccacaac taagtcaacg ttactgatct ttgttgtttt tagcttatca aaatctgttc

acctcttaat tttg-----t gacgctgttg actcacacac attac----- ----------

---------- ---------- ---------- ---------- ---------

>19LG3

taataataca ttcttttgtg gcacgaatag ttttggagca cgacgacgga gtgctataga

atgccataat taagtcaacg atactaattt ttgttgttct tagcttatca aaatatgttt

acctcttaat tttgtcaatt gaccctgttg actcacacac attaccccag ccagcactgc

caacaactcc taaaaattta ctaaaaggag ttgaaaag-- ---------

>266LG2

---------- tcctattgtg gcacgaatgg ttctggagca cgtcgaagga gtgctgtaaa

atgccacaat taagtcaacg atactaattt ttgttgtttt tagtttatcg aaatatgttc

acctcttaat tttgtcaat- ---------- ---------- ---------- ----------

---------- ---------- ---------- ---------- ---------

>20LG2

tatatatacg ttttattgtg gcacgaatgg ttttggaaca cgacgaagga gtgctaaaga

atgccacaat taagtcaacg atactagttt ttgttgtttt tagcttatca aaatctgttc

acctcttaat tttgtcaat- -------ttg actcacacat agtagcccag ccagcactgc

ctacaacttc taaaaattta ctaaaactag ttgaaaacct ttgtgaaac

>25LG2

ttattatacg tactactgta acacgaatgg ttttggaaca cgacgaagga gtgctataaa

atgccacaac taagtcaaca atactaattt ttgttgattt tagcttatta aaatctgttt

atctctaaat tttgtcattt gacgtctttg attcacacac gttaccctag ccagcactgc

caacaactcc caaaaattta ctaaaaggag ttgaaaagtt ttgtgaaac

>13LG2

-tattattcg ttctattgtg ccacgaatgg ttttggagca cgacgaagga gtgctataga

atgccacaat taagttaacg ttactaattt ttgttgtttt tagcttatca aaatctgttc

acctcttaat tttgtcaat- -------ttg acttaaatac attgccccag ccagcactgc

caacaactcc taaaaattta caaaaaagag ttgaaaagct ttgtgaaac

>200LG6

ttattatacg ttcagttgtg gcaccaatgg ttttggagca cgacgaagga gtgctataaa

atgccacaat taagtcaacg atactaattt ttgttgtttt tagcatataa aaatctattc

gtctcttaat tttgtcaatt gacgctgta- ---------- ---------- ----------

---------- ---------- ---------- ---------- ---------

>9LG3

ttattatacg tcctattgtg tcacgaatgg ttttagagca cgacgaagga gtgctataaa

atgccacaat taagtcaacg atactaattt ttgttgtttt tagcttatca aaatctgttc

atgtcttaat tttgtcaatt gacgctgttg actcacacac attaccccag tcagcactgc

caacaacttc taaaaattta ctaaaagcag ttgaaaagct ttgtgaaac

>75LG2

ttattacacg ttctattgtg gcacgaatgg ttttggagca agacgaaaaa gtgctataga

atgccacaat taagttaacg atactatttt ttgttgtttt tagcctattg aaatctgttc

accttttaat tttgtcaatt gacgctgttg acttacacac attaccctag ctagcacttc

caacaactcc taaaaattta cta------- ---------- ---------

>102LG3

ttattatacg ttctattgtg gcacgaatgg ttttggagca cgacgaagga gtgctataga

atgccacaat taagtcaacg atactaattt tttttgtttt tagctcatca aaatctgttc

atcgcttaat tttgtcaatt gtcgctgttg acttacacac attac----- ----------

---------- ---------- ---------- ---------- ---------

>136LG5

ttattaaacg ttctattgtg gcacgaatgg ttttggagca cgacgaaaga gtgctataga

atgccacagt taagtcatcg atactaattt ttgttgattt tagcttatca aaatctgttc

acctcttaat tttgtcaatt gatgctgttg actcacacac att------- ----------

---------- ---------- ---------- ---------- ---------

>197LG3

ttattatacg ttctacagca gcacgaatgg ttttggagca cgacgaagga gtgctataaa

atgccacaat taagtcaacg atactaattt ttgttatttt ttgcttatca aaatctgttt

accccttaat tttgtccatt gacgctgttg actcacac-- ---------- ----------

---------- ---------- ---------- ---------- ---------

>14LG2

ttattatacg ttctattgta gcacgaatgg ttttggagca caacaaagga gtgctataga

atgacacaat taagtcaacg atactaattt ttgttgtttt tagcttatca aaatctgttc

acctcctaaa tttttcaatt gacgctgttg actcacactc attac--tga gacaccaagc

caacaactcc taaaaatttg ctaaaaggag ttgaaaagca ttgtgaaag

>274LG8

---------- --atattgtg gcataaatgg ttttagagca cgacgaaaga gtgctaaaaa

atgccacaat taaatcaatg atactaattt ttgttgtttt taacttttga aaatctcttc

acgtcttaat tttgtcaatt gacgctgtag actcaca--- ---------- ----------

---------- ---------- ---------- ---------- ---------

>31LG9

tattattacg ttctattgtg gcatgaatgg ttttggagca cgacgaagga ctgctataaa

ttaccacaat taagtcaacg atacaaattt ttgttgtttt tagcttatca aaatctgttc

atctgttaat tttgttaatt gacgctgttg attcacatac aataccccag acagcactgc

caacaactcc taaaaattta ctaaaaggag ttgaaaaact ttggga---

>158LG3

------tacg ttctattgtg gcacgaatga ttttggagca cgacgaagga gtgctataga

ttaccacaat taattcaacg atactaattt ttgttgtttt aagctaatta aaatctgttt

acatcataat tttctcaatt gacgctgttg attcacacac attacct--- ----------

---------- ---------- ---------- ---------- ---------

>97LG6

ttat--tacg ttctattgtg gcacgaatga ttttggagca cgacgaagga gtgctataga

atgccacaat taagttaacg acattaattt ttgttgtttt taccttatca aaatctgttc

acctgttaat tttgttaatt gacgctgttg ----acacac attaccccag ccagcactgc

caacaactct taaaaattta ctaaaaagag t--------- ---------

>3LG10

ttattatacg ttctattaag gcacgcatgg ttttggagca cgacgaagga gtgctatag-

atgcaacaat taagtcaacg ataataactt ttgttgtttt tagcttatca aaatctgttc

accgctaaat tttgtcaatt gattctgttg actcaaacac attaccccca ccagcactgc

caacaactcc taaaaattta ctaaaagtag ttgaaaagct ttgtgaaac

>130bLG8

---------- ---------- ---------- ---------- ---------- ----------

---------- ------aacg atactacttt ttgttgtttt taggttatca aaacctgttc

acctcttaat tttgtcaatt gacgctgttg gctcacacac attacctctg ccagcactgc

caacaactcc taaaaattta ctaaaagtag ttcaaacgtt ttgtgaaac

>27LG10

---------- ---------- ------atgg ttttggagca cgacgaaaga gtgctataaa

ataccataat taagtcaacg atacaaattt ttgtcgattt tagtttatca aaatctgttc

acctcttaat tttgtctatt gacgttgatg actcacacac attaccccag ccagtactgc

caacaactcc taaaaattta ctaaaaggag ttgaaa--ct ttgtgaaac

>2LG2

ttattattcg ttctattgtg gcgcaaatag ttttggagca cgacgaagga gtgctataga

atgccacaat taagtcaacg atactaattt gtgttgcttt tagtttatca aaatttttta

atctcttaat tttgtcaatt gacgctgttg actcacacac attaccctag ccagcactac

caagagctcc taaaaattta ctaaaaagag ttgaaat-ct ttgttcaac

>35LG3

ctattatacg ttctattgtg gcacgaatgg ttttggagca cgacaaagga gtgctataga

aggcaacaaa taagtcaacg ctactaattt ttgttgtttt tagcttatca aaatctatta

tccttttaat tttgtcaatt gacgctgttg acacacacac attaccccag ccagcactgc

caacaactcc taaaaattta ctaaaagaag ttgaa----- ---------

>30LG4

ttattatacg ttctattgtg acacgaatgg ttttggagca cgacgaagga gtgctataga

at-------- taagtcagcg atactaagtt ttgttgtttt tagtttatca atacctgttt

acctcttaat tttgtcaatt gatcctgttg actcacacac attaccccag ccagcactac

caacaactcc taaatattta ttaaaaggag ttgaaaagct ttgtaaaac

>181LG8

---------- ---------- ---------- ---------- ---------- -------cga

aggccacaat gaagtcaaca atagtaattt ttgttgtttt tagcttatca aaatctgttc

tcctcttaat tttatcagtt gacgctgttg actcagacac attaccccag caagcactgc

caacaactct taaaaattta ctaaa----- ---------- ---------

>11LG3

ttattatacg ttctattgtg gcaggaatgg ttttggagca cgacgaagga gtgccataga

aagccacaat aaagtcaacg atactaattt ttgttgtttt aagcttatca aaatctgttc

-cctcttaat tttgtcagtt gacgctgttg actcacacac attagcccag ccggcactgc

caacaactcc taaaaattta ctaaaagtag tggaaaaact ttatgaaac

>258LG7

---------- ---------- ---------- ---------- ---------- ----------

---------- --agtcaacg atactaattt ttgttatttt tagcttatca aaatctg---

-cctcttcct tttgtcaatt gacgctgttg acccacacac attaccccag ccagtactgc

caacaactcc taaaaattta ctgaaaggag atgaaaaa-- ---------

>26LG4

ttattatacg ttctattgag gcataaatgg ttttggagca cgacgaaaga gtgctataga

atgctacact taagttaacg atactaattt ttgatgtttg tagcttatca aaatctgttt

acctctttat tttatcaatt gacgctgttg actcacaccc attaccccag ctagcactgc

caacaacttc taaaaattta ctaaaaggag tagaaaaatt ttgtgaaac

>79 corrLG8

ttattata-g ttcta--gtg gcacgaatgg ttttagagca caac------ --------ga

atgtcacaat aaagtcaacg atactaattt ttattgtttt tagcttttca aaatctgttc

atctcttgat tttgtcaatt gacgctgttg acttacacac attaccccag ccaacactgc

caacaactcc taaaaattta ctaaaaggag ttgaaaagct ttgtaaaac

>364LG9

---------- ---------- ---------- ---------- ---------- ----------

---------- ---------- ---------- -------ttt taatttctca aaatctgttt

atcttttaat tttgtcaatt gacgctgtag acttacacac attaccccag ccaggactgc

caacaactcc taaaaattta ctag------ ---------- ---------

>163aLGX

---------- ttctgttgta acacgaatga ttttggagca cgacga-gga gtgctataga

atgc------ ------aacg atactaattt ttgttgtttt tagtttatca aaatctgttc

atct-ataat tttgtcaatt gacgctgttg actcacacac attaccccag ctagcactgc

caa------- tagaaattta ctaaaaggag ttgaaaagct ttgtgaaac

>163bLGX

---------- ---------- ---------- ---------- ---------- ----------

---------- ---------- ----ttattt ttgttgtttt taatttatca aaatctgttc

atctattaat tttgtcaatt gacgctgttg acccacacac attaccccag ctagcactgc

caa------- tagaaattta ctaaaaggag ttgaaaagct ttgtgaaac

>163eLGX

---------- ---------- ---------- ---------- ---------- ----------

---------- ---------- ------attt ttgttgtttt taatttatca aaatctgttc

atctattaat tttgtcaatt gacgctgttg acccacacac attaccccag ctagcactgc

caa------- tagaaattta ctaaaaggag ttgaaaagct ttgtgaaac

>163jLGX

---------- ---------- ---------- ---------- ---------- ----------

---------- ---------- ------attt ttgttgtttt tagtttatca aaatctgttc

atctattaat tttgtcaatt gacgctgttg actcacacac attaccccag ctagcactgc

caa------- tagaaattta ctaaaaggag ttgaaaagct ttgtgaaac

>163dLGX

---------- ---------- ---------- ---------- ---------- ----------

---------- ---------- ------attt ttgttgtttt tagtttatca aaatctgttc

atctattaat tttgtcaatt gacgctgttg actcacacac attaccccag ctagcaccgc

caa------- tagaaattta ctaaaaggag ttgaaaagct ttgtgaaac

>163fLGX

---------- ---------- ---------- ---------- ---------- ----------

---------- ---------- ------attt ttgttgtttt tagtttatca aaatctgttc

atctattaat tttgtcaatt gacgctgttg actcacacac attaccccag ctagcaccgc

caa------- tagaaattta ctaaaaggag ttgaaaagct ttgtgaaac

>163gLGX

---------- ---------- ---------- ---------- ---------- ----------

---------- ---------- ------attt ttgttgtttt tagtttatca aaatctgttc

atctattaat tttgtcaatt gacgctgttg actcacacac attaccccag ctagcaccgc

caa------- tagaaattta ctaaaaggag ttgaaaagct ttgtgaaac

>163hLGX

---------- ---------- ---------- ---------- ---------- ----------

---------- ---------- ------attt ttgttgtttt tagtttatca aaatctgttc

atctattaat tttgtcaatt gtcgctgttg actcacacac attaccccag ctagcactgc

caa------- tagaaattta ctaaaaggag ttgaaaagct ttgtgaa--

>163cLGX

---------- ---------- ---------- ---------- ---------- ----------

---------- ---------- ------attt ttgttgtttt tagtttatca aaatctgttc

atctattaat tttgtcaatt gacgctgttg actcacacac attaccccag ctagcaccgc

caa------- tagaaattta ctaaaaggag ttgaaaagct ttgtgaaac

>163iLGX

---------- ---------- ---------- ---------- ---------- ----------

---------- ---------- --acttattt ---ttgtttt tagtttatca aaatctgttc

atctattaat tttgtcaatt gacgctgttg actcacacac attaccccgg ctagcaccgc

caa------- tagaaattta ctaaaaggag ttgaaaagct ttgtgaaac

>37LG4

---------- tcctattgtg gctcgaatgg ttttggagca cgacgcagaa gtgctatagg

atgccacaat taagtcaacc atattaattt ttgttgtttt tagct--tca aaatatgttc

acttcttaat tttgtcaatt gacgctgttg actcacacac gttacgctat ccagcactgc

taacaactcc taaaaattta ctaaaaggag ttgaaaagct ttgtgaaac

>125LG10

ttgttatacg ttctattgtg gcacgaatgg ttttggagca cgacgcagta gtgctttaga

atgtcacaat taaatt---- -tactaattt ttgttgtttt tagcttataa aaatatattc

accacataat tttgtcaatt gacgctgttg actcacacac attaccccaa ccagcactgc

aaacaactcc taaaaagtta ctaaaaggag ttgaaaagct ttgtt----

>356LG3

---------- ---------- ---------- ---------- ---------- ----------

---------- ---------- ---------- ---------- ---------- ------attc

acctcttaat tttgtcaatg ttgacagatt tgtcacacac attactccag ccagcactgc

caacaactcc taaaaattta ctaaaagaag ttgtaaagc- ---------

>104LG9

ttattatacg ttttattgtg gcacgaattg tt-------- ---------- -----gccat

atgccacaat taaatcaacg atactagttt ttgttgttct tagcttatca agatctgttc

acctcttaat tttgttaatt gacgctgttg actgacacac attactccag ccagcactgc

caacaactcc taaaaattta ctaaaaggag ttgaaaagct ttatgaaac

>67LG10

---ttatacg ttctattgtg acacgaataa ttttggagca cgacaaagga gtgctatcga

atgccacaaa taagtcaaca atactaattt ttg-----tt tagcttatca aaatctgttc

acttcctaat tttgtcaatt gacgctgttg actcacacac attaccctag ccaatactgc

caacaactcc taaaaattta ctaaaaggag ttgcaaag-t ttgtgaaac

>70LG8

-taatatacg ttttattgtg gcatgaaagt ttttggagca cgtcgaagga gtgctataga

atgccccaac taagttaaca atactaattt ttgttgtttt tagcttatta aaatttgttc

atttcttaat tttgtcaatt gacactgtgg actcacgcac attaccctag ccagcactgc

caacaactct taaaaagtta ctaataggag ttgaaaagct ttgtgaaaa

>46LG8

ttattatacg ttctattgtg gctcgaa--- ---tgtggca ctccttcgtt gtgctataga

atgctacaat ttagtcaacg atacta-cta tttttgtttt tagcttatca aaatctgttt

acatcttagt tttgtcaatt gacgctgttg actcacacac attactccag tcagcactgc

caacaactcc taaaacttca ctaaaaggag ttgaaaagct ttgtgagtt

>212LG9

---------- ---------- ---------- ---------- ---------- gtgctataga

atgccacaat taagtcaaca atactaattt tt-ttgtttt tagcttatca aaatctgttt

atctcttaat tgtgtcaatt gacgctgttg actcacacat ataaccctag ccagcactgc

caacaa---- ---------- ---------- ---------- ---------

>108LG10

---------- ---------- ---------- -----aagca cgacgaagca gtgctataga

ataccacaat taaatcaaca atacta-ttt ttgttgtttt tagcttatca aaatgtgttc

atctattaat tttgtcaatt gacgctgttg actaacacac attaccccag ccagaactgc

caacaactcc taaaaatgtt ctaaaaggag ttg-aaacct ttgtgaatc

>114LG3

ttattgtacg -----ttgta ccatgaatgg ttttggagca cgacgaagga gtgctataga

atgccacaat taagtcaacg atactaattt ttgttgtttt taccttatca aaatctgttc

acctcttaat tttgtcaatt aatgctgttg actcacacac attaccccag ccagcactgc

caa---ctcc taaaaattt- ---------- ---------- ---------

>15LG2

ttattatacc ttctattgtg gtactaatgg ttttggatca ca-----aaa ttattataga

atgccgcaat taagtcaacg atagtaattt ttgttgtttt aagcttatca aaatctgttc

acctcttaat tttgtcagtt gacgctgttg actcacacac atttc----- ----------

---------- ---------- ---------- ---------- ---------

>127LG8

---------- ttctattgtg gcacgaatgg ttttggagca cgacg----- -tgctataga

atactacaat taagtcaacg acacaaattt ttgttgtttt tagcttatca aaatctgttc

acctcttaat tttgtcaatt aacgctgttg actcacacac attaccccag ctcgcactgc

aaacaactcc taaaaatttt agaaaaggag ttgaaaagct tc-------

>289LG6

---------- ---------- ---------- ---------- ---------- -----ataga

atgccacaat taagttaccg atactaattt ttgttgtttt tagcttatca aaatctgttc

acctcttaat tttgtcaatt gacgac-ttg actcacacac actac----- ----------

---------- ---------- ---------- ---------- ---------

>157LG3

---------- ---------- -------ggc ttttggagca ctacgaagga atgctataga

atgctacaat taagtcaacg atactaattt ttgttgtttt tagctaatca aaatctggta

accttttaat tttgtcagtt gacgctgtta actcacacac attaccccag ccaacactgc

caacaactcc taaaaattta ctaaaagaag ttg------- ---------

>130aLG8

ttattatacg tcttattgtg acacgaatat tttt------ ---------- -----gaaga

atgccacaat taagtcaacg atactagttt ttgttgtttt tagtttatta aaatctgttc

acctcttaat tttgtcaatt gacgctgttg gctcacacac attaccaaaa ccagcactgc

---cgactcc aaaaaaatta cttaaagggg ttgataagct ttgtgaaac

>189LG10

---------- ---------- ---------- ---------- ---------- gtgctataga

atgccacaat taagttaaca atactaattt ttgttgtttt tggcttttcg aaatctgttc

acttcttaac tttgtcaatt gacgctgttg actcacacac attac----- ccagcactgt

caacaaattt taaaagttta ctaaaagaag ttgaaaagtt ttgtgaaac

>162LG10

---------- ---------- ---------- ---------- ---------- ---ttataga

atgccatagt caagtcgaca gtattaattt ttgttgtttt tagcttatca aaatctgtta

atttcttaat tttgtcagtt gacgctgttg acccacacac attaccccag tcagcactgc

caacaactcc taaaaattta ctaaaaggag ttgaaaggcg ttgtgaaac

>8LG2

ttattatacg ttctattgtg gcacgagtgg ttttggagca cgacgaaaga gtgctataga

atgccacaat taagtcaacg atacta-att ttgttgtatt tagcttatca aaatctgttc

cccttttaat tttgtcaatt gacgctattg actcacacac attactccaa ccagcactg-

--acaactcc taaaaattta ctaaatggag ttgaaaagct ttgtgaaac

>65LG4

ttattatacg ttcttttgtg acacgagtag ttttggagca cgacgcagaa gtgctataga

atgccacaat taagtcaacg ataata--tt ttgttgtttt cagcttatca gaatctgttc

acctcttgat tttgtcaatt gacgctgttg actcacacac attacctcag acagcactgc

caacaactcc taaaaattta ctaaaaggag ttgaaaagct ttgtgaaac

>155LG8

---------- ---------- ---------- ---------- ---------- ----------

---------- ---------- -tactaattt ttgttgtttt tagcttatca aaatctgttc

acctcttaat ttagtcaatt gacactgttg acttacttgt attaccccag ccagcactgc

caacaactcc taaaaattta ctaaaaggag atgaaaaact ttgtgaaac

>350LG3

---------- ---------- ---------- ---------- ---------- ----------

---------- ---------- ---------- ---------- ---gttatca acatctgttt

atctcttaat tttgtcattt gacgctagtg acccatacac attacctcag ccagcactgc

caacaactcc caaaaattta ctacaaggag ttgaaaagct ttgtgaaa-

>105LG4

----tatacg ttttattgtg gcacgaatgg ttttggagca cgtcgaaaga gtgctatcaa

atgctacaat taagtcaacg atactaattt ttgttgtt-- ---ccaatca aaatctgttc

atttcttaat tttgtcaatt gacgctgttg actcacacac attaccccag ccagcactgc

caacaactcg taaaaattga ctaaaaggag ttgaaaagct ttgtgaaac

>17LG8

ttattatacg ttctattgtg gcacgaatgg ttttggagct cgacgaagga gtgctataga

atgccacaat taagtcaacg atactagttt ttgttttttt tagcttatca aaatctgttg

atcttttaat tttgtcaatt gacgatgttg actcacacac attacccctg ccagcactgc

caacaactcc aaaaaaatta ctaaaagaag ttgaaaacct ttgtgtaac

>68LG10

ttattatacg ttctattgtg gcacaaatgg ttttcgagca cgacgaagga gtgcaataga

atgccacaat taagttaacg atactaattt ---ttgtttt tagcttatca aaatctgttc

acctcttaat tttgtcaatt gacgctgttg actcacacac attaccccag tcagaactgc

caacaactcc tacaaattta ctaaaaggag ttgaaaagtt ttgtgaaac

>243LG2

ttattatacg ttctattgtg gcacgaatgg ttttcgagca cgacgaagga gtgctataga

atgccacaat taagtcaacg atactaattt tt------tt ttacttatca aaatttgttc

atctcttaat tttgtcag-- ---------- ---------- ---------- ----------

---------- ---------- ---------- ---------- ---------

>121LG5

ttatcatacg atctattgtg gcgcgaatgg ttttggaaca caacgaagga gtgctataga

atgccacaat taagtcaacg atactaattt ttgttgtttt ttacttatca aaatctcttc

atctcttaat tttgtcaatt gacgctgttg actcacacac attacccttg acagcact--

---------- ---------- ---------- ---------- ---------

>47LG3

ttattatacg atctattgtg gcacgaatgg ttttggagca cgacgaagga gtgcgataga

atgccacaat taagttaacg atactaattt ttgtaatttt ttgcttttga aattctgttc

acctcttaat tttgtcaatt gacgctgtta actcacacat attatcccag ccagcactgc

caacaactgt taaaaattca ctaaaagtag ttgaaaagct ttgtgaaag

>117LG5

ttattatacg ttcccttgtg gcacgaatgg ttttggagca cgtggaagga gtgctataga

atgccacaat taaatcaacg atactacttt ttgttttttt tagcttatca aaatatgttc

accttttaat tttgtcagtt tacgctgttg gctcatacac cttac----a ccagcactgc

caacaacttc taaaaatcta ctaaaaggag ttgaaat--- ---------

>63LG2

ttattatacg ttctattgtg gcgcgaatgg ttttagagca cgacgaaaga gtgcgataga

atgtcacagt taagtcaaca atactggttt ttgttgtttt tagcttactg aaatttgttc

acttcttaag tttgtcaatt gacgctgttg actcacacac attgc----- ----cactgc

caacaactcc taaaaattca ctaaaaggag ttgaaaagct ttgtgaaac

>216LG9

---------- ---------- --------ag ttttagagca cgtcaaagaa gtgctataga

atgccacaat taagtcagcg atactaattt ttgttgattt tagcttatca aaatctgccc

acctcttaat tttgtca--t gatgctgttg actcacacac attac----- ----cactgc

taacaactcc taaaaattta ctaaaaggag ttgaaaaact tt-------

>245LG9

---------- ---------- ---------c ttttagagca cgtcaaagaa gtgctataga

acgccataat taagtcagcg atactaattt ttgttgattt tagcttatca aaatctgccc

acctcttaat tttgtca--t gatgctgttg actcacacac attac----- ----cactgc

taacaactcc taaaaattta ctaaaaggag ttaaaaaact tt-------

>33bLG2

ataataatac ttctatcgta gcacgaatgg ttttagagca cgacgaagga gtgctataga

atgccacaat taagttaacg atactacttt ttgttgtttt -agcttctca aaatctgttc

acctcttcat tttgtcaatt gacgctgttg aca------- ---------- ----------

---------- ---------- ---------- ---------- ---------

>244LG4

---------- ----attgtg gcacgaatga ttttctagca cgaagaagga gtgctataga

atgccacaat taagtcaacg atactaattt ttattgtttt tagtttatca aaatcttttc

acctcttaat tttgtcaatt gacgctgttg ac-------- ---------- ----------

---------- ---------- ---------- ---------- ---------

>141LG3

ttattatacg ttctgttgtg gaacgaatgg atttggagca cgacgaagga gtgctataga

atgccacaat taagtcaacg atactaattt ttattg--tt tagcttatca aaatcagttc

acctcttaat tttgtcaatt ggcccttttg actcacaaat attac---ga ccagcactgc

caacaactcc taaaaattta ctaaagag-t ttgaat---- ---------

>98LG9

tcattatacg ttctattgtg gcccaattgg ttttggagca cgacgaagga gtgctataga

atgccacaat taagtcaacg aaactaattt ttgttgtttt tagcttatta aaatctattc

acttcttaat tttgtcaatt gacgctgtta actcacacac actaccccat taagcactgc

caataaatac taaaaattta ctaaaaggag ttgaaaagct ttgtgtaac

>319LG9

----tataca ttttattgag gcaggaatgg ttttgaagca ccacaaagaa gtattataga

atgccacaat taagtcaacg atactaaact ttgttgtttt taggttttca aaatctgttc

acctcttcat tttg------ ---------- ---------- ---------- ----------

---------- ---------- ---------- ---------- ---------

>191LG4

ttattatacg ttctattgtg gcacgaaggg ttttggagca cgacgaagga gtgctataga

atgccacaat taagccaacg atactaattt ttgttgtttt tagcttttaa aaatctgttc

atctcttaat t--------t gacgctgttg acttacacat attaccccag ccagcactgc

caacatttcc tagaaattta ttaaaaggag ttgaaaagct ttgtgaaaa

>318LG5

ttattatacg ttctggtgtg ttacgaatag ttttggagca cgacgaagga gtg--gagaa

ttgccacagt taagtcaacg atactaattt ttgttgtttt tagcttatca aaatctgttc

atctcttaat t--------- ---------- ---------- ---------- ----------

---------- ---------- ---------- ---------- ---------

>262LG6

ttattatacg ttctactgtg ccacgaataa ttttggagca cgacaaagga gtgctataga

atgccacatt taaatcaacg atattagttt ttgttgtatt tagcttatca aaatctgttt

atctcttaat ttggttaatt a--------- ---------- ---------- ----------

---------- ---------- ---------- ---------- ---------

>16LG3

ttattatacg ttctattgtg gcacgaatgg ttttggagca cgacgaagga gtgctgtaca

atgccacaat taagttaacg atacaaattt ttgttgtttt tagcttttca aaatctgttc

atctttttat tttgtcaatt gacgctcttg actcacacac attacccgaa ccagcactgc

caacgattcc taaaaattta ctaaaagaag ttgagaagct tagtgaatc

>227LG7

ttattatatt tactattgtg gcacgaatgg ttttggagca cgacaaagga gtgctataga

atgccacaat taagtcaacg atact-attt ttcttatttg tagcttttca aaatctgttc

atctcttaat tttgtcaatt g--------- ---------- ---------- ----------

---------- ---------- ---------- ---------- ---------

>229LG10

ttattatacg ttctattgtg gcatgaatgg ttttgtagca cgacgaagga gtgctataga

atgccacaat taagtcaacg atgtttgttt ttgttgtttt tagcttatca aaatctattt

acctcttaat tttgtaaatt gacgctgttg ac-------- ---------- ----------

---------- ---------- ---------- ---------- ---------

>122LG3

ttattatccg ttctattgtg gtacgaatgg ttttggagca cgacgaaggg gtgctacaga

atgccacaat taagtaaacg atactagttt tggttgtttt tagcttatca aaatctgttc

atctcttaat tttgtgaatt gaccctgttg actcacacac attacctcag cca-------

---------- ---------- ---------- ---------- ---------

>184LG2

ttattatagc ttctattgcg gcacgaatag ttttggagca cgacgaaaga gtgctataga

ataccacaat taagtcaacg atactagttt ttgttgtttt taacttatca aaatctgttg

acttcccaat tttgtcaatt gatgctgttg actcacacac attac----- ----------

---------- ---------- ---------- ---------- ---------

>152LG8

ttattatacg ttctattgta acacgaatag ttttggagca cgaagaagga gtgctgtgga

atttcacaat taaatcaata atacttattt ttgttgtttt taatttatca aaatctgttc

acctcttaat tttgtcaatt gacgctgttg agttacacac attac----g ccaacactgc

caacaacttc taaaaatttt ctaaaaagaa ttgaaaaact ttgtgaaac

>54bLG2

ttattatacg ttctattgtg acacgaacgg ttttagagca cgacgaagga gtgctgtaga

atgccacaat taagtcaacg atactaattt ttgtt-tttt tagcttgtca aaatctgttc

acttcttaat tttatcaatt gactctgttg actcacacac attacctcag tcagcactcc

caacaacttc taaaaattta ctaaaaggag gtgaaacgct ttgtgaa--

>18LG8

ttattgtacg ttttattgtg gcacgaatgg ttttggagca cgacgaagaa gtgctataga

ataccccaat aaagtcaacg atactaattt ttgttgtttt tagcatatta aaatctgttc

atctcttaat tttgtcagtt aactctgttc attcacacac attactccag ccagcactgt

taacaactac taaaaattta ctaaaagcag ttgaaaagct ttgtgaaac

>39LG3

--attatacg ttctattaaa gctcgaatgg ttttggagca cgacgaagga gtgctttaga

atgccacaat taagtcaacg atacta---- ------tttt tagcttatca aaatctgttc

accccttaat tttgtcaatt gtcgctgttg acttacacac atta-cccag ccagcactgc

caacaactcc tacaaagtta ctaaaaggag ttgaaaagtt ttgtgaaac

>126LG3

---------- ttctatcgtg gcacgaatgg ttttggagca cgacgaagga gtgatataga

atgccacaat taagtcaacg ataataa--- --------tt gagcttatta aagtctgttg

acctcttaat tttgtcaatt ggcgctgttg actcacagat attacgctag ccagcactgc

caacaactcc taaaaattta ctaaaaggag ttgaaaagct ttgtgaaat

>38LG5

ttattatacg ttctcttgtg gcataaatag ttttggagca cgacgaagga gtgctataaa

atgccacaat taaatcaatg atactattta ttgttgtttt tagcttatca aaatctgttc

atcttttaat tttgtcaat- --------tg actcacagac attaccccag ctagcactgc

caacaactcc taaaaagtta ctaaaaagag ttggaaagct ttgtgacac

>404LG7

---------- ---------- ---------- ---------- ---------- ----------

---------- ---------- ---------- ------tttt tagcttatca aaatctgttc

acctcttacc tttgtgaatt ga-------- ---cacacac attacgtcag ctatcactgc

taacaactcc taaaaattta ctaaaaggaa ttgaaaagct ttg------

>205LG3

ttattatatt ttctattgtg gcacgaatgg ttttggagca cgacgaagga gtg-------

---------- ---------- -cactaattt ttgtattttt tagcttatca aagtctgttc

acctcttaat tttgtcaatt gaagctattg actaacacac attaccccag ccaagactgc

caacaactcc taaaaattta ccaaaaggag ttgaaaagct ttgtgaaac

>107LG5

----tatacc ttctattgtg acacgaatgg ttttcaagca cgacgaagga gtactataga

atgccactgg taagtcatca a-----atct ttgttgtttt ttacttatca aaatctgttc

atctcctaat tttgtcaatt aacgctgttg actcacacac attaccccag ctagcattgc

caacaactcc taaaaattta ctaaatggag ttgaaaacct ttgc-----

>74aLG8

---------- -----ttgtg gtacgaatgg ttttggagca cgacgaagga gagcaa-aga

atgtcacaat taagtcaacg atactaattt ttgttgcttt tagtttatca aaaactattc

accttttaat attgtcaatt ---------- --acacacac attaacccag ccagcactgc

caaccactcc taaaaattca ctaaaaggag ttgaaaagct ttgtgacac

>74bLG8

ttactatacg ---tattatg gcacgaatgg ttttggagca cgacgaagga gtgcaa-aga

atgccacaat taagtcaacg atactaattt ttgttgcttt tagtttatca aaaactattc

acctcctaat attgtcaatt gacgctgttt acacacacat attaacccag ccagcactgc

caaccagtcc taaaaattta ctaaaaggag ttgaaaac-- ---------

>99LG9

atattatacg ttctattgtg gcacgaatgg ttttgaagca caacgaagga gtgctataga

atgccacaat taa------- -tactaattt ttgttgtttt tagcttatca aaatctgttc

-cttcttaat tttgtcaatt aacgctgttg actcacacac attactccag ccagcactgc

caacaactcc taaaaattta caaaaaggag ttgaaaagct ttgtgaaac

>269LG3

atattatacg ttctatggtg gcacgaacgt ttttgaagca cgacgaagga gtgttataga

atgccaccat taagtc---- --agtgattt ttgttgtttt tagcttttca aaatctgttc

acctcttaat ttagtcaatt gacgctgtta actcacacac attgc----- ----------

---------- ---------- ---------- ---------- ---------

>215LG10

---------- ttctattgtg gcacgaatgt ttttgcagca cgtcgaagga gtgctataga

ataccacaat taagtc---- -tactaattt ttgttgtttt tagcttatca aaatctgttt

accttttaat ttagtcaatt cacgttgttg actcacacac attaccctag ccaacattgc

caacaactcc taaaaattta ctaaaaggag ttgaaaagct ttgtgaaac

>151LG10

atattatatg ttctagtgtg gcacgaatgg ttttggagca cgacgaagga gtgctataga

atgccacaat taagtcaac- ------attt ttgttgtttt tagattatca aaatctgttc

acctcttaat tttgttaatt gacgctgtt- --tcacactc attaccccag ccagcactgc

caaaaactac taaaaattta caaaaaggag ttgaaaag-- ---------

>465LG6

ttattatacg ttctattgtg gcacaaatag ttttggagca caacgaaaga gtgctataaa

atgccacaat taactcaa-- ---------- ---ttttttt taccttatca aaatctgttc

acctcttaat tttgtcaat- ---------- ---------- ---------- ----------

---------- ---------- ---------- ---------- ---------

>57LG3

ttattatacg ttctattgtg gcacaaatgg ttttggagca cgaccaagga gtgctgcata

atgccacaat taagacaacg atactaattt ttgttg--tt tagcttatcg aaatctgttc

acttcttaat tttgtcaatt gacactattg actcacacat attattccag ccagcactgc

---caactcc taaaaattta ctaaaagtag ttgaaaagct ttagtagaa

>28LG9

ttaatatacg ttctattgtg gcacgaacag ctttggagca cgacaaagga gtgctataga

atgccacaat tgggtcaacg atactaattt ttgttgtttt tagcttttca acttctgttg

atctcttaat tttgtcgaat gccgctgttg actcactcac attaccccag ccagcactac

taacaactcc taaaaattta ctagaagaag tt-aaatgtt ttgtgaaac

>106LG8

ttattatacg ttctattgtg gcacgaatgg ttttggagca cgacgaggga gtaatataga

acgccacaat taagtcacgg atactaactt ttgttgtttt tagcttatga acatctgttc

ccctc----t tttgtcaatt gacgctgttg actcacacac attaccccag ccagcactac

aaacaactcc tgaaaattta cta------- ---------- ---------

>23LG2

ttattatatg ttctattgtg gcacgaatgg ttttggagca cgacgaagga gtgctataga

atgctacaat taagtcaaag gcacaaattt ttgtagtttt tagcttatca aaatatgttc

acttattaat tttgtcaatt gacgatgtt- attcacacac attaccacag acagcactgc

caacaactcc taaaaattca ctaaaaggag ttgaaacgct ttgtgatac

>66LG10

ttattatacg ttctattgtg gcacaaatag ttttggagca caacgaagga gtgctataaa

aggctacaac taaatcagcg atactaattt ttgttgtttt tagcttatga aaatctgttc

a-ctcttaat tttgtcaatt gacgctgttt actcacacac attatcccag ccagcactgc

caacaactcc aattaattta ctaaaagaag ttgaaaaact ttgtgaaac

>252LG6

ttattgtatg ttctattggg gcacgaatgg ttttagagca cgacgaagga gtaccataga

atgctttagt taagtcaacg atactagttt ttgttgtttt tatcttatca aaatttataa

actactta-a tttgtcaatt gacgctgttg actcacacac attac----- ----------

---------- ---------- ---------- ---------- ---------

>22LG8

ttattatacg ttcttttgtg gcacgaatgg ttttggagca cgacgaagga gtgctaaaga

atgccacaat taagtcaacg ataccaatt- ---ttggttt tagcttatca acatctgtcg

a---cttaat tttgtcaatt aaccctgttg actcacacac attaacccag ccagcagtgc

caacaactcc taaaaatttg ctaacaggtg ttgaaaagct ttaggaaac

>71LG8

atattatacg ttctgttgtg gcacgaatgg ttctggagca cgacgaagga gagctataga

atgccacaat taagtcagcg atactaattt ttgttatttt tagcttataa aaatctgttc

acgtcttcat tttgtcaatt gacgcagttg actcacacac attaccccat tccgcactgc

caacaactcc taaaaattta tttaaaggag ctgtaaagct ttgtgctac

>64LG2

ttattatacg ttctattgtg gcacaaatgg ttttggagca cgacgaacga gtgctataga

atgcctcaat taagtcaacg atactaattt ttg----ttt tagcttatca aaatctgttc

acctcttaat tttg--aatt gacggtgttg agtcacatac attaccccag ccagcactac

caacaactcc taaaaattta ctaaaaggag ttgaaaag-- ---------

>88bLG2

ttattatacg ttctattgtg gcacgaatgg ttttggagca caacgaagaa gtgctataga

atgtctcaat taagttaact atactaattt ttg----gtt tagcttatca aaatatgtt-

-cctcttaat tttgtcaatt gacgctgttg actcgcacat attaccccag ccagcgcagc

caacaacttc taaaaattta ctaaaaggag ttgaaaag-- ---------

>33aLG2

ttattatacc ttctatagtc gcacgaatga ttttggagca cgacgaagga gtgttacaga

atgccacaat taagtcaacg atattaattt ttgttgtttt aagcttatca aaatctgttc

accttttaat tttgtcaatt aacgctgttg actcacacac attaccacag caagcactgc

caacaactcc taaaaattta t--aaaggag ttgaaaagct ttgtgaaac

>154LG4

ttaatatacg ttctat--cg gcacgaatgg ttttgaagca cgacgaagga gtgctaaaga

atgttacaac taaatgaacg acactagttt tggttgtttt tagcttatca aaatctgttc

atctcttaat tttgttaatt gaccctcatg ccttacacac attaccctaa gcagcactgc

caacaactcc taaaaatttg ctaaaaggag t--------- ---------

>45LG4

tatatttacg ttctattgtg gcacgaatgg ttttggagca cgacgaaggg gtgctataga

atgccacaag taagtcaacg atactaattt ttgttgtttt tagcttatca aaatctgtt-

-cctcttaat tttgtcaatt tacgatattg ------acac attaccccag ccagtaccgc

caacaactcc taaaaattta ctaaaaggag ttgaaaagct ttgtgaaac

>199LG3

----tatacg ttctagtgtg gcacgaatgg ttttggagca cgacgaagga gtgctacaaa

atgccacaat ggattcaacg atactaattt ttgttgttat tagtttatca aaatctgttc

accttttaat tttgtcaat- ---------- ------acac agtatcccag ccagcactgc

caacaacttc taaaaattta ctaaaagtag ttgaaaagct tt-------

>417LG6

---------- ---------- ---------- ---------- ---------- ---------a

aggctacaat taagtcaacg acactaattt ttgttgtt-- ---tttatca aaatctgttc

acctcttaat ttcgtcaatt ---------- -------gtc attatcctac ccagcactgc

caacaactcc taaaaattta ctaaaaggag a--------- ---------

>288LG4

tatttttacg ttctattatg gcacgaatag ttttggagca ccacgaagga gtgctgtaga

atgccacagt taagtgaacg acactaattt ttgttggttt taa------- aaatctcttc

acctcttaat tttgtcaatt gaccctgtt- ---------- ---------- ----------

---------- ---------- ---------- ---------- ---------

>41aLG9

ttattatacg ttctgttgtg gcacgaatgg ttttggagca cgacgaagga gtgctatcaa

atgccacaat caagtcaacg atactaattt tttttgtttt tagtttatca gaatctgttc

atcctcttat tttattaatt gacgctgttg attcacacac attaccccag ccagaactac

taacaactcc taaaaactca ctaaaaggag ttgaaaag-- ----gaaag

>224LG2

ttattatacg ttctattgtg gcaggaatgg ttttggagca cgacgaaaga gtgctataaa

atgccacaat gaagtcaacg acactaattt ttgttgttta aagcttatca aa--------

-cctcttaat tttgtcaatt gaccctgttg --atccacac attca----- ----------

---------- ---------- ---------- ---------- ---------

>51LG9

ttaatatacg ttctattgtg gcacgaatgg ttttagagca cgaaggagga gtgctataga

atgttacaat taagtgatcg aaactaattt ttgttgtttt aagcttatca aaatctattc

acgtcttaat tttgacaatt gacgctgttg actcatacac attac----- ccagcactgc

caacaacttt attgaattta ctaaaagcag ttgaaaagct ttgtgaaac

>177LG4

ttattatacg ttctattgtg gcacgaatgg ttttagagca cgacgaagga gtgctatgaa

atgccacaat taagtctacg atactaattt ttgttgtttt aagcttatca aaaactgtt-

-tctcttaat tttg--acat gacgctgtta actcacacac attac----- ----------

---------- ---------- ---------- ---------- ---------

>49aLG3

ttattatacg ttctattgtg gcacgaatgg ttttggagca cgacgaagg- -------cga

atgccacaat taagtcaacg atactaatgt atgt--tttt tagc---taa aaatatgttc

atctcttaat tttgtcaatt ggcgctattg gctcatacac attaccccag ccagcactgc

caacaacttc taaaaactta ctaaaaggaa ttgaaaagct ttgtgaaac

>52LG3

ttattatacg ttttattgtg gtacgaatgg ttttggagca cgacgaag-- ----------

--gccacaat gaagtcaacg atactaattt ttgttgtttt taacttatca aaatctgttc

acctctgaat tttgtcattt gaccctgttg actcacacac attactccaa ctagcactgc

caacaactcc taaaatttta ctaaaagtag ttgaaaagct ttgtaaaac

>60LG3

ttattatacg ttctattgta gcacgaatgg ttttggagca cgacaaag-- ----------

--gccacaat taagtcaacg atactaattt ttgttgtttt tagcttatca aaatctgttc

acgtcttaat tttg----tt gacgatgtcg agtcatacac attaccccag ccagcactgc

caacaccttc caaaaattta ctaaaagcag ttgaaaagct ttgt--gac

>378LG4

ttattatacg ttctattgtg acacgaatgg ttttggagca cgacgaag-- ----------

--gtcacaat taagtcaacg ataccaattt ttgttgtttt t--cttatca aaatctgttc

acctcttaat tttg------ ---------- ---------- ---------- ----------

---------- ---------- ---------- ---------- ---------

>336LG6

---------- ttttattgtg gcacgaatgg ttttggagca cgacgacg-- ----------

--gccacaat taagtcaacg atactaattt ttgttgtttt tagcttatca aaatctgttc

accacttaat tttatcaat- ---------- ---------- ---------- ----------

---------- ---------- ---------- ---------- ---------

>242LG3

ttattatacg ttcgattgtg gcacgaatgg ttttggagca cgacgaaa-- ----------

--gctacaat taactcaatg atactaattt ttgttatttt tagcttatcg aaatatgttc

acctcttaat tttgtcaatt gacgctgttg ac-------- attaccccag tcagcattgc

caacaactcg taaagattta ctaaaaggag ttgaaaag-- ---------

>72LG3

atattatacg ttctattgtg gcacgaatga ttttggagca cgacgaag-- ----------

--gctacaat taagtcaacg atagtaattt ttgttgtttt tcactaatca aaatctgttc

aactcttaat tttgtcagtt cacgctgttg actcacacac attaccccag ccagcactcc

caacaactcc taaaaattta ctaaaagaag ttaaaaagct ttaaggaac

>176LG3

tttttataca ttctattgta acacgaatgg ttttggagca cgacgaag-- ----------

--gccacaat tacgttaatg atactaattt ttgttgtttt --gcttatta aaatctgttt

acctcttaat tttgtcaatt gacgctgttg actcacacac attaccctag ccaacactgc

caacaactcc taaaagttta ctaaag--ag ttgaaaagat ttgtgaaa-

>298LG4

ttattatacc ttctactatg gcacgattgg ttctggagca cgacgaag-- ----------

--gccaatat taagtcaacg atactaatgt ttgttgttgt tagcttatca aaatctgttc

acctcttaat tttgtcgact gccgctgttg actcactcat attat----- ----------

---------- ---------- ---------- ---------- ---------

>160LG5

ttattatacg ttttattgtg gcgcgaatag ttatcaagtt ataagaat-- ----------

--gccacaat taagtcaact atactaattt ttgttgtttt tagcttatca aaatctgttc

acctcttaat tttgtcaatt ggcgctgttg actaacacac a----cccag ccagcactgc

caacaactcc taaaaactta ctaaaagaaa aaaaaaagct ttgtgaaac

>109LG8

ttattataca ttctattgtg gcacgaatga ttttggagca cgacgaat-- ----------

--gccacaat taagtcaacg acactgattt ttattgtttt tagcttatca aaatctgttc

actacttaat tttgtcaatt gatgctgttg actcacacag atcaccccag ccagcactac

caataactcc tgaaaattca ctaaaaggag ttgaaaagct ttgtgaaac

>120LG8

atattataca ttctattgtg gtacgaatgg ttttggagca cgacgaat-- ----------

--gccacaat taagtgaacg atactaattt ttgttgtctt tagcgtacct atatctgttc

acttattcat tgtgtcaatt gacgctgttg actcacacac attatcccag tcagcactgc

taacaactcc taaaaattta ctaaaaggag ttgaaaagct ttgtgaaat

>124LG9

ctattataca ttctattgtg acacgaatgg ttttggagca cgacgaagg- -------aga

ctgccacagt taagtcaacg atgctagttt ttgtactttt tagtttatca aaatctgttc

acctctttat tttgttaatt gacgttgtta acacacacac attattccag ccagaactgc

caacaacgcc taaaaatgtt ctaaaaggag ttgaaaagct tttagaaac

>85LG3

ttattatacg tttttttgtg gcacgaacgg ctttagagca cgacgaag-- ----------

--gccacaaa taagtcaacg atactattta ttgttgtttt aagcttttca aaatctattc

aacttttaat tttgtcaatt gatgctgttg actcacacac attacctcac ccagcactgg

caacaactct t-aaaattta ctaaaagcag ttgaaaagct gtgtgaaac

>366LG7

ttattatacg ttctattgtg gcacgaacgg ttttagagca cgacgaag-- ----------

--gccacaat taagtcaacg atacta---- ------tctt tagctt---- --atctgttt

acctcttaat tttgtcaatt gacgctgttg actcactcac attac----- ----------

---------- ---------- ---------- ---------- ---------

>340LG3

ttattatacg ttctattgtg gcacgaatgg ttttggagca tgacgaag-- ----------

--gctacaat taggtcaacg atattaatct ttgtagtttt tagcttatca aaatctgttc

a---cctaat tttgtcaact g--------- ------acac attac----- ----------

---aatttac taaaaattta ctaaaaggag ttgaaaagct ttgtgaatc

>383LG3

ttattatacg ttgcgttatg gcatgaatga ttttggagca cgacgaag-- ----------

--gccacaac taagtcaacg atactaattt ttgtggttat tagcttatca aaatcta---

acctcttaat tttgtcaat- ---------- ---------- ---------- ----------

---------- ---------- ---------- ---------- ---------

>322LG9

ttattatacg ttctattatg gcacgaatgg ttttggagca cgacgatg-- ----------

--gctacaat taagtcaacg atactaattt ctgttgtttt tagcttatca aaatctgttc

acctcttaa- tttgtaaatt gacgctgttg actcac---- ---------- ----------

---------- ---------- ---------- ---------- ---------

>118aLG3

ttattatacg ttttattgta gcacgaatgg ttttagagca cgacgaagga at--------

-tgcctcaat taggtcagcg ttactaattt ttattgtttt tagttgatca aaatctgttc

acctcttaat tttgtcaatt gacgcttttg actcacacac gttacctcag ccagcactgc

caacaactcc tataagttta ctaaaaggag ttgaaaattt ttgtgaaa-

>287LG7

ttattatacg ttctattgta gcacgaatag ttttggagca cgacgaag-- ----------

--gccacaat caggtcaacg atactaattt tagttgtttt tagcttatca gaatctgttc

acctcttaat tttgtcaatt gacgctgttg actcacatac attac----- gcatcactgc

caacaactcc caaaaattta c--------- ---------- ---------

>291LG5

ttattatacg ttctattgtg gcacgaatga ttttgaagca cgacgcagga ----------

--gtctgaat taagtcaacg atactaattt gtgttgtttt tagtttagca aaatcagttc

acctcttaat tttgtcaatt gacacggttg acccacatac attac----- ----------

---------- ---------- ---------- ---------- ---------

>185LG3

ttattataca ttctgttgtg gcacaaaagg ttttggagca cgtcgaag-- ----------

--gccacaat taagtctacg atgccaattt ttgtcgtttt taccttatca aaatctgtta

atctcctaat tttgtcaatt gacgcctttc attcacacac attattccag ccagcactgc

taacaactcc taaaaattca ctaaaaggag ttg-----ct ttgtgaaac

>311LG3

atattatacg ttctattgtg ccacgaatga ttttggagca cgacgaag-- ----------

--gccacaac t--gtcaacg atactaa--- --------ca tagcttatca aaatctgttc

acttcttaat tttgttaatt gacgctgttg actcacacat aataccccag ccggcactgt

caacgactcc taaaaattta ctaaaagtag ttgaaaagct ttc------

>282LG3

---------- ttctattgtg gcacgaatag ttttggagca cgacgaaa-- ----------

--gccacagt tgagttaacg atactaattt ttgttatttt tagtttatcg aaa----ctt

acctcttaat tttgtcaatt gacgctgttg actcacacac attaccccag ccagcactgc

caacaactcc caaaaattta ctaaaac--- ---------- ---------

>394LG9

ttatcaaacg ttctattgtg gcacgaatgg ttttggagca cgacgaag-- ----------

--gccacagt taagttaacg atactaat-- ------tttt ttacttatga aaatctgttc

acttattaat tttgtcaatt gacactgttg agacact--- ---------- ----------

---------- ---------- ---------- ---------- ---------

>302LG3

ttgttatacg ttctattgcg gcaccaatgg ttttggagca cgaccaag-- ----------

--gccccaac taagtcaacg atactaa--- --------tt tagcttgtca aaatctgttc

acctcttaat tttgtcaatt gacgctgtga ------acac attaccccag ccagcact--

-aacaactcc taaaaattta ctaaaaggag ttg-----tt ttgtaaaac

>380LG10

atatagtata ttcaattgta tcacgaatgg ttttggagca cgacgaag-- ----------

--gtcacaat taagtcaacg atactaagtt ttgttg--tt tagcttatca aaatctgttc

atctcttaat tttgtcattt ggcg------ ---------- ---------- ----------

---------- ---------- ---------- ---------- ---------

>132LG3

ttattatacg ttttattgtg gcacgaatgg ttttggagca cgacgaagga gtgcaatgga

atgccataat taagtcaacg atac-aatta atgttgtttt tggcttatca aaatctgttc

acttcttaat tttgtcgatt gacgctgttg actcactcac attaccccag ccagcgctgc

caataactcc taaaaattta ctaaaaggat ttaaaaagct ttgtgaaac

>143LG5

tgattataca ttatattgtg gcacaaatgg ttttggagca cgacgaagga gcgctataga

atgcttcaac taaatcaacg atactaattt ttgttgtttt tagcttatca aaatgtgttc

acctcttaat tttgccaatt gacgctgttg ac--aatcac ataaccctag ccaacactgt

caacaattcc caaaaattta ctaaaagaag ttaaaaagat ttgtgaaac

>324LG7

ttattataca ttctattgca gcacgaatgg ttttggagca cgacgaagaa gtactgtaga

tt-------- ---gacaacg atactaattt ttgttgtttt cagcttatca aaatctgttt

ccctcttaat tttgttaatt a--------- ---------c ---------- ----------

---------- -----gctta ctaaaaggag ttgaaaagct ttgtgaaac

>334LG8

ttattataca ttctattgtg gcacgaatgg ttttggagca tgacaaagga gtgctataga

aa-------- -----gaacg ttactaa-tt ttgttgtttt tagcttatca aaatctgttc

acttcttaat tttg------ ---------- ---------- ---------- ----------

---------- ---------- ---------- ---------- ---------

>29LG3

-tattatgcg ttctattgtg gcacaaatgg ttttagagca cgacaaagga gtgctataca

atgccacaat taagtcaacg atact-attt ttgttgtttt taacttatca aaatctgttc

acctcttaat tttgtcaatt -acggtaata actcacacaa attaccctag ccagcactgc

caatctcctt taagaattta ctaaaaggag ttgaaaagct tcgtgaaac

>58LG3

---------- ttctattgtg gcacgaatgg ttttagagca cgacgaagga atgctaaaga

atgccacaat taagtcaacg atactaattt ttg-tgtttt tagcttttta aaatttg---

-ccttttaat tttgtcaatt gaggctgttg actcattcac ataaccccaa ccagcactgc

caacaactct taaaaaatta ctaaaaggag ttgaaaagct ttgtgaaac

>209LG7

---------- ---------- ---------- ---------- ---------- -----atagt

atgccacaat taagtcaacg atactaattt ttgttgattt taa------- ----------

-cctcttaat tttgtcaatt gacgctgttg actcacccac attaccccag ccagcactgc

caacaactcc taaaaattta ctaaaaggaa ttgaaaagct ttgtgaaac

>373LG7

---------- ---------- ---------- ---------- ---------- ----------

---------- ---------- ---------- ---------- ---------- ----------

-cctcttaat tttgtcaatt gacgctgttg ac-------- atgaccctaa ccagcactgc

caacaactcc taaaaattta ctaaaaggag ttgaaaagct ttgtgaaac

>235LG3

---------- ttctattgtg gaacgaatgg ttttggagca cgacaaaaga gtt-------

--agttaagt taagtcaacg ataccaattt ttattgtttt caa------- ----------

-cctcttatt tttgttaatt gacgctgttg actcacacac attaccccag ccagcactgc

caacaactct taaaaattta ctaaaaagag ttgaaaagct tagagaaac

>142LG4

-cattatacg ttctattgca ctacgaatgg ttttggagca cgacgaag-- ----------

--gccacaat taagtcaacg atactaactt atgttgtttt ta-catttca aaatctgttt

acctctcaat tttgtcattt gacgctgttg actcacacac attaccccaa ccagcagtgt

caacaactcg taaaaattaa ctaaaaggag ttaaaaagtt ttatgaaac

>90LG5

---------- ---gattgtg gcacgaatgg ttttggagca cgacgaagga gtc-------

--tataaaat taagtcaacg atactaattc ttgttgtttt taccttatca aaatctgttc

acctcttgat tttttcaatt gacgctgttg acccacacac ataagcccag ccagcactgc

caacaactca caaaaattta ctaaaaggag ttgaaaagct ttgtgaaac

>208LG4

---------- ---------- ---------- ---------- ---------- ----------

---------- ---------- atactaattt ttgttgtttt tagcttatca aaattttttc

acctcttaat ttcgttaatg cacgctgttg attcacacac atgactccag ccagcactac

caacaact-- -aaaaattta ttaaaaggtg ttgaaaagct ttgtgaaac

>147LG5

---------- ---------- ---------- ---------- ---------- ----------

---------- ------aacg atactaattt ---ttgtttt tagcttatca aaatctgttc

acctcttaat tttgtcaatt gacgctgtta actcacacac attaccccag tcagaactgc

caacaactcg taaaaatttc ctaaaagaag ttaaaaaatt ttgtgaaac

>233LG8

----tatacg tcctattgtg acacgaatgg ttttggagca cgacgaag-- ----------

---------- ---------g atactaattt ttgttgtttt ttgtttatca aaatctgttc

acctcttaat gttgtcaatt gacgctttag acttacacac attaccccag ccagcacatc

caagaact-- -----atcta ctaaaagtaa ttgaaaagct ttgtgaaac

>236LG2

---------- ---------- ---------- ---------- ---------- ----------

---------- ---------- ---------- --------tt ttgtttatca aaatctgttc

acctcttaat gttgtcaatt gacgctgttg act--cacac attaccccag ccagcactgc

aaacaacacc taaaaattta ctaaaaaaag ttgaaaagct ttgtgaaac

>50LG8

--attatacg ttctattata gcacgaatag ttttagagca cgacgaagga gtgctctaaa

atgccacaat taagtcaa-- ataccaattt ttgttgtttt tagcttatca aaatctgtta

acctcttaat tttgtcaatt gacgctgttg att--gacac attaccccag ccagcactgt

caacaacttc taaaaattta ctaaaagcag ttgaaaagct tggttaaac

>174LG9

---------- ntctattgta gcacgcatga ttttggagca cgacgaagga gtgttataga

atgccacaat taagtcaacg atacttattt ttgttgtttt taacttatca aa--------

-cttctttat tttgttaatt gacgctgttg actcacacac attactccag tcagctctgc

caacaactac taaaaaatta ctaaa----- ---aaaagct ttgtgaaac

>175LG3

---------- ---------- ---------- ---------- ---------- ----------

---------- ------aagg atactaattt ttgttgtttt ta-tttatca aaatctgttg

acctcttaat tttgtcaatt gacgctgtag attcacacac attaccccag ccagcactgc

caataactcc taaacattta ctggaaatag ttcaaaagct ttgtgaaac

>359LG9

---------- ---------- ---------- ---------- ---------- ----------

---------- ---------- atactaattt ttgttgtttt tagtttatca aaat------

-cctcttaat tttgtcaatt gacgctgttg actctgacac attacctcag ccagcactgc

cac------- ---------- ---------- ---------- ---------

>305LG10

---------- ---------- ---------- ---------- ---------- ----------

---------- ------gacg atactaattc ttgttgtttt tagcttatca aaat------

-cctcttaat tttgtaaatt aaggctgttg acacacactc attatcccag ccagcactgc

caacaactcc taaaaaatta ctaaaaggag ttgaaagt-- ---------

>317LG4

---------- ---------- ---------- ---------- ---------- ----------

---------- caagttgacg atattaattt ttgttgtttt tagcttatca aaatctg---

-tctcttaat tttgtcaatt gacgctgttg actcacacac attatcccag acagtactac

caacaactcc taaaaattta ctaaaaagag ttgaaaagct t--------

>326LG6

---------- ---------- ---------- ---------- ---------- ----------

---------- ---------- -tactatttt ttgttgtttt tagcttatc- ----------

-cctcttaat tttgtcaatt gacgctgatg actcacacac attacaccag ccagcactgc

caacaactcc taaaaattta ctaaaaagag ttgaaaag-- ---------

>226LG8

ttattatacg gtttgttctg gcccgaatgg tttt------ ---------- ----------

---------- ---------- ------attt ttgttgtttt taacttatca aaatctgttc

acctcttgat tttgacaatt gacgctattg actcacatac attaccccag tcagcactgc

caataacccc ttaaaattta ctaaaatgag ttgaatagct ttgtgaaac

>59LG3

ttattatacg ttctattctg gcacgaatgg ttttggagca cgacaaagga gtgctataaa

atgctagaat taagtcaaat atacaaattt ttgttgtttt cagcttatga atatctgttc

acctcttaat tttgtcaatt gacgctattg actcacacac attaccccag tcagcactgc

caacaactcc taaaaattta ctaaaaggag ttgaaaagct ttcttaaac

>92LG3

ttattatacg tcctattgtg gttcgaatag ttttagagca cgacgaagga gtgctataga

atgccacaat taagtc---a atactaattt ---ttgtttt tagtttatga aaatctgttc

acctcttaac tttcccaatt gacgctgtag actcatacaa attactgaca ccagcactgc

caacaactcc taaaaattta ctaaaactag ttgaaaagct ttgt-----

>111LG7

ttattaaacg ttt------- -cacgaatgg ttttggagca cgacggagta gtgctataga

ataccataat taagtcaacg atactaattt ttgttgtttt aagcttatca aaatctgttc

acctcttaat tttgtcaatt gacgctgccg actcacacac attaccccag ccagcactgc

caacaactcc taaaaattta ctaaaagaag ttgaaaagct ttgtgaaac

>133LG6

ttattataca ctctatggtg gctcgaatgg ttttggagca cgacgacg-- ----------

--gccacaat tacgtcaact atactaattt ---ttgtttt taggttatca aaatctgttc

acctcttaat tttgtcaatt ggcgctgtta actcacacac attaccttag ccagcactgc

caacaaatac taaaaattta ctaaaaagag ttgaaaagct tcgtgaaaa

>278LG9

---------- ---------- ---------- ---------- ---------- ----------

---------- ---------- -tactaattt ---ttgtttt tagtttatca aaatctgttc

acctcttaat tttgccaatt gacgctgttg acacacacac gttaccctag ccagcactgg

caacaactcc taaaaattta ctaaaaggag ttgaaaagct ---------

>137LG3

---------- ---------- -----aatag ttttggagca cgacgacaga gtgctataga

atgccacaat taagtc---- -tactaattt ttgttgtttt taacttatca aaatctgttc

acttcttaat tttgtcaatt gacgttgttg actcacacat attaccctaa ccagcactgc

caacaactcc aaaaaaatta ctaaaaggag ttgaaaagcc ttgtgaaac

>196LG3

---------- --------ag gcacgaatgg ttttggagca caacaaagga gtgctataga

atgtcgcaat taagtc---- -cactaatat ttgttgtttt tggtttaaca aaatctgttc

atttttaaat tttgtcaatt gacgctgttg actcacacac attatctcag ctagcactga

caacaactct taaaaattta ctaaaaggag ttaaaaagct ttgtgaaac

>135LG8

ttattatatg ttc---tgtg gcacaaatgg ttttggagca cgaagaagga gtgctataga

ataccacaat taagacaacg aaactaattt ---ttgtttt tagctcatca aaatctattc

acctcttaat tttgtcaatt gatgttgttg actcacaccc attaccccag taagcactgc

taacaacttc taaaaattta ctaaaaggag ttgaaaag-- ------aac

>240LG3

ttatcataag ttctattgtg acacgaatgg ttttggagca cgacgaagga gtg-------

---------- ------aacg attctaattt ttgttg--tt tagtttatca aaatctgttc

accacttaat tttgttaatt gacgcttttg atttatacac attaccccag ccagcattgc

caacaacttc taaaaattta ctaaaagaag ttgaaaaact ttgtgaaac

>93LG2

---------- ttctattgtg gcacggatgg ttttggagca cgacgaagga gtgctgtaga

acgccacaac taaatcaacg atactaattt t-------tt tagcttatca aaatctgctg

acctcttaat tttatcaatt gacgctgttg actcacacac attacgacag tcagcgttgt

caacaactcc taaaaatttg ctaaaaggag ttgaaaagct ttgtgaaac

>78LG2

ttattatacg ttcaattgt- gtacgaatgg ttttagagta cgacaaaaga gtgctataga

atgccgcaat taagtcaacg atattacttt ttgttgtttt tagcttagca aaatctgttc

acctcttaat tttgtcaatt gacgctgttc actcacacac attaccactg tcagctctgc

caacaacttc caaaaattta ctaaaaggaa ttgaaaagct ttttaaaac

>198LG10

---------- ---------- ---------- ---------- ---------- ----------

---------- -aagtcaacg attataagtt ttgttgttta tagattgtca aaatctgttc

atctcttaat tttgtcaatt ggcgctgttg actcacacac attac----- ccagtactgc

caacaactct taaaaattta ctaaaaggag ttgaaaagct ttgtgaaac

>316LG8

---------- ---------- ---------- ---------- ---------- ----------

---------- ---------- ---------- ---------- ---cttatca aaatcggtta

acctcttaat tttgtcaatt gacgctgttg actcacacat attac----- ---gtactga

cagttgtcaa taaaaattta cttaaaggag ttgaaaagtt ttgtgaaac

>273LG6

ttattatacg ttcttttgtg gcacgaatgg ttttggagca cgacgaagga ----------

---------- ---------- -tattaattt ttgttgtttt tagcttatta aaatttgttc

accttttaat tttgttaatt gacgctgttg actcacacac attac----- agtacactgc

cagcaactcc aaaaaatttg ctaaaagaag ttgaaaagct ttgtaaaac

>113LG8

---------- ttctattgtg gcacgaatgg ttttggagca cgacaaagga gtgcaataga

atgccgcaat tacgtcaact atacaaattt ttgatgtttt caccttatca aaatctgttc

acctcttaat tttgtcaatt gacgctgata actcatacac attaccccag gctgaactgt

caacaacttc taaaaattta ctaaaaaaag ttaaaaggct ttgtgaaac

>61LG3

---------- ---------- gcatgaatgg ttttggagca cgacaaagga gttctataga

ttgccacaat taagtcaacg atactaattt ttgttgtttt tagcttatca aaatctgttc

acctcttaac tttgtcaatt gacgctgttg actcacacac attacctcag tcagcactgc

caacaactcc taaaaattta ctaaaaggag ttg-aaagct ttgtgaaac

>84LG9

ctattatacg ---tgttgtg gcacgaatga ctttggagca cgacgaagga gtgctataga

atgccacaat gaagtcaacg atattaattt ttgttgtttt tagcttttca taatctgttc

acttcttaat tttgtcaatt gacgctgttg actcatacac attatcctaa ccagcactgc

caacaactca tcaaaattta ccaaaaggag ttaaaaagct ttgtgaaac

>44LG5

ttattatacg ttctattgta acacgaatgg ttttggagca cgacgaagga gtgctataga

atgccacaa- ttactcaacg atactaattt ttgttgtttt tagcttatca aaatctgttc

a----taaat tttgt-aatt aacgctgtag actcacacac attaccccag ttagcactgc

cagtaacttc taaaaatttg ct-aaagaag ttgaaaagct ttgtga---

>303LG2

ttattatacg ttctattgtg gcatgaatgg ttttggagca cgacgaagga gtgctataca

ttgccacaa- taagtcaacg cttttaattt ttgttgtttt taactgatca aaatctgttc

acctcttaat tgtg------ ---------- ---------- ---------- ----------

---------- ---------- ---------- ---------- ---------

>275LG10

ctattatacg ttctattgcg gcatgaatgg ttttggagca cgacgaagga gtgctaaaga

atgtcacaat taagtcaacg ataccaattt ttattttttt tagcttatca aaatctgttt

acttcttaat tttg-----g gacgctgtag actgacacac attac----- ----------

---------- ---------- ---------- ---------- ---------

>179LG5

ttactatacg ttctattgtg gcacgag--- ---------- ---------- -ttttataga

atgccac-at taagtcaacg atactagttt ttgttgtttt tagtttatca aaatctgttc

acctcttaat tttgtcaatt gaccctgtag acttacacaa attac----- atgacactgc

taataactcc taaaaattta ctaaaagtag ttgaaaagct ttgtgaaac

>337LGX

ttattatacg ttctattgta gcac------ ---------- ---------- -----ataga

atgccataac taagtcaaca atactaattt ttattgtttt tagcttatca aaatctgttc

acctcttatt tttttcaatt gacgctgttg acctacac-- ---------- ----------

---------- ---------- ---------- ---------- ---------

>285LG6

ttattatacg ttctattgtg gcacgaatga tttta----- ---------- ----------

---------- taagtcaacg atactaattt ttgttatt-- ---cttatca aaatctgttc

acctcttaat tttgtcaatt aacgctgttg actcacacac attaccccag ccagcactgc

caacaactcc taaaaattta ctaaaaggag ttgaaaagct tt-------

>95LG2

ttattatacg atctactggt gcacgaaagc tttaggatca cgacgaagga gtgctataga

atgccacaat taagtcaacg atactaattt ttgttgtttt tagcttatca aaatctgttc

ccctctaaat tttgtcaatt gacgctgttg actcacacac attaccccag ccagcaatgc

caggaactcc taaaaattta ctaaaagaag ttgaaatgct ctgtaaaac

>213LG3

---------- ---------- ---------- ---------- ---------- -------cga

atgccacaat taagtcaacg atactaattt ttattgtttt aagcttatca aaatgtgttc

tcgttttaat tttgtcaat- aacgctgttg actcacacac attaccccag ccagcattgc

caacacctct gaaaaattta ctaaaaggag tggaaaagct ttgtgaaac

>202LG4

---------- ---------- ---------- ---------- ---------- -------cga

atgcctcaat tcagtcaacg atactaattt ---ttgtttt tagcttatca aaatctgttt

acctcttaat tttgtcaatt gacactgttg agtcacgcac attaccccag ccagcactgt

caacaacttc ataaaattta ctaaaaggag ttgaaaagct ttgtgaaac

>306LG7

---------- ---------- ---------- ---------- ---------- ----------

--gccacaat taagttaatg atacaaattt ttgttgtttt tggcttatca aaatctgttc

acctcttaat tttgtaaatt gccgctgttg actcacacac attacctcaa cgagcgctgc

caacgacttc taaaaattta ctac------ ---------- ---------

>134LG3

ttattatacg ttctattgtg gcacgaa--g ttttggagca cgacgaag-- ----------

--cccacaat gaagtcaacg atactaagta ttgtcgtttt taccttatca gaatctgttt

acctcttggt tttgtcaatt gatgctgttg actcac-cac attaccctcg ccagcactgt

caacaactcc taaaaattta ctaaaagtag ttgaaaagct gtgtgaaac

>161LG10

ttattatacg ttctattgtg gcacgaa--g ttttggagca cgacgaag-- ----------

--cccacaat gaagtcaacg atactaagta ttgtcgtttt taccttatca gaatctgttt

acctcttggt tttgtcaatt gatgctgttg actcac-cac attaccctcg ccagcactgt

caacaactcc t-aaaattta ctaaaagtag ttgacaaact gtgt-gaac

>138LG2

---------- ---------- ---------- ---------- ---------- -------cga

atgccacaat taggtcaacg atactaattt tttctatttt tatcttatca aaatctgttc

acttctcaat tttgtcaatt gccgctgttg actcacacac attaccccag ccagcactgc

caacaactcc taaaaattta ctaaacgtag ttaaaaagct ttctgcaac

>231LG5

---------- ---------- ---------- ---------- ---------- ----------

---------- ----tcaacg atactaattt ttcttgtttt tagcttagca aaatttgttc

acctcttaaa tttgtcaatg gcagctgttg actcacacac attacctcag ccagcactgc

caacaacttc taaaaattta ctaaaaagag ttgaac---- ---------

>81bLG8

ttattataca --------tg gcacaaatgg ttttggagca cgacgaaaaa gtgcaataga

atgccacaat taagtcaatg ataataattt ttg------- ---ttttgca aaatgtgttc

acctcttaat tttgtcaatt gacgctgttg acttacacac atgaccctag ccagcactgc

caacaacttc taaaaattta ctaaaaagag ttgaaaaggt ctgtgaaac

>218LG9

---------- ---------- ---------- ---------- ---------- ---ctgtaga

atgccacaat tgagtcaatg atacgaa-tt ttgtcgtt-- ---tttataa aaatctgttc

acttcttaat tttgtcaatt gacgctgttg actgacacac attaccctag ccagcactgc

caacaactcc taaaaattt- ---------- ---aaaagct ttgtgaaac

>159LG5

---------- ttctattgtg gcacgaatga ttttggagca cgacgacgga gtgctataga

ataccacaat taagtcaacg gttctaattt --------tt ttgtttttta aaatctgttc

acctcttaat tttgtcaatt gacgctgttg actcacacac attactctaa ccagcagtgc

caacaactcc taaaaattta ctaaaaggag ttgaaaagct ttgcgaaat

>183LG3

---------- ttttattgta gcacgaatgg ttttggagca cgacgaggga gtgctataga

atgtcaaaat taagtcaatg atactaattt ---atttttt tttcttatca aaatctgttc

acctcttaat tttatcaatt gacgctgttg atacacacac attactccag ctagcactgc

caacaaccct taaaaattta ctaa------ ---aaaagct ttgt--gac

>187LG9

---------- ---------- ---------- ---------- ---------- -------aga

atgccacaat taagtcaacg atacaaattt ttgtagattt tagcttatca aaatctgttc

acctcttaat tttgtcaatt gatgctgttg actcatacac gttcctccag ccagcactga

caacaactcc taaaaattta ctaaaaggaa caaaaaagct ttgtgaaac

>388LG7

---------- ---------- ---------- ---------- ---------- ----------

---------- ---------- ---------- --------tt aatattatca aaatctgttc

acctcttaat tttttcaatt gacgctgttg ac----acac attaccccag ccagcagtgc

caacaactct taaaaatttg ctaaatggag ttgaaat--- ---------

>101aLG9

---------- ---------- ---------- ---------- ---------- ----------

---------- ---------- acactaattt ttg-----tt ttgtttataa aaatctgttc

acctcttaag tttgttaatt gacgctgttg actcagacac attaccctag ccagcactgc

caacaactcc taaaaattta ct-aaaggag ttgaacagct ttgtgaaac

>101bLG9

ttattatacg ttctattgtg gcacgaatgg -tttggaaca cgacgaagga gtgctataga

atgccacaat taagtcaacg atactaattt --------tt ttgcttatca aaatctgttc

accccttaat tttgttaatt gacgctgttg actcacacac attaccctag ccagcactgc

caacaactct taaaaattta ctaaaaggag ttgaa----- ----gaaac

>271LG9

---------- ---------- ---------- ---------- ---------- -----ataaa

atgccataat taagtcaacg acactaattt ttgttgtttt tagcttatca aaatctgttc

atctcttaat ttagtcaatt ---------- actcacacac attaccccag ccagcactgc

caacaaccct taaaaattgg ctaaaaggag tttaaaaa-g ttatgaaac

>91LG3

--------gg ttctattgtg gtacgaatgg ttttggagca cgaaacagga gtgctataga

atgctacatt taagtcaacg atactaattt ttgttgtttt tagcttatca acatctgttc

acgtcttaat tttatcaatt gacgctgttg actcacacac attacccctg acagcactgc

caacaactac taaaaattta ctaaaaggag ttgataag-- ---------

>178LG8

-----ataag ttatattctg gtacgaatga gtttggagta cgacaaaaga gtgctataga

atgccacaat taagtcaaca atactaattt ttgttgtttt tagcttatca aaatttgtcc

atctcttaat gttgtcaatt gtcgct---- -ctcacacac attacgccag ccagcactgc

caacaaa--- ---------- ---------- ---------- ---------

>188LG3

---------- ---------- ---------- ---------- ---------- ----------

---------- ---------- -tactaattt ttgttgtttt ttgtttatca aaatctgtt-

-cctcttagt tttgtcaatt gacgcttttg attcacacag atta-cccag ccagcactgc

caacaactcc taaaaatttt ctaaaaggag ttgaaaagtt ttatgaaac

>247LG3

tcattatatg ttctattgag acacgaatgg ttttggagca cgacgaagga g---------

---------- ------aacg atactaatat ttgtagtttt taacttatca aaatttg---

-----ttaat tttgttaatt gacgctgttg actcacacac attaccccag ccagcactgc

caacaactcc taaaaattta ctaaaaagag ttgaaaagct ttgtgaaag

>372LG8

---------- ---------- ---------- ---------- ---------- ----------

---------- ---------- atactaattt ttgttgtttt tagctcatca aaatt-----

-cctcttaat tttgtcaagt gacgctgttg actcacacac attacccccg ctagcactgc

caacaactcc taaatatttg ---------- ---------- ---------

>193LG5

ttattatatg ttctattgtg gcacgaatgg ttttgaagca caatgaa--- -------gca

atgccacaat taacgatata ctactaattt ttgttgcttt caacttatca aattc---tc

atctcttaat tttgtcaatt gacgcttttg actcacacac attaccccag ccagctgtgc

caacaactcc taaaaattta ctaaaaggag ttg-----tt ttgtaaaac

>131LG3

ttattatacg ttctgttgtg acacgaatgg ttttggagca cgcccaaggg gtgccataga

atgccacaat taagttaacg atact-attt ttgttgtttt tg-cttatca aaatctgttc

-cctcttaat tgtgtcaatt gacgctgttg actcacacac attac----- ccagcactgc

caacaactcc aaataattta ctaaaaggag ttgaaaagct ttga-----

>139aLG7

ttattgtacg ttctattgtg acactaatgg ttttggagca cgacgaagga gtactataaa

atgcaataac taaatcaacg actattattt ttgttgtttc tagcttatca taacctgttc

atctcctaat tttgtcattt gacgctgttg attcatacac attaccccag ccagcactgc

taacaacttc taaaaattta ctaaaagaag tt-------- ---------

>219LG3

ttattatccg ttctattgtg gcactaatgg ttttggagca cgaccaagga gtgctataca

ataccacaat taagtaaacg a-tctaattt ttgctgtttt tagcttatca aaatctgttc

accgcttatt tttgtcagtc gacgctgttg actcacacat atgac----- ----------

---------- ---------- ---------- ---------- ---------

>112LG7

ttattatacg ttctattgtg gcacgaatgg ttttggagca cgacgaagga gtgctataga

atgtcacaat taagttaacg atactaattt ttgttgtttt cagctaatca aaatgtattc

accttttaat tttgtcaatt gacgc----- ---------- attaccccag ccagcactgc

cgacaactct tacaaattta ctaaaaggag ctgaaaagct ttgtggaac

>253LG2

---------- ttctattgtg acacgaatgg ttttggagca cgacaaagaa gtgctataga

atgtcacaat taagtgcacg atactaattt ttgttgtttt tagctcatca aattatgttc

acctcttaac tttgtcaatt gacgc---tg actcacaaac at-------- ----------

---caactcc taaaaattta ctaaaaagag ttgaaaagct ttgtgaaac

>276LG7

---------- ttctattgtg atacgaatgg ttttagagca caacgaagga gtgctataga

atgccacaat taggtcaacg atactatttt gtgttgtttt tagcttatca aaatctgttc

acctcttaat tttgtcaatt aatgctgtt- ---------- ---------- -----actgc

caacaactca taaaaattta ctaaaaggag ttgaaaagct ttgtgaaac

>353LG9

----tatacg ttctattgtg gcacgaatgg ttttagagca cgacgaagga gtgcaataga

atgccacaat taagtcaacg c-----atct ttgttgtttt tagtttatca aaatctgttc

acctcttaa- tttgtcaatt a--------- ---------- ---------- ----------

---------- ---------- ---------- ---------- ---------

>123LG2

ttattatacg ttctattgcg gcacgaatgg ttttggagca cgacgaagca gtgctataga

atgtcacaat taagtcaacg atacta-cta tttttgtttt tagcttatca aaatctgttc

atctcttaat tttgtcaatt gacgttgttg att--cacac attaccccag gcagaactgt

caa-tataac tacaaattta ctaaaaggag ttgaaaagct ttgtaaaac

>173LG4

ttatcatacg ttctattgtg gcacgaatgg ttttggagct cgacgaaggg gtgctataga

atgccaaaat taagtcaacg atacaa-tcc actt--tttt tagcttatca aaacgtgttc

acctcttaat tttgtcaatt gaccctgttg acccacacac attaccccag ccagcaatac

caacaactcc taaaaattta ctcaaaggag ttgaaaagat ttgtgaaac

>270LG3

ttattatacg ttctattgtg ggccaaatgg tttaagagca cgacgaagga gtgctataga

atggcacaat taagtcaaca atacta---- ------tttt taacttatca aaatctgttc

acctcttaat tttgtcaatt gacgctgttg actcacacac agtaccccag tcagcac---

---------- -----a---- ---------- ---------- ---------

>408LG3

---------- gtttatt--- gcacaaatga ttttggagca cgactaagga gtgctgtaga

atgccacaac taagtcaacg atactaattt ttgttgtt-- ---cttatca aaatctgttc

acctcttaat tttgtcaatt gacgctgttg tctcacacac attac----- ----------

---------- ---------- ---------- ---------- ---------

>327LG5

ttattattcg ttctattgtg gcacgaatgg ttttggacca cgacgaagg- ----------

atgccacaat taagtcaaca atacta---- atgttatttt tagcttatca aaatctgttt

acctcttaat tttgtcaatt ggcgctgttg actcacatac attaccctag ctagcactgc

caacaactcc tgaaaattta ctaaaaggag ttc------- ---------

>115LG7

ttattatgcg ttctattgtg gcatgaatgg ttttggagca cgacgaagga gtgttataga

atgtcacaat gaagtcaacg atactgattt ttgttgtttt ttacttatca aaatctgttc

acc---tatt tttgtcaatt gacgctgttg actcacacac attaccccag ---------c

caacaacttc taaaaattta tt-aaaggag ttgaaaagct c--------

>148LG3

ttaatatacg ttctattgtg gcacgaatgg ttttggagca cgacgcagga gtgctacaga

atgccac--- --agtcaacg atgcaaattt ttgttgtttt ta-cttttca gaatctgttt

-cctcttaat tttgtcaatt gacgctgctg actcacacac atgac----- ----------

---caactcc taaaaattta ctaaaaagag ttgaagggtt ttgtgaaac

>265LG3

-----atacg ttctgttgag gcacgaatgg ttttggagta cgacgaagga gtgtc----a

atttaactct taagtaaacg atactgattt ttgtcgtttt ttgcttatca aaatctgttc

atcttttaat tttgtcaatt gacgctgttg actcacactc ataac----- ----------

---------- ---------- ---------- ---------- ---------

>217LG7

ttattatact ttatattgtt gcacgaattg ttttggagca caacgaagga gtgct-----

-----ccaat taagttaacg atactaattt ttgttgtttt tagcgtaata acatctg---

-cctcttaat tttgtcaatt gacgctgttg actcacacac attaccccag cctgcactgc

caaccactcc taaaaattta ccaaaaggag ttgaaaaaca ttgtgtaac

>386LG9

ttattatgcg ttctattgtg gcacgaatgg ttttggagca cgacgaag-- ----------

--gtcacaat taagtcgacg atactaattt ttgttgtttt taacttatt- ----------

-cctcttaat tttgtcaatt aacgctgttg actc------ ---------- ----------

---------- ---------- ---------- ---------- ---------

>304LG3

ttattatacg ttctattgtg gcacgaatgg ttttggagca cgatcaaaga gtgta-----

-------aat aaataaaacg atactaattt ttgttgtttt tagct----- -aaattgttc

atctcttaat tttgacaatt ggtgctcatg actaatacac attaccccag ccatcactgc

caacaact-c taaaaattta gtaaa----- ---------- ---------

>331LG4

ttattatacg ttctattgca gtacgaatgg -tttggagca cgacgaag-- ----------

--gccacaac taagtcaacg atattaattt ttgttatttt tagcttatca aaat------

-cctcttaat tttgttagtt gacgcagttg actcacacac attaccccag ----------

---------- -----a---- ---------- ---------- ---------

>362LG3

ttattatacg ttctattgtg gtacga-tgg ttttggagca cgacgaag-- ----------

--gccacaat taagtgaacg ataaca---- --------tt aagcttatca aaatctgttc

acttcttaat tttgtcaatg gacgctgtgg acccacacac ataaccccag ----------

---------- -----gagta ctaaaaggag ttgaaaagct ttgtaaa--

>300LG9

ttattatacg ttctattgtg gcacgaatgg ttttggagca cgacgaa--- -----agaga

aggccacaac ------aacg ataccaattt ttgttgtttt tagcttctta aaatctgatc

acgtcttaat tttgtcaatt gacgctgttg acccatacgc attac----- ----------

---------- ---------- ---------- ---aaaagct ttgtgaaac

>346LG3

---------- ---------- ---------- ---------- ---------- ----------

---------- ------aacg agactaattt ttgttgtttt tagcttatca aaatatgttc

gtctcttaat tttgtcaatt ggcgctgttg gcctacacac attaccccag ----------

---------- ---------- ctaaaacgag ttgaaaagct ttgtgaaac

>249LG7

ttattatacg ttctattgtg gcacgaa--- ---tgaagca caacgaagga gtgctataga

atgccacaat gaagtgaacg a-----attt ttgttgtttt tagcttatca aaacctgttt

acctgttaat tttgtcaatt gacgctgttg actcacacac attac----- ----------

---------- ---aaattta ctaaaaggag ttgaaaag-- ---------

>447bLG3

ttgttatacg ttctattgtg gcacgaatgg ttttggagca cgaataagga gtgcaataaa

atgccaccat taagtcaacg a--------- --------tt tagcttatca aaatctgtt-

atctctt-at tttgtcaatt gacgctgttg aagcaca--- ---------- ----------

---------- ---------- ---------- ---------- ---------

>210LG9

ttattatacg ---tattgtg gcacgaatgg ttttggagca ctacgaagga ----------

---------- ---------- -tgctaattt ttgttgtttt tagcttatca aaatctgtta

acctcttatt tttgtcaatt gacgctgttg actaacagac attaccccag ccagcactgc

caataactaa taaa-attta ctaaaaggtg ttgaaaagct ttgtgaaac

>477LG5

ttattataca ttctattgtg gcacgaatgg ttttggagca caacgaagga gtg-------

---------- ---------- ------attc ---ttgtttt tagcttatca aaatctgttt

acctcttaat tttggcaatt gac------- ---------- ---------- ----------

---------- ---------- ---------- ---------- ---------

>295LG3

ttattatacg ttttattgtg gcacgaatag ttttggagca cgacgaa--- ----------

---------- ------aacg atacttattt ttgttgtttt tagcttatca aaatctgttc

accccttaat tttgttaatt gacgctgttg acacacgcac attaccccag ctaatagtgc

caacaa---c taaaaagtta ctaaaaggat ttgaaaaact ttgtgaaaa

>260LG8

ttattattcg ttctattgtg gcacgaatgg ttttggagca cgacgaag-- ----------

--gccccaac taagtcaa-- ------attt ttgttgttct tagcttatca aaatctgttc

agctcttgat tttgtcaatt gaggtcgttg actcacacac attaccccag ccagtactgc

caacaactac taaaaattta ctcaaaggag ttgaaaagct ttgtcaaac

>156LG7

ttattatacg ttctattgtg gcacgaatgg ttttggagca cgtcgaagga gtgctataga

at-------- taagtcaaac ataaatattt tttttattat aagggtatca aaatctgttc

acctcttaat tttgtcaatt gacg--gttg actcacaccc attac----- ccagcactgc

cgacaactcc taaaaatttt ctaaaaggag ttgaaaagtt ttgtgaaac

>351LG2

-tattatacg tcctattgtg gcatgaattg ttttggagca cgacgaag-- ----------

--gccacaat taagtcaacg ataaatatt- --------tt tagcttatca aaatctgatc

acttcttaat tttgtgtttt aacgctgttg gctcacacac attgc----- ----------

-aacaacttc taaaaattta caagaaggag ttgaaaagct tagtgaaac

>504LG2

--attatacg ttctattgag gcacgaatgg ttttagagca cgacgaag-- ----------

--gccacaat taagtcaacg ataata---- --------tt tcgtttatca aaa-----tc

acctcttaat tttgtcaatt gacacta--- ---------- ---------- ----------

---caactcc taaaaattta ctaaaaggag ttgaaaaact ttgtgaaac

>309LG8

ttattatacg ttctattgtg gcacgaatag ttttggagca cgactaag-- ----------

---------- ---------g atactaattt ttgttgtt-- ------atga aaatctcttc

acctctaaat tttgtcaatt ga-gctgttg actcacac-- -----ctcag ccagcactgc

caacaacttc taaaaattta ttaaaaggag ctgaaaagct ttgtgaaac

>468LG10

ttattatacg ttctattgtg gcacgaatgg ttttggagca cgatgaagga gtg-------

---------- ------aacg atactaattt ttgttgtt-- ---------- ----------

----tttagt tttgtctatt g--------- ----acacac attacctcag ccagcactgc

caacaacttc taaaaattta ctaaaacgag ttgaaaagct ttgtgaaac

>83LG9

atattatacg ttctattgtg gcacgaatga ttttgtagca cgacgaagga gtgctagaga

atgctacact tgagtcaacg atagtaattt ttgttgattt tag-ttatca aaatctgttc

gcctcctaat tttgtcaact gacgctgttg actcacacac attacaccag ccagcactgc

caacaa---t taaaaattta ctaaaagtag ttgaaaagct ttgtgaaac

>116LG7

ctattatacg ttttattgtg gcacgaatga ttttggagca cgacgaagga gtgctataaa

atgtcacaat gaagtcaacg atactaattt ttgttgtt-- ---------- -----tgttc

acctcttaat tttgtcaa-t gaccctgttg gctcacacac attaccccag ccagcactgc

caacaactcc aaaaaattta ctaaaagtaa ttgaaaagct ttgtgaaac

>203LG2

ttattatacg ttttattgtg gcacgaatgg ttttggagca cgacgaagga gtgctgtaaa

atgccacaat aaagttaacg atagtatttt ttgttgtttt tagtttatca aaatctgttc

accttttaat tttg------ ---------- --tgacactc attaccccag ccagcactgt

caacaactcc taaaaattta ctaaaagtat ttgaaaagct ttgtgaaac

>344LG4

--------cg tcctattgtg gcacgaatgg ttttggagca cgacgaag-- ----------

--gccacaat tgagtcaaca ataccaattt ttgttgtt-- ---------- ----------

-ccccttgat tttgtcaatt gacggtgttg actcacacac atgatcccag ccagcactgt

ccacaactcc taaaaattta ccaaaaggag ttgaaaagct ttgtgaaat

>419LG9

tcattataca ttcta-tgtg acacaaatgg ttttggagca cgacgaag-- ----------

--ggcacaat taagtcatcg atactaattt ttgttgtg-- ---------- ----------

-tctctaaat tttgttaatt gacgctgttg acacacacac attacctcag ctagcactgc

caacaactcc taaaaattta cta------- ---------- ---------

>312LG8

atattatacg ttctgttgtg gcacgaatgg ttttggagca cgacgaag-- ----------

--gccacaac taagccaacg atacta---- --------ta tagtttatca aaatctgttc

atttcttaat tttgtcaat- --------ag actcacacac attaccccag gcagcactgc

caacaactcc taaaaattta ctaaaagaag ttgaaaagat ttgtgaaat

>502LG5

atattataca ttttattgtg gcactaatgg ttttggagca cgacgaag-- ----------

--gccacaat taagttaacg acacta---- --------tt tagcttaaca caatttgtta

atttcttaat tttg------ ---------- ---------- attatcccag tcag------

---caactcc taaaaactta ccaaaaagag ttgaaaagct ttgtgaaac

>447aLG3

-------acg ttctattgtg gcacaaatgg ttttggagca cgacgaag-- ----------

--gccacaat taagtcaacg gtactaattt ttgttgtttt tagcttatta aagtctg---

-cctcttaat tttg------ ---------- ---------- -----cccag ccagcactcc

caacaactcc taaaaattca ctaaatggag ttgaaaagtt ttgtgaaac

>297LG3

ttattataca ttctgatgtg gtacgaatgg ttttggagca cgacgaagga gtgctaaaga

ataccacaat taagtcaacg atactaattt tt-ttgtttt taggttagca aaatctattc

attttgtaat tttg------ ---------- ---------- -------aag ccatcactgc

caacaactcc taaaaattta ataaaagtag ttgaaaagct ttgtgaaac

>357LG7

ttattatacg ttcaattgtg gcacgattgg ttatggagca cgacgaatga gtgctttaga

atgccacaat taagtcgacg atacta---- ---------- ---------- taatctgttt

acctcttaat tttgtcaaat gacgctgttg actcgcacac attac----- ---acccaac

ccacaatttc taaaaagtta ctaaaaagag ttgaaaagct ttgtgaaac

>96LGX

ttattatacg ttctattgtg ctacaaatgg ttttggagca caacgaagaa gtgctacaga

atgctacaat aaagtcaacg atattaattt ttgttgtttt tagcttatca aaatctgttc

acctcttaat tttg-----t gacgctgttg actcacacaa attaccccag ccagcactgc

caagaactcc taaaaattta ctaaaaggag ttgaaaagct ttgt-gaac

>239LG9

---------- ---------- -------tgg ttttggagcg cgacgaagga gtgcttttga

atgccacaat taagtcaacg atacaaattt ttgttgtttt tagcttatct aaatctgttc

atctcttaat tttgtcaat- --------ta acttacacac attaccccgg ccagcactgc

caacaactcg taaaaattta ctaaaaggag --------at ttgtgaaac

>264LG10

---------- ---------- ---------- ---------- ---------- ----------

--gccacaat taagtcaacg atactaattt ttgttgtttt aagtttatca aaattttttc

acctcttaat tgtg-----t gacgctgttg acccacacac attaccccag tcagcactgc

caacaaatcc taaaagttta ctaaaag--- -----aagct ttgtgaaac

>387LG3

---------- ---------- ---------- ---------- ---------- ----------

--gccacaat taagtcaatg gtactaattt ttgtggttat tagcttatca aaatctgttc

acctcttaat tttgtcaat- ---------- --------ac attaccccag tcagcaatgc

caaaaactcc taaaaattta ctaaaaggag --------ct ttgtgaaac

>354LG6

---------- ---------- ---------- ---------- ---------- -tgctataga

atgctacaac taagtcgacg ataataattt ttg------- ---tttactt taatcttttc

atctcttaat tctctcaat- --------tg agtcatacac atcagcccag ccagtactgc

caccaactcc taaaaaatta ctaaaaggag ttgaaaagct ttgtgaaac

>110LG2

ttattatacg ttctattgtg gtacgaatgg ttttggagca cgacgaagga gtgctataga

atggcacaat taagtctacg atactaattt ttg-----tt tggcttatga taatctgttc

acctcttatt tttgtcaat- ----ctgttg attcacacac attaccacag ctagcactgc

caacaactcc taaaaattta ctaaaaggag ttgaaaagct ttgtaaaac

>263LG5

ttattatacg tcctactacg gcacgaatgg ttttggggca cgacgaagga gtgctataaa

ataccacaat taagtcaacg atacttattt ---ttgtttt tagcttatca agatctgttc

acctcttacg tttgtcaatt gacgctgttg actcac---- ---------- ----------

---------- ---------- ---------- ---------- ---------

>413LG3

ttattatacg ttctattgtg gcacgaatgg ttttggagca cgacgaagaa gt---ataga

atgccacaat taagtcaacg atacta---- ---------- ---catatca aaatttgttc

acttcttaat tttgtcaat- ---------- at----acg- ---------- ccagcactgc

caacaactcc taaaaattta ctaaaaaaag ttgaaaag-- ---------

>221LG3

ttattatatg ttctcttgta gcacgaatgg ttttggagca cgacgaaggg gtgttataga

atgccacaat taaaacaacg atactaattt ttgttgtttt tagcttatca aaatctgttt

acctcttatt tttgt-gatt gacccagcca ---------- -----cccag ccagcaatgc

caacaacttc taaaaattta ctaaaaggag ttcacaagct ttgtgaaac

>391LG3

---------- ---------- ---------- ---------- ---------- --------ga

atgccacaat taaatcaacg tttttaattt ttgttgtttt tagcttatga aaatctgttc

atctcttaat tttgtcggtt gacgctgttg ct-------- -------ctg ccagcactgc

caacaactcc taaa------ ---------- ---------- ---------

>238LG9

atattatacg acctattgtg acataaatga ttttagagca cgacgaagga gtgct-----

atgccacaat ctagtcaacg atactaattt ttgttgtttt tagtttgtta aaatctgtac

acctctgaat tttgtcaact gactttgtag ac------tc attaccccag ccagtactgc

caacaactcg taaaaattta ct-------- ---------- ---------

>368LG9

ttattatgca ttttattgta gcacgaatcg ttttggagca caacaaagga gtactataga

atgtcacaat taagtcaaca ctaataattt ttgttatttt taacttatca aaatctgttc

acctcttaat tttgc----- ---------- ---------- -----cacag ccagcactgc

caacaactcc taaaaattta ctaaatggag ttgaaaagct ttgtgaaac

>153LG6

---------- ---------- ---------- ---------- --------aa gtgttataga

atgccacaat taagtcaacg atacaaattt ttgttgtttt tagcttatca aaatctgttc

acattttaat tttgtcaatt gacgctgttg attaacacac actaccccag ccagcactac

caacaactcc taaaaattta ccaaaaggag ttgaaaagct ttgtgatat

>286LG10

---------- ---------- ---------- ---------- ---------- ----------

---------- taagtcaacg atattagttt ---ttgtttt tcgcttatca caatcttttc

attaattaat tttgtcaatt gacgctgtcg actcacacag attaccccag ccagcactgc

caacaactcc taaaaattta ctaaaagggg ttgaaaagct ttgtgaaac

>192LG5

---------- ---------- ---------- ---------- ---------- -----ataaa

atgccacaat taagtctacg atactaattt ttgttgtttt tagcttatca aac-------

-tctattaat tgtgttaatt gacgctgttg actcactcac attaccccag tcagcact-c

caagaactcc taaaaattta ctaaagagag ttgaaaa-tt ttgtgtaac

>376LG9

---------- ---------- ---------- ---------- ---------- -----ataga

atgccacaat taagtcaacg atactaattt ttgttgtttt tag------- ----------

---------- ----taaatt gacgttgttg acctacacac attatgccag ccagcactgc

caacaactcc taaaaattta ctaaaaggag ttaaaaa-at ttgtgaaac

>301LG3

---------- ---------- ---------- ---------- ---------- --------ga

aggccacaac taagtcgacg ata---attt ttgttgtttt tagtttatca aaatttgttt

atttataaat tttg-----t gacgctgttg atacacacac gtaatcccag ccaacactgc

caacaactcc taaaaattta ctaaaagtag ttgaaaagct ttct-----

>333LG3

---------- ---------- ---------- ---------- ---------- ---------a

aggccataat taagttaacg ataataattt ttgttgtatt tagcttatca aaatctgttc

gt-------- ----tcaaat gacgctgttg actcacacac attaccccag ccagcactcc

caacaactcc -aaaaattta ctaacagtag ttgaaaag-- ---------

>228LG7

ttattattcg ttctattgtg atacgaatgg ttttggagca cgacgagg-- ----------

--gccgcaat taagtctgcg atactaattt tcgttgtttt tagcttttca aaatttgttt

a-tttttaat ttcgtcaatt gacgctgttg actcatacac attactccag ccagcactac

caacaactcc taaaaattta ccaaaaagag ttgaaaag-- ---------

>389LG3

---------- ---------- ---------- ---------- ---------- ----------

--gtcacaat taagtctacg atcatagttg ttgttgtttt tagcttatca aaatctgttc

atttttttat tttgtcaatt gacgctattg gctcacatac attac----- ----------

---------- ---------- ---------- ---------- ---------

>256LG4

attttatacg ttctattata -------cta ttatatagac tgatccagaa atactataga

atgccacaat taagtcaagg atactatttt ttgttgtttt tagcttataa aaaatctgtt

tcctcttaat tttgccaatt gacgctgttg actcacacac attaccccag ctagcaatgc

caagaactac taaaaatttc ctaaaaggag tagaaaaa-c ttgtaaaaa

>365LG3

---------- ---------- ---------- ---------- ---------- ----------

--gccacaat taagtcaacg atgctaattt ttgttatttt gagcttatca aaatttgttc

agctcttaat cttatcaatt gtcgttgttg ac-------- atgacctcag ccagtactgc

caagaattcc taaaaattta ctaaaaggag ttaaaaag-- ---------

>214LG3

atattatact ttctatt--- ----gaatgg ttttggagca tgacgaagga gtgctataga

ctgccacaat aagtttaaaa atactaattt ttgttgtttt tagcttatca aaatctgttc

aacttttaat tttgtcaatt gacgctgttg actcacttac attaccccag ----------

-------tcc taaaaattta ccaaaaggag ttgaaaagtt ttgtgaaac

>250LG7

ttattatacg ttctattgtg gcacgaa--- ---------- -------gaa gtgctataga

atgccacagt taagttaacg aaactaattt ttgttgtttt ttgcttatca aaatctg---

-cctcctaat tttgtcaatt gacactgttg acttacaaac attaccctcg ccagccctg-

---------c caaaaattta ctaaatggag ttgaaaagct ttgagaaac

>292LG3

---------- ---------- -------tgg ttttggagca cgactaagga gtgctataga

atgccacaat taagtcaacg atactaattt ttgttgcttt tagcttatca aaatct----

-cctcttaat tttgcaaaat gacgctgttg actcacacac agtac----- ----gactgc

caacaacttc taaagtttta ctaaaaggag ttgaaaagct ttgt-----

>267LG10

tttttatacg ttttattgtg gtaccaatga atttggagca cgacgaaaga gtgctatgga

acgccaca-- ------aacg gtactaattt ttgttgtttt tagcttatca aaatctgttc

a--------- ----tcaatt gtcactgttg actcatacac attagcccag ---atactgc

caacaacttc taaaaatttg ctaaaaagag tttaaaaact ttgtgtaac

>237LG9

---------- ttctattgtg gcacgaatga ttttggagca cgacaacaga -----gtaga

atgtcagaat taagtcaacg atacaatcta tttttgtaat taactaagca aagtcaataa

accttttaat cttgtaaatt gacgctgttg actctcacac attacctgag ccagcactgc

caacaactt- caaaaattta ctaaaaagag ttgaaaagct ttgtgaaac

>406LG3

---------- ttctattgtg gcacgaatgg ttttggagca caacgaag-- ----------

--gccacaat taagtcaacg a--------- ---------- ---------- ----------

--------at tttg-----t gacactgttg actttcacac attaccccag ccaccactac

caacaac--a taaaaattta ctaaaaggag ttgaaaagca ttgtgaaac

>280LG5

ttattatacg ttcta----- ---------- ---------- ---------- ----------

---------- ------aacg atactaattt ttgttgtt-- ---catatca aaatctgttc

tcatcttcat tttgtgaatt aacgctgttg atccacacac attaccccag ccagcactgc

caataactcc caaaaatgta ttaaaaggag ttgaaaagcc atgtgaaac

>355LG3

---------- ---------- ---------- ---------- ---------- ----------

---------- ---------- ---------- ---------- ---------- ------gttc

acttcttaat tttgtt--tt gacgctgttg actgacacat attaccctag ccagcactac

caacaactcc taaaaattta ctaaaaaaag ttgaaaatct ttgggaaac

>268LG10

---------- ---------- ---------- ---------- ---------- ---------a

aggccacaat taagtcaacg atactaattt ttgttgtttt tagaaaat-- atgtatattc

accacttaat tttgtcaatt gacgctgttg actcacacac attat----- --gtcactgc

caacaactc- caaaaattta ctaaaaggaa ttgaa---tt ttgtgaaat

>321LG7

ttattatacg ttctattgtg gcacgaatcg ttttggagca cgaccaagga gtgctatag-

---------- ------aacg ataccaattt ttgttgtttt tagcttatca aaatctgttc

acctcttaat tttatcaatt gacgctgttg actcacacac attaccccag caagcactgc

caacaactcc taaaag---- ---------- --------ct ttgtgaaac

>469LG3

---------- ----gttgtg gcacgaatgg ttttggagca cgacgaag-- ----------

--gcctcaat taagtcaaca atacaatttt ttgttgtttt tagcttatca aa--------

-cctcttaat tttgtcaatt gacgctgttg attcgcacac attac----- ----------

---------- ---------- ---------- ---------- ---------

>194LG7

---------- ---------- ---------- ---------- ---------- ----------

---------- ---gtcaacg atactaattt ttgttgtttt tagcttatca aaatctgtt-

acttcttaat tttgtcaatt gaccctgttg actctgagat ---accccag tcagcactgc

caacaactca caaaaattta ctaaaagtag ttgaaaagct ttgagaaac

>323LG2

---------- ---------- ---------- ---------- ---------- ----------

---------- ---------- ---------- -----ggttt tggcttatta aaatctgtta

ccctcttcat tttgtcaatt gacggtgttg actctcacag --tgacctag cccgtactgc

caacaacatc taaaaattga ttaaaagtag ttgaaaagct ttgtgaaac

>313LG4

---------- ttctattgtg ---------- -------gca ctccttcgcc gtgctaaacc

atgccacaat taagtcaacg atactaatct tggttttgtt aaacttgtca aactctgttc

acctcttaat tttgtcaatt gaccctgtta actcacacat attaccccag ccagcactgc

gaacaattta taaaaattta c--------- ---aaaagct ttgtgaagc

>443LG6

---------- ---------- ---------- ---------- ---------- ----------

---------- ---------- ---------- --------tt tagcttagca aaatctgttc

accttttaat tttgtcaac- ---gctgttg actaacatac attacctcag ccagcacatc

caacaacttt taaaaattta ct-------- ---aaaagct ttgtgaaac

>360LG3

---------- ---------- ---------- ---------- --acgaatga gtgctataga

atgccataat taagtcttcg acactaattt ---ttgtttt tagcttatca aaatttgttc

acctcttaat tttgtcaac- ---gctgttg actcacacac attacctcag tcaccactgc

caacaactcc taaaaattta ctaa------ ---------- ---------

>41bLG9

---------- ---------- ---------- ---------- ---------- ----------

---------- ---------- ---------- --------tt tagcttatca aaatttgttt

cccctttaat tttgtcaatt ggtgctgttg actcacacac attactc--- ----------

---caacttc taaaaattta ctaaaagcag ttgaaaattt ttgtgaatg

>204LG9

-tattatacc ttctagtatg gcttgaatgg atttggagca cgacgaagga gtgttataga

atgccacaat taagtcaacg atactaattt ttgttgtttt taacttatca aagtcagttt

acctcttaac tttgtcaatt aacgccggtg actc--acac attattccag -------tac

caatgagttg cacaaa---- ---------- ---aaaagct ttgtgaaac

>399aLG9

---------- ttctattgtg gcacgaatgg ttttggagca cgacgaagga gtactacaga

atgctataaa taagtcaacg agattt---- ------tttt tagcttatca aaatttgttc

atttgttaat tttg-----t gacgctgttg actcacacac attaccccag ----------

---------- ---------- ---------- ---------- ---------

>399bLG9

---------- ttctattgtg gcacgaatgg ttttggagca cgacgaagga gtgctataga

atgctac--- ------aacg atacta---- --------tt cagcttatca aaa--tgttc

acctcttaat tttg-----t gacgctgttg actcacacac attaccccag ----------

---------- ---------- ---------- ---------- ---------

>399cLG9

---------- ttctattgtg gcacgaatgg ttttggagca cgacgaagga gtgctataga

atgctac--- ------aacg atacta---- --------tt tagcttatta aaa--tgttc

acctcttaat tttg-----t gacgctgttg actcacacac attaccccag ----------

---------- ---------- ---------- ---------- ---------

>207LG2

ttattatacc ttctattgtg gcacgaatgg ttgtggagca caacgaagga gtgctataga

atgcctcaat taagtcaaca atacta---- --------tt tagctcatca aaatatgttc

acctcctaat tctgtcaatt gac----ttg aatcaca-ac attaccctaa ccagcactgc

caacaactcc taaaaattta ct-aaaggag ttgaaaagct ttgtgaa--

>348LG3

---------- ttcaattgtg gcacgaatag ttttggagca cgtcgaagga gtgctataga

ataccacaat taagtcaacc ata---attt ttgtattttt tagcttatga atatttgttt

acctcttaat tttgtcaatt gacgctgat- ---------- -----cccag ccagcactac

caacaaaccc taaaaattta ctaaaaagag ttgaaaagat ttgtgaaa-

>410LG6

ttattgttcg ttctagtatt acacaaatgg ttttggagca cgacgaag-- ----------

--gccacaat taagtcaacg atactaa--- --------tc tagcttatca aaaacagttc

acctcttaat tttgtcaatt gacactgttg acttgcagac -----aacag ccagcactgc

caagaactcc gaaaaa---- ---------- ---------- ----g----

>328LG9

ttattatacg ttttattgtg gcacgaatca ttttggagca cgactaagga gtgctataag

gtgc------ ------aaag ataaaaattt ttgatgtttt tagattagca aaatatgttc

acctcctaat tttgtgaat- ---------- --aaacacac attaccccag ccagcactgc

caacaactcc taaaaattta ctaagaggag ttgaaaagct tt-------

>371LG3

---------- ---------- ---------- ---------- ---------- ----------

--gccacaat taagaaaacg atactaattt ---ttgtttt cagcttgtca aaa-------

-cctcttaat tttgtcaatt aacgc----- ---------- attaccccag ccag------

---caactcc taacaattta ctaaaaggag ttgaaaagct ttgtgaaac

>439LG7

ttattatatg ttttatcgtg gcgcgaatgg ttttggagca cgacgaagga gtg-------

---------- ------acta ttactaattt ttgttgctct tg-cttttca aaatctgttc

acctcttaat tttgtcaatt gacgctgttg ---------- attacgccag ccagcactgc

catcaactct taaaaa---- ---------- ---------- ---------

>457LG3

ttattatacg gtctactgtg gtacgaatgg ttttggagca cgacgaag-- ----------

--gccacaat taagacaacg atacaa---- ---------- ---------- ----------

-tctcttaat tttgttaatt gacgct-ttg actcacacac attaacccag ccagcactgc

caaaaactct aaaaacttta ctaaaaggaa ttgaaaagct ttgtgaaa-

>456LG3

--attatacg ttctattgtg gcacgaatgg ttttggagca cgacgaag-- ----------

--gccgcaac taagccaacg atactaattt ttgaaa--aa taagtcaata attttaaaag

atttcttaat tttgtcaatt gacgcggttg attcacacat attaccctag ccagcactgt

caacaa---c taaaaattta ctaaaaggag ttcaaaagct ttgtgattt

>381LG4

---------- ---------- ---------- ---------- ---------- ---------a

aggccacaat taactcaaag atactaattt ttgttg--tt tagattatca aaatctgttc

acct------ --------tt gacgctgctg actcacaca- -----cccag ccagcactgc

ccacaactcc taaaaatttc ctaaaaaaag ttgaaaag-- ---------

>261LG8

ttattataca ttctattgtg attcgaatgg ttttggagca cgacgaagga gtgctataga

atgccacaat taagtgattg ttactaattt tt-tcgtttt aagcttatta aaatctgttc

acctcttaat tttgtgaatt gacgctgttg actcacatac attac----- ----------

---------- ---------- ---------- ---------- ---------

>88aLG2

ttattataac ttctattgtg gcacgaatgg ttttggagca cgactaagga gtacaataga

atgcctcaat taagttatcg atactaattt ttgttgtttt taccttatca aa--------

--------at tttgtctatc gacgatgttg actctcacac attaccccag ccagcactgt

caacaactcc taaaaattca ctaaaagcag ttgaaaag-- ---------

>385LG6

aaattttaag ttctattgtg gcacgaatgg tttttgagca cgacgaagga gtgctataaa

attctacaat taagtcaacg atactaattt ttgttgtttt tag------- ----------

---------- ----tcaatt gacgctgttg actcagacac ataaccccag cacccactgc

caacaactcc taaaaattta ctaattaaag ttgcaaag-- ---------

>482LG3

cga------- ttctattgtg gcaagaatgg ttttggagca cgacgaagga gtgc------

----ctcaat taagtcaacg agactaattt ttggtgcttt tagcttatca aaatct----

---------g tttg-----t gacgctgttg actcacacac attaccctag ccagcactgc

caacaacttc ta-tttttta ctaaa----- ---------- ---------

>454LG7

---------- ttctattgcg gcgcgaatgg ttttggagca cgacgaagga gtgtcataaa

atgacaaaat aaattcaacg atactaattt ttgatg---- ---------- ----------

-cctcttaat tgtgtcaatt gatgcttttg ac-------- ---------- actgcactgc

caacaactcc taaaaatttt ctaaaaggag ttgaaaagct tgtggaa--

>398LG10

---------- ---------- ---------- ---------- ---------- ----------

---------- --tgtcaacg atactagttt ttgtcgtttt tagcttatca aaatttgttt

acatattaat tttgtcagtt gacgctgttg actcattttt ttcgatacaa ---aacttat

caataacttc taaaaattta ctaaaaggag ttgaaaacct ttttgaaac

**D Alignment of repeats belonging to dispersed TCAST6 elements**

>0024_LG10

aaccttatcg cgcacccaag ataatggcga cttttgatga aataaaaatc ctgaaatttt

actcaccagc ctgtacattg ataaggacca ttctcgattt ttattttttt gttaataaat

gaagtagcag ttttgtgagt ttttgggcat taactgttaa tttgaaagtt tttagaccaa

atgaatcctt acctgctagc ccatcgaacg ctggtatcag aattataaat tacttggttg

gtgaaataat cgttttttat ttagacgcaa actagacgcg ttatttatta gaagatgata

taatttgcca acaaagggat cagttaccag tagccgacct ttttgaagcc ccttatttac

agtcttgcaa taaaactgat gcctgcattc aaatatccta tcaaaatctt ttacaatctc

agttcacatt gtca------ ---------c cacagccact c--aaataat ttttaaagtt

aaaatgtaaa agtgcgttta attcaaaatc tcattggtgt ttttcttcgc taatttcgta

aacaattatc tgaagtgaat acgcgtggtt tactataaaa tttagaattt gactctttgg

tcgaaaatta ctttttttaa attattattt ttatctgcaa gtgaaaaatt accaaaacat

taggggagat acctttcgaa cgtacagtca ggcgtgtaaa atgcaat-tt tttttattta

attagaaatc gcgattttct tagatgcgcg ataaa

>0022_LG8

aaccttatcg cgcacccaag aaaatcgtga cttctaatga aaaaaaaatt ttgaaatttt

acacaccagc ctgtacattg ataaggacca gtctcgattt ttaatttttt gtaaataaat

aacgtagcag ttttatgagt ttttgggcac taactgttga tttaaaagct tttagactaa

acgaatcctt acctgctagc ccatcgaacg ttagtatcga aattataaat tacttggttg

gtgaaataat cgttttttat ttagatgcaa accagacgcg tgatttatta gaagatggta

taatttgcca acaaagggat cagttaccag tagccgacct ttttgaagcc ccttatttac

aattttgcaa taaaactgat gcctgcattc aaatatccca acgaaatctt ttacaatctc

agttcacatt gtcacctaaa tttacgacgc cacagcaaca catatataat ttttaaagtt

aaaatgtaaa agtgctttta attcaaaacc tgattggtat ttttcttcgc taatttcgta

aacaattatc tgaagtgaaa acgcgtggtt tagtataaaa ttgagaattt ggttttttgg

tcgaaaat-- --ttttttta actattattt ttatgtgcaa gtgaaaaatt accaaaaaat

taagggagat acctttcgaa cgtacagtca ggttcgtaaa atgcaatatt tttttattta

attagaaatc gcaattttct taggtgcgcg atagg

>0031_LG8

---------- ---------- ---------- --cctaatga aatgaaaatc ctgaaatttt

acacaccagc ctgtacattg ataaggacca ttctcgattt ttttttttct gttaataaat

aaagtagcag ttttgtgagt ttttgggcac taactgttaa tttaaaagtt tttagaccaa

atgaatcctt acctgctagc ccatcaaacg ctggtatcag aattataaat tacttggttg

gtgaaataat cgtttttaat ttagacgcaa actatacgcg tgatttatta ggggatggta

taatttgcca acaaagggat cagttaccag tagccgacct ttttgaagcc ccttatttac

aattttgcaa taaaactgat gcctgcattg aaatatccca acgaaatctt ttacaatctc

agttcacatt gtcacctaaa tttacgacgc cacagcaact cataaataat ttttaaagtt

aaaatgtaaa agtgcgttt- attcaaaatc tgtttggtat tttt-ttcgc taattccgta

aacaattatc tgaaatgaat acgcgtggtt tagtataaaa tttagaattt gattttttgg

tcgaaaatta ctttttttta attgtcattt ttatctgcaa gtgaaaaatt accaaaaaat

taggggagat accattcgaa cgtacagtca ggcgtgtaaa atgcaatatt ----------

---------- ---------- ---------- -----

>0085_LG8

---------- --cacccaag aaaatcgcga cttctaatga aataaaaatc ctgaaatttt

acacaccagt ctgtacattg ataaagacca ttctagattt ttattttttt gttaataaat

aaagtagcag ttttgtgagt tttttggcac ttactgttaa tttaaaagtt tttagaccaa

atgaatcctt acctgctagc ccatcgaacg ctggtatcag aattataaat tacttggttg

gtgaaataat cgttttttat ttagaggcaa accagacgcg tgatttatta gaagatggta

taatttgcca ---------- -----accag taaccgacct ttttgaagcc tcttatttac

agtgttgcaa taaaactgat gcttgcatta aaatatccca acgaaatctt ttacaatctc

tgttcacatt gtcaccaaaa tttacgacgc cacagcaact cataaataat ttttaaagtt

aaaatgtaaa agtgcgtgta ag-------- ---------- ---------- ----------

---------- ---------- ---------- ---------- ---------- ----------

---------- ---------- ---------- ---------- ---------- ----------

---------- ---------- ---------- ---------- ---------- ----------

---------- ---------- ---------- -----

>0015_LG3

---------- cgcacccaag aaaatcgcca cttttaatga aataaaaatc ctgaaatttt

acacaccagc ctgtacattg ataaggacca ttctcgattt ttattttttt gttaataaat

aaagtagtaa ttttgtgagt ttttgggcac taactgttaa tttaaaaatt tttagaccaa

atgaatcctt acctgctagc ccatcaaacc ctggtatgag aattataaat tacatggttg

gtgaaataat cgttttttat ttagagacaa accagacgcg tgatttatta gaagatgata

taatttgcca acaaagggat cagttaccag tagccgacct ttttgaagtc ccttatgtac

agtcttgcaa taaaactgat acttgcattg aaatatccca acgaaatctt ttacaatctc

agttcacatt gtcacctaaa tttatgacgc cacagcaact cataaataat ttttaaagtt

aaaatgtaaa aatgcgttta attcaaaatc tgtttggtat ttttcttcgc taattttgta

aacaagtatc tgaagtgaat acgcgtggtt tagtataaaa tttagaattt gactttttgg

tcgaaaatta cttttttt-- -taattattt tcatctgcaa gtgaaaaatt accaaaaaaa

taggggagat acctttcgaa cgtacagtca ggcgtgtaac atgcaatatt tttttattaa

attagaaatc gcgattttct taggtgcgcg ataag

>0080_LG8

---------- ---------- ---------- ---------- ---------- ----------

---------- ---------- ---------- ---------- ---------- ----------

---------- ---------- ---------- ---------- ---------- ----------

---------- ---------- ---------- ---------- ---------- ----------

---------- ---------- ---------- ---------- tgatttatta gaagatggta

taatttgcca acaaagggat cagttaccag tagccgacct ctctgaagcc ccttatttac

agtctcgcaa taaaactgat gcctgcattg aaatatccca acgaaatctt ttacaatctc

agttcacatt gtcacctaaa tttacgacgc cacagcaact cataaataat ttttaaagtt

aaaatgtaaa agtgcgttta attcaaaatc tgattggtat ttttcttcgc taatttcgta

aacaattatc tgaagtaaat acgcgtggtt tagtgtaaaa tttaaaattt gattttttgg

tcgaaaatta ctttattt-a attatcattt ttatctgcaa gtaaaaaatt accaaaaaat

tacaggagat accattcgaa cgtacaacca gacgtggaaa atgcaatatt tttttattta

atttgaaatc gcgattttct taggtgcgcg ataag

>0007_LG8

aaccttatcg cgcatccaag aaaatc-caa aaaataatga aataaaaatc ctgaaatttt

acacaccagc ctatacattg ataaggacca ttctcgattt ttattttttt gttaataaat

aaagtagcag ttttctgagt ttttgggcac taactgttaa tttaaaagtt tatagaccaa

ctgaatcctt tcctgctagc ccatcgaacg ctggtatcag aattataaat tacttggttg

gtgaaataat cgttttttat ttagacgcaa actaggcgcg cgatttatta gaagatagta

taatttgcca acaaagggat cagttaccac tagccgacct ttttgaagcc ccttatttac

agtcttgcaa taaaactgat gcctacattg aaatatctca acgaaatctt ttacagtctc

agttcacatt gtcacctaaa tttacgacgc cacagcaact cataaatact ttttaaagtt

aaaatgtaaa agtgcgttta attcaaaatc tgattggtat ttttcttcgc taatttcgta

aacaattatc tgaagtgaat acgcgtggtt tagtataaaa tttagaattt gattttttag

tcaaaaatta cttttttt-a attattattt ttatctgcaa gtaaacaatt atcaaaaaat

taggggagat acctttcgaa cgtacagtca ggcgtgtaaa atgcaatatt tttttattta

attagaaatt gcgattttct taggtgcgcg ataag

>0004_LG3

aaccttatcg cgtacccaag aaaatcgcga cttctaatga cataaaaatc ctgaaatttt

acacaccagc ctgtacacag ataaggacca ttctcgattt ttattttttt cttaatgaat

aaagtagcag ttttgtgagt ttttgggcac taactgttaa tttaaaagtt tttagaccaa

atgaatcctt atctgctagc ccatcgaacg ctggtatcag aattataaat tacttggtta

gtgaaataat cgttttttat ttagacgcaa actagacgcg tgatttatta gaatatggta

ttatttgcca acaaagggat cagttaccag tagccgacct ctctgaagct ccttatttac

agtcttgcaa taaaactgat gcctgcattg aaaaatctca acaaaatctt ttacaatctc

agttcacatt gtcacctaat tttacaacgc cacagcaact tataaataat ttttaaagtt

aaaatgaaaa agtgcgttta attcaaaatc tgattagtat atttcttcgc taatttcgaa

aacaattatt tgaagtgaat acgcgtggtt taatataaaa tttaaaattt gattttttgg

tcgaaaatta cttttttt-a attattattt ttatctgcaa gtgaaaaatc accaaaaaat

taggggagat acctttcgaa cgtacagtca ggcgtgtaaa atgcaatatt tttttattta

attagaaatc gcgattttct taggtgcgcg ataag

>0050_LG8

---------- -acacccaag aaaatcgcga cttctaatga aataaaaatc ctgaaatttt

acacaccagc ctatacattg aaaaggacca ttctcgattt ttattttttt gttaataaat

aaagtaacag ttttgtgagt ttttgggcac taactgttaa tttaaaagtt tttagaccaa

a--------- ---tgctagc ctatcgaatg ctagtatcag aattataaat tacttggttg

gtgaaataat cgttttttat ttagacgcaa actagacgcg tgatttatta gaagatggta

taatttgcca acaaagggat cagttaccag tagccgacct ttttgaagcc ccttatttac

agtcttgcaa taaaactaat gcctgcattg aaatattcca atgaaatctt ttacaatctc

agttcacatt gtcacctaaa tttacaacgc cacagcaact cataaataat ttttaaagtt

aaaatgtaca agtacgttta attcaaaatc tgattggtat ttttcttcgc taacttcgta

aacaattatc tgaagtgaat acgcgtggtt ttgtataaaa tttagaattt gactttttgg

tcgaaaatta cttttttt-a attattattt ttatctgcaa gtgaaaaatt accaaaaaat

taggggagat acctttcga- ---------- ---------- ---------- ----------

---------- ---------- ---------- -----

>0003_LG3

atccttatcg cgcacccaaa aaaatcacga cttctaatga aataaaaatc ctaaaatttt

acacaccagc ctgtacattg ataaggacca ttctcgattt ttgttttttt gttaataaat

aaagtagcag ttttgtgagt ttttgggcac taactgttaa tttaaaagtt tttagaccaa

ataaatcctt agctgctagc ccatcgaacg ctggtatcag aattataaat tacttggttg

gtgaaataat cgttttttat ttagacgcaa actagacgcg taatttatta gaagatggta

taattcgcca acaaagggat cagttaccag taaccgacct ttttgaagcc ccttatttac

agtcttgcaa taaaattgat gcctacattc aaatatccca acgaaatctt ttacaatctc

agttcacatt gtcacctaaa tttacgacgc cacagcaact catacataat ttttaaagtt

aaaatgtaaa agtgccttta attcaaaatc tgattggtac ttttcttcgc taatttttta

aacaattatc tgaagtgaat acgcgtggtt tagtataaaa tttagaattt gattttttgg

tcgaaaatta cttttttt-t attattattt ttatctacaa gtaaaaaatt accaaaaaat

taggagagat acctttcgaa cgtacaatca ggcgtgtaaa atgcaatatt tttttattta

attagaaatc gcgtttttct taggtgcgcg ataag

>0012_LG2

aaccttatcg cacacccaag aaaatcgcga cttctaatga aataaaaatc ctgaaatttt

acacaccacc ctgtacactg ataaggacca ttctcgattt ttattttttt gttaataaat

aaagtagcag ttttgtgagt atttgggcac taactgttaa tttaaaagtt tttagaccaa

atgaatcctt acctgctagc ccatcaaacg ctggtatcag aattaaaaat tacttggttg

gttaaataat cgttttttat ttagacgcaa actagacgcg tgatttatta gaagatggta

gaatttgcca acaaacggat cagttaccag tagctgaact ttttgaagct ccttatttac

agtcttgcaa taaaactgat ggctacattg aaatatccca acgaaatctt ttacaatctc

agttcacatt gtcacctaaa tttacgacgc cacaggaact cataaataat ttttaaagtt

aaaatgtaaa agtgcgttta attccaaatc tgactggtat ttttcttcgc taattttgta

aacaattatc tgaagtgaat acgcgtgctt tagtataaca tttagaattt gactttttgg

tcaaaaatta catttttt-a attattattt ttatctgcaa gtgaaaaatt accaaaaaat

taggggagat acctttc--- --tcaggtca ggtgtgtaaa gtgcaatatt tttttattta

attagatatc gcgattttct taggtgcgcg ataag

>0001_LG2

aaccttatcg cgcacccaag aaaatcgcga cttctaatga aataaaaatc ctgaaatttt

acacaccagc ctgtacattg ataaggacca ttctcgcttt ttattttttt gttaataaat

aaagtagcag ttttgtgagt ttttgggcac taactgttaa tttaaaagtt tttagaccaa

atgagtcctt acctgctagc ccatcgaacg ctggtatcag aattattaat tacttggttg

gtgaaataat cgttttttat ttagatgcaa actagacgcg tgatttatta gaagctggta

taatttgcca acaaagggat cagttaccag tagccgaact ttttaaagct ccttatttac

agtcttacaa taaaactgat gcctgcattg aaatatccca acgaaatctt ttacaatctc

agttcacatt gtcacctaaa tttacgacgc cacagccact cataaataat ttttaaagtt

aaaatgtaaa tgtgcgttta attcaaaatc tgattggtat ttttcttcgc taatttcata

aacaattatc tgaagtgaat acgcgtggtt tagtataaaa tttagaattt gactttttgg

tcgaaaatta ttttttttaa attattattt ttatctgcaa gtgaaaaatt accaaaaaat

taggggagat acctttcgaa cgtacaatca gtcgtgtaaa atgcaatatt tttttattta

attagaaatc gcaattttct ta-------- -----

>0009_b_LG2

---------- -acacccaag aaaatcgcgg cttctaataa aataaaagtc ctgaaatttt

acacaccagt ctgtacattg ataaaaaccg ttttcgattt ttattttttt gttaataaat

aaagtagcaa ttttgtgagt ttttgagcat taactgttaa tttaaaagtt attagaccaa

ataaatcctt acctgttagc ccatcgaagg ctggtatcag aattataaat tacttggtta

gtaaaataat cgttttttat ttagacgcaa accagacgcg tgatttatta gaagatggta

taatttgcca acaaaaggat cagttaccag tagccgacct ttttgaagcc ccttatttac

agtcttgcaa caaaactgat gcctgcattg aaatatccca acgaaatctt ttacaatctc

agttcacatt gtcacctaaa tttacgacgt cacagcaact cataaataat ttttaaagtt

aaaatgtaaa agtgcgttta attcaaaatc tgattggtat ttttcttcgc taatttcgta

aacaatcatc tgaagtgaat acgcgtggtg tagtataaaa tttagaattt gactttttgg

tcaaaaatta ctttttttcg attattattt ttatctgcaa gtgaaaaatt accaaaaaat

tagaggagat accttttgaa cgtacagtca ggcgtgtaaa atgcaatatt tttttattta

attagaaatc gcgattttct taggtgcgcg ataag

>0002_LG2

aaccttatcg cgcacccaag aaaatcgcga cttctaacga aataaaaatc ctgaaatttt

acacaccagc ctgtacatta ataaggacca ttctcgattt ttattttttt gttaataaat

aaagtagcag ttttgtgtgt ttttgggcac taactgttaa tttaaaagtt tttagaccaa

atgaatcctt acctgctagc ctattgaatg ctggtatcag aattataaat tacttaattg

gtgaaataat cgttttttat ttagacgcaa actacacgcg tgatttatta gaagatggta

taatttgcca acaaagggat cagttaccag tagccgac-- ttttgaagcc ccttatttac

agtcttgcaa taaaactgat gcctgcattt aaatatccca acaaaatctt ttacaatctc

agttcacatt gtcacctaaa tttacgacgc tacagcaact cataaataat tttttaagct

aaaatgtaaa agtgcgttta attcaaaatc tgattggtat ttttcttcgc taatttcgta

aacaattatc tggagtgaat acgcgtggtt tagtataaaa tatagaattt gactttttgg

tcgaaaatta ctttttttaa attattattt ttatctgtaa gtgaaaaatt accaaaaaat

taagggagat acctttcgaa catacagtca ggcgtgtaaa atgtaatatt tttttattta

attagaaatc gcgattttct taggtgcgcg ataag

>0043_LG2

---------- ---------- ---------- ---------- aataacaatc ctgaaatttt

acacacgacc ctgtacattg ataaggacca ttctcgattt ttatttttt- -gttataaat

aaagttgcag ttttgtgagc ttttgggcac taactgttaa tttaaaagtt tttagaccaa

attaatcctt acctgctagc ccatcgaacg ctggtatcag aattataaat tacttagttg

gtgaaataat cgttttttat ttagacgcaa actagacgcg tgatttatta gaagatggta

taatttgcca acaatgggat cagttaccag tagccgatct ttttgaagcc ccttatttac

agtcttgcaa ttaaactgat gcctgcattg aaatatccca acgaaatctt ttacaatctc

agttcacatt atcacctaaa tttacgacgc cacagcaact cataaataat ttttaaagtt

aaaatgtaaa agtgcgttta attcaaaatc tgattggtat ttttcttcgc taatttcgta

aacaattatc tgaagtgaat acgcgtggtt tagtataaaa tttagaattt gattttttgg

tcgaaaatta ctttttatca attattattt ttatttgcaa gttcaaaatt accaaaaaat

taggggagat acctttcgaa caaacagtca ggcgtgtaaa atgcaatatt tttttattt-

------catc gcgattttct taggtgcgcg ataag

>0093_LG5

---------- ---------- ---------- ---------- ---------- ----------

---------- ---------- ---------- ---------- ---------- ----------

---------- ---------- ---------- ---------- ---------- ----------

---------- --ttgctagc ccatcaaacg ctggtatcag aattataaat tacttggttg

gtgaaataat cgttttttat ttagacgcaa acaagacgcg tgatttatta gatgatggta

taatttgcca agaaagagat cagttaccag taaccgacct ttttgaagct ccttatttac

actcttgcaa taaaactgat gcttgcattg aaatatccca acgaaatctt ttataatctc

agttcacgtt gtcacctaaa tttacgaaaa cacagcaact cataaataat ttttaaagtt

aaaatgtaaa agtgcgttta attcaaaatc tgattggtat ttttcttcgc taatttcgca

aacaattatc cgaaatgaat acgcgtggtt tagtatagaa tttggaattt aaatttttgg

tcgaaaatta ctttttttta att------- ---------- ---------- ----------

---------- ---------- ---------- ---------- ---------- ----------

---------- ---------- ---------- -----

>0021_LG5

----ttatcg cgcacccaag aaaatcgcga cttctaataa aataaaaatc gtgaaatttt

acacaccagc ctgtacattg ataaggacca ttctcgattt ttattttttt gttaataaat

aaagtagcag ttttgtgagt ttttgggcac taatggttaa tttaaaagtt tttggaccaa

atgaatcctt acctgctagc ccatcgaacg ctggtatcag aattataaat tacttggttg

gtgaaataat cgttttttat ttagacgaaa accagacgcg tgatttatta gaagatggtc

aaatttgcca acaaagggat cagttaccag tagccgacct ttttgaagcc ccttatttac

agtcttgcaa taaaactgat gcctgcattg caatatccca acgaaatctt ttacaatctc

agttcaaatt gtgacctaaa tttaccgcgc cacagcaact cataaataat ttttaaagtt

aaaatgtaaa agtgcgttta attcaaaatc tgattggtat ttttcttcgc taatttcgta

aacaattatc cgaagtgaat acgcgtggtt tagtataaaa tttagaattt gattttctgg

tcgaaaattc cttttttt-a attattattt ttatctgcaa gtgaaaaatt accaaaaaat

taggggagat acctttcgaa cgtaca---- -----gtaaa atgcaata-t tttttattta

ataagaaatc gcgaatttct taggtgcgcg atat-

>0008_LG9

aaccttatcg cgcacccaag aaaatcgcga cttctaatga aataaaaatc ctaaaatttt

acactccagc ctgtacattg ataaggacca ttctcgattt ttattttttt gttaataaat

aaagtagcag ttttgtgagt ttttgggcac taactgttaa tttaaaagtt tttagaccaa

gtgaatcctt acctgctagc ccatcgaacg ctgatatcag aattataaat tacttggttg

gtgaaataat cgttttttat ttagacgcaa accagacgct tgatttatta gaagatgtca

taatttgcca acaaagggat cagttaccag tagccgacct ttttgaagct ccttatttac

agtcttgcaa taaaactgat gactgcattg aaatatccca acgaaatctt tcacaatctc

agt------- -tcacctaaa tttacaacgc cacagcaact cataaataat ttttaaagtt

aaaatgtaaa agtgcgttta attcaaaatc tgattggtat ttttcttcgc taattttgta

aacaattatc tgaagtgaat acgcgtggtt tagtataaaa tttagaattt gattttttgg

tcgaaaatta ctttttttta attatcattt tcatctgcaa gtgaaaaatt accaaaaaat

taggggagat acctttcgaa cgtacagtca gacgtgtaaa gtgcaatatt tttttattta

attaaaaaac gcaattttct taggttcgcg ataag

>0028_LG3

---------- ---------- ----tcgcga cttctaatga aataaaaatc ctgaaatttt

acacaccagc ctgtacattg ctaaagatca ttctagattt ttattttttt gttaataaat

aaagtactag ttttgtgagt ttttgggctc taactgttag tttgaaagtt tttagaccaa

atgaatcctt acctgctagc ccatcgaacg ctggtatcag aattataaat tacttggttg

gtgaaataat cgttttttat ttagacgcaa actagacgcg tgatttatta gaagatggta

taacttgcca acaaaggaat cagttaccag tagccgacct ttttgaagcc ccttatttac

agtcttgtaa gaaaattgat gcctgcattg aaatatccca atgaaatctt ttacaatctc

agttgacatt gtcacctaaa tttacgacgc cacagcagct cataaataat ttttaaagtt

aaaatgtaaa agtgcgttta attcaaaatc tgattggtat ttttctacgc taatttcgta

aacaattatc tgaagtgaat acgtgtggtt tagtataaaa ttaagaattt gaatttttgg

tcgaaaatta cttttttt-a attatt-ttt ttatctgtaa gtgaaaaatt accaaaaaat

taagggagat accttttgaa cgtacagtca ggcgtgtaaa atgcaata-t tttttattta

attagaaatt tcgattttct taggtt---- -----

>0006_LG3

aaccttatcg cggacccaag aaaattgcga cttctaatga aataaaaatc ctgaaatttt

acacaccagc ctgtacattg ataaggacca ttttcgattt ttattttttt gttaataaat

aaagtagcag ttttgtgagt ttttgggcac taactgttaa tttaaaagtt tttagaccaa

atgaatcctt acctgctagc tcatcgaacg ctgttatcag aattataaat tacttggttg

gtgaaataac cgttttttat ttagacgcaa actagacgcg tgatttatta gaaaatg--a

taatttgcca acaaagggat ctgttaccag tagccgacct ttttgaagcc ccttatttac

agtcttgtaa gaaaactgat gcctgcattg aaatatccca acgaaatctt ttacaatctc

agtttacatt gtcacctaaa tttacgacgc cacagcaact cataaatatt ttttaaagtt

aaaatgtaaa agtgcgttta attcaaaatc tgattgctat ttttcttcgc taatttcgta

aacaattatc tgaagtgaat aagcgtggtt tagtataaaa tttagaattt gattttttgg

tcaaaaatta cattttttta attatcattt ttatctgtaa gtgaaaaatt accaaaaaat

tagggaagat attttgcgaa cgtacagtca ggcgtgtaaa atgcaatatt tttttattta

attagaaatc gcgattttct taggtgcgcg ataag

>0078_LG7

---------- ---------- ---------- ---------- ---------- ----------

---------- ---------- ---------- ---------- ---------- ----------

---------- ---------- ---------- ---------- ---------- ----------

---------- ---------- ---------- ---------- ---------- ----------

---------- ---------- ---------- ---------- ---tttatta gaagatggta

taatttgcca acaaagggat cagttaccag tagccgatct ttttgaagcc ccttatttac

agacttgcaa taaaactgat gcctgcattg aaatatccca acgaaatctt ttacaatctc

agttcacatt atcacctaaa tttacgacgc cacagcaact cataaataat ttttaaagtt

aaaatgtaaa agtgcgttta attcaaaatc tgattggtat ttttcttcgc taattttgta

aacaattatc tcaagtgaat acgcgtggtt tagtataaaa tttagaattt gactttttgg

t-gaaaatta cttttttt-a attattattt ttatctgcaa gtgaaaaatt accaaaaaaa

taggggagat accttt---- ---agagtca ggcgtgtaaa atgcaatatt tttttattta

gttagaaatt gcgattttct taggtgcgca ataag

>0044_LG3

caccttatcg cgcacccaag aaaatcgcga cttctaatga aataaaaatc ttgaattttt

acacaccagt ctgtacattg ataaagacca ttctcggttt tta-tttttt gttaataaat

caagtaacag ttttgtgagt ttttgggcaa taactgttaa tttg------ ------ccaa

atgaatcctt tacga--atc ccatcgaacg ctggtatcac aattttaaat tacttggttg

gtgaaataat tgatttttat ttagacgcaa accagacgcg tgatttatta gaagatggta

taatttgcca acaaagggat cagttaccag tagccgacct ttttgaagcg ccttatttac

agtcttgcaa taaagctgat gcctgcattg aaatatccca acgaaatctt ttacaatctc

agttcacatt gtcacctaaa ttcacgacgc cacagcaact cataaataat ttttaaagtt

aaaatgtaaa agtgcgttta attcaaaatc tgattagtat ttttcttcgc taatttcgta

aacaatcatc tgaagtgaat tcgcgtggtt tagtataaaa tttaaaattt acctttttgg

taaaaatt-- --tttttttg attattattg ttatcagcaa gtgaaaaatt accaaaaaat

taggggagat acctttcgaa ggtacagtca agcgtgtaaa atgcagtatt tttttattta

attagaaatc gcgattttct taggtgcgcg ataag

>0013_LG3

aaccttatcg cgcacccaag aaaatcgcga cttctcatga aataaaaatc ctgaaatttt

acacatcagt ctgtacattg ataaagacca ttctcgattt ttattttttt gttaataaat

aaagtagcag ttttgtgagt ttatgggcac taactgttaa tttaaaagtt tttagaccaa

aggaatcctt acctgctagt ccatcgaacg ctgg------ ---actaaat taattggttg

gtaaaataat cgttttttat ttagacgcaa accagacgcg tgatttatta aaagatggta

taatttgcca acaaagggat cagttaacag tagccgacct ttttgaagcc tctcatttac

aatcttgcaa taaaactgat gcctgcattc aaatatccca acgaaatctt ttacaatctc

agttcatatt gtcacctaaa tttacgacgc cacagcaact cataaataat ttttaaagtt

aaaatgtaag agtgcgttta attcaaaatc tgattggtat ttttcttcgc taatttcgta

aacaagtatc tgaagtgaat acgcgtggtt tagtataaaa tttagaattt gactttttgg

tcgaaaatta cttttttttg attattattt ttatctgcaa gtgaaaaatt accaaaaaat

taggggagat acctttcgaa cgtacagtca ggcgtgtaaa atgcaatatt tttttattta

attagaaatc gcgattttct tgggtgcgcg ataag

>0020_LG9

aaccttatcg cgcacccaag aaaatcgcaa cttctaatga aataaaaatc ctgaaatttt

acacaccagc ctgtacattg aaaaggacca ttctcgattt ttattttttt gttaataaat

aaagtagcag ttttgtgtgt ttgtgggcac taactgttaa tttaaaagtt tttagaccaa

atgaatcctt acctgctagc ccatcgaatg ctagtatcaa aattataaat tacttggttg

gtgaaataat cgttttttat ttagacgcaa accagacgcg tgatttatta gaagatggta

taattcgcca acaaagggat cagttaccag tagccgacct ttttgaagtc ccttatttat

agtcttacaa taaaactgat gcctgcattg aaatatcaca acgaaatctt ttacaatctc

aattcacatt gtccccaaaa tttacgacgc cacagcaact cataaataat ttttaaagtt

aaaatgtaaa agtgcgttta attcaaaatc tgattggcat ttttcttcgc taatttcata

aacaattatc tgaagtgaat acgcgtggtt tagtataaaa tttagaattt gactttttgg

tcgaaaatta cttcttttta attattattt ttatctgcaa gtgaaaaatt accaaaaaat

tatgggagat actttttgaa cgtacagtca gtcgtgtaaa atgcaatatt tttttattta

attagaaatc gccattttct taggtgcgcg ttaag

>0038_LG3

aaccttatcg cgcacccaag aaaatcgcga cttttaatga aataaaaatc atgaaatttt

acacaccagc ctgtacattg ataacgacca ttctcgattt ttattttttt attaataaat

aaagtagcag ttttgtgagt ttttgggcac taactgttaa tttaaaagtt tttagaccaa

atgaattctt acttgctagc ccatcgaacg ctggtatcag aattataaat tacttggttg

gtgaaataat cgtttcttat t--------- --tagacgcg tgatttatta gaagatggta

taatttgcca acaaatggat cagttaccag tagccgacct ctttgaagcc ccttatttac

agtcttgcaa taaaactgat gcctgctttg aaatatccca acgaaatctt ttacaatctc

agttcacatt gtcaccaaaa tttacgacgc cacagctact cataaataat ttttaaagtt

gaaatgtaaa agtgcgttta attcaaaatc tgattggtat ttttcttcgc tactttcgta

aacaattatc tcaagtgaat acgcatgatt tagtataaaa tttagaattt gactttttgg

tcaaaaat-a tattttttta attattattt ttatctgcaa aagaaaaatt accaaaaaat

tgggggagat acctttcgaa cgtacagtca ggcgtgtaaa atgaaatatt ttt-tatata

---------- ---------- ---------- -----

>0087_LG3

---------- ---------- --tatcgcga cttctaatga aataaaaatc ctg-aatttt

acacaccagc ctgtacattg ataaggacca ttctcgattt ttattttttt gataataaat

aaagtagcag ttttgtgagt ttttgggcac taactgtcaa tttaaaagtt tttagaccaa

atgaatcctt acctgctagc ccatcgaacg ctggtatcag aattataaat tacttggttg

gttaaataat cg-------- --------aa accagacgtg tgatttatta gaagatggta

taatttgcta acaaagggat cagttaccag tagccgacct ttttgaagcc ccttatttac

agtcttgcaa taaaactgat gcctgcattg aaatatccca acgaaatctt ttacaatctc

tgttcacatt gtcacccaaa tttacgacgc cacagcaact cataaataat ttttaaagtt

aaaatgtaaa agttccttta ---------- ---------- ---------- ----------

---------- ---------- ---------- ---------- ---------- ----------

---------- ---------- ---------- ---------- ---------- ----------

---------- ---------- ---------- ---------- ---------- ----------

---------- ---------- ---------- -----

>0023_LG5

aaccttatcg cgcacccaag aaaattgcga cttataatga aataaaaatc ctgaaatttt

gcacagcagc ctgtacatcg ataaggacca ttctcgattt ttattttttt gttaataaat

aaagtagcag ttttgtgagt ttttgggcac taactgttaa tttaaaagtt tttagaccaa

atgaatcctt acctgctagc tcatcgaagg ctggtatcag aattataaat tacttggttg

gtgaaataat cattttttat ttagacgcaa actagacgcg tgatttatta aaagatggta

taatttgcca acaaagggat cagttaccag tagccgacct ttttgaagcc ccttatttac

agtcttgcaa taaaactgat gcctgcattc aaatatccca acgaaattat ttacaatctc

agttcacatt gtcacctaaa tttacgaagc cacagcaact cacaaataat ttttaaagtt

aaaatgtaaa agtgcgttta attcaaaatc tgattggtat ttttcttcgc tattttcgta

aacaattatc tgaagtgaat acgcgtggt- ---------- tttagaattt aattttttgg

tcgaaaatta ctttttttta attattattt ttatctgtaa gtgaaaaatt accaaaaaat

taatggagat acctttcgaa cgtacagtca ggggtgtaaa atgcaatatt tttttattta

attagaaatc gcgattttct ta-------- -----

>0018_LG7

aaccttatcg cgcacccaag aaaatcgcga cttctaatga aataaaaatc ctgaaatttt

acacaccagt ctgtacattg ataaggacca ttcttgattt ttattttttt gttaataaat

aa------ag ttttgtgagt ttttgggcac taactgttaa tttaaaagtt tttagaccaa

atgaatcctt acctgctagc ccatcgaacg ctggtgtctg aattataaat tacttggttg

gtgaaataat cgttttttat ttagacgcaa accagacgct tgatttatta gaagatggta

taatttgcca acaaagggat cagttaccag tagccgacct ttttgaagcc ccttatttac

agttttgcaa ttaaactgat gcctgcattg aaatatccca acgaaatctt ttacaatctc

agtttacatt gtcatgtata tttacgacgc cacagcaact tataaataat ttttaaagtt

aaaatgtaaa agtgcgttta attcaaaata tgattggtat ttttcttccc taatttcgta

aacaattatc tgaggtgaat acgcgtggtt tagtataaaa tgtataattt aactttttgg

tcgaaaatta cttttttt-g attattattt ttatctgcaa gtgaaaaatt accaaaaaat

taggggagat acctttcgaa cgtacagtca ggcatgtaaa atgcaatatt cttttattta

attagaaatc gtgattttct taggtgtgcg ataag

>0009_a_LG2

aaccttatcg cgcacccaag aaaatctcga tttctaatga aataaaaatc ctgaaatttt

acacaccagc ctatacattg atatggacca ttctcgcttt ttattttttt gttaataaat

aaagtagcag --ttgagagt ttttgggcac taactgttaa tttaaaagtt tttagaccaa

atgaatcctt atctgctagc ccatcgaacg ctggtatcag aattataaa- ---ttggttg

gtgaaataat cgttttttat ttagacgcaa accagacgcg tgatttatta gaagatggta

taatttgcta acaaagggat cagttaccag tagccgacct ttttgaagcc ccttatttac

agtcttgcaa taaaactgat gcctgcatta aaatatccca acaaaatctt ttacaatctc

agttcacatt gtcacctaaa tttacgacgc cacagcaact cataaataat ttttaaagtt

aaaatgtaaa agtgcgttta attcaaaatc tgaccggtat ttttcttcgc taatttcata

aacaattatc tgaagtgaat acgcgcggtt tagtataaaa tttagaattt gactttttgg

tcaaaaatta cttttttt-t attattattt ttatctgcaa gtgcaatatt accaaaaaat

tagaggagat acctttcgaa cgtacagaca ggcgtgtaaa atgcaatatt tttttattta

attagaaatc gcaattttct taggtgcgcg ataag

>0019_LG2

aacctaatcg cgcacccaag aaaatcgcga cttctaatga aataaaaatc ctaaaatttt

acacaccagc ctgtacattg ataaggaaca ttctcgattt ttatttgttt gttaataaat

aaagtagcag ttttgcgagt ttttgagcac taactgttaa tttaaaagtt tttagaccaa

atgaattctt acctgctagc ccatcgaacg caggtatcag aattatgaat tacttggttg

gtgaaataat cgttttttat ttagacgcaa accagatccg tgattcatta gaagatggta

taatttgcta ataaagggat cagttaccag tagccgacct tttagaagtc ccttatttac

agtcttgtaa taaaactgat gcctgcattg aaatatccca acgaaatctt atacaatctc

agttcacatt gtcacctaaa tttacgacgc cacagcaact cataaataat ttttaaagtt

aaaatgtaaa aatgcgttta attcaaaatc tgattggtat ttttctttgc taatttcgta

aacaattatc tcaagtgaat acgcttggtt tagtatcaaa tttagaatta gattttttgg

tcaaaaatta cttttttt-a attattattt ttatctgcaa gtgaaaaatt accaaaaaat

ttgaagagat acgtttcgaa cgtacagtca ggcgtgtaaa atgcaatatt tttttaatta

attagaaatc gcgattttct taggtgcgcg atgag

>0016_LG3

aaccttatcg cgcacccaag aaaatcgcta cttctaatga aataaaaatc ctgaaatttt

acacaccagc ctgtacagtg ataaaaacta ttctcgattt ttatattttt gttaataaat

aaagtagcag ttttgtgagt ttttgggcac taactgttaa tttaaaagtt tttagaccaa

atgaatcctt acctgttagc ccatcgaact ctggtatcag aattataaat tacttggtta

gtgaaataat cgttttttat gtagacgcaa actaaacgcg tgatttatta gaagatggta

taatttgcca aaaaaggaat cagttaccag tagccgacct ctttgaagcc ccttatttac

agtcttgcaa taaaactga- ---tgcattg aaatatccca acgaaatctt ttataatctc

agttcacatt gtcacctaaa tttacgacgc cacagcaact cataaataat ttttaaagtt

aaaatgtaaa agtgcgttta attcaaaatc taattgggat ttttcttccc taatttggta

aacaattatc tgaagtgaat acgcgtggtt tagtataaaa tttagaatta gattttttga

tcgaaaatta ctttttttta attattattt ttacctgcaa gtgaaaaatt accaaaaaat

taggggagat accttttgat cgtacagtca ggcgtgtaaa atgcaata-t tttttattta

attagaaatc gcgattttct taggtgcgcg ataat

>0047_LG10

---------- ---------- ---------- ---------- ---------- ----------

---------- -tgtacattg ataaggacca ttcttgattt ttattttttt gttaataatt

aaagtagcag ttttgtgaga ttttgggcac taattgttaa tttaaaagtt ttaagactaa

gtgattcctt acgtggtagc ccatcgaacg ctggtatcag aattataaat tacttggttg

gagaaataat cgttttttat ttagacgcaa accagacgcg tgatttatta gaagatgtga

aaatttgcta acaaagggat cagttaccag tagccgacct ttttgaaacc ccttatatac

agtcttgcaa taaaattgat gcctgcattg aaatatccca acgaaatctt ttacaatctt

agttcacatt gtcagcgaaa tttacgacgc cacagcagct cttaaataat ttttaaagtt

aaaatgtaaa agtgcgttta attcaaaatc tgattggtat ttttcttcgc taatttcgta

aacaatcatc tgaagtgaat acgcgtagtt tagtataaaa tttagaattc gactttttgg

tcgaaaatta cttttttt-g attattattt ttatctgcaa gtaaaaaatt accaaaaaat

tagaggagat acctttcgaa cgtacagtcc ggcgtgtaaa atgcaatatt tttttattta

attaaaaatc gcggttttct taggtgcgca ataag

>0045_LG2

---------- ---------- ---------- ---------- ---------- ----------

---------- -----cactg ataaggacca ttctcgattt ttattttttt gtcaataaat

aaagtagcag cttggtgagt ttttggacac taactgttaa tttaaaagtt tttagaccaa

atgaatcctt acctgctagc ccatcgaacg ctggtatcag aattataaat tacttggttg

gtgaaataa- --ttttttat ttagacgcaa actaaacgcg tgatttatta gaagatggta

taatttgcca acaaagggat cagttaccag tagccgatct atttgaagcc ccttatatac

agtcttacaa ttaaactgat gcttgcattg aaatatccca aagaaatctt ttacaatctc

agttcacatt gtcacctaaa tttacgacgc cacagcaact cataaataat ttttaaagtt

aaaatgtaaa tgtgcgttta attcaaaatc tgattggtat ttttcttcgc taatttcgta

aacaattgtc tgaagtaagt acgcgtggat tagtataaaa tttagaattt gacttttttg

tcgaaaatta cttttttt-a attattattt ttatctgcaa gcgaaaaatt accaaaaaat

tagaagagat acctttcgaa cgtacagtca ggcgtgtaaa atgcaatatt tttttaatta

aataaaaatc gcgattttct taggtgcgcg ataag

>0017_LG4

aaccttatcg cgcacccaag aaaatcgcga cttctaatga ataaaaaata ctgaagtttt

acacaccagc ctgtacattg ataaggacca ttctcgattt ttattttttt gttaataaat

aaagtagcag ttttgtgagt ttttgggcac taacttttaa tgaaaaagtt tttagaccaa

atgaatcctt acctgctagc ccatcgaacg ctagtatcaa aattataaat tacttggctg

gtgaaataat cgttttttat ttagacgcaa accagacgcg tgatttatta gaagatggta

taatttgcca acaaagggat cagttaccag tagccgacct ttttgaaacg ccttatttac

agtcttgcaa taaaactgat gcctgcattg aaatatccca acgaaatctt ttacaatctc

agttcaca-- gtcacctaaa tttacgacgc tacagcaact cataaatatt tttttaagtt

aaaatgtaaa agtgcgttta attcaaaatc tgattggtat ttttcttcgc taattttgta

aacaattatc tgacgtgaat acccgtggtt tagtataaaa tttagaattt gactttttgg

tagaaactta cttttttt-a attattattt ttatctgcaa gtggaaaatt accaaaaaat

taggggaaat acctttcgaa cgtatagtca ggcgtgtaaa atgcaatatt tttttattta

attagaaatc gcgattttct taagtgcgcg ataag

>0036_LG9

---------- ----ccaaag aaaatcgcga cttctaatga aataaaaatc gtgaaatttt

acacaccaat ctgtacactg ataagaacca ttctcgattt ttattttttt gttaataaat

gaagtagcag ttttgtgagt ttttgggcac taactgttaa tttaaaagtt tttagaccaa

atgaatcctt acctgctagt acatcgaacg ctggtatcag aattataaat tacttggttg

gtgaaataat cgttttttat ttagacgcaa actagacgcg tgatttatta gaagatggta

taatttgcca acaaagggat cacgagccag tagccgacct ttttgaagcc ccttatttac

agtcttgcaa taaaactgat gactgcattg aaatatccca acgaaatctt tta------c

agttcacatt gtcacctaaa tttacgacgc cacagcaact cataaataat ttttaaagtt

aaaatgtaaa agtgcgttta attcaaaatc tgattggtat ttttcttcgc taattccgta

aacaattatc tgaagtaaat acgcgtggtt tagtataaaa tttagaattt gactttttgg

tcgaaaatta ctttttttta attatcattt ttatctgcaa gtgaaaaatt accgaaaaat

taggggagat accttt---- cgtacagtca ggcgtgtaaa atgcaatatt tttttattta

attagaaatc gcgattttct taggg----- -----

>0035_LG9

---------- --aacccaag aaaatcgcga cttctaatga cacaaaaatc ctgaaatttt

acacaccagc ctgaacattg ataagaacca ttctcgattt ttgttttttt gttaataaat

aaagtagcag ttttgtgagt ttttgggcac taactgttaa tttgaaagtt tttagaccaa

atgaatcctt acctgctagc ccatcgaacg ctggtatcag aattatacat tacttggttg

gtgaaataat cgttttttat ttagacgcaa accagacgcg tgatttatta gaagatggta

taatttgcca acaaagggat tagttaccag tagccgacct ttttgaagcc ccttatttac

agtcttgcaa taaaactgat gcctgcattg aaatatccca acgaaatctt tta------c

agttcacatt gtcacctaaa tttacgacgc cacagcaact catacataat ttttaaggtt

aaaatgtaaa agtgcgttta attcaaaatc tgattggtat ttttcttcgc tgatttcgta

aacaattacc tgaagtgaat acgcgtggtt tagtataaaa tttagaattt gattttttgg

tcgaaaatga ctttttttta attattattt tt-----taa gtgaaaaatt aacaaaaaat

tagaggagat acctttcgaa cgtacagtca ggcgtgtaaa atgcaata-t tttgtattta

attagaaatc gcgatttttt ttagcgcg-- -----

>0037_LG3

---------- ---------- -aaatcgcga cttctaatga aataaaagtt ttgaaatttt

acataccacc ctgtacgttg ataacgacca ttcttgattt ttattttttt gttaataaat

aaagtagcag ttttgtgagt ttttgggcac aaactgttaa tttaaaagtt tttacaccaa

atgaatcctt acctactagc ccgtcgaacg cttttatcag aattataaat tacttgattg

gtgaaataat cgtttttcat ttagtcgcaa actagacgcg tgatttatta gaagatggta

taatttgtca acaaagggat cagttaccag tagccgacct ttttgaagcc ccttatttac

agtcttgcaa taaaactgaa gcctgcattg aaatatccca acgaaatctt ttacaatctc

aattcacatt atcacctaaa tttacgacgc cacagcaact cataaataat ttttaaagtt

aaaatgtaaa agtgcgttta attcaaaatc tgattggtat ttttcatcgt taatttcgta

aacaattatc tgaagtgaat acgcgtggtt taggataaaa tttagaa-tt gactttttgg

tcgaaatt-- --ttttttta actatttttt taatctgcaa gtgaaaaatt accaaaaaat

taggggagat actttttgaa cgtacagtaa ggcgtgtaaa atgc-atatt tttttattta

attagaaatc gcgatttttt taggtgt--- -----

>0027_LG3

aaccttatcg cgcactcaag aaaatcgcga cttctaatga aataaaaatc ctaaaatttt

acacaccact ctatacattg atatggacaa ttctcgattt tcattttttt gttaataaat

aaagtagcag ttttgcgagt ttttggacat taactgttaa tttaaaagtt tttagatcaa

atgaatcctt acctgctagc ccatcgagcg gtggtatcag aattaaaaat tacttgtttg

gtgaaataat cgttttttat ttagacgcaa accagacgcg tgatttatta gaagatggta

taatttgcca acaaagggat cagttaccag tagccgacct ttttggagtc ccttatttac

agtcttgcaa taaaactgat tcctgcattg aaatatccca acgaaatctt ttacaatctc

agttcacatt gtcacctaaa tttacgacgc cacaacaact cataaataat ttttaaagtt

aaaatggaaa agtgcgttta attcataatc tgattggtat tttttttcgc taatttcgta

aacaattatt tgaagtgaat acgcgtggtc tggtataaaa tttataattt gactttttgg

tcgaaaatta ctttttttta attattattt ttatctgcaa gtgaaaaatt accaaaaaat

tagaggagat actttttgaa ggtacagtca ggcgtgtaga atgcaatact tttttattta

attagaaatc gcgattttct taggtgcgcg ataag

>0065_LG3

aaccttatcg cgcacccaaa aaaatcgcta cttctaatga aataaaaatc ctgaaatttt

atacattagc ctgtac---- ---------a ttctagattt ttattttttt gttaataaat

aaagtagcag ttttgtgagt ttttgggcac taactgttaa tgtaaaagtt tttagatcaa

atgagtcctt acctgctagc ccatcgaacg ctggtatcag aattatgaat tatttggttg

gtgaaataat cgttttttat ttagacgcaa actagacgcg tgatttatta gaagatggta

taatttgcca acaaagggat cagttaccag tagccgacct ttttgaagcc ccttatttac

agtcttgcaa taaaactgat gcctgcattg aaatattcca acgaaatctt ttacaatctc

agttcacatt gtcacctaca ttt------- ---agcaact cataaatatt tttcaaagtt

aaaatgtaaa agtgccttta attcaaaatc tggttggtat ttttcttcgc taatttcgta

aacaattatc tgaagtgaat acgcgtggat tagtataaaa tttagaattt gactttttgg

tcgaaaatta ctttttttta attattattt taatctgcaa gtgaaaaatt accaaataaa

ttt------- ---------- ---------- ---------- ---------- ----------

---------- ---------- ---------- -----

>0005_LG3

aaccttatcg cgcacccaag aaattcgcta cttctaatga aataaaaatc ctaaaatttt

acacaccagc ctgtacacag ataaggacca ttctcgattt ttattttttt cttaatgaat

aaagtagcag ttttgtgagt ttttgggcac t--------- ----aaagtt tttagaccaa

atgaattctt atctgctagc ccatcgaacg ctggtatcag aattataaat tacttggttg

gtgaaataat cgttttttat ttagacgcaa accagacgcg tgatttatta gaagatggta

ttatttgcct acaaagggat cagttaccag tagccgacct ctctgaagct ccttatttac

agtcttgcaa taaaactgat gcctgcattg aaatattccc acgaaatctt ttacaatctc

agttcacatt gtcacctaaa tttacgacgc caaagcaact tataaataat ttttaaagtt

aaaatgaaaa agtgcgttta atttaaaatc tgatt-gtat ttttctttgc taatttcgta

aacaattatc taaagtgaat acgcgtggtt tagtataaaa tttagaattt gactttttgg

tcgaaaatta ctttttttta attattattt ttatctgcaa gtgaaaaatt accaaaaaat

tagaagagat acctttcgaa cgtacagtca gtcgtgtaaa atgcaata-t tttttattta

attagaaatc gcgattttct taggtgcc-- -----

>0042_LG3

---------- ---------- ---------- -----gatga aataaaaatc ctgaaatttt

acataccagc ctgtacgttg ataaggacca ttcttgattt ttattttttt gttaataaat

aaagtagcag ctttgtgagt ttttggacac taattgttaa tataaaagtt tttagaccaa

atgaatccct acctgctagc tcatcgaatg ctggtatcag aattataaat tacttggttg

gtgaaataat cgtttattat ttagaggcaa actagac-cg tgatttatta gaagatggta

taatttg--- ---------t cagttaccag tagccgacct ttttgaagcc ccttatttac

agtcttgcaa taaaactgat gcctgcattg aaatatccca acgaaatctt ttacaatctc

agttcacatt gtcacctaaa tttacgacgc cacagcaagt cataaataat ttttaaagtt

aaaatgtaaa agtgcgttta cttcaaaatc tgattggtat ttttcttcgc taatttcgta

aacaattatc tgaagtgaat acgcgtggtt tagtataaaa tttagaattt gattttttgg

ttgaaaatta ctttttttta attactattg ttttctgcaa gtgcaaaatt accaaaaaat

tagaggagat atccttcgaa cgtacagtca ggcgtgtaaa atgcaatatg tttttattta

attagaaatc gcgattttct taggtgcgcg at---

>0011_LG2

aaccttatcg cacacccaag aaaatcacga cttgtaatga aataaaaatc ctgatatttt

acacatcagc ctatacatta ataaagacca ttctcgattt ttattttttt gttaataaat

aaagtagcag ttttgtgagt ttttgggcac taactgttaa tttcaaagtt tttagaccaa

atggatctct acatgctagc ccatcgaacg ctgttatcag aattataaat tacttggttg

gtgaaataat cgttttttat ttagatgcaa actagacgcg tgatttattg gaagatggta

taatttgcca acaaagggat caattaccag tagccgacct ttatgaagct tcttatttac

agttttgcaa taaaactgat gccagcattg aaatatccca acgaaatctt ttacaatctc

agttcacatt gtcacctaag tttacgacgc cacagcaact caaaaataat ttttaaagtt

aaaatgtaaa aatgcgttta attcaaaatc tgatt-gtat ttttcttcgc taatttcata

aacaattatc tgaagtgaat acgcgtggtt tagtataaaa tttagaattt gactttttgg

tcaaaaatta cttttttt-- attattattt ttatctgcaa gtgaaaattt accaaaaaat

tagaggagat acctttcgaa cgtacagtga ggcgtgtaaa atgcaatatt tttttatttc

attagaaatc gcgattttct ta-------- -----

>0082_LG8

aaccttatcg cgcacccaag aaaatcgcga cttctaatga aataaaaatc ctgaaatttt

acacaccagt ctgtacattg ataaggacca ttctcgattt ttattgtttt gttaataaat

aaagtagcag ttttgtacgt ttttgggcac taactgttaa tttaaaagtt tttagtccaa

atgaa----- ---------c ccatcgaaca ctggtatcag aattataaat tacttggttg

gtaaaataat tgtttttaat ttagacgcaa actagacgct tgatttatta gaagatggta

tgatttgtca acaaagggat cagttaccag tagccgacct ttttgaagcc ccttatgtac

agtcttgcaa taaaactgat gccggcattg aaatatccca acgaaatctt ttacaatctc

agttcacatt gtcacctaaa tttacgacgc ca---caact cataaataat ttttaaagtt

aaaatgtaaa agtgctttta attcaaaatc ---------- ---------- ----------

---------- ---------- ---------- ---------- ---------- ----------

---------- ---------- ---------- ---------- ---------- ----------

---------- ---------- ---------- ---------- ---------- ----------

---------- ---------- ---------- -----

>0029_LG3

aaccttatcg gacacccaag aaaatcgcta cttctaatga aataaaaatc ctgaaatttt

acacaccacc ctgtacattg ataaggacca atctcgattt ttattttttt -ttaataaat

aaagtagtag ttttatgagt tcttgggcac taactgttaa tttaaaagtt tttggactaa

atgaattctt acctgctagc ccatcgaact ctggtatcag atttataaat tacttggttg

gtgaaataat cgttttttat ttcgacgcaa accagacgcg tgatttgtta gaagatggta

taatttgcca acaaagggat cagttaccag tagccgacct ttttgaagtc ccttatgtac

agtcttgcaa taaaactgat gtctgcattg aaatatccca acgaaatctt ttacaatctc

agttcacatt gtcacctaaa tttacgacgc cacagcaact cataaataat tttttaagtt

aaaatgtaaa agtgcgttta attcaaaatc tgattggtaa ttttcttcgc taatttcgta

gacaattatc tgaagtgaat acgcgtggtt tagtatgaaa ttcagaattt gactatttgg

ttgaaaatta ctttttttta attcttatct ttatcagtaa gtgaaaaatt accaagaaat

taggaaagtt ccctttcaaa cgtacagtca ggcgtgtaaa atgcaatttt cttatattta

tttagaaatc gcgattttct taggtgcacg ataag

>0041_LG3

aaccttatcg cgcacccaag aaaatcgcga cttctaatga aataaaaatc ctgaaatttt

acacaccagc ctgtacatt- ---------- -----gattt ttattttttt gtt----aat

aaagtagcag ttttgtgagt ttttgggcac taactgttaa tttaaaagtt tttagaccaa

atgaatcctt acctgctagc ccatcgaa-- ctggtatcag aattataaat tacttggttg

gtgaaataat cgttttttat ttagacgcaa actagacgcg taatttatta caagatggta

taatttgcca acaaagggat cagttaccag tagctgacct ttttgaagcc tcttatttac

agtcttgcaa taaaactgat gcctgcattg aaatatccca acgaaatctt ttacaatctc

agttcccatt gtcacctaaa tttaacacgc cacaacaact gataaatgat ttttaaagtt

aaaatgtaaa agtgcgttta attcaaaatc tgattggtat ttttctttgc taatttcgta

aacaattatc tgaagtgaat acgcgaggtt tagtataaaa tttcgaattt taatttttgg

tcgaaaatta ctttttttta attattattt ttatccgcat gtgaaaaatt accaaaaaat

tgggggagat accttttgaa cgtacagtca ggcttgtaaa atgcaatatt tttttattta

attat----- ---------- ---------- -----

>0034_LG4

---------- -gcacccaag aaaaccgcga cttctaatga aataaaaatc ctgaaatttt

acacaccacc ctgtacattg ctaaggatca ttctcgattt ttgttttttt gttaataaat

aaagtagcag ttttgtgtgt ttttgggcac taactgttaa tttaaaag-t tttagaccaa

atgaatcctt acttgctagc ccatcgaatg ctggtatcag aattataaat cacttggttg

gtgaaataat cgttttttat ttagaggcaa accagacgcg tgatttatta gaagatggta

taatttgcca acaaagggat cagttaccag tagctgactt ttttgaagcc tcttatttac

agtcttgc-a taaaactg-- ------attg aaatatccca acgaaatctt ttacaatctc

agtccacatt gtcacctaaa tttacgacgc cacagcaact cataaataat ttttaaagtt

aaaatgtaaa agtgcgttta attcaaaatc tgattggtat ttttcttcgc taattttata

aacaattatc tgaa-tgaat acgcgtggtt tagtataaaa tttagaattt gactttttgg

tcgaaaatta c-gattttta attattattt ttatctgcaa gtaaaaaatt accaaaaaat

tagaggagat acctttcgaa cgtacagtca gtcgtgtaaa atgcaatatt tttttattta

attaaaaatc gcgattttct tagg------ -----

>0025_LG4

aaccttatgg cgcacccaag aaaatcgcca cttctaatga aataaaaatc ctgaaatttt

acacaccacc ctgtacattg ataaggacca ttctcgattt ttattttttt gttaataaat

aaagtagcag ttttgtgtgt ttttgggcac taactgttaa tttaaaagtt tttagaccaa

ataaatcctt acctactagt ccatcgaatg ctggtatcag aaatataaat tacttggttg

gtgaaataat cgttttttat ttagaggcaa accagacacg tgatttatta gaaaatggta

taatttgcca acagagggat cagttaccag tagccgactt ttttgaagcc tcttatttac

agtcttgcaa taaaactg-- ------attg aaatatccca acgaaatctt ttacaatctc

agttcacatt gtcacctaaa tttacgacgc cacagcaaca cataaataat ttttaaagtt

aaaatgtaaa agtgcgttta attcaaaatc tgattggtat ttttcttcgc taattttata

aacaattatc tgaa--gaat acgcgtggtt tagtataaaa tttagaattt gactttttgg

tcgaaaatta c-ttttttta attattattt tcatctgcaa gtgaaaaatt accaaaaaat

taagggagat acctttcgaa cgt--agtca ggcgtgtaaa ataaaatatt tttttattta

attacaagtc gcgatttttt --agttcgcg ataag

>0030_LG5

aaccttgtcg cgcacccaag aaaatcgcga cttctaatga aataaaaatc ctgaaatttt

acacaccagc ctgtacatta ataaggacca ttctcgattt aaa---tttt gtaaataaat

gaagtagcag ttttgtgagt ttttgggcat taactgttaa tttaaaagtt tttagaccaa

atgaatcctt atctgctagc ccattgaacg ctgatgtcag aattataaat tacttggttt

gtgaaataat cgttttttat ttagacgcaa actagacgcg tgatttatta gaagacggta

taatttgcca acaaagggat cagttaccag tagccgacct ttttgaagcc ccttatttac

agtcttgcaa taaaactgat gcctgcatag aaatatctca acgaaatctt ttacaatctc

agttcacatt gtcacctaaa tttacgacgc cacagcaact c--aaataaa ttttaaagtt

aaaatgtaac agtgcgttta attcaaaatt tgattggtat tttttttcgc taatttcgta

aataa-tatc tgaagtgaat acgcgtggtt tagtataaaa tttagaattt gactttttgg

tcgaaaatta ctttttttca attattattt ttatctgcaa gtgaaaaatt accaaaaaat

taggagagat atctttcgaa cgtacagaca ggcgtgtaaa atgcactatt tttttattta

t--------- ---------- ---------- -----

>0079_LG3

---------- ---------- ---------- ---------- ---------- ----------

---------- ---------- ---------- ---------- ---------- ----------

---------- ---------- ---------- ---------- ---------- ----------

---------- ---------- ---------- ---------- ---------- ----------

---------- ---------- -----cgcaa actagacgc- tgatttatta gaagatagta

taatttgcca acaaagggat cagttaccag tatcctacct ctttgaagcc ccttatttac

agttttgcaa taaaactaat gcctgcattg aaatatccca acgaaatctt ttacaatctc

agttcacatt gtcgcctaaa tttacgacgc cacagcaact tataaataat ttttaaagtt

aaaatgtaaa tgtgcgttta attcaaaatc tgattggtat ttttcttcgc taaattagta

aacaattatc tgaagtgaat acgcatggtt tagtataaaa tttagaattt gactttttgg

tcaaaaatta c-ttttttta attattattt ttatctgcaa gtgaaaaatt accaaaaaat

ttggggagat acctttcgaa cgtacagtca ggcgtgtaaa atgcaatatt tttgtattta

attagaaatc gcgattttct tagatgcgcg attag

>0049_LG3

-----tatcg cgcacccaag aaaatcgcga cttctaatga aataaaaatc ctaaaatttt

acacaccagc ctgtacattg attaagacca ttcccgattt t--ttttttt gttaataaat

gaagtatcag ttttgtgaag ttttgggcat taactgttaa tttaaaagtt t---------

--------tt acctgctagc ccatcgaacg ctgg------ ---tataaat tacttggttg

gtgaaataat cgttttttat ttagacgcaa actagacgcg tgatttatta gaagatagta

taatttgcca acaaagggat cagttaccag taactgacct ttttgaagcc ccttatttac

agtcttgcaa taaaactgat gcctgcattg aaatatccca acgaaacctt ttacaatctc

agctcacatt gtcacctaaa tttaggacgc cacagcaact cataaataat ttttaaagtt

aaaatgtaaa agtgtgttta attcaaaatc tgattggtat ttttcttcgc taatttcgta

aacaataatc tgaagtgaat acgagtggtt cagtataaaa tttagaattt gattttttgg

tcaaaaatta c-ttttttta attattattt ttatctgcaa gtgaaaaatt accaaaaaat

taggggagat acctttcgaa cgtacagtca ggcgtgt-aa atgcaata-t tttttattta

attagaaatc gcaattttct taggtgcgcg ataag

>0040_LG3

aacattatcg cgcacccaag aaaaacgcga cttctaatga aataaaaatc ctgaaatttt

acacaccact ctgtacattg gtaaagacca ttctcga-tt ttattttttt gttaataaat

aaagtagcag ttttgtgagt ttttgggcac taactgttaa tttaaaagtt tttaga----

---------- ---------- ccatcgaacg ttggtatcag aattataaat tacttggttg

gtgaaataat cgttttttgt ttagacgcaa actagacgca tgatttatta gaagatggta

taatttgcca acaaagggat cagttaccag tagccgacct ttttgaagcc ccttatttac

agtcttgcaa taaaaccgat gcttgcattg aaatatccca acgaaatctt ttacaatctc

agttcacatt gtcacctaaa tttacgacgc cacagcaact c--aaataat ttttaaagtt

aaaatgtaaa agtgcgtttg tttcaaaatc tgattggtat ttttcttcgc taattttgta

aacaactatc tgaagtgaat acgcgtggtt tagtataaaa tttagaattt gattttttga

tcgaaaatta ctttttttta attattattt ttatctgcaa gtgaaaaatt accaaaaaat

ttggggaaat accattcgaa cgtacagtca ggcgtgtaaa atgcaata-t tttttattta

attagaaatc acgattttct taggtgcgtg ataag

>0026_LG5

aaccttatcg cgcacccaag aaaaatgcga cttctaatga aataaaaatc ctgaaatttt

acacactagc ctgtacattg ataaggacca ttcaggattt ttattttttt gttaataaat

aaagtagcag ttttctgagt ttttgggcac taactgttaa tttgaaagtt tttagaccaa

atgattcctt acctgctagc ccatcgaacg ctggtatcag aattataaat tacttggttg

gtgaaataat cgttttttat ttagacgcaa actagacgcg tgacttatta gaagatggta

taatttgcca acaaagggat cagttaccag tagccgacct ttttgaag-c tcttatttac

a-----gcaa taaaactaat gcctgcattg aaatatccca acgaaatttt ttacaatctc

agttcacatt gtcacctaaa tttacgacgc cac------- -tcaaataat ttttaaagtt

aaaatgtaaa agtgcgttta attcaaaatc tgattggtat ttttcttcgc taatttcgta

aacaattatc tgaagtgaat acgcgtggtt tagtataaaa tttagaattt gactttttgg

tcgaaaatta c-ttttttta attatcattt ttatctgcaa atgaaaaatt accaaaaaat

tagggtagat acctttcaaa cgtacagtca ggcgcacaca atgcaatatt tttttattta

attagaaatc gcgattttct taggtgcgcg ataag

>0062_LG9

---------- ---------- ---------- ---------- ---------- ----------

---------- ---------- ---------- ---------- ---------- -------aat

aaagtaacag ttttatgagt ttttgggcac taactgttga tttaaaagtt tttacactaa

gtgaatcctt agctgctagc ccatcaaacg ctggtatcag aattataaat tacttggttg

gtgaaataat cgttttttat ttagacgcaa actagacgtg tgatttatta gaagatggta

taatttgcca acaaagggat cagttaccag tagccgacct ttttgaag-c ccttatttgt

aatcttgtaa tgaaactgat gccagcattg aaatatccca acgaaatctt ttacaatctc

agttcacatt gtcacctaaa tttacgacgc cacagcaact caaaaataat ttttaaagtt

aaaatgttaa agtgcgttta attcaaaatc tgattggtat ttttctgcgc taatttcgta

aacaattatc tgaagtgaat acgcgtggtt tagtataaaa tttagaatta gattttttgg

tcgaaaatta t-ttttttta attatcattt ttatctgcaa atgaaaaatt accaaaaaat

taggggagat accattcgaa cgtaccgtca gacgtataaa atgcaatatt tttttattta

attagaaatc gcaattttct taggtgcgcg ataag

>0033_LG5

aaccttatcg cgcacccaag aaaatcgcga cttcaaatga aataaaaatc ctaaaatttt

acacaccagc ctgtacatta ataaggacca ttcttgattt tta-tttttt tttaataaat

gaagtagcat ttttgtgagt ttttgggcat taactgttaa tctaaaagtt tttagaccaa

atgaatcctt atctgcaagc ccaggaaacg --ggtatcag aattataaat tatttggttg

gtaaaatatt cgttttttat ttagacgcaa actagacgcg tgatttatta gaagatggta

taatttacca acaaagggat aagttaccag tagccgacct ttttgaagcc tcttatttac

agtcttgcaa taaaactgat gccggcatta aaatatccca acgaaatctt ttacaatctc

agttcacatt gtcaccgaaa tttacgacgc cacagcaact cataaataat ttttaaagtt

gaaatataaa agtgcgttta atacaaaatc tgattggtat ttttcttcgc taatttcgta

aacaattatc cgaagtaaat acgcgtggtt tagtataaaa tttagaattt gactttttgg

tcgaaaatta cttttttt-t attattattt ttatctgcaa gtaaaaaatt atcaaaaaat

taggggagat acctttcgaa ggtacagtca ggcgtataaa atgcaacatt cttttattta

attagaaatt gcgattttct taggtgtgcg ataag

>0083_LG3

---------- ---------- ---------- ---------- ---------- ----------

---------- ---------- ---------- ---------- ---------- ----------

---------- ---------- ---------- ---------- ---------- ----------

---------- ---------- ---------- --tgtatcag aattataaat tacttggtta

gtaaaataat cgtt------ ---------- ---------- ---tttatta gaaaatggta

taatttgcca acaaaggaat cagttaccag taaccgacct ctttgaagcc ccttatttac

agttttgcaa taaaactgat ggatatattg aaatatccca acgaaatctt ttacaatctc

agttcacatt gtcacctaaa tttacgacgc cacagcaact cataaataat ttttaaagtt

aaaatgttaa agtgcgttta atttaaaatc tgattggtat ttttcttctc taattttata

aataattatc tgaagtgaat acgcgtggtt tattataaaa tttagaattt gacttttt--

ttcgaaaata ctttttttta attattatta ttatctgcaa gtgaaaaatt accaaaaaat

taggggagat acctttcgaa cgtacagtca ggcgtgtaaa atgcaatatt tttttattta

attagaaatc gcgattttct tagatgcgcg ataag

>0039_LG2

aaccttatcg cgcacccaag aaaatggcga cttctaatga aataaaaatc ctgaaatttt

acacagtagc ctgtacgttg ataaagacca ttctcaattt ttgtgttttt gtaaataaat

gaagtagcag ttttgtgagt ttttgggcac taactgttat tttgaaagtt ttcagaccaa

ataaattctt acctgctagc ccatcgaacg ctggtatcag aattataaat tacttggttg

gtgaaataat cgttttttaa ttagacgcaa accagacgcg tgatttatta gaagatggta

taatttgcca acaaaaggat cggttaccag tagccaacct ttttgaagcc ccttatttac

agttttgcaa taaaactgat gcctgcattc aaatatccca acgaaatctt ttacaatctc

agttcacatt gtcacctaaa tttacgacgc cacagctacc cataaataat ttttaaaggg

aaaatgtaaa aatgcgtttt attcaaaatc tgattggtat ttttcttcac taatttcgta

aacaattatc tgaagtgaat acgcgtggtt tagtataaga tttagaattt gactttttag

tcaaaaatta ctttttttta attattattt ttatgtgtaa gtgaaaaaaa gtgaaaagtg

aaaagtagat acctttcgaa cggacagtca ggcgcgtaaa atgcaatgtt tttttattta

attagaaata acgattttct taggtgcgcg ataaa

>0068_LG3

aaccttatcg cgcaccgaag aaaatcgcga cttctaatga aataaaaatt ctgaaatttt

acacatcagc ctgtacgttc ataaggacca ttctcagttt ttattttttt gttaataaat

aaagtagcag ttttgtgagt ttttaggcac taactgttaa tttaaaagtt tttaaactaa

ataaatcctt acctgctagc ccatcaaacg ctggtttcag aattataaat tacttggttg

gttaaataat cgtttttgat ttagacgcaa accggacgcg tagtttatta gaagatggta

taatttgcca actaagagat cagttaccag tagcagacct ttt----gtc ctttttttac

agtcttgcaa taaaactgat gcctgcattc aaatatccca acgaaatctt ttacaatctc

agttcacatt gtcacctaaa tttacgacgc cacagcaact cataaataat ttttaaagtt

aaaatgtaaa agtgcgttta attcaaaatt tgattcttat ttttctgcac taatttcgta

aacaattatt tgaagtgaat acgcgtggtt tagtataaaa ttcaaaattt aagttttcgg

tcaaaaatta c--tatttca attattatct tta------- ---------- ----------

---------- ---------- ---------- ---------- ---------- ----------

---------- ---------- ---------- -----

>0056_LG6

aaccttattg cgcacccaag aaaatggcga cttctaatga aataaaaatc ctgaaatttt

acacaccagc ctgtacattg ccaaggacca ttttcgattt ttattttttt gttaataaat

aaagaagcaa ttttgtgaga ttttaggcac taactgttaa tttaaaagtt tttagactaa

gtgaatcctt acctgctatc ctgtcgaacc ctggtatcag aattataatt tatttggttt

gttaaataat cgttttttat ttagacgcaa accagaggcg tgattcatta gaagatggta

taatttgcca acaaagggat cagttaccag tagccaatct tttcgaagcg ccttatttac

agtcttgcaa taaaactgat gcctgcattg aaatatccca acgaaatctt ttacaatctc

aattcccact gtcacataaa tttacgacgc cacagcaact cataaataat ttttaaagtt

aaaatataaa agtgggatta attcaaaatc tgattggtat ttt--tttgc taatttcgta

aacaattatc tgaaataaat acgtgtagtt tagtataaaa ttcagaattt gactttttgg

ccgaaaatta ctttttctca attattatct ttatctgtaa gtaaaaaatt accaaaaaat

taggagagac accttttgaa cgt--aatca cgcgtgtaaa atgca----- -----atttt

attagaaatc gcgattttct tagt------ -----

>0066_LGX

---------- ---------- ---------- ---------- ---------- ----------

---------a ctgtacattg ctaaggacca ttctcgattt ttgttttttt gttaataaat

aaaatagcag ttttgtgaga ttttgggcac taactgttaa ttta--agtt tttagactta

gtgaatcttc acctgctcgc ccatcgaacc ctgatatcag aattataaat tacttggttg

attaaatgat ggtttttcac ttagacgcaa accagacccg tgatttatta aaagatggta

taaattgcca acaaagggat cagttaccaa tagccgacct ttttgaagcc ccttatttac

agttttgcaa taaaactgat gtctgcaatc aaatatctca acaaaatctt ttacaatctc

agttcacatt gtcacctaaa tgtacaacgc cacagcaact cataaataat gtttaaagtt

aaaatgaaaa attgcgttta attcaaaatc tgattggtat ttttcttcgc taatttcgta

aacaattatc tgaagtgaat acgcttggtt tagtataaaa ttcaaaattt aacttcttgg

tcaaaaatta ctttttttta attattattt ttatctgtaa gtgaaaaatt accaaataat

taggggagat acctttcgaa cgtacagtca -gcgtgtaaa atgcaatatt ttttt-----

---------- ---------- ---------- -----

>0052_LG3

aaccttatcg cg-acccaag aaaatcgcga cttctaatga aataaaaatc ctgaaatttt

acacaccagc ctatacactg ataaggacca ttcacgattt ttattttttt gttaataaat

aaagtaccag ttttgtgagt ttttgggcac caactgttaa tttgaaagtt tttagaccaa

atgaatcctt acctgctagc ccattgaacg ctggtatcag aattataaat tgcttggtta

gtgaaataat cg----ttat ttagacgcaa actaggcgcg tgatttatta gaagatggta

taatttgcca acaaagggat cagttatcag tagccgacct ttttgaagcc ttttatttac

agtcttgcaa taaaactaat gcttgcattg aaatattcca acgaaatctt ttgcaatctc

agttcacatt gtcacctaaa tttacgacgt cacagcagct cataaataat ttttaaagtt

aaaatgtaaa agtgtgttta attcaaaatc tgattggtat ttttcttcgc taatttcgta

aacagttata tgaagtgaat acgcgtggtt tagtataaat tttagaattt gattttttgg

tcgaaaatta ctttttttaa attattattt ttatctgcaa gtgaaaaatt acgaaaaaat

taggagagat ---------- ---------- ---------- ---------- ----------

---------- ---------- ---------- -----

>0127_LG9

aaccttatcg agcaccaaag aaaatcgcga cttctaatga aataaaaatc ctgaaatttt

acacacaagt ctgtacattg ataaggacaa ttttcgattt ttgttttttt gttaataaat

aaagtagcag ttttgtgtgt ttttgggcat taactgtaat tttaaaagtt tgcagaccaa

ataaatcctt acctgctagc ccatcgaacg ctggtatcag aattataaat tacttggttg

gtgaaataat cgttttttat ttagacgcaa accagacgcg tgatttatta aaagatggta

caatttgcca acaaagagat cag------- ---------- ---------- --ttatttac

agatttgcaa taaaactgat gcctgcattg aaatatttca acgaaatctt taacaatctc

agttcacatt gtc------- -----gacgc cacagcaact cataaataat ttttaaagtt

aaaatgtaaa agtgcgttta attcaaaatc tgattggtat tttttttcgc taatttcgta

aacaattatc taaagtgaat acgcgtggtt tagtataaaa tttagaattt gactttttgg

tcgaaaatta ctttttttta attattattt ttatctgcta gtaaaaaatt accaaaaaat

taggggagat accattcga- ---------- ---------- ---------- ----------

---------- ---------- ---------- -----

>0055_LG2

aaccttatcg cgcacccaag aaaatcgcga cttctaatga aataaaaatc ctgaaatttt

acacactagc ctgtacattg atatgaacca ttctcgattt ttattttttt gttaataaat

aaagtagcag ttttgtgagt ttttgggcac taactgttaa tttaaaagtt tttagaccaa

atgaatcctt acctgctagc ccatcaaacg ctggtatcag aattataaat tacttggttg

gtgaaataat cgttttttat ttagacgcaa actagacgag tgatttatta gaagatggta

taatttgcca acaaaggaat cagttactag tagccgacct ctttgaagcc ctttatttac

agttttgcaa taaaactga- ---tgcattg aaatattcca acgaaatctt ttacaatctc

acttcacatt gtcacctaaa tttacgacac cac------- -------aat ttttaaagtt

aaaatgtaaa agtgcgttta attcaaaatc tgattggtat ttttcttcgc taatttcgta

aacaattatc tgaagtaaat acgcgtagtt tagtataaaa tttagaatta gattttttgg

ttgaaaatta cttttttt-t attattattt ttatctgcaa gtaaaaaatt accaaaaaat

ta-gaaagat acctttcaaa cgtacagtca ggcgtgtaaa ata------t tttttattta

aatagaagtc gcgattttct taggtgtgcg ataag

>0046_LG6

aaccttatcg cgcacccaag aaaatcgcga cttctaatgt aataaaaatc ttgaaatttt

acacaccagc ctgtacatta ataaggacca ttctcgattt ttattttttt gttaataaat

aaagtagcag ttttgtgagt ttttgggcac taactgttaa tttaaaagtt tttagactaa

ataaatcctt acctgctagc ccatcgaacg ctggtatcag aactataaat tacttggttg

gtttaataat cgttttttat ttagaggcaa accagacgcg tgatttatta gaagatggta

taatttgtca acaaagggat tagttaccag tagcctacct ttttaaagcc cgttatttac

agtcttac-- ---------- ---------- aaaaattcca acgaaatctt ttacaatctc

agtccacatt gtcacctaaa tttacgacgc cacagcaact cataaataat ttttaaagtt

aaaacgtaaa agtgcgttta attcaaaatc tgattggtat tttt-ttcgc taatttcgta

aacaactatc tgaagtgaat acgcgtggtt tagtgtaaaa ttcagaattt gacattttgg

tcggaaatta ctttttttta attatttttt ttatctgcaa gtgaaaaatt accaaaaaat

tagtggagat acctttcgga cgtagagtca cgtgtgtaac atgcaatatt tttttattta

ataagaaatc gcgattttct taggtgcgcg ataag

>0163_LG4

aatcttatcg cgcacccaag aaaatcgcga cttctaatga aataaaaatc ctgaaatttt

acacaccagc ctgtacattg ataaagacta ttctcgattt ttattttttt gttaataaat

aaagtagcag ttttgtgagt ttttggtcac taactgttaa tttgaaagtt tttacaccaa

atgaatcctt acctgctagc ccatcgaacg ctggtatc-- ---------- ----------

---------- ---------- ---------- ---------- ---------- ----------

---------- ---------- ---------- ---------- ---------- ----------

---------- ---------- ---------- ---------- ---------- ----------

---------- ---------- ---------- ---------- ---------- ----------

---------- agtgccttta attctaaatc tgattgatat ttttcttcgc taatttcgta

aacaatcatc tgaagtgaat acgcgtggtt tagtttaaaa tttagaattt gactttttgg

tcgaaaatta cttttttttt attattattt ttatctgcaa gtgaagaatt aacaaaaaat

taggaaagat accttccgaa cgtacagtca ggcgtgtaaa atgcaata-t tttttattta

attagaaatc gcgattttct tgggttcgcg ataag

>0110_LG3

aaccttatag cgcacctaag aaaatcgcga cttctaataa aataaaaata ctgaaatttt

acacaccagc ctgtacattg ataaggacca ttctccattt ttattttttt gttaataaat

aaagtagcag ttttatgagt ttttggacac taactgttaa tttaaaagtt tttagaccaa

atgaattctt acctgctggc ccatcgaacg ctggtatcag aattataaat tacttggttg

gtgaaataat cattttttat ttagacgcaa actagacgcg tgatttatta gaagatggta

taatttacca acaaagggat cagttaccag taa------- ---------- ----------

---------- ---------- ---------- ---------- ---------- ----------

---------- ---------- ---------- ---------- ---ataaaat ttttaaagtt

acaatgtaaa agagcgttta atttaaaatc tgattggtat ctttcttcgc taattttgta

aacaattatc tgaagtgaat acacgtggtt tagtataaaa tttagaattt gattttttgg

tcgaaaataa c-ttttttaa attattattt ttatctgcaa gtgaaaaatt accaaaaaat

tagtggagat acctttcgaa cgtacagtca agcttgtaaa atgcaatatt tttttattta

attagaaatc gtgattttcc taggtacgcg atatg

>0054_LG3

---------- -gcacccaag aaaatcgcga cttctaatga aataaaaatc ctgaaatttt

acacaccagc ctgtacattg ---aggacca ttctcaattt ttattttttt gttaataaat

aaagtagcag ttttgtgtgt ttttgggcac taactgttaa tttagaagtt tgtagtccaa

atgaatcctt acctcctagc ccatcga--- -----atcag aattaaaaat tacttggttg

gtgaaatatt tgttttttat ttagacgcaa accagatgcg tgatttatta gaagatggta

taatttgcca acaaagggat cagttaccag tagccgacct tt-------- -----tttac

agtcttgaaa taaaattgat gcctgcattg aaatattcca acgaaatctt ttacaatctc

agttcacatt gtcacctaaa cttacgacgc cacagcaact cataaataat ttttaaagtt

aaaatctaaa agtgcgttta attcaaaatc tgattggtat ttttcttcgc taatttcaca

aacaattatc tgaagtgaat acgcgtggtt tagtataaaa tttagaattt agc-ttttgg

tcgaaaatta cttttatc-c attattattt tcatctgcaa gtgaaaaatt accaaaaaat

taggggagat acctttcgaa cgtagagtcg ggcgtgtaaa atgcacta-t tttttattta

attagaaatc gcgattttct taggtgcgcg -----

>0048_LG8

---------- ---------- ---------- ---------- ---------- -------att

acacaccagc ctgtacattg ataaggacca tttccgattt ttattttttt gttaataaat

aaa--agcag ttttgtgagt ttttgggcat taactgttaa tttaaaagtt tttacaccaa

atgagtcctt acctgctagc ccatcgaaca ttggtatcag aattataaat tacttggttg

gtaaaataat cgttttttat ttagacgaaa accagacgcg tgatttttta gaagatggta

taatttgcca acaaagggat cagttaccag tagccgacct ttttgaagcc ccttatttac

agtcttgcaa taaaactgat acctgcattg aaatatccca acgaaatctt tttcaatctc

agttcacatt gtcacctaaa tttacgacgc cacagcaact cataaataat ttttaaagtt

aaaatctaaa aatgcgttta atttaaaatc tgagtggtat ttttcttcgc taatttcgta

atcaattatc tgaagt--at acgcgtggtt tagtataaaa tttagaattt gtctttttgg

tcgaaaatta cttttttt-- attattagtt ttatctacaa gtgaaaaatt accaaaaaat

taggggagat acctttcaaa cgtacagtca agcgtgtaaa atgcaataat tttttattta

attagaaatc gtgattttct tgggtgcgcg atatg

>0073_LG7

---------- ----cccaag aaaatcgcga cttctaatga aataaaaatc cttaaatttt

acacaccagt ctgtacattg ataaagaccg ttctcgattt ttattttttt gttactaaat

aaagtagcag t----tgagt ttttggggac taactgttaa tttaaaagtt tttag-----

---------- acctgctagc ccatcgaacg ctggtatcag aattataaat tacttggttg

gtgaaataat cgttttttat ttagacgcaa accagacgcg tgatttatta gaagatggta

taatttgcca acaaagggat cagttaccag tagccgacct ttttgaagcc ccttatttac

agtcttgcaa taaaactgat gcctgcattg aaatatccca acgaaatctt ttacaatctc

agt------- -tcacctaaa tttacgacgc cacagcaact cataaatagt ttttaaagtt

aaaatgtaaa agtgcgttta attcaaaatc tgattggtat ttttcttcgc taatttcgta

aacaatcatc tgaagt---- ---------- ---------- ----gaattt gactttttgg

tcgaaaattt cttttttttg attattattt tcatctgcaa gtgaaaaatt accaaaaaat

tagtggagat acctttcgaa cgtacagtca ggcgtgtaaa atgcaatatt tttttattta

attagaaatt ---------- ---------- -----

>0057_LG10

aaccttatcg cgcacccaag aaaattgcga cttctaatta aataaaaatt ctgaaatttt

acacaccagc ctatacattg atagggaccg ttctcgattt ttagtttttt gttaataaat

aaagtagcag ttttgtgagt ttgtgggcac taactgttaa tttcaaagtt tttagaccaa

a--------- ---tgctagc ccatcgaatg ctggtatcag aattataaat tacttagttg

gtgaaataat cgttttttat ttagacgcaa accagacccg tgatttatta gcagatagta

aaatttgcca acaaaggaat cagttaccag tagccgactt ttttgaaatc ccttatatac

agtcttgcaa taaaactgat gcctgcattg aaatatctca acgaaacctt ttacaatctc

agttcacatt gtcacccaaa tttatgacgc cactgcaact tataa--aat ttttaaaatt

aaaatgtaaa agtg-gatta attcaaattt tgattgatat ttt--tttgc taattttgtt

aacaattatc tagagtgaat acgtgtagtt tagtataaaa ttcagaattt gactttttgg

tcgaaaatta ctttttttta attattattt ttatctgtag gggacaaatt atcaaaaaat

tagtggagat atctttcgaa cgtacagtca ggcgtataat atgcaatatt tttttattta

attaaaaatc gcgattttct taggtgcgcg ataag

>0069_LG5

---------- ---------- ---------- ---------- ---------- ----------

---------- ---------- ---------- ---------- ---------- ---------t

aaagtagcag ttttgtgaat ttttgagcac tacctgttaa tttaaaaatt tttagaccaa

atgaatcctt acctgctaac tcatcgaacg ctggtatcag aattataaat tacttagttg

gtgaattaat cgttttttat ttagacgcaa act--acgca tgatttatta aaagatggta

taatttgcca acagagggat cagttaccag tagccgacct ttttgaagtc ccttatttac

actcttgcaa taaaactgat gcctgc-ttg aaatatccca acgaaatctt ttacaatctc

agttcacatt gtcacctaaa tttacgacgc cacagcaagt catacataat ttttaaagtt

aaaatgtaaa agtgcgttta a--------- ---------- --ttattcgc taatttcgta

aacaattatc tgaagtgaat acgcgtggtt tagtataaaa tttagaattt gattttttgg

acgaaaatta ctttttttta attattattt ttatttgcaa gtgaaaaatt accaaaaaat

tagggaagat acctttcga- ---acagtca ggcgtgtaaa atgcaatatt tttttattta

attagaaatc gcgattttct tagatgtgcg ataag

>0064_LG5

aaccttaccg cgcacccaag aaaatcgcga gttctaatga aataaaaatc ctgaaatttt

acacaccag- ---------- ---------- ---------- ---ttttttt gttaataaat

aaaatagcag ttttgtacgt ttttgggcac taactgttaa tttaaaagtc tttagtccaa

atgaatcctt acctgctaac ccatcgaacg ctggtatcag aattacaaat tacttggttg

gtgaaataat tgttttttat ttagacgcaa actagacgcg tgatttatta gaagatggta

taatttgcca acaaagggtt cagttaccag tagccgacct ttttgaagcc ccttatttac

agtcttgcaa taaaactgat gcctgcattg aaatatccca acgaaatctt ttacaatctc

agttcacatt gtcacctaaa tttacgacgc cacagcaact cataaataat ttttagtttt

ta-------- ---gctttta gttcaaaatc tgattagtat tttt-ttcgc taatttcgta

aacaattatc tgaagtgaat acgcgtggtt tagtataaaa ttgagaattt gattttttgg

tcaaaaatta ctttt-ttta attatta-tt ttatctgcaa aagaaaaatc accaaaaaat

tagtggagat acctttcaaa cgtacagtca ggcttgtaaa atgcaat-tt tttttattca

attagaaatc gcgattttct taggtgcccg ataaa

>0077_LG3

aaccttatcg cgcacccaaa aaaatcgcga cttctaataa aataaaaatc ctgaaatttc

acacaccagc ctgtacattg ataagaatca ttttcggatt ttattttttt tttaataaat

aaagtagcaa ttttgtgagt ttttgggcac taactgttaa tttaaaagtt tttagaccag

atgaatcctt acctgctagc ccatcgaacg ctgacatcag aattataaat tacttggttg

gtgaaacaat cgttttttat ttagacgcaa accagacgcg taatttatta gaagatggta

taatttgcca acaaagggat cagttaccag tagccgactt ttttgaagcc ccttatttac

agtcttgcaa taaaactgat gcctgcattg aaata--cca acgaaatctt ttacaatct-

---ttacatt gtcacctaaa tttacgacgc cacagcaact cataaataat ttttaatgtt

aaaatgcaa- ---------- ---------- ---------- ---------- ----------

---------- --aagtgaat acgcgtggtt tagtataaaa ttttgaattt gactttttgg

tcaaaaattt ctt------- ---------- ---------- ---------- ----------

---------- ---------- ---------- ---------- ---------- ----------

---------- ---------- ---------- -----

>0058_LG3

aaccttatcg cgcacccaag aaaatcgcaa cttttaatta aatgaaaatc ctgaaatttt

acacaccagc ctgtacattg ataaggaccg ttctcgattt ttattttttt attaataaat

aaagtagcag ttttgtgaga ttttgagcac taactgttaa ttta-cagtt tttagactaa

gtgcatcctt acctgctggc ccatcgaacc ctggtatcag aattataaat tacttgattg

gtgaaataat cgttttttat ttagacgcaa accagacgcg tgatttatta gaagatgata

taatttacca acaaaaagat cacttaccag tagccgacct ttttaaagcc ccttatttac

agtcttgcaa t--------- ---tgtactg aaatatccca actaaatcta ttacaatctc

agttcacatt gtcacctaaa tttacgacgc cacagcaact cataaataat gtttaaagtt

aaaatgtaaa agtgcgttt- attcaatatt tgattggtat tttt-tttgc taatttcgta

aacaattatc tgaagtaaat atgagtagtt tagtataaaa ttcagaattt cactttttgg

tcgaaaatta ctttt-tcca attattatct ttatctgtac gtaaaaaatt accaaaaaat

tatgggagat accttttgga agtacagtca ggcatgtaaa atgcaaatta tttttattta

actagaaatc gcaattttct taggtgcgcg ataag

>0061_LGX

aaccttatcg cgcacccaag aaaatcgcaa cttctaatga aataaaaaac ctgaaatttt

acacaccagc ctgtacgttg ataagggcca ttttcaattc ttattttttt gttaataaat

aaagtagcag ttttgtgagt ttttg-gcac taactgttaa tttgaaagtt tttaaactaa

atgaataata acctgctaac ccatcaaagg ctggtttcag aattataaat tacttgtttg

gttaaatatt cgttttttat ttagacgcaa accagacgcg tgatttatta aaaggtggta

taaattgcca acaaagggat caattaccaa tagccgacct ttttgaagcc ccttatttac

agttttgcaa taaaactgat gcctgcattt aaatatccca acgcaatctt ttacagtctc

agttgacttt atcacctaaa tttacgacgc cacagcaac- --taaataat ttttgatgtt

aaaatgt--- ---------- ---caaaata tgattggtat ttttctccgc taatttcgta

aacaattatc tgaagtaaat acgcgtggtt tagtttaaaa ttcagaattt gacttttcgg

tcgaaaatta ctttgtttta attattatct ttatctgtaa gtgaaaaatt atcaaaaaat

taaggaagat acctttcgaa cgtacagtta ggtgtgtaaa atgcaatatt tttttatttc

attagaaatc gcggttttct taggtgcgcg ataag

>0059_LG8

aaccttatcg cgcacctaag aaaatcgtga cttctaatga aataaaaatc ctgaaatttt

actcaccagc ctgtacattg ataaagacca ttatcgattt ttattttttt gttaataaat

gaagtagcag ttttgtgagt ttgtgggcac taactgttaa tttaaaattt ttttgaccaa

a--------- ---tgctagc ccatcgaacg ctggtataag aattataaat tacttggttg

gtaaaataat cgttttttat t--------- --tagacgcg tgatttatta gaagatggta

tagtttgcca acaaagggat cagttaccag tagccgacct ttttgaagcc cctta-ttac

agtcttgcaa taaaactgat gtctgcattg aaatatccca acgaaatttt ttacaatct-

--tttacatt gtcacctaaa tttacgacgc cacagcaact aataaataat tttta-----

aaaatgtaaa agtgcgttta attcaaaatc tggt-----a tttttttcgc taatttcata

aacaattatc tgaagt--at acgcgtggtt taatataaaa tttagaattt gtttttttgg

tcgaaaatta ctttttttta attattattt ttatctgcaa gtgaaaaatt accaaaaaat

taggagagat atctttcgaa cgtacagtca ggcgtgtaaa atgcaacatt tttttattta

attaaaaatc gcgattttct taggtgcgcg a----

>0123_LG3

---------- ---------- ---------- ---------- ---------- ----------

---------- ---------- ---------- ---------- ---------- ----------

---------- ---------- ---------- ---------- -----gagtt tttggactaa

atgaatcctt acttgctagc ccatcgaacc ctggtatcat aattataaat tacttggttg

gttaaataat cgttt----t ttagacgcaa actaaacgtg tgatttatta gaagatggta

taatttgcca acaaagggat cagttaccag tagccgacct ttttcaagcc ccttatttac

agtcttgcaa taaaactgat gcctgcattg aaatatccca acgaaatctt ttacaatctc

agttcacatt gtcacctaaa tttgcgacgc cacagcaact cataaattat ttttaaaatt

aaaatgtaaa agtgcgctt- ---------- ---------- ---------- ----------

--------tc gtaagtgaat acccgtggtt tagtataaaa ttcagaattt gattttttgg

tctaagacta cttttttaaa attattatct ttaactgtaa gtaaaaaatt accaaaaaat

taggggagat acctttcgaa agtacaatca agcgtgtaaa atgcggtatt tttttattta

attagaaatc gcgattttct taggtgcgcg ataag

>0131_LG8

---------- ---------- ---------- ---------- ---------- ----------

---------- ---------- ---------- ---------- ---------- ----------

---------- ---------- ---------- ---------- ---------- ----------

---------- ---------- ---------- ---------c aattataaat tatttagttg

gttaaataat cgttttttat ttaaacgcaa accagacgtg tgattcatta caagatggta

taatttgcca acaaagggat cacttaccag tagccgacct ttttgtagcc ccttatttac

agtaattcca taaaactgat gcctgtattc aaatatccc- acgaaatctt ttacaatttc

agttcgcatt gtcaattaca tttacgacgc cacagcagct cataaataat ttttaaagtt

aaaatgtata agtgcgttta attcaaaatc tgattggaat tttt-ttcgt taatttcgta

aacaattatc tgaagtgaat acgcgtagtt tagtataaaa ttcagaattt ----------

---------- ---------- ---------- ---------- ---------- ----------

---------- ---------- ---------- ---------- ---------- ----------

---------- ---------- ---------- -----

>0071_LG3

aaccttatcg cgcacccaag aaaatcactt ct-------- -----gaacc ccgaaatttt

acacaccagc ctgtacattg ataaggacca ttctcgatta ttattttttt gttaagaaat

aaa------- ----gtgaga ttttgggcat ttactgttaa tttaaaaatt tttagactaa

gtgaatcttt acctgctagc ccatggaacc atagtatcag aattataaat tacttgattt

gttaaataat cgttttctat ttagacgcaa accagacgct tgatttatta gaagatggta

aaatttgcta acaaagggat cacttaccaa ttgccgactt ttttgaagct ctttatttac

agttttgcaa taaaactgat gcttgcattg aaatatccca acgaaatctt ttacaatctc

agttcacatt gtcacctaaa tttacgacgc cacagtcact cataaataat ttttaaagtt

aaaacgtaaa agtgcattta atttaaaatc tgatgggtat ttt--tttgc taatttcgta

aac-cttatc tgaagtgaat acgcatagtt tagtataaaa ttcagaattt gactttttgg

tcgaaaattc ctttttttca attatta-cc ttatctgtaa gtaaaaaatt accaaaaaat

tagggaaaat actttttgaa cgtacagtca agcgtgtata atgca--att tttttattta

at-agaaatt gcgattttct taggtgcgcg ataag

>0106_LG3

---------- ---------- ---------- ---------- ---------- ----------

---------- ---------- ---------- ---------- ---------- ----------

---------- ---------- ---------- ---------- ---------- ----------

---------- ---------- ---------- ---------- ---------- ----------

---------- ---------- ---------- ---------- ---------a gaagatggta

tagttcacca acaaagggct cacttaccag tagccaacct ttttgaagcc ccttatttac

agttttgcaa taaaactgat gc---cattg caatatccca acggaatctt ttacaatctc

agttcacaat gtcacctaaa tttacgacgc cacagcaagt cataaacaat ttttaaagtt

aaaatgtaaa agtgcgttta attca--atc tgattggtat ttt--ttcgc taatttcgta

aacaattatc tgaagtgaat actcgtactt tagtataaaa ttcagaattt gactttttgg

tcgaaaatta ctttt-ttga attattatct ttatatgtaa gtgaacaatt atcaaaaaaa

tagggaacat acctttcgaa cgtacggtca ggcgtgtaaa atgca--att tttttattta

attagaaatc gcgattttct taggtgagct ataag

>0094_LG7

aaccttatcg cgcacccaaa aaaatcgcga -ttctaatga aataaaaatc ctgaaatttt

ttacaccagc ctgtagaatg ataaggaccg tttttggttt ttaatttttt gttaataaat

aaagtagcag ttttataagt ttttgggcac tgactgttaa tttaaaagtt tttagactaa

gtgaatcctt gcctgctagc tcatcaaaca ctggtatcag aattataaat tacttggtta

cttaaataat cgttttgtat ttaaacgcga aacagacgca tgatttatta gacgttagta

taatttgcca acaaagagat cacttaccag tagccgacct ttttgaagtc ccttatttac

agtcttacaa taaaactgat gcctgcactg aaatatccca ac-aaacctt ttacaatctc

agttcacatt gtcacc-aaa tttacgacgt cacagcaact cataaataat ttttaaactt

aaaatgtaaa attgcgttta attcaacatc tg-------- ---------- ----------

---------- ---------- ---------- ---------- ---------- ----------

---------- ---------- ---------- ---------- ---------- ----------

---------- ---------- ---------- ---------- ---------- ----------

---------- ---------- ---------- -----

>0092_LG10

aaccttatcg cgcacccaag aaaatcgcga cttctaatga aatga----- ---aaatttt

acacaccagc ctgtacattg ataa--acca ttctcgattt ttattttttt gttaataaat

aaagtagcag ttttgtgaga ctttgggcgc taactgttaa tttaaaagtt tttagactaa

gtgattcctt acgtgctagc ccattgaacc ctggtatcag aattataaat tacttggttt

gttaaattat cgttttttgt tcagacgcaa accagacgcc tgatttatta gaatatagta

taatttgcca acaaagggat cacttacctg tagccgatct ttttgaagcc ccttatttac

tgttttgcaa taaaactgat gcttgcattg aaatatccca acgaaatctg ttacaatctc

agttcacatt gctaccgaaa tttacgacgc cacagcaact cataaataat ttttaaagtt

aaaatgtaaa agtgcgttta attcaaaatc tgaa------ ---------- ----------

---------- ---------- ---------- ---------- ---------- ----------

---------- ---------- ---------- ---------- ---------- ----------

---------- ---------- ---------- ---------- ---------- ----------

---------- ---------- ---------- -----

>0097_LG5

---------- ---------- -------cga cttctaatga aatgaaaaac ttgaaatttt

acacaccagc ttgtacactg ataaggacca ttctcgattt ttactttttt gttaataaat

aaagtagcag ttttgtgaga ttttgggcac taactgttaa tttaaaaatt tctagactga

gtgcatcctc agctgttagc tcatcgaacc ctggtatcag aattataagt tacttggttt

gttaaataat cgttttttat ttagatgcaa accagacgcg tgatttatta gaagatagta

taatttacca acaaagggat cacttactag tagctgacct ttttgaagcc ccttatttac

agttttgcaa taaaactgat gcctgcattg aaatatccca acgaaatctt ttacaatctc

agttcacatt ctcacctaaa tttacaacgc cacagcagct cagaagtaat ttttagagtt

gaaatgtaaa agtacgttta attcaacgtt g-------gt atttttttgc taatttcgta

aacaattatc tgaagt---- ---------- ---------- ---------- ----------

---------- ---------- ---------- ---------- ---------- ----------

---------- ---c------ ---------- ---------- ---------- ----------

---------- ---------- ---------- -----

>0067_LG4

---------- -------aag aaaatcgcga cttctaataa aataaaaatc ctgaaatttt

acacaccagc ttgtacaatg attaggacca ttctcggttt ttattttttt gttaataaat

aaagtagcag ttttgtgagt ttttgggcac taactgttat tttaaaagtt tttagactaa

gtgaattctt acccgctagc ccatcgaacc ctggtatcag aattataaat tacttggttg

gttaaataat cgttttatgt ttagacgcaa actagacgcg tgatttatta gaagatggta

tagtttgcca acaaagggat cagttactag tagccgacct ttttgaagcc ccttatttac

agtcttgcaa taaaactgct acctgctttc aaatatcaca acgaaatctt ttacaatctc

agttcacatt gtcacctaaa tttacgacgc cacagcagct cataaataat ttttgaagtt

aaaacttaaa agtgcgttta attcaaaatc tgat---gtt tttttttcac taattttgta

atcaattat- ----gtgaat acacgtagtt tcgtataaaa ttcagaattt gaatttt-gg

tcaacaat-- --tttttttt attcttatct ttatttgtaa gtgaataatt accagaaaat

taggggacat tcctttcgaa gatacagtca ggcctgtata aagcaa-aaa aaattattta

ataagaaatc gcgattttct tagttacgcg aaaag

>0141_LG2

---------- ---------- ---------- ---------- ---------- ----------

---------- ---------- ---------- ---------- ---------- -------tat

aaagtagcag ttttatgagt ttttgggcac taactg---- tttaaaagat tttagaccaa

atgaatcctt acctgctagc ccatagaacg ctggtttcag aattataaat ttcttgattg

gtgaaataat cgttatttca ttagacgcaa accagacgcg tgatttatta gaaggtggta

taatttgcca ccaaagg--- ---------- ---------- ---------- ----------

-gtcttgcaa taaaactgat gcctgcattg aaatatccaa acgaaatctt ttacaatctc

agttcacatt gtcacctaaa tttacgacgc cacagcaact aataaataat ttttaaagtt

aaaatgtaaa agtgcgttta attcaaaatc tgattggtat ttttcttcgc taatttcgta

aacaattatc tgaagtgaat acgcgtgatt tgatataaaa tttagaattt gattttttgg

tcaaaaatta ctt------- -------ttt ttatctgcaa gtgaaaaatt accaaaaaat

taggggagat acctttcaaa cgtacactc- ---------- ---------- ----------

---------- ---------- ---------- -----

>0103_LG4

---------- ---------- ---------- ---------- ---------- ----------

---------- ---------- ---------- ---------- ---------- ----------

---------- ---------- ---------- ---------- ------agtt tttagacaaa

atgaatcctt acctgctagc ccatcgaacg ctggtatcag aatgataaat tat-tggttg

gttaaatagt tgttttc--- ---------- ---------- ---------- --------tt

taatttgcca acaaagggat cagttaccag tagccgacct tttcaaagcc ccttatttac

agttttgtaa taaaactgat gcctgcattg aaatatccca acgaaatctt ttacaatctc

agttcacatt gtggctttaa tttaagacgc cacaggaact cataaataat ttttaaagtt

aaaatgtaaa agtgcgttta attcaaaatc tgat-----t ttttttttgc taatttcgta

aacaattatt tgaagtgaat acgtgtagtt tagtataaaa ttcagaattt aactttttgg

tcgagcttta ctttt-ttca attattattt ttatctataa ttaaaagatt accaaaaaat

taggggagat accttttgaa cgtacggtca ggcgtgtgaa atgca--att tttttatgta

attagaaatc cctattttct taggttcgcg ataag

>0158_LG4

aaccttatcg cgcacccaag aaaatcgtga cttctaatga aataaaaatc ctgaaatttt

acacaccagc ctgtacattg ataaggacca ttctcgattt ttaaaaaatt gttaataaat

aaagtagcag ttttgtgaga ttttgtgcac taactgttaa tttaaaagtt tttagaccaa

atgaatcctt acctgctagc tcatcgaacg ctggtatcag aattataaat tacttggtt-

---------- ---------- ---------- ---------- ---------- ----------

---------- ---------- ---------- ---------- ---------- ----------

---------- ---------- ---------- ---------- ---------- ----------

---------- ---------- ---------- ---------- ---------- ----------

---------- -gtgcgttta attcaaaacc tgattggtat ttttcttcgc taatttcgta

aacaattatc tgaagtgaat actcgtggtt tagtataaaa tttagaattt gattttttgg

tcgaaaatta c--------- -------ttt ttatctgcaa gtgaaaaatt accaaaaaat

taggggagat accattcgaa cgtacagtca ggcttgtaaa atgcaatatt tttttattta

attagaaatc gcgattttct caggc----g ataag

>0100_LG4

aaccttatcg cgcacccaag aaaagcgtga cttctaatga aataaaaatt atgaaatttt

acacaccagc ctgaacattg ctaaggacca ttctcgattt ttattttttt gttaataaat

aaagtagcag ttttgtgagt ttttgggcac taactgttaa tttaaaagtt tttagaccaa

atgaatcctt acctgctagc ctatcgaact ctgatatcaa aattataaat tacttggttg

gtaaa----- ---------- ---------- ---------- ---------- -aagatggta

taatttgcca acaaagggat cagttaccag tagctgacct ttttgaagcc ccttatttac

agtcttgcaa taaaactgat gccagcattg aaatatccca acaaaatctt tta------c

agttcacatt gtgacttaaa tttacgtcgc cacagcaact cataaataat ttttaaagtt

aaaatgtaaa agtgcgttta attcaaaaac tgaatggtat ttttcttcgc taatttcgta

aacaattatc tgaagtgaat acgcgtggtt tagtataaaa ttttgaattt gac-------

tcgaaaatta cttttttt-- --aattattt ttatctgcaa atgaaaaatt accaaaaaat

ta-gggagat acctttcgga ggtacagtca ggcgtgtaaa atgcaatatt tttttattta

attagaaact gcgattttct taggtgcgcg ataag

>0109_LG3

aaccttatcg cgaacccaag aaaatcgcga cttctaatga aaaaaaaatc ctgaaagttt

acacaccagc ctgtacattg ataagaacca ttctcgattt ttattttttt gttaataaat

aaagttgcag ttttgtgagt ttttgggcac taactgttaa tttgaaagtt tttaaaccaa

atgaatcctc acctgctagc cc-------- ---------- ---------- ----------

--------at cgttttttat ttagacctaa accagacgcg tgatttatta gaaaatgcta

taatttgcca acaaagggat cagttaccag tagccgacct ttttgaagcc ccttatttac

acccttgcaa tatattttat gcctgcattg aaatatccaa acgaaatctt ttacaatc-c

agtttatttt gtcaccccca cccacaacgc tacagcaact aatagataat ttttaaagtt

aaaatgtaaa agtgcgttta attcaaaata tga----tat tttacttcgc taatttcgta

aacaattatc tgaagtgaat acgcctggtt tagtataaaa ttaagaattt gattttttgg

tcgaaaatta ctttttttta attattattt ttatctgcaa gtgaaaaatt accaaaaaat

tagaagagat accttttgaa cgtacagcca a--------- -------tat tttttattta

attagaaatc gcgattttct taggtgcgcg ataag

>0129_LG7

-----tatcg c--------- ---------a cttctaatga aataaatatc ctgaaatttt

acacaccagc ctatacattg ataaggacca ttttaaattt ttattttttt gtaaataaat

aaagtagcag ttttgtgagt ttttaggcac caactgttaa tttaaaagtt tttagactaa

atgaatcctt acctgctagc ccatcgtacg ctggtatcag aattataaag tacttggttg

gtgaaataat cgtttttga- ---------- ---------- ---------- ----------

---------- ---------- ---------- ---------- ---------- ----------

---------- -------aac tccttattta taaaaaccca acgaaatctt ttacaatctc

agttcacact gtcacctaaa tttacgacac cacagcaact cataaataat ttttaaagtt

aaaatgtaaa agtgcgttta atttaaagtc tgattggtat ttttgttcgt taatttcgta

aacaattatc tgaagtgaac acgcgtggtt tagtataaaa tttaaaattt gagtttttga

tcgaaaatta ctttttttta attattattt ttatctgcaa gtaaaaaatt accaaaaaat

taggggagat accttttgaa cgtacagtca ggtgtgt--- ---------- ---ttattta

attagaaatt gcgattttct taggtgcgcg ataag

>0072_LG9

---------- ---------- ---------- --gctaatga aatgaaaatc ctgaaatttt

acacaccagc ctgtacattg ataagaactg ttctcgattt ttattttttt gttaataaat

aaagtagcag ttttgtgaga ttttgggcac taactgttaa cttaaaagtt tttagactaa

gtgattccat acctgttagc ccatcaaacc ctggtatcag aattgtaaat cacttggttt

gctaaataat cgttttttat ttagacgcaa accagatgcg tgatttatta gaagatggta

caatttgcca acaaaaggat cacttatcag tagccgacct ttttgaagtc -----tttac

aatcttgcaa taaaactgat gcctgcattg aaatatccca acgaaatctt acccca---c

agttcacatt gtcacctaaa tttacgacgc cacagcaatt cataaatgat ttttaaagtt

aaaatgtaaa agtgcgttta attcaaaa-- ---------- atttcttcac taatttcgta

aacaattacc tgaagtgaat acgcgtgatt tagtataaaa ttcagaattt gactatttgg

tcgaaaatta cttttctt-a attat--ttt ttttctgtaa gt-aaaaatt accaaaaaat

tatgggaaat accttttgga cgtacagtaa ggcgtgtaaa atgca--ttt tttttattta

attagaaatc gcgtttttct taagtgcg-- -----

>0075_LG5

---------- ---------- ---------- ------atgc aatgaaaatc ctgaaatttt

acacaccagc ctgtacattg aaaaggacca ttctggattt ttattttttt gttaataaat

aaagtagcag ttttgtgaaa ttttgtgcaa taattgttaa tttaaaagtt tttagactaa

ctgaatccct acctgttagc tcatcgaacg ctggtatcag aattattagt tactttgttt

gttaaataat cg-tttttat ttagacgcaa accagacacg taatttatta gaagatggta

taattttccc agaaaaagat catttacca- ---------- ----gaagcc ccttatttac

agtcttgcaa taaaactgat gcctg-attg aagtatccta acgaaatctt ttacaatctc

agttcacatt gtcacctaaa tttacgacac cacagcaact cataaataat ttttaaagtt

aaaatataaa agtgcgttta attcaaaatc tgatctgtat ttt--tttgc taattttgta

aacaattatc tgaagtaaat atacgtagtt tagtataaaa ttcagaattt aactttttag

tcgaaaatta ctttttttca attaatattt ttatctgcga gtaaaaaatt ataaaaaaat

taaaggagat atcttttgaa cgtgtagtca ggcaaa-aaa atgcaatttt tttttattta

ttttattttc gcgagtttcc taggtgcgcg ataag

>0088_LG2

---------- ---------- ---------- ---------- ---------- ----------

---------- ---------- ---------- ---------- ---ttttttt gttaattaat

aaagt----g ttttgtaagt ttttggacat taactgttac ttta---gtt tttacactga

gtgaatcctt acctgctagc ccatcgaacc ctagtatcaa aattatgaat tacttggttg

gtttaataat cgttttttat ttagacgcaa accagacgcg tgatttatta gaagatggta

taatttgccc acagagggct cacttaccag tagcagacct ttttgaagcc ccttaatgat

aat------- --caactgat gcctgcattc aaatatccca acgaaatctt ttacaatctc

aattcacatt gtcacctaaa tttacgatgc catagcaact cataaataat ttttaaagtt

aaaatgtaaa agtgcgttta attcaaaatc t--------- --------gc taattgcgta

aacaattttc tgaagtgaat acgcgtagtt tggtataaaa ttcagaattc gactttttgg

tcgaaaatta ctttt-ttta attattatct ttatctgtga gtgaaaaatt accaaaaaat

tagtggagat acattttgaa tgtatagcca ggcgtgtaaa attaa--ttt ttgttattta

attagaaatc gcgattttct taagtgcgcg ataag

>0090_LG3

---------- ---------- ---------- ---------- ---------- ----------

---------- ---------- ---------- ---------- ---------- ----------

---------- ---------- ---------- ---------- ---------- ----------

---------- ---------- ---------- ---------- ---------t tacttggttg

gtgaaataat cgttttt--- ---------- attagacgcg tgatttatta gaagatggta

taatttgcca ac-aagggtt cagttaccag aagccgacc- ttttgaagcg ccttatttac

agtcttgcaa taaaactgat gcgtgcattc aaatatcgca aggaattttt ttacaatctc

agttcacatt gtcacctaaa tttacaacgc aatagcaact cataaataat tttttaagtt

aaaatgtaaa agtgcgtttc attcaaaatc tgattggtat ttttcttcgc taatttccta

aacaattatc tcaagtgaat acgcgtcgtt tagtatgaaa tttagaattt gactttttag

tcgaaaatta ctttttttta attattattt ttatcagcaa gtgagaaatt accaaaaaat

tagggaagat acctttcgaa cgaacagtca ggcgtgcaaa atgcagtatt tttttattta

attagaaatt gcgattttct taggtgcgtg ataag

>0091_LG3

--tcttatcg cgcacccaag aaaatcgcga attttaataa aataaaaatc ctgaattttt

acacaccagc ttgtacaatg ataaggacca ttctcgattt ttattttttt gttaataaat

aaagtagcag ttttgtgaga ttttgggtac taactgttaa cttaaaagtt tgtagactaa

gtaaattttt acttgctagc ccattgaacc caggtatcaa aattctaaat aaat------

---------- --ttttttat ttatacgcaa accagacgcg tgatttatta gaagatgata

taatttgcca acaaaggaat cacttaccag tagccgacct ttttgaagcc ccttatttac

agtcttacaa taaatctgtt gcctgaattt aaaaatctca acgaaatctt ttacaatctc

agttcacatt gtcacctaaa tttac---gt gacaacaatt cagaaataat ttttaaagtt

aaaatgtaaa agtgca---- ---------- --------gt atttttttga taatttcgta

aacaatttta tgaagtgaat acgcgtattt tagtataaag ttcagaattt gactttttgg

tcgaaaatta cttttt---- ttaattatct ttatctacaa gtaaaaaatt accaaaaaat

taggaaagat accttttgaa cgtacagtca ggcgtgtaat atgc---att tttttattta

attagaaatt gctattttct tagatgcgcg ataag

>0115_LG9

aaccttatcg cacacccaag aaaatcgcta cttctaatga aatgagaatc ctgaaatttt

acacacccgc ttgtatattg ataaggatca ttctcgattt tttatttttg gt-----aat

aaagtaaccg ttttgtgagt ttttgggcac taactgttaa tttaaaagtt ttgagactaa

gtgtattctt acctgctagt ccatcgaacc ctggtatcag aattataaac tacttggttg

attaaataat cg-------- ---------- ---------- ---------- ----------

---------- --aaagggat cacttaccat tagccgatct ttttgatgta ccttatttac

agtcttgcaa taaaattgat gtctgcattc aaatacgcca acgaaatctt ttacaatccc

agttcacatt gtcacctaaa tttacgacgc cacagcagct cataaatgat ttttaaagtt

aaagtgtaaa agtgcgttta attcaaaatc tgattggtat tttt-ttcgc taatttcgta

aacaattatc tgaagtgaat atgcgtggtt aagaataaac ttcagaatct aactttttgg

tcgaaaattg ctttttgtta attattatct ttatctgtaa atgaaaaatt accaaaaaat

taggggagat gcctttcgag agtacagtca gacgtgtaaa atgc---att tttttattta

actagaaatc acgattttct taggtgcgcg ataag

>0180_LG9

---cttatgg cgcacccaag aaaatcgcca cttctaatga aatgaaaatc ctgacatttt

acacaccagc cagtacaatc ataaggacca ttctcgattt tta-tttttt tttaataaat

aatgtagcag ttttgtgagt ttttgggcac taacagttaa tttgaaagtt ttgagactaa

gtgaatcctt acctgctagc ctatcgactc ctggtgtcag aaatataaat tacttggttt

gttaaat--- ---------- ---------- ---------- -aaattatta aaagatggta

taatttgcca acaaagggat caattaccag tagccaacct ttttgaagct gtttatttac

agtcttgcaa taaaactgat gcctgcattt aaatatccta acgaaatctt ttacagtctc

agttcacatt gttacataaa tttgcgaaac cacaccaacc cataaataa- -tttaaagtt

aaaatgtaaa agtgcgttta attcaaaatc tgat------ ---------- ----------

---------- ---------- ---------- ---------- ---------- ----------

---------- ---------- ---------- ---------- ---------- ----------

---------- ---------- ---------- ---------- ---------- ----------

---------- ---------- ---------- -----

>0185_LG4

aaccatatcg cccacccaag aaaatcgcga cttctaatga aataaaaatc ctgaaagttt

acacaacaac ctatacactg acaaggaccg ttctcgattt ttgatttttt gtttttaaat

aaagtagcag ttttgtgagt ttttgggcac taactattaa tttaaaagtt tttagacaaa

gtgaa----- ---tgtttgc ccatcgaact ctggtatcag aattataaat ctcttggttg

gttaaaaaa- ---------- ---------- ---------- ---------- ----------

-taattgcca acaaagggat cacctaccag tagccgatct ttttgaagcc ctttatttac

aaccttgcaa taaaactgat gcctacattc aaatatccta acgaaatatt ttacaatctc

agttcacatt gtcacctaaa tttacgacac cacaca---- ---------- ----------

---------- ---------- ---------- ---------- ---------- ----------

---------- ---------- ---------- ---------- ---------- ----------

---------- ---------- ---------- ---------- ---------- ----------

---------- ---------- ---------- ---------- ---------- ----------

---------- ---------- ---------- -----

>0098_LG9

---------- ---------- ---------- ---------- ---------- ----------

---------- --------ag ataaggacca ttctcgattt ttgtttgttt attaataaat

aaagtagcag ttttgtgaga ttttcggcac taactgttaa -ttaacagtt tttagacaaa

gtgaatcctc acctgctagc ccatcgaacc caagtatcag aactataaat tacttggttt

gttaaataat cgttttttat ttatacgcaa accagacgcg tgatttatta gaagatggta

taatttgcca acaaagggat cacgagccag tagccgagct ttttgaagcc ccttatttac

agtcttgcaa taaaactgat gcttgcattg aaatatccca acgaaatctt ttacaatctc

agttcacatt ggcacctata ---------- ---------- ---aaatcat tttccaaatt

aaaatgtaaa agtgccttta attcaaaatt tgactgctat tttt-ttagc taattttgta

aacaattatc cgaagtgaat acgcgtagtt tagtataaaa ttcagaactt gactctttgg

tcgaaaatta cttttt---- ---------t caattaaaaa ttaaaaaatt accaaaaaat

taggggagat accttttgaa tgtacagtca ggcgtgtaaa atgc---att tttttattta

attagaaatc gcgattttct tgg------- -----

>0143_LG8

aaccttatcg cgcacccaag aaaatcgcga cttctaatga aataaaaatc ctgaattttt

acacaccagc ctgtacattg ataaggacca ttctcgattt ttattttttt gttaataaat

aaataaa-at aaatgtgtgt ttttgggcac taactgttaa ttaaaaagtt tttagaccaa

ataaatcctt acctgctagc ccatcgaacg ctagtatcag aattataaat taattggttg

gttaaataat cgtttttcat ttagacgcaa accagacgcg tgatttatta ggagatggta

taatttgcca at-------- ---------- ---------- cattgaagcg ccttatttac

agtcttgcaa ta-------- ---tgcttgc aaatatccca atgaaatgtt ttaca-----

---------- ---------- ---------- -----taaaa cataaataat ttttaaaatt

aaaatgtaaa agtgcgttta attcaaaatc tgattggtac ttttcttcgc taatttcgca

aataattatc tgaagtgaat acgcgtagtt tagtaaaaca ttcagaattt cactttttg-

---aaaaata ttttttttta attattattt ttatctgtaa gtcaaaaatt accaaaaaat

tttggaagat acctttggaa cgtacagtca agagtgtaca atgcaatatt tttttattca

attagaaatc gcgattttct taggtgcgcg ataag

>0135_LG9

aaccttatcg cgcacccaag aaaatcgcga cttctaatga aataaaaata ctgaaatttt

actcaccagt ctgtacaatg ataaggacca ttctcgattt ttaaaaaatt gtaaataaat

aaagtagcag ttttgtgagt ttttgggtac taacggttaa tttaaaagtt tttagaccaa

atgaatcctt acctgctagc ccatcgaacg ctggtatcag aattataaat tacttggttg
[truncated: 351,851 more chars]
