# Supplementary figures and images for: Dispersion Profiles and Gene Associations of Repetitive DNAs in the Euchromatin of the Beetle Tribolium castaneum
Source: G3 (Bethesda). 2018 Jan 8;8(3):875–86. doi: 10.1534/g3.117.300267 (PMC5844308; doi:10.1534/g3.117.300267)

## A Tcast3

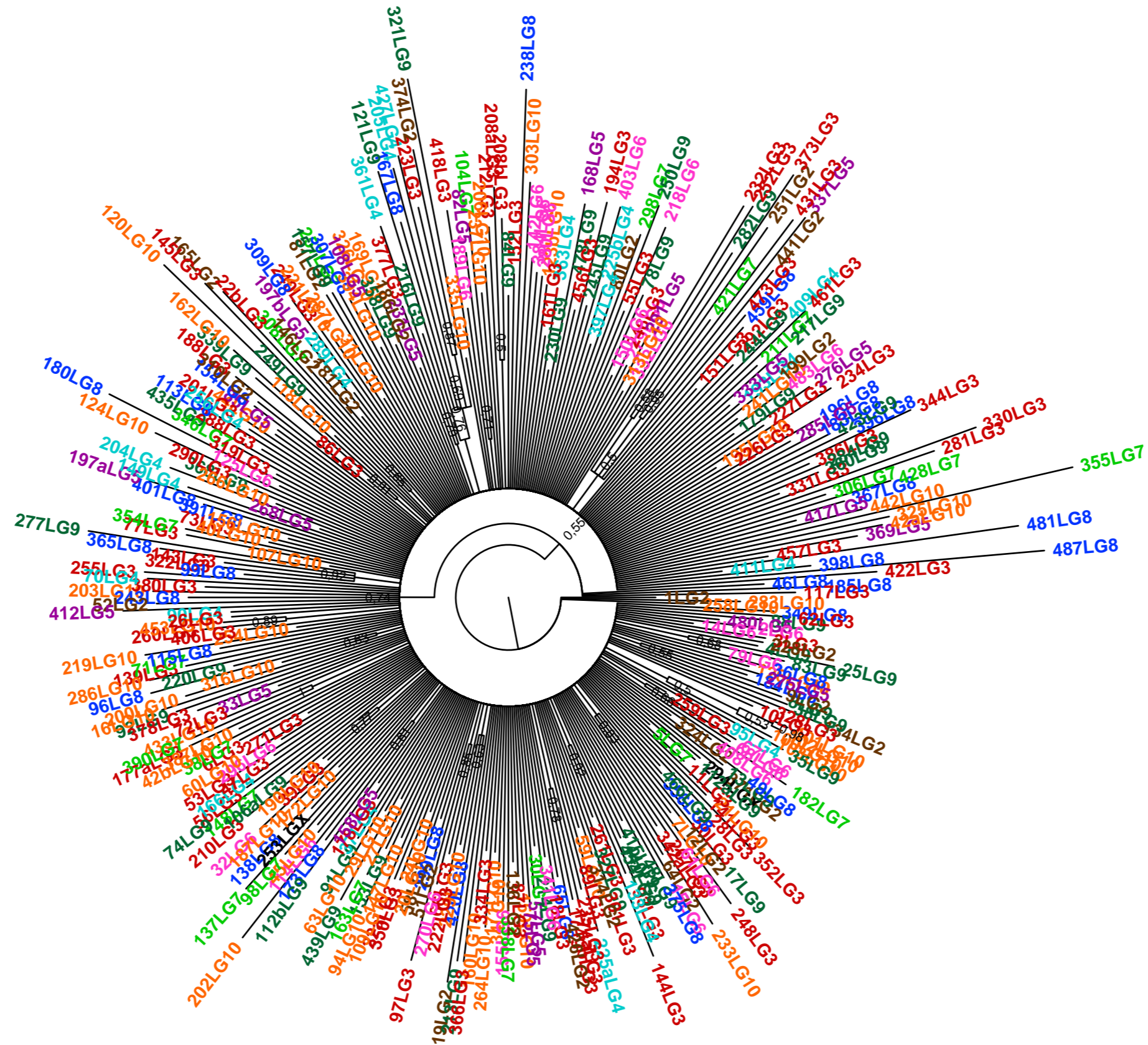

0.03

B Tcast4

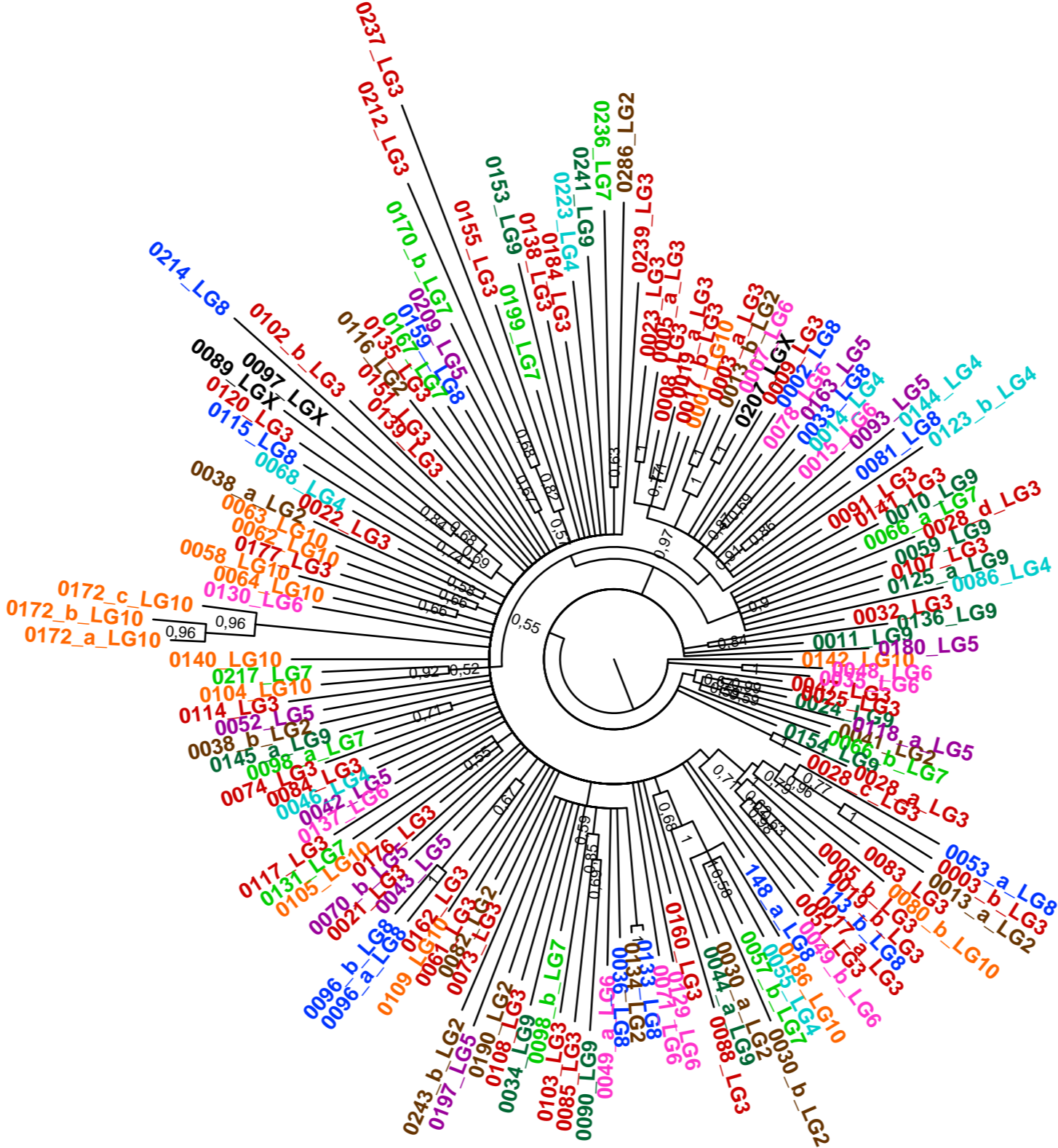

## C Tcast5

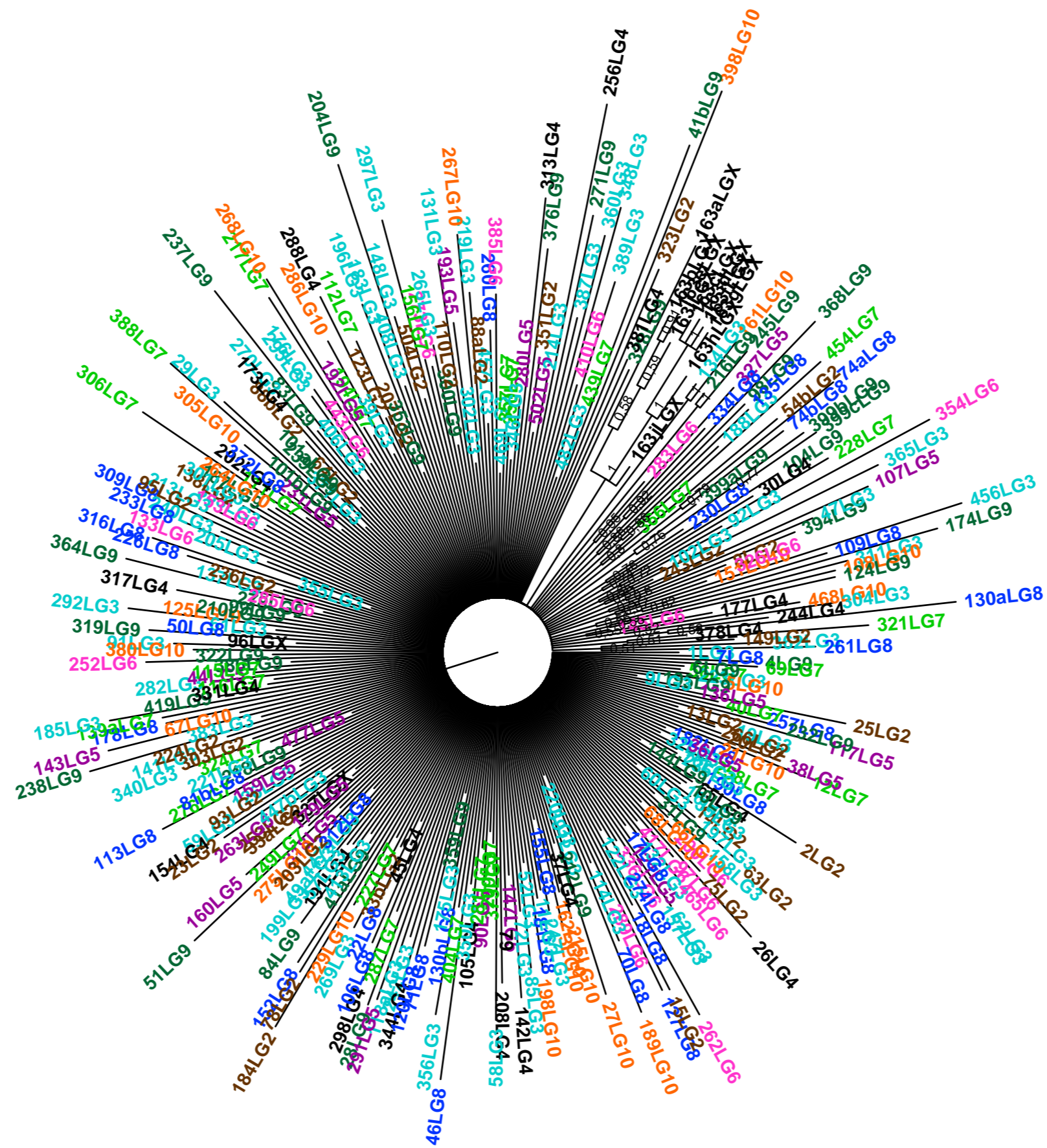

0.04

D Tcast6

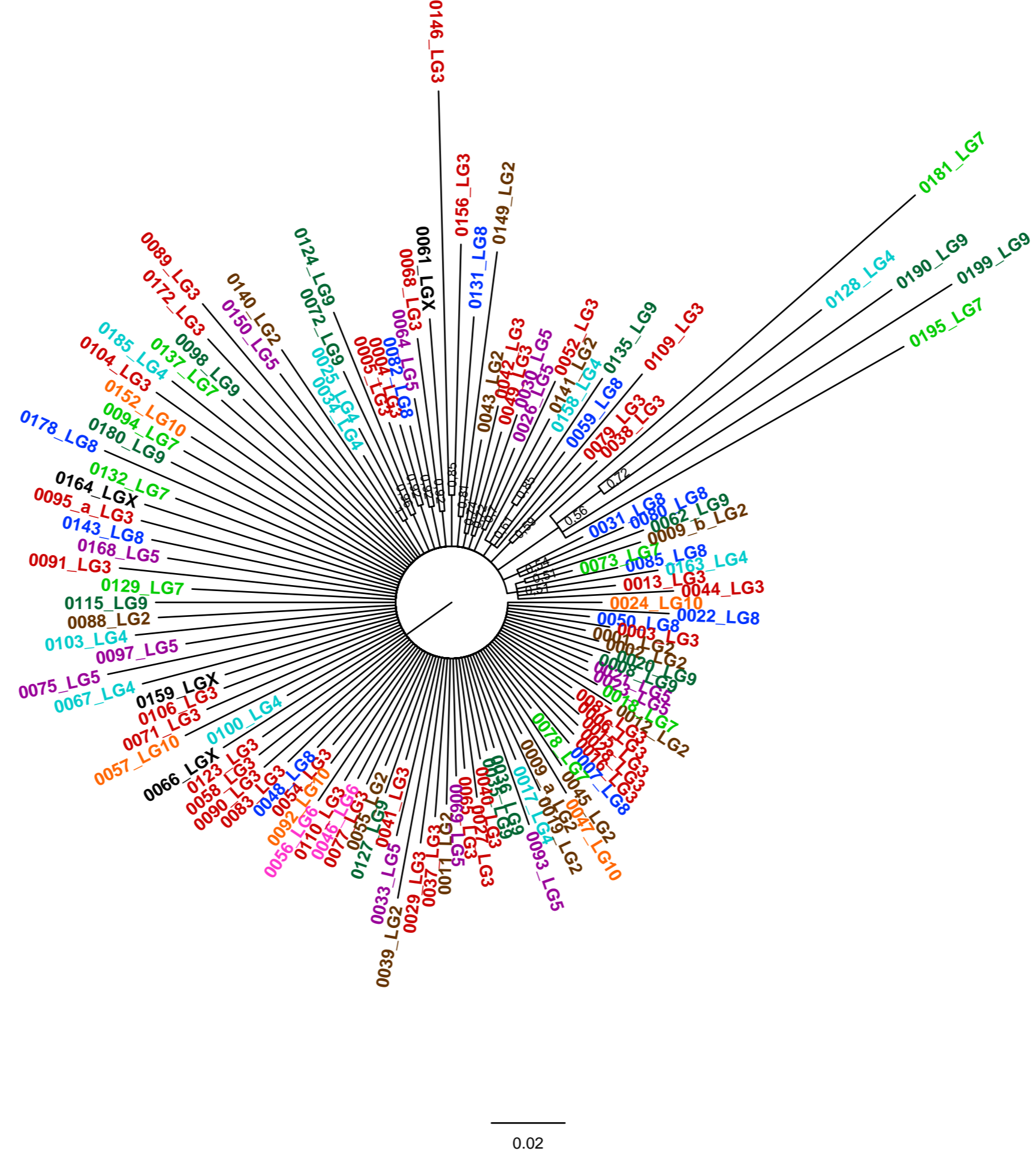

E Tcast7

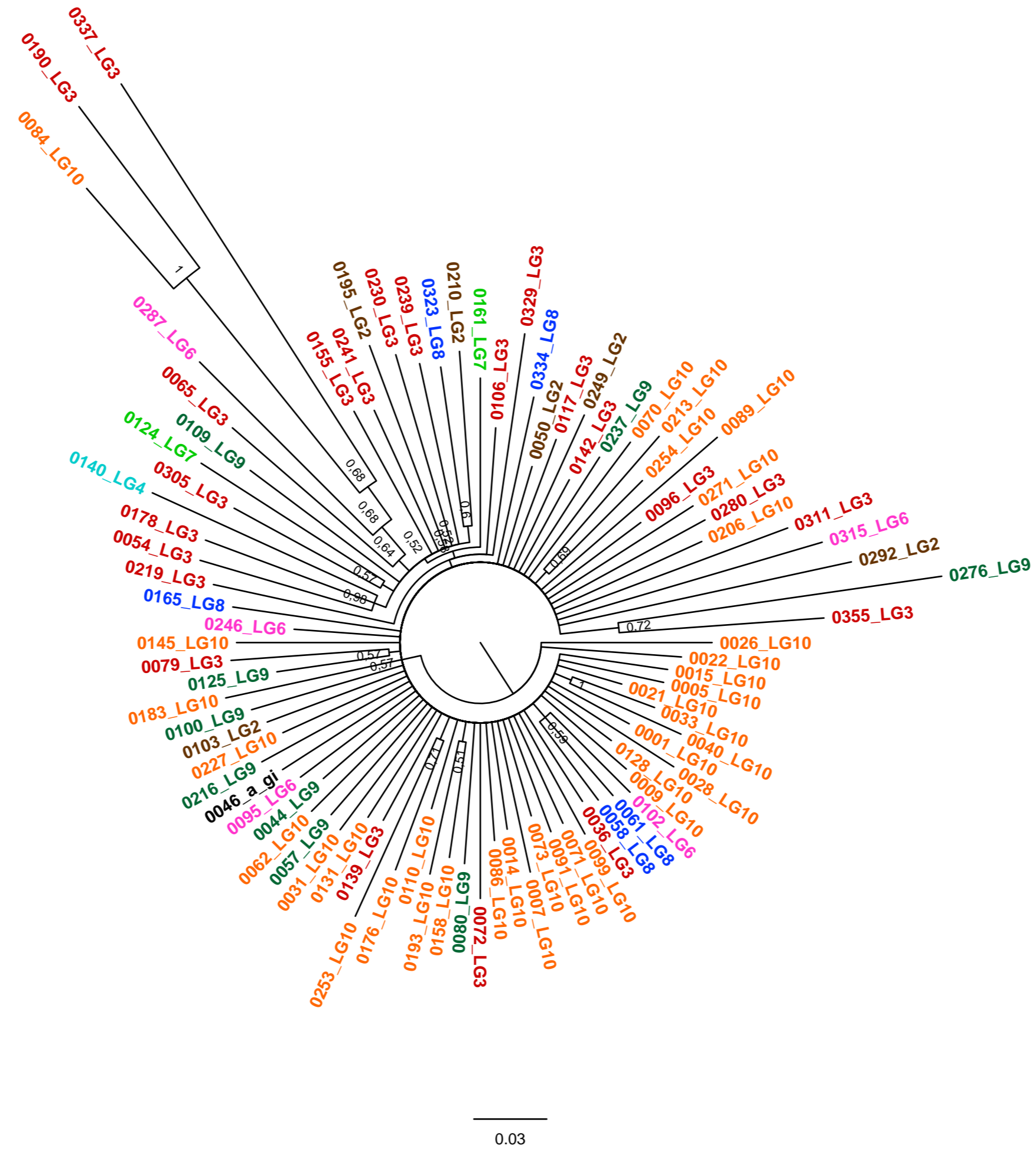

F Tcast8

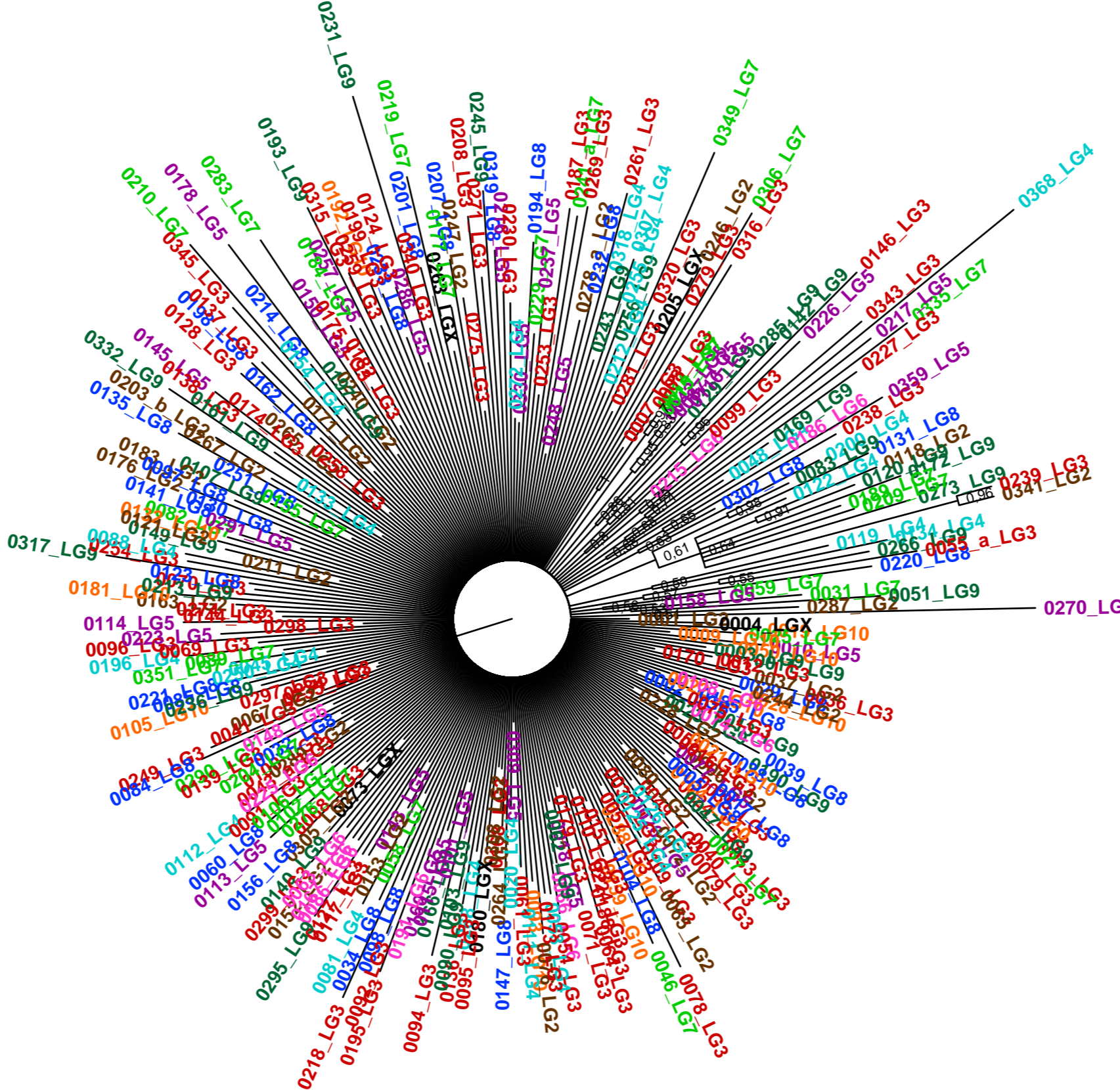

0.04

G Tcast9

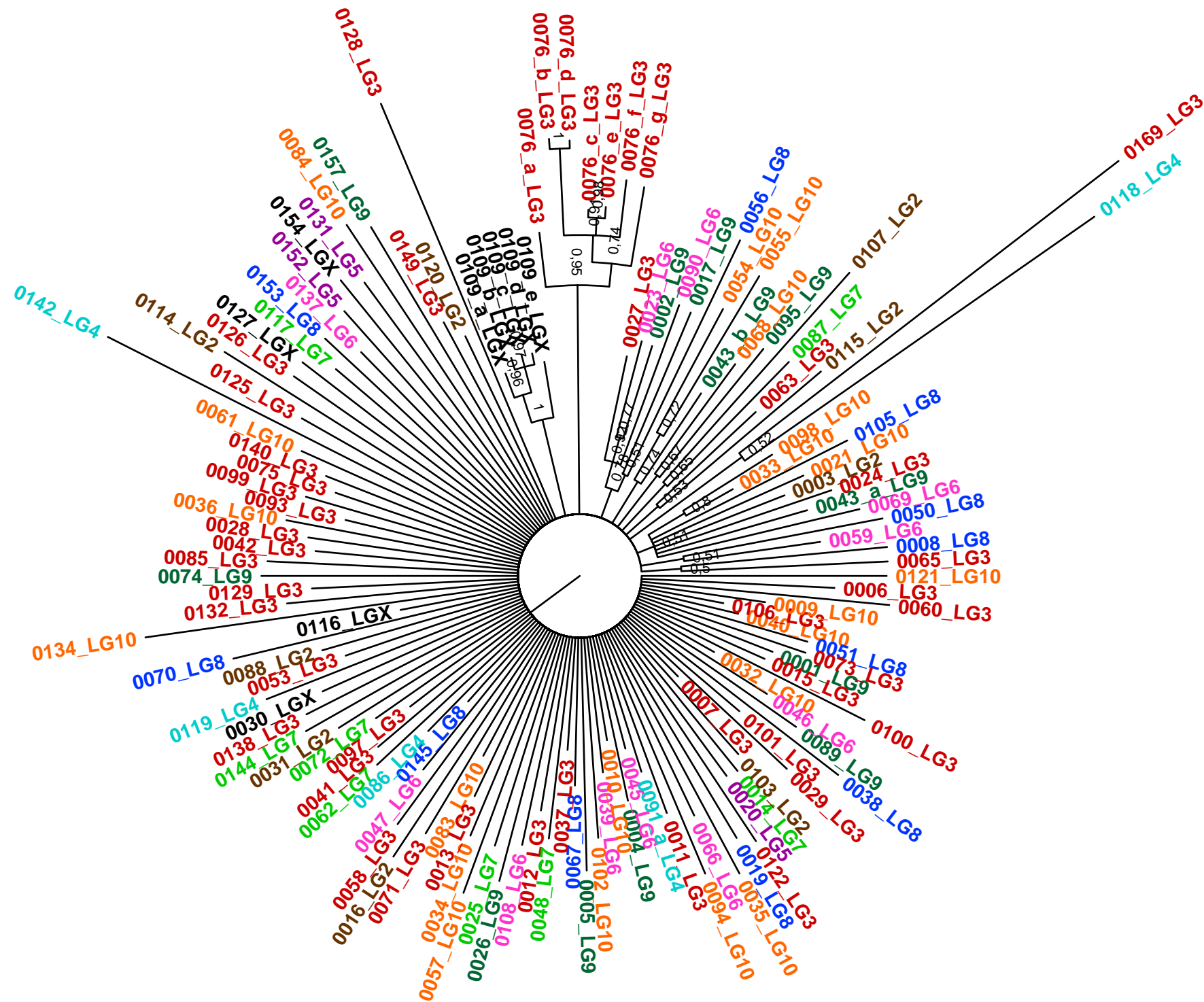

H Tcast10

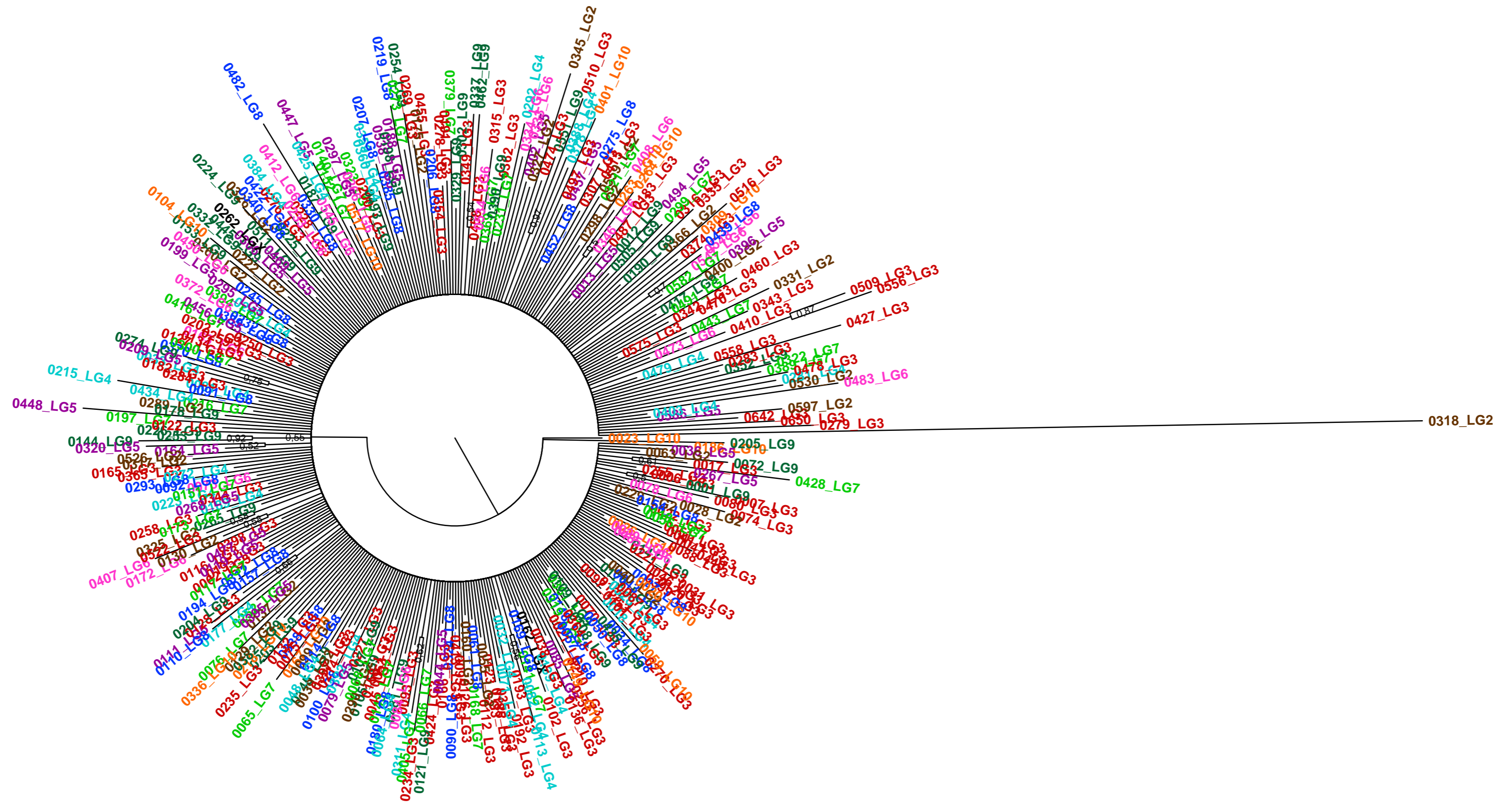

0.2

Supplement: Supplementary file 2 [file 875FileS2.pdf]
