# Supplementary material for: Dispersion Profiles and Gene Associations of Repetitive DNAs in the Euchromatin of the Beetle Tribolium castaneum
Source: G3 (Bethesda). 2018 Jan 8;8(3):875–86. doi: 10.1534/g3.117.300267 (PMC5844308; doi:10.1534/g3.117.300267)
Supplement: Supplementary file 3 [file 875FileS3.doc]

**Table S1**. List of overlapping TCAST elements.

| **overlapping elements** | **coordinates** |
| --- | --- |
| TCAST3, TCAST3 | LG10:9420636-9422205, LG10:9420730-9420917 |
| TCAST5, TCAST10 | LG2:375333-376892, LG2:376126-376352 |
| TCAST6, TCAST5 | LG2:5695057-5697923, LG2:5695845-5696252 |
| TCAST5, TCAST10 | LG3:27076318-27076530, LG3:27076529-27076754 |
| TCAST5, TCAST10 | LG4:13542809-13542998, LG4:13542995-13543640 |
| TCAST5, TCAST8 | LG7:12180511-12181376, LG7:12180861-12181153 |
| TCAST5, TCAST10 | LG9:2176865-2178430, LG9:2177832-2178032 |
| TCAST2, TCAST1 | LG9:5501177-5501476, LG9:5501465-5501622 |
| TCAST4, TCAST10 | LG9:10515479-10516496, LG9:10515695-10515918 |

**Table S2**. List of primers used for PCR amplification of TCAST repeats.

| **TCAST element** | **Primers** |
| --- | --- |
| **TCAST3** | aF GCTCTCAACAGGTGGGTT |
|  | aR ACTGTGTGGCTTATGGTAGTT |
|  | bF TGGTCCACTACGTTATTCTTC |
|  | bR GAAGTTAATTTACCTCTGCTG |
| **TCAST4** | aF TACAGAAGACGGGAGCTG |
|  | aR GCTCAGTTATTAGTAAAGTTGG |
| **TCAST5** | aF TGCTCCAATAGAATGAGTGCCA |
|  | aR CTCCTTCGTCGTGCTCCAA |
| **TCAST6** | aF AGTTACCAGTAGCCGACCT |
|  | aR ACTGATCCCTTTGTTGGCA |
| **TCAST7** | aF GCGATAACATATTACTTGCAG |
|  | aR TGTTGATTTGTTATACCTCGG |
|  | bF TCGATATCTTTGACAGTGTG |
|  | bR AATGAATCAAATGGCACTGC |
| **TCAST8** | aF CGTGTCTTAATTTTAACAGGT |
|  | aR CGTATTTCTGATACACCCT |
| **TCAST9** | aF AGTAGGACTTTCACCACTT |
|  | aR CTTTTCTANGATTCTRDGT |
|  | bF TGCATGTTTCTAAAATATTGCG |
|  | bR GCAGAAACGCAGCTATAATC |
| **TCAST10** | aF TCTCGTAAACGGTAAATGTTATC |
|  | aR TGCAATAACCGATACATTTGACC |

**Table S3.** List of primers used for expression analysis of TCAST repetitive families.

| **TCAST element** | | **Primers** | |  |
| --- | --- | --- | --- | --- |
| **TCAST1** | | F CCATAAGCGAGTTATAGAGTTGG | |  |
|  | | R CTTTAGTGACTTTTATGTCTTCTCC | |  |
| **TCAST2** | | F TGAGTTTGAGTGTAAATCGGACGC | |  |
|  | | R GTTTGATTTATTGCGCCTCGTGG | |  |
| **TCAST3** | | F AGGAATTCGAACCTCCCACAC | |  |
|  | | R AAATGGAGATGGTGGCGCAT | |  |
| **TCAST4** | | aF AGTGTTAGGATATGCAATATGGTTG | |  |
|  | | aR CGAAGATGGCTCCAATCCTTT | |  |
|  | | bF GGATTGGAGCCATCTTCGACT | |  |
|  | | bR ACGTAAATTCTAAAACATTCGGAGT | |  |
| **TCAST5** | | F CGACGAAGGAGTGCTCCAAT | |  |
|  | | R TGTCAACAGCGTCAAGTCGT | |  |
| **TCAST6** | | F CCTTACCTGCTAGCCCATCG | |  |
|  | | R ACGCGTCTAGTTTGCGTCTA | |  |
| **TCAST7** | | F CCGAAAGATACCCCCGACAG | |  |
|  | | R TGCGTCTGCAGGCACATAAT | |  |
| **TCAST8** | | F ACCCGCCTATGGGTACAAAAA | |  |
|  | | R ACGAGCCGGTCAGTGTTTA | |  |
| **TCAST9** | | F GCTGTCCCAACGATCCGAAA | |  |
|  | | R AACATGCAGCAGAAACGCAG | |  |
| **TCAST10** | | aF CCAATCGATCCCATGTGTGC | |  |
|  | | aR GGGTAGCGTAGACGTGCATA | |  |
|  | | bF GCACGTCTACGCTACCCATA | |  |
|  | | bR TTTTGAGAGAAAATGCGACGTT | |  |
|  | | cF AAAGTCCCAATCGATCCCATGT | |  |
|  | | cR GGGTAGCGTAGACGTGCATATT | |  |
|  |  | |  |  |
|  |  | |  |  |
|  |  | |  |  |
|  |  | |  |  |
|  |  | |  |  |
|  |  | |  |  |
|  |  | |  |  |
|  |  | |  |  |
|  |  | |  |  |
|  |  | |  |  |

**Table S4.** Distribution of repetitive families TCAST1-TCAST10 as well as genes, along *T. castaneum* chromosomes. Number of TCAST1-10 elements and genes along the regions corresponding to 1/10 of each chromosome length is shown.

**Table S5.** TCAST elements overlapping exons

**Table S6.** A list of genes (NCBI-GeneIDs) associated with TCAST1 elements as well as a list of TCAST2-TCAST10 elements additionally associated with the genes. Presence of an additional TCAST element in the vicinity of a gene is indicated with a green rectangle. If more elements of a particular TCAST family are present, the number is indicated within the rectangle.
